# Mechanism of C-3 Acyl Neighboring Group Participation in Mannuronic Acid Glycosyl Donors

**DOI:** 10.1021/jacs.4c13910

**Published:** 2024-12-18

**Authors:** Frank F. J. de Kleijne, Peter H. Moons, Floor ter Braak, Hero R. Almizori, Luuk J. H. Jakobs, Kas J. Houthuijs, Giel Berden, Jonathan Martens, Jos Oomens, Floris P. J. T. Rutjes, Paul B. White, Thomas J. Boltje

**Affiliations:** † Department of Synthetic Organic Chemistry, Institute for Molecules and Materials, Radboud University, Heyendaalseweg 135, Nijmegen 6525 AJ, The Netherlands; ‡ FELIX Laboratory, Institute for Molecules and Materials, Radboud University, Toernooiveld 7, Nijmegen 6525 ED, The Netherlands

## Abstract

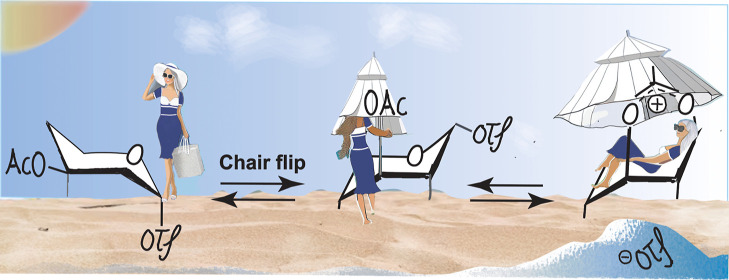

One of the main challenges in oligosaccharide synthesis is the stereoselective introduction of the glycosidic bond. In order to understand and control glycosylation reactions, thorough mechanistic studies are required. Reaction intermediates found by NMR spectroscopy often cannot explain the glycosylation’s stereochemical outcome. Hence, reactions may proceed through low-abundance reaction intermediates that are difficult to detect, according to a Curtin–Hammett scenario. We have previously observed that manno-type sugars can engage in C-3 acyl neighboring group participation. Herein, we report the detection of glycosyl dioxanium ions that result from C-3 neighboring group participation in mannuronic acid donors. Using a suite of exchange NMR techniques, we were able to dissect the kinetics of the conformational ring-flip that precedes C-3 acyl participation and the participation event itself in various manno-type sugars. Hence, this study provides a complete picture of mannosyl dioxanium ion formation.

## Introduction

A major challenge in oligosaccharide synthesis is controlling the stereoselectivity of the chemical glycosylation reaction. In this reaction, a glycosidic bond is formed when an electrophilic glycosyl donor reacts with a nucleophile, the glycosyl acceptor. The nucleophile can attack from either the α-face or the β-face of the glycosyl donor, yielding the corresponding α- or β-glycoside. A well-known and reliable way to direct the facial selectivity of the glycoside-forming step is the use of neighboring group participation (NGP). The most well-established example is the incorporation of a C-2 acyl protecting group into the glycosyl donor. Upon activation of glycosyl donor, the glycosyl cation is stabilized by C-2 acyl NGP. The resulting *cis*-fused bicyclic dioxolanium ion intermediate reacts in a stereospecific manner with glycosyl acceptor to form 1,2-*trans* glycosides.^1,2^ Other forms of intramolecular stabilization, such as NGP by C-3, C-4, or C-6 acyl protecting groups, have long been suggested to impact stereoselectivity in glycosylation reactions, but whether this can be ascribed to NGP or to stereoelectronic effects has been a subject of debate.^3−5^ The reaction intermediates that arise from C-3, C-4, and C-6 acyl NGP are likely highly reactive and short-lived, making them exceedingly difficult to detect and characterize.

Recently, we and others^6−16^ showed that infrared ion spectroscopy (IRIS) can be used to characterize such unstable glycosylation reaction intermediates in the gas phase. Glycosyl cations were generated using a tandem mass spectrometry (MS/MS) scheme, collected in an ion trap, and characterized by infrared multiple-photon dissociation (IRMPD) spectroscopy.^6^ The resulting IR spectra were assigned using density functional theory (DFT)-computed IR reference spectra. A large number of glycosyl cation structures were elucidated using this method.^7−16^ Markedly, these experiments highlighted that C-3 acyl NGP was most pronounced in manno-type sugars. As C-3 acyl-protected mannosyl donors are known to provide α-products, we explored the detection of C-3 mannosyl dioxanium ions under relevant glycosylation conditions (solution phase) using variable-temperature (VT) NMR. In the solution phase, we expected a very low dioxanium ion population due to a rapid equilibrium with the more stable α-glycosyl triflate intermediate. We utilized this chemical equilibrium to detect 1,3-bridged mannosyl dioxanium ions using chemical exchange saturation transfer (CEST) NMR. This proved that C-3 acyl NGP is possible under relevant conditions and is likely responsible for α-product formation via a Curtin–Hammet scenario.^17^ Next, we demonstrated C-3 NGP is also possible in C-4,6 benzylidene-protected mannosides^18^ and rhamnosyl donors (6-deoxy-l-mannose).^14^ In addition, the interconversion kinetics of the α-triflate and the low-abundant reactive intermediates could be derived using ^1^H and ^13^C CEST NMR and ^19^F exchange spectroscopy (^19^F EXSY) NMR.^14,17−21^ In contrast, the analogous C-3 acylated glucosyl donor did not show C-3 dioxanium ion formation upon activation nor glycosylated with a high α-selectivity.

Computational studies attributed this glucoside-mannoside difference to the conformational changes required for 1,3-dioxanium ion formation. In the case of glucosides, significant flagpole interactions between O-2 and H-5, as well as the unfavorable orientation of the anomeric triflate, discourage glucosyl dioxanium ion formation. In the case of mannosides, the flagpole interactions between H-2 and H-5 are less severe, and the anomeric triflate position allows for an interaction between the triflate and the H-2 and H-5 protons.^8^ However, experimental evidence supporting this hypothesis is lacking. Therefore, it remained unclear whether glycosyl dioxanium ion formation proceeds via anomeric triflate dissociation and trapping of the glycosyl cation or if the α-glycosyl triflate ring-flips to its ^4^C_1_-chair conformer to facilitate C-3 NGP from an axial position.

**Figure 1:**
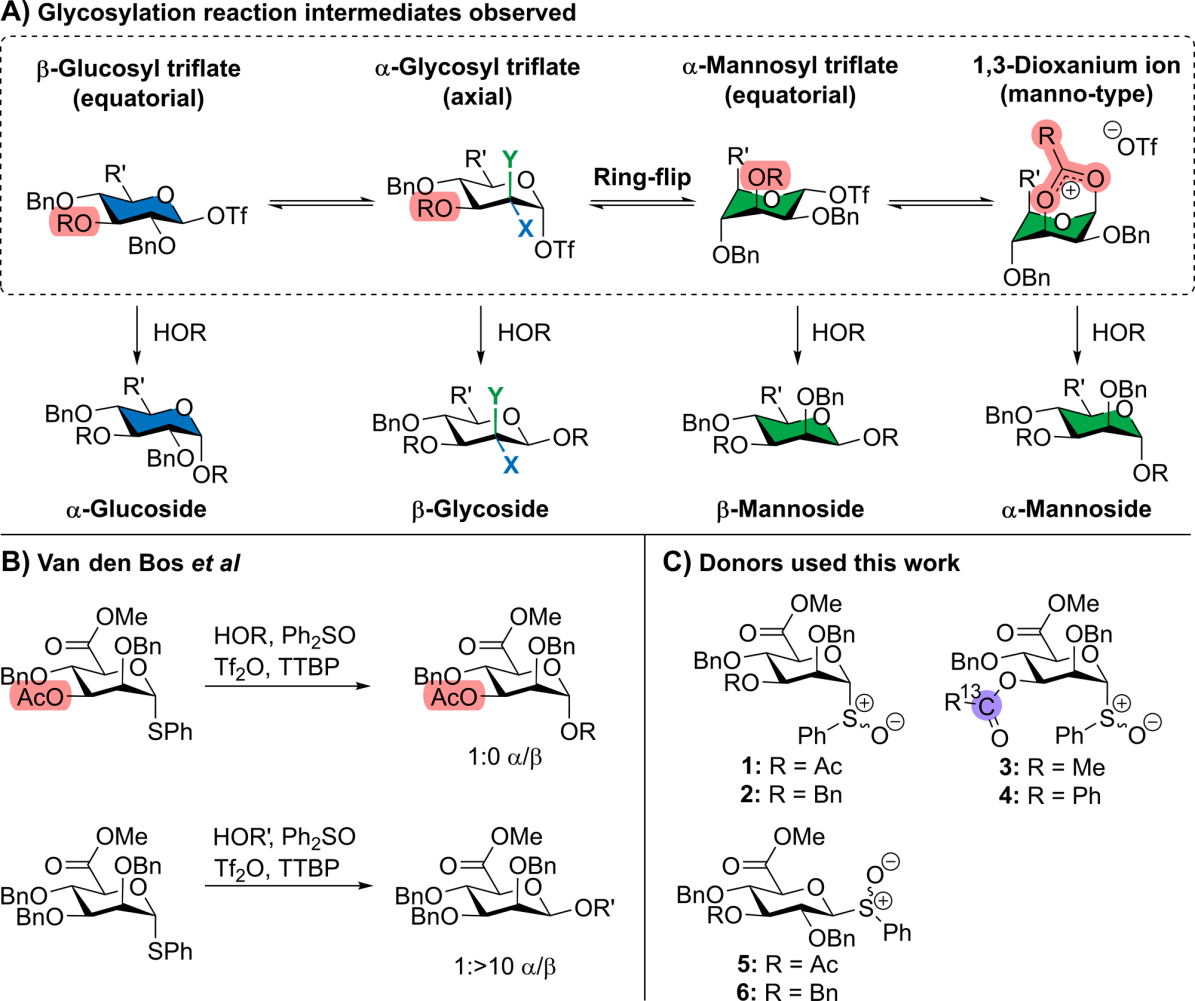
Glycosylation reaction mechanism of uronic acids. (A) Glycosylation reaction intermediates observed in this work. Gluco-type sugars are displayed in blue, and manno-type sugars are displayed in green; (B) stereoselective glycosylations reported by Van den Bos et al.; (C) C-3 acylated and benzylated mannuronic acid derivatives and glucuronic acid derivatives were used in this work. d-Mannose, R′ = CH_2_OBn, d-mannuronic acid, R′ = CO_2_Me. Y = H, X = OBn, Gluco. Y = OBn, X = H, Manno.

Herein, we report the investigation of C-3 NGP in mannuronic acid donors (Figure 1). The goal of this was 2-fold. First, mannuronic acids protected with non-participating protecting groups are known to glycosylate with a very high β-selectivity.^22−25^ However, upon the introduction of a C-3 acyl group, complete α-selectivity was observed, similar to that of the related mannose and rhamnose systems (Figure 1B). This suggests that C-3 acyl NGP also takes place in mannuronic acid derivatives, which has been proposed but never demonstrated experimentally.^24,26,27^ Second, glycosyl triflate intermediates derived from mannuronic acid donors are known to be conformationally flexible and equilibrate between the ^1^C_4_ and ^4^C_1_ conformers (Figure 1A).^28,29^ We wanted to investigate whether C-3 NGP occurs from the ^1^C_4_ conformer rather than the ^4^C_1_ conformer, thereby providing an additional understanding of the C-3 NGP pathway. Using a combination of ^1^H CEST, ^13^C CEST, and ^19^F EXSY NMR, we demonstrate that mannuronic acid donors can engage in C-3 acyl NGP. We establish that the reactive ^1^C_4_ conformer affords the dioxanium ion. In addition, we show that this also occurs in mannosylations and rhamnosylations. Lastly, we measured the exchange kinetics of the dioxanium ion and glycosyl triflates, thus allowing us to rationalize the observed glycosylation stereoselectivity and provide evidence of the participating mechanism in C-3 acyl-protected manno-type sugars.

## Results and Discussion

### Glycosyl Donor Synthesis

To enable the characterization of reaction intermediates derived from C-3 ether- or ester-protected glucuronic and mannuronic acid esters with NMR and IRIS, we prepared glycosyl sulfoxide donors **1**–**6**. Glycosyl sulfoxides are versatile precursors as they can provide reactive intermediates using collision-induced dissociation (CID) for the IRIS experiments^9^ or by activation in solution using triflic anhydride (Tf_2_O) in the case of NMR experiments.^30,31^

**Scheme 1:**
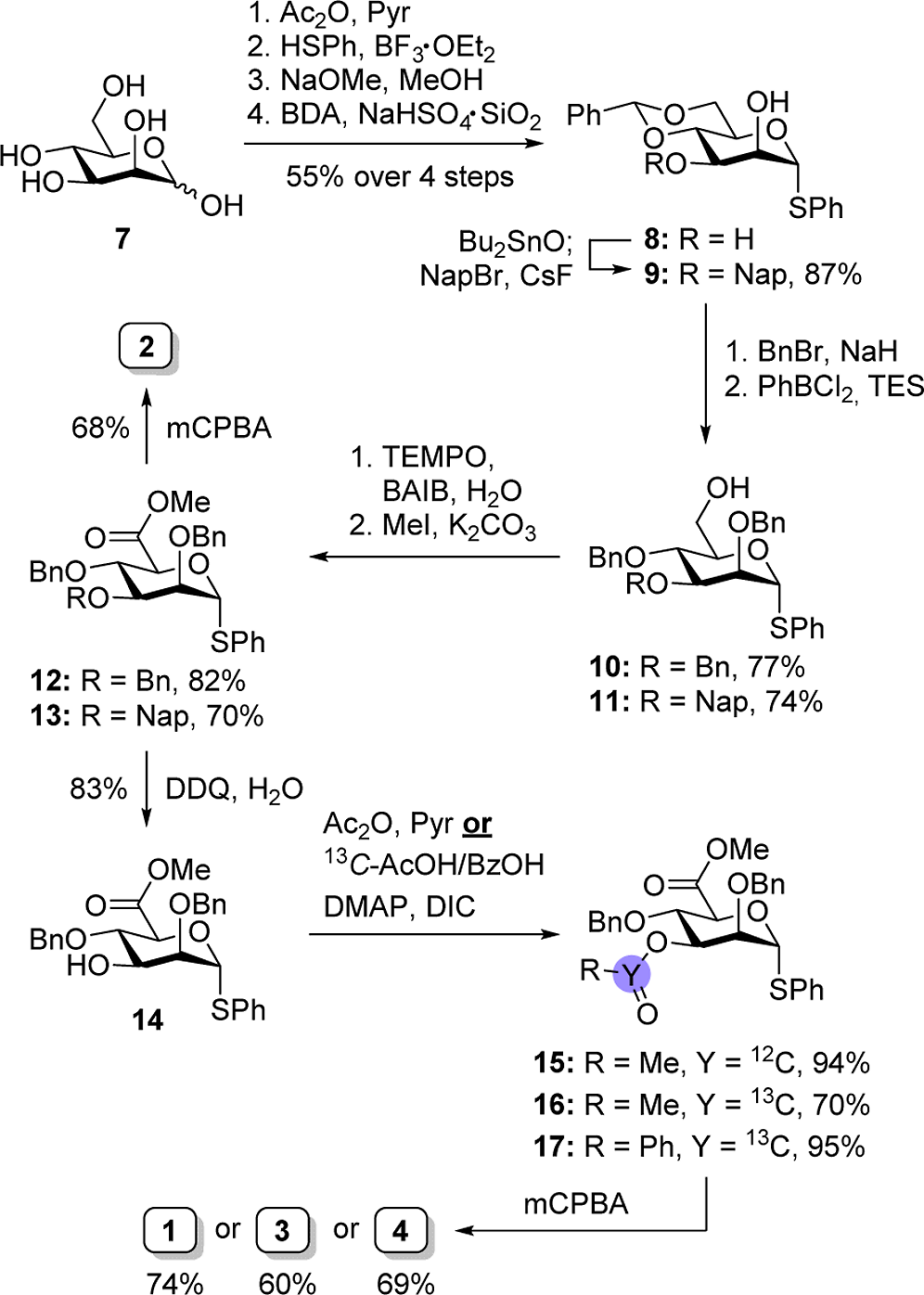
Synthesis of Mannuronic Acid Donors **1**–**4**

The synthesis of mannuronic acid precursor **1**–**4** started with the peracetylation of d-mannose (**7**) using acetic anhydride (Ac_2_O) in pyridine (Scheme 1). A subsequent reaction with thiophenol and BF_3_·OEt_2_ in dichloromethane (DCM) afforded the corresponding thioglycoside, which was deprotected using Zemplén deacetylation to afford thioglycoside. Next, the 4,6-benzylidene acetal was installed with silica-supported NaHSO_4_ (NaHSO_4_·SiO_2_) as an acid catalyst in MeCN.^32^ Increasing the concentration of the reaction mixture to 0.20 M greatly enhanced the reaction efficiency, as thioglycoside product **8** precipitated, thereby preventing additional benzylidene formation at C-2/C-3. Central intermediate **8** was used to obtain various C-3 protected building blocks. Benzylation afforded 2,3-di-*O*-benzyl-protected derivative **10**, while selective protection employing stannylene acetal-mediated chemistry afforded 2-methylnaphthyl (Nap) derivative **9**, which afforded orthogonally protected mannoside **11** after benzylation. Both molecules were converted into mannuronic acid esters by a sequence of reductive benzylidene opening using PhBCl_2_ and triethylsilane (TES), chemo- and regioselective oxidation of the primary alcohol with 2,2,6,6-tetramethylpiperidinyloxyl (TEMPO) and bis(acetoxy)iodobenzene (BAIB), and finally methylation of the uronic acids to yield methyl esters **12** and **13**.^33,34^ To afford C-3 acyl-protected derivatives, the C-3 Nap ether was oxidatively cleaved using DDQ in DCM/H_2_O.^35^ At this stage, the C-3 participating acetyl group was installed. To increase the sensitivity of the ^13^C NMR experiments, ^13^C-enriched acetyl and benzoyl ester derivatives **16** and **17** were also prepared using a Steglich esterification.^36^ Finally, glycosyl sulfoxide derivatives **1**–**4** were obtained by treatment with a stoichiometric amount of *meta*-chloroperoxybenzoic acid (*m*CPBA) in DCM at low temperature.^37^ The glucuronic acid ester derivatives **5** and **6** were synthesized via an analogous procedure (please see Supporting Information).

### NMR Experiments

NMR spectra of various mannuronic acid derivatives shown in Scheme 1, in particular, those with bulky benzyl or naphthyl groups, displayed broad ^13^C NMR resonances or broadened into the baseline. Typically, this type of signal broadening is suggestive of conformational isomerism. Therefore, the NMR measurements were performed at an elevated temperature (50 °C) in order to obtain defined and sharp spectra (Supporting Information Figures S3 and S4). Moreover, clear differences between the diastereomers of sulfoxide derivatives **1** and **2** were observed, which could be separated by column chromatography. Based on scalar couplings (^3^*J*_H–H_), ^1^H NMR analysis demonstrated that one diastereomer adopted a ^1^C_4_-chair conformation (**1**_**eq**_ and **2**_**eq**_), while the other diastereomer adopted a ^4^C_1_-chair conformation (**1**_**ax**_ and **2**_**ax**_) (Supporting Information Figures S5–S6). Unfortunately, we were unable to determine the orientation of the sulfinyl stereocenter, as the compounds were isolated as oils. Notably, this contrasting conformational behavior was not detected in the analogous glucuronic acid, mannose, and rhamnose derivatives used in this study.

To enable the detection of dioxanium ions derived from uronic acid ester donors using NMR, the glycosyl sulfoxide donors were activated using Tf_2_O in the presence of the non-nucleophilic base 2,4,6-tri*-tert*-butylpyrimidine (TTBP) in CD_2_Cl_2_ at −80 °C. All donors displayed clean activation into their α-glycosyl triflate intermediate, yet clear differences in conformational behavior were observed (Supporting Information Figures S7–S15). After activation, glucuronic acid derivatives **5** and **6** formed an axial α-glycosyl triflate in the ^4^C_1_ conformation as the main species irrespective of its substitution at C-3 (Figures S16–S21). In contrast, mannuronic acid donors **1**–**4** produced two conformers of the α-glycosyl triflate species, namely, an axial α-glycosyl triflate (^4^C_1_ conformer) and equatorial α-glycosyl triflate (^1^C_4_ conformer). Notably, this type of conformational behavior has previously been reported for 2-*O*-benzyl, 2-azido, and 2-fluoro derivatives of methyl 4-*O*-acetyl-3-*O*-benzyl-d-mannuronate before by Walvoort et al. and was shown to be affected by C-2 substitution.^28,29^ In our case, C-3 benzyl derivative **2** produced an equimolar conformational mixture (^4^C_1_/^1^C_4_ ≈ 1:1), whereas C-3 acetyl derivative **1** resulted in a reduced population of the ^1^C_4_ conformer (^4^C_1_/^1^C_4_ ≈ 2:1). We expected the α-selective glycosylations with the C-3 acylated mannuronic acid derivatives to arise from reactions with 1,3-bridged dioxanium ions, similar to those observed in mannose and rhamnose.^14,17,18^ Moreover, we envisioned that the axial α-glycosyl triflate (^4^C_1_) first required a ring-flip into its equatorial conformer (^1^C_4_) in order to bring the C-3 ester in the axial position to enable NGP and concurrent displacement of the triflate.

**Figure 2:**
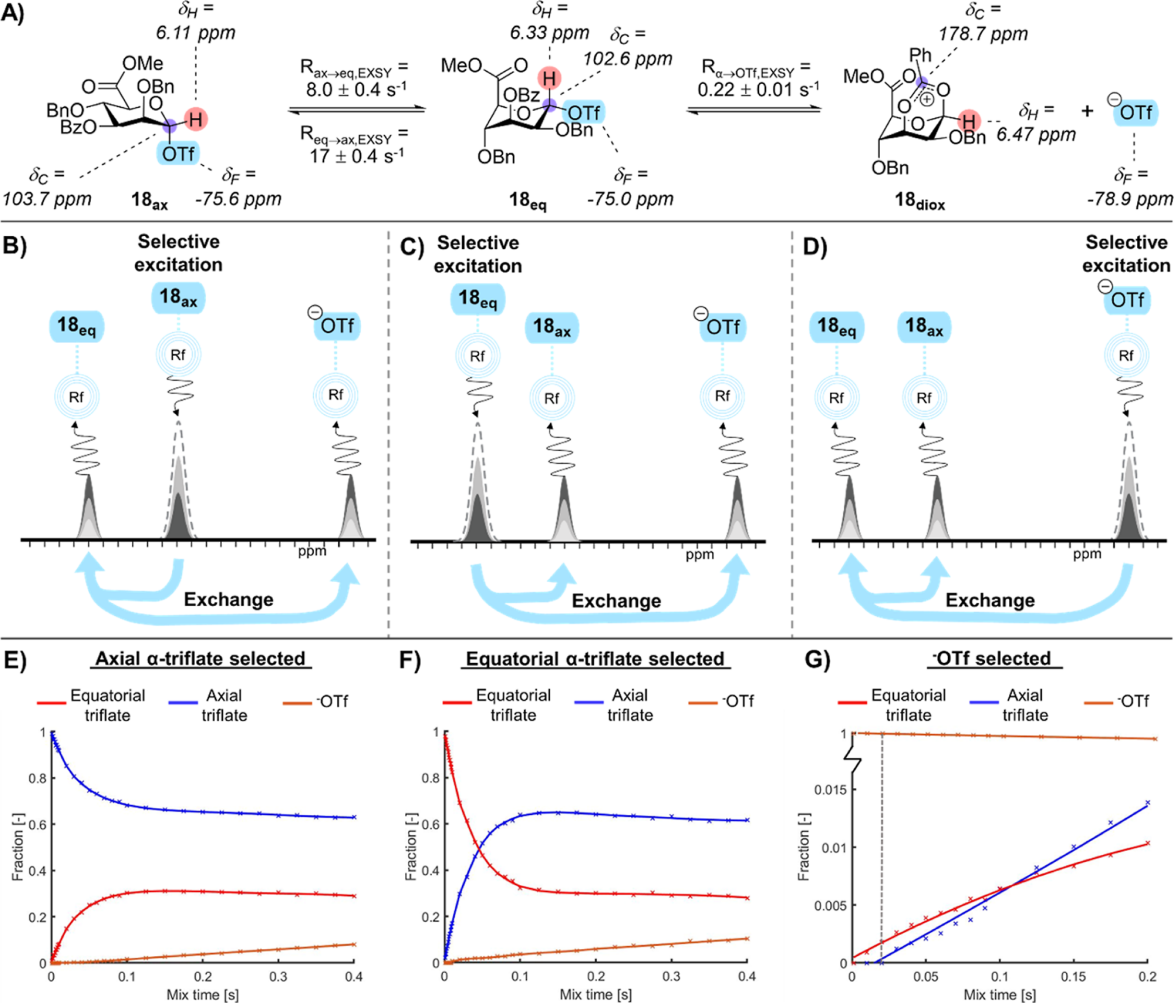
NMR experiments on activated mannuronic acid **4**. **4** was preactivated using Tf_2_O/TTBP. (A) Observed glycosylation reaction intermediates; (B,E) selective excitation of **18**_**eq**_ followed by magnetization transfer to **18**_**ax**_ and ^–^OTf; (C,F) selective excitation of **18**_**ax**_ followed by magnetization transfer to **18**_**eq**_ and subsequently ^–^OTf; and (D,G) selective excitation of ^–^OTf followed by magnetization transfer to **18**_**eq**_ and subsequently **18**_**ax**_.

Therefore, mannuronic acid α-glycosyl triflate interconversion was studied at −80 °C. Since both axial or equatorial α-glycosyl triflate derivatives of the mannuronates **1** – **4** were readily observable at equilibrium (Figure 2A; Supporting Information Figures S7–15), ^19^F EXSY experiments could be used to extract their conformational interconversion kinetics (Tables S2 and S3). A narrow excitation pulse was applied to either the equatorial or the axial α-glycosyl triflate ^19^F resonance. As these conformers are in chemical exchange with each other, the degree of magnetization transfer to each species can be measured in order to obtain the interconversion rate (Figure 2B–D).^18^ Interestingly, ring-flipping from the axial α-triflate to the equatorial α-triflate was much faster for the C-3 benzyl derivative compared with those of the corresponding C-3 acetyl derivative. Ring-flipping from the equatorial α-triflate to the axial α-triflate proceeded with comparable rates (Tables S2 and S3). Notably, 10% conversion of the selected resonance to its ring-flipped resonance occurred within 10 ms, nearing the limits for an exchange rate measurable with EXSY NMR using initial rate conditions. Moreover, magnetization transfer to the ^–^OTf resonance was observed after exciting the equatorial α-glycosyl triflate resonance of the C-3 acetyl mannuronic acid. Markedly, this was not observed when selecting the axial α-glycosyl triflate within a mixing time of 10 ms. These observations suggest that the equatorial α-glycosyl triflate more readily dissociates its triflate compared to the axial α-glycosyl triflate, which is consistent with the hypothesis that the equatorial α-glycosyl triflate is the reactive conformer that induces triflate dissociation through C-3 NGP.

To gain further mechanistic insight, the fraction of magnetization transfer from selective excitation of either the axial α-glycosyl triflate, the equatorial α-glycosyl triflate, or unbound triflate was plotted as a function of the mix time by incrementing mix times (0–400 ms) (Figure 2B–G). When the axial and equatorial α-glycosyl triflate resonances were used as inputs for the EXSY experiment, equilibrium was established after a mixing time of 100 ms (Figure 2E,F). Interestingly, magnetization transfer to the ^–^OTf was also observed for the axial triflate at longer mix times (>30 ms). Faster release of ^–^OTf by the equatorial α-triflate was observed compared to the axial α-glycosyl triflate (see Supporting Information, Figure S22). Plotting the fraction of free triflate resulting from selecting either the equatorial or axial triflate as a function of the mix time indeed confirmed a faster release of the free triflate when the equatorial triflate was selected (see Supporting Information, Figure S22). Plausibly, the axial α-glycosyl triflate first ring-flips to a ^1^C_4_ conformation before it releases ^–^OTf as a result of C-3 NGP. Hence, this process only becomes apparent at longer mixing time (>30 ms). Again, these observations were consistent with the hypothesis that C-3 NPG induces triflate dissociation via the equatorial α-glycosyl triflate. Finally, excitation of ^–^OTf first leads to the formation of the equatorial α-triflate, followed by conversion into the axial α-glycosyl triflate at longer mixing times (>30 ms) (Figure 2G). This is consistent with a pathway involving nucleophilic attack of ^–^OTf on the dioxanium ion, affording the equatorial α-glycosyl triflate, which rapidly interconverts into the axial α-glycosyl triflate.

### Conformational Behavior of Other Manno-Type Sugars

We previously reported the characterization of mannosyl and rhamnosyl dioxanium ions resulting from C-3 acyl NGP. Likewise, we expected the formation of an equatorial α-glycosyl triflate prior to C-3 NGP. This translates to a ^1^C_4_ or ^4^C_1_ conformer for d-mannose and l-rhamnose, respectively. However, these species were not detectable at equilibrium, hence excluding the use of EXSY because it requires interconverting species to be visible in 1D NMR. Instead, we used CEST NMR, which is a powerful technique for detecting “invisible” species.^17^ CEST enhances the sensitivity to low-abundance reaction intermediates by saturating at a given frequency while monitoring the saturation transfer via chemical exchange to a readily observable resonance, which is typically the axial α-triflate or free triflate. Once a low-abundant reaction intermediate is saturated, it causes a decay in the observable resonance.^19^

**Figure 3:**
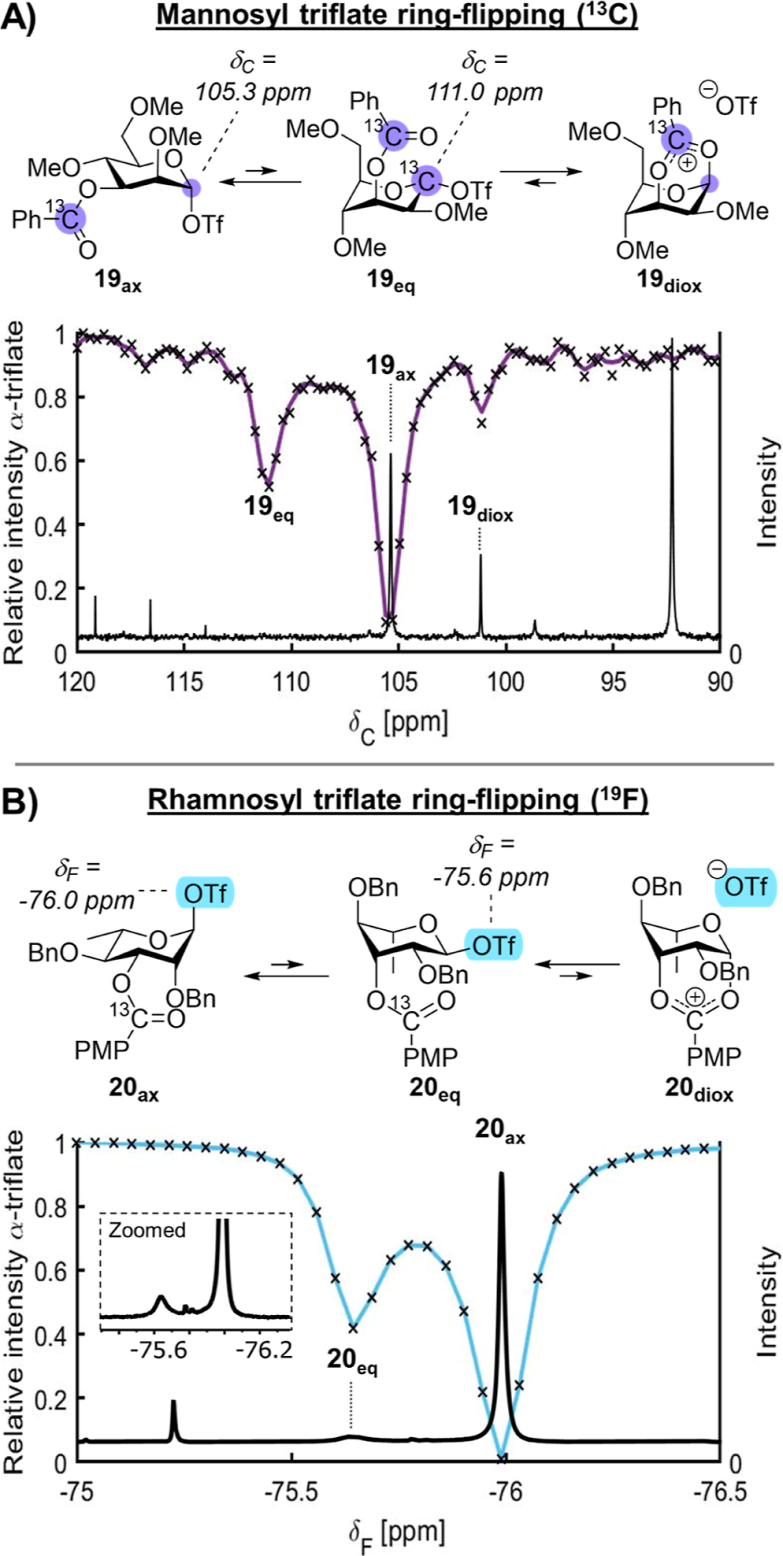
(A) Studying the presence of ring-flipped mannosyl α-triflates using ^13^C CEST NMR; (B) studying the presence of ring-flipped rhamnosyl α-triflates using ^1^H CEST NMR.

Given that the ring-flipped equatorial α-triflate **18**_**eq**_ is the key intermediate resulting in C-3 acyl participation, we set out to investigate a similar mechanism in d-mannose and l-rhamnose glycosyl donors. In the case of mannose, a broad anomeric ^13^C resonance was previously observed for the mannosyl α-triflate **19**_**ax**_ (δ_C_ = 105 ppm), whereas the anomeric ^13^C resonance of the corresponding dioxanium ion (**19**_**diox**_) remained a sharp doublet (δ_C_ = 101 ppm, Figure S23). This suggests that the axial α-glycosyl triflate was in a faster chemical exchange with an intermediate other than the dioxanium ion. No evidence for the oxocarbenium ion was found in a 1D ^13^C NMR spectrum nor with ^13^C CEST NMR. We have also shown that equatorial β-glycosyl triflates can be detected using exchange NMR, although the latter was not observed to be in chemical exchange with α-triflate **19**_**ax**_ through ^19^F EXSY experiments. Moreover, for **19**_**ax**_, triflate dissociation was shown to proceed exclusively via a unimolecular mechanism. Instead, we reasoned that we could investigate the presence of an equatorial α-glycosyl triflate using ^13^C, ^1^H, and ^19^F CEST experiments. Unfortunately, the latter two techniques did not provide any evidence of other low populated intermediates. However, ^13^C CEST clearly displayed the presence of a transient intermediate at δ_C_ = 111 ppm that is in chemical exchange with the axial α-glycosyl triflate (Figure 3A). It is unlikely that the chemical shift observed in ^13^C CEST NMR δ_C_ = 111 ppm corresponds to an equatorial β-glycosyl triflate given that the rate of triflate dissociation is independent of the unbound triflate concentration.^18^ Therefore, we propose that the intermediate associated with this chemical shift is the ring-flipped equatorial α-glycosyl triflate in ^1^C_4_-chair **19**_**eq**_.

In the case of rhamnoside **20**, ^1^H and ^19^F CEST NMR were conducted as these techniques do not require an anomerically ^13^C-labeled substrate. ^1^H CEST only revealed that the axial α-glycosyl triflate was in chemical exchange with the dioxanium ion **20**_**diox**_ (δ_H_ = 6.46 ppm), which was also evident from our previous studies (Supporting Information Figure S24).^14^ In contrast, the ^19^F CEST profile did display a clear CEST signal corresponding to a low-abundant intermediate that was in chemical exchange with the axial α-rhamnosyl triflate **20**_**ax**_ and the unbound triflate ion resonance at δ_F_ = −75.6 ppm (Figure 3B). We therefore measured the rate at which **20**_**ax**_ (δ_F_ = −76 ppm) became the species with a resonance at δ_F_ = −75.6 ppm using ^19^F CEST NMR (Supporting Information page S56–S57). This was established to be 4.2 ± 0.1 s^–1^, which is substantially faster than the rate of the axial α-triflate becoming the equatorial β-triflate (0.10 s^–1^).^14^ Hence, based on both the ^19^F CEST profile and the exchange kinetics, we expect the signal observed at δ_F_ = −75.6 ppm in the ^19^F CEST profile and the 1D ^19^F NMR to correspond to the ring-flipped equatorial α-rhamnosyl triflate (^4^C_1_-chair conformer, **20**_**eq**_) as the equatorial to axial α-triflate exchange rate is much faster than triflate dissociation that results from β-triflate formation.

Differences in detection between different CEST experiments (^1^H, ^13^C, and ^19^F) can be explained by the requirements of the CEST viewing window, which requires the chemical shift difference (Δϖ) between two exchanging species to be larger than their chemical exchange rate (Δϖ > *k*_1_ + *k*_–1_).^38^ Hence, reaction intermediates may be detectable in ^19^F CEST NMR if Δϖ > *k*_1_ + *k*_–1_ but undetectable in ^1^H CEST NMR if Δϖ < *k*_1_ + *k*_–1_. In addition, the viewing window is affected by factors that influence the exchange rate, such as the type of glycoside, stereoelectronic effects, and the protecting group pattern. For example, the C-3 benzylated counterpart **22**_**ax**_ displayed a much slower triflate dissociation compared to conformer interconversion. Hence, the system completely equilibrates first upon selection of either conformer before the triflate ion is dissociated, prohibiting the tracing of its origin with ^19^F EXSY.

### Studying Glycosyl Triflate Reactivity and Exchange Mechanism Using ^19^F EXSY

**Figure 4:**
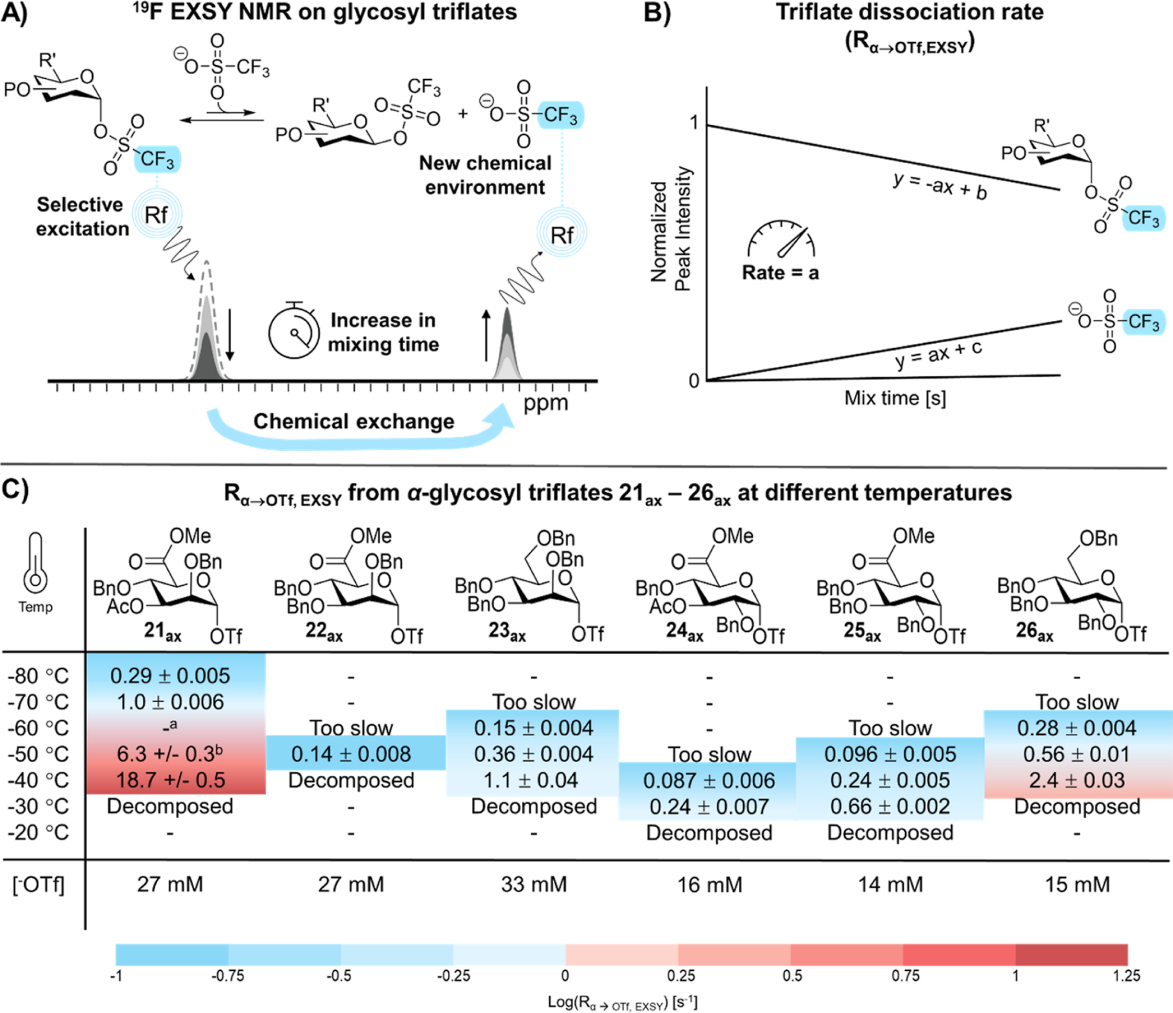
Triflate dissociation kinetics measured with 19F EXSY NMR. (A) Graphical visualization of ^19^F EXSY NMR in glycosyl triflates; (B) plotting the degree of magnetization transfer against the mix time affords R_α→OTf, EXSY_; and (C) triflate dissociation rates for mannosyl and glucosyl α-triflates determined by ^19^F EXSY as a function of temperature. ^a^Exchange between axial and equatorial α-triflates was in the intermediate fast exchange regime (Figure S25). ^b^Data was recorded using a 300 MHz magnet instead of a 500 MHz magnet.

Recently, we introduced an NMR workflow that was used to unravel the triflate dissociation mechanism of a series of mannosides and glucosides.^18^ Now, we applied this workflow to preactivated glycosyl donors **1**, **2**, **5**, and **6** in order to gain insights on their exact glycosylation reaction mechanism. The armed perbenzylated α-glycosyl triflates **23**_**ax**_ and **26**_**ax**_ were used as a benchmark. First, we measured the triflate dissociation kinetics at different temperatures, which can be done accurately from approximately 0.1 s^–1^ to 100 s^–1^ (Figure 4). For this experiment, all possible α-triflate resonances were excited simultaneously by making use of a broad excitation pulse. This enabled measurement of the overall triflate dissociation rate from the glycosyl triflate intermediates (R_α→OTf, EXSY_). Markedly, the gluco-type donors displayed dissociation kinetics in the order of **26**_**ax**_ > **25**_**ax**_ > **24**_**ax**_, which is consistent with the disarming properties of their protecting groups and C-5 carboxylate or alkoxymethyl group. In contrast, manno-type donors displayed dissociation kinetics in the order **21**_**ax**_ > **23**_**ax**_ > **22**_**ax**_. The C-3 acetyl derivative **21**_**ax**_ exhibited the fastest R_α→OTf, EXSY_, which may be driven by anchimeric assistance and was also observed in C-3 acyl-protected mannosides.^18^ In addition, the results show a limited influence and contribution of the C-5 carboxylate ester to triflate dissociation in both gluco- and manno-type glycosides. Hence, it is unlikely that the C-5 NGP substantially induces triflate dissociation in the solution phase. Rather, the inductive effect of the carboxylate ester slows triflate dissociation by destabilizing a glycosyl cation intermediate. Introduction of a C-3 acyl group in the glucosyl series led to a further reduction of triflate dissociation, which was in sharp contrast to its mannosyl analogue.

**Figure 5:**
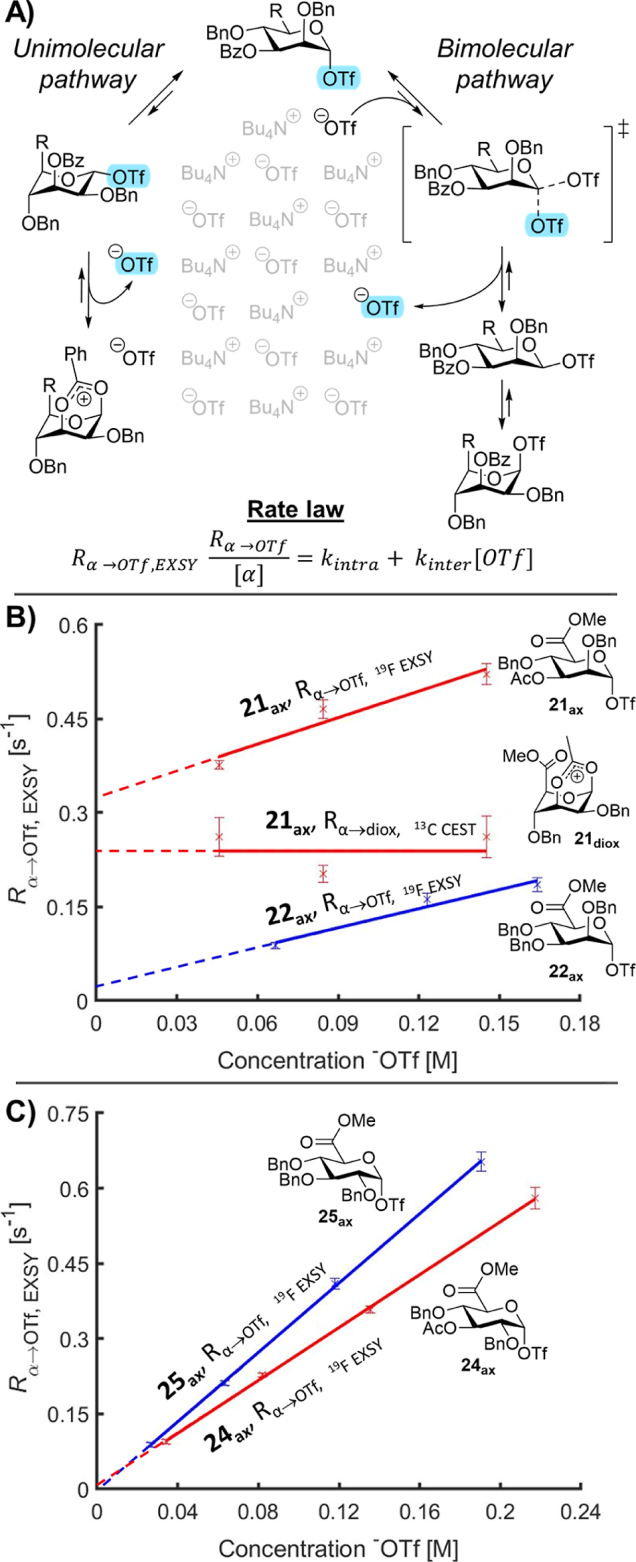
Mechanistic insights into triflate dissociation using exchange NMR. (A) Possible mechanisms of triflate dissociation; (B) triflate dissociation kinetics of mannosyl triflates **21**_**ax**_ and **22**_**ax**_, experiments conducted at −80 °C and −60 °C, respectively; and (C) triflate dissociation kinetics of glucosyl triflates **24**_**ax**_ and **25**_**ax**_, experiments conducted at −40 °C and −50 °C, respectively.

Next, we probed the mechanism of the triflate dissociation. Two mechanisms of triflate dissociation can be distinguished (Figure 5A), a unimolecular and a bimolecular reaction pathway. In the former pathway, triflate dissociation is the result of intramolecular stabilization or direct dissociation independent of the free triflate (^−^OTf) concentration. In the second pathway, displacement of the α-glycosyl triflate by free ^–^OTf in solution affords a reactive β-glycosyl triflate and releases a selectively excited triflate back in solution. This bimolecular process can be seen as a first-order [^−^OTf]-dependent process. Hence, studying the triflate dissociation rate at varying concentrations, ^–^OTf provides information about the mechanism of exchange.^14,18,21^

The experiment was conducted by setting the probe temperature to achieve a triflate dissociation rate of approximately 0.1 s^–1^ and where the α-triflates are thermally stable. In most cases, the exchange rate is expected to increase with increasing ^–^OTf concentrations; hence, starting at a dissociation rate of 0.1 s^–1^ allows a sufficient window to increase the measured rate while still being below the maximum rate measurable with EXSY (≈100 s^–1^). All uronic acid donors showed an increase in R_α→OTf, EXSY_ at increasing [^−^OTf] and were thus triflate-dependent (Figure 5B,C). R_α→OTf, EXSY_ should theoretically be zero in the absence of -OTf if the reaction solely proceeds via a bimolecular reaction mechanism, and C-3 or C-5 NGP is not operative. Indeed, for three out of four donors, extrapolation of R_α→OTf, EXSY_ intercepted at zero, at which both the [^–^OTf] and R_α→OTf, EXSY_ are nearing zero; thus, in this case, no C-3 or C-5 NGP is observed. The exception is the C-3 acyl derivative **21**_**ax**_. In the latter, R_α→OTf, EXSY_ was higher to begin with. Moreover, extrapolation of the curve resulted in a *y*-axis intercept ([^−^OTf] = 0) at R_α→OTf, EXSY_ ≈ 0.3 s^–1^. This glycosylation reaction thus presented significant unimolecular character, which may be explained by the formation of a 1,3-bridged dioxanium ion **21**_**diox**_.

### Detection of Low-Abundant Intermediates in the Solution Phase

**Figure 6:**
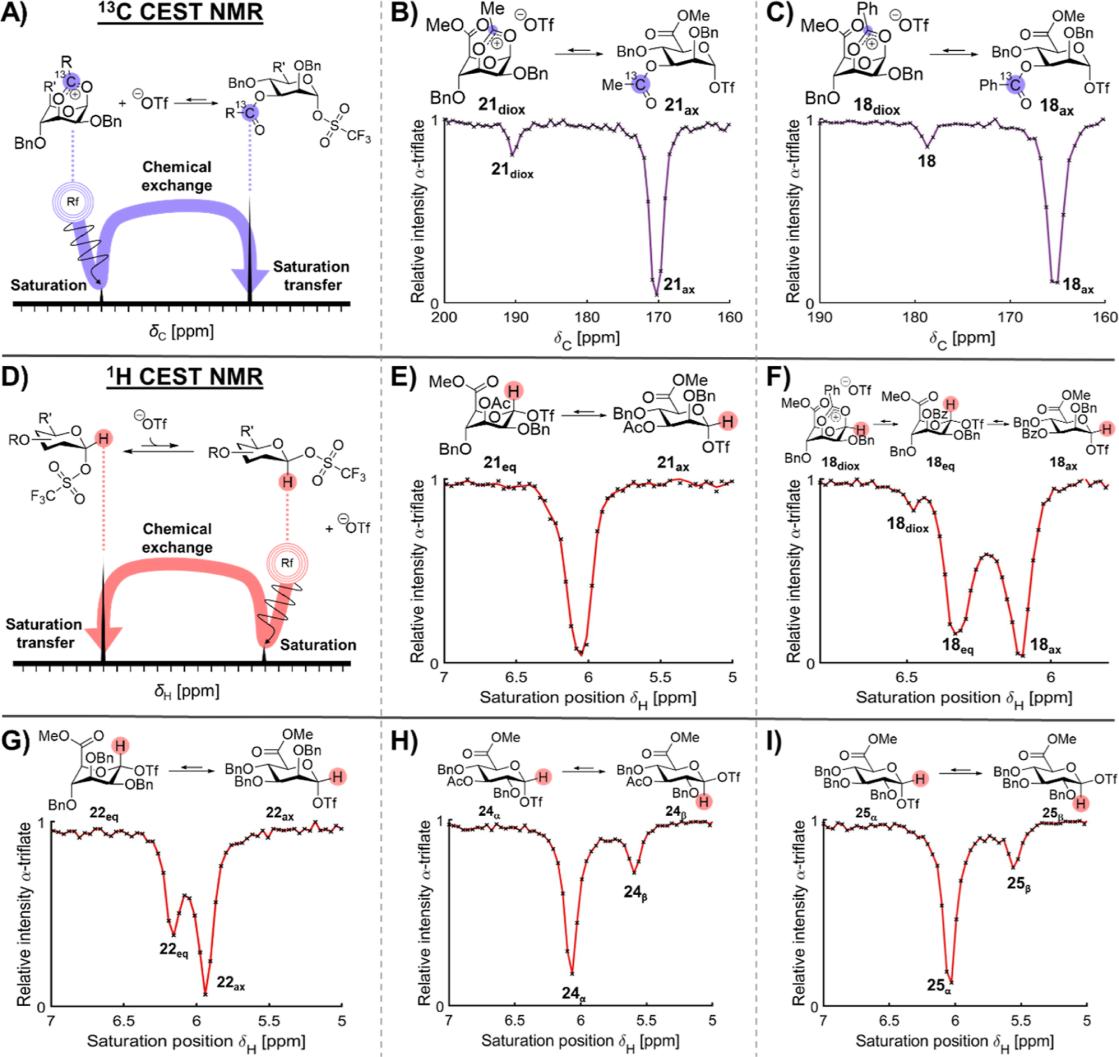
CEST profiles of activated acylated uronic acid donors **1** and **3–5** and perbenzylated donors **2** and **6**. (A) Graphical visualization of ^13^C CEST NMR; (B) ^13^C CEST profile of **21**; (C) ^13^C CEST profile of **18**; (D) graphical visualization of ^1^H CEST NMR; (E) ^1^H CEST profile of **21**; (F) ^1^H CEST profile of **18**; (G) ^1^H CEST profile of **22**; (H) ^1^H CEST profile of **24**; and (I) ^1^H CEST profile of **25**.

To detect and characterize the structure of the highly reactive dioxanium ion or equatorial glycosyl triflate intermediates that may form upon the activation of uronic acid donors **1–6**, we first employed ^13^C CEST NMR (Figure 6A). CEST is highly dependent on the choice of nucleus because the exchange process with one nucleus might be within the boundaries of CEST, while it is not with another nucleus. For the detection of 1,3-dioxanium ions, the chemical shift difference between the carbonyl carbon of an acyl group and the corresponding carbon of the dioxanium ion is generally sufficient (Δϖ > *k*_1_ + *k*_–1_). Therefore, ^13^C-enriched donors **3** and **4** were prepared to increase the sensitivity of the ^13^C CEST experiments. The ^13^C CEST profile of C-3 acetylated mannuronic acid α-triflate displayed a resonance that was in chemical exchange with the ^13^C carbonyl carbon of the α-glycosyl triflate (Figure 6B). Moreover, it correlated to the expected ^13^C chemical shift for a dioxanium ion resulting from C-3 acetyl NGP (δ_C_ = 190 ppm) (Figure 6A,B).^17^ However, no additional structural information on the dioxanium ion could be obtained with traditional 1D and 2D NMR. Therefore, the ^13^C-enriched benzoyl analogue **4** was used to increase the dioxanium ion population by stabilization of the cation.^17^ Again, the ^13^C CEST profile displayed a signal that correlated with the formation of a dioxanium ion resulting from C-3 benzoyl participation (δ_C_ = 178 ppm) (Figure 6C), yet no significant increase in dioxanium ion population was detected. As dioxanium ion formation occurs with the concomitant release of the anomeric triflate, we measured the dioxanium ion formation rate using ^13^C CEST (R_α→diox,13C CEST_) as a function of triflate concentration.^39^ As expected, R_α→diox,13C CEST_ corresponded to the y-intercept value and was independent of [^−^OTf] (0th order) since glycosyl dioxanium ion formation is a unimolecular reaction pathway (Figure 5B).

Next, ^1^H CEST experiments, which focused on the H-1 resonance, were conducted. No dioxanium ion or conformer interconversion could be detected after activation of C-3 acetylated mannuronic acid donor **1**, possibly because the anomeric signals overlap and fall outside of the CEST viewing window (Figure 6E). However, both conformers and an extra exchanging species at δ_H_ = 6.47 ppm were visible in the case of benzoylated derivative **4** (Figure 6F). As both H-1 and the ^13^C resonance of the participating group displayed chemical shifts consistent with previously reported dioxanium ions, it is likely that the dioxanium ion was observed.^17,18^ While having confirmed C-3 acyl NGP, the ^19^F EXSY experiments (Figure 5) demonstrated a minor influence of [^−^OTf] on the exchange rate of triflate dissociation. This suggests the formation of a β-mannosyl triflate. Hence, ^1^H CEST was utilized to scan frequencies characteristic for the anomeric proton of the β-glycosyl triflate (5–6 ppm).^18,21^ No indication of β-mannosyl triflates was found. However, it must be noted that when intermediates cannot be detected using CEST NMR, this does not mean that they do not exist. It is possible that the chemical exchange is either too fast (Δϖ ≪ R_ex_) or too slow to allow sufficient saturation to pass to the reporter peak before the saturation relaxes back due to T_1_-relaxation.^40^ Alternatively, the population could be too low to allow sufficient saturation to pass to the reporter peak before T_1_-relaxation occurs.

Finally, we investigated the glycosylation reaction mechanism of perbenzylated mannuronic acid donor **2** and glucuronic acid donors **5** and **6**. These intermediates displayed a clear bimolecular triflate dissociation mechanism (vide supra), which suggested the formation of a β-glycosyl triflate.^41^ In order to prove the existence of such an intermediate, ^1^H CEST NMR was employed once more (Figure 6D–I). In the case of mannuronate triflate **22**, the ^1^H CEST profile displayed two peaks that were in chemical exchange, namely, the two α-triflate conformers, yet no other exchanging intermediates were found. Surprisingly, detection of β-glucuronosyl triflates **24**_**β**_ and **25**_**β**_ could only be observed at slightly elevated ^–^OTf concentrations (70 and 140 mM). The difficulty in their detection likely results from its low population in combination with relatively slow exchange rates. This results in an insufficient amount of saturation passing from the β-glycosyl triflate to the selected reporter peak (the α-glycosyl triflate anomeric resonance). Increasing [^−^OTf] therefore conveniently enhanced the rates of chemical exchange, thus allowing sufficient saturation transfer from the β-glycosyl triflate to visibly affect the α-glycosyl triflate signal (Figure 6H,I).

Although the exchange NMR studies enabled the indirect detection of the dioxanium ion in solution, it was not possible to observe this species in 1D and 2D NMR to characterize it. We have been able to characterize mannosyl and rhamnosyl dioxanium ions before by boosting their population using a better participation group (e.g., benzoyl or *para*-methoxybenzoyl) and by omitting the base (TTBP) during activation.^14,17,18^ However, in the case of uronic acid **3** and **4**, substitution of the C-3 acetyl did not yield the expected dioxanium ion population boost. Additionally, the conditions could not be tuned (omitting TTBP) to increase the population of the dioxanium ion to a detectable extent before significant degradation of the glycosyl triflate occurred. Hence, we turned to gas-phase conditions to enable the experimental characterization of uronic acid dioxanium ions. To this end, we employed electrospray ionization mass spectrometry (ESI-MS) to generate glycosyl cations derived from glycosyl donors **1**, **2**, **5**, and **6**, which were mass-isolated in an ion trap. Here, the molecular ions were irradiated by a tunable free-electron laser (FELIX) operating in the frequency range from 750 to 1850 cm^–1^. The high laser power of FELIX ensures multiphoton absorption whenever the laser frequency is resonant with a vibrational band of the investigated ion, leading to ion fragmentation, which is monitored with the mass spectrometer. By plotting the fractional ion dissociation as a function of the laser frequency, an IR spectrum can be reconstructed.

The FELIX frequency range is well-suited for distinguishing between potential cation isomers, including the glycosyl oxocarbenium ion, the C-1,C-3-dioxanium ion, and the C-1,C-5-dioxolanium ion. A significant difference in the O=C^+^–O stretching frequency is observed between the C-1,C-3-dioxanium ion (around 1585 cm^–1^) and the C-1,C-5-dioxalanium ion (around 1650 cm^–1^). This difference results from variations in the normal mode vibrations of the endocyclic vs exocyclic OR groups. In contrast, the oxocarbenium ion is identified by a C-1 C=O^+^ carbonylonium stretch around 1600 cm^–1^ and preservation of the C=O carbonyl stretch(es) at approximately 1750 cm^–1^. The structural assignment of the observed spectra (black) is further validated by comparison with DFT-calculated IR spectra (filled) using a previously described workflow,^7,42^ as is detailed in Supporting Information.

**Figure 7:**
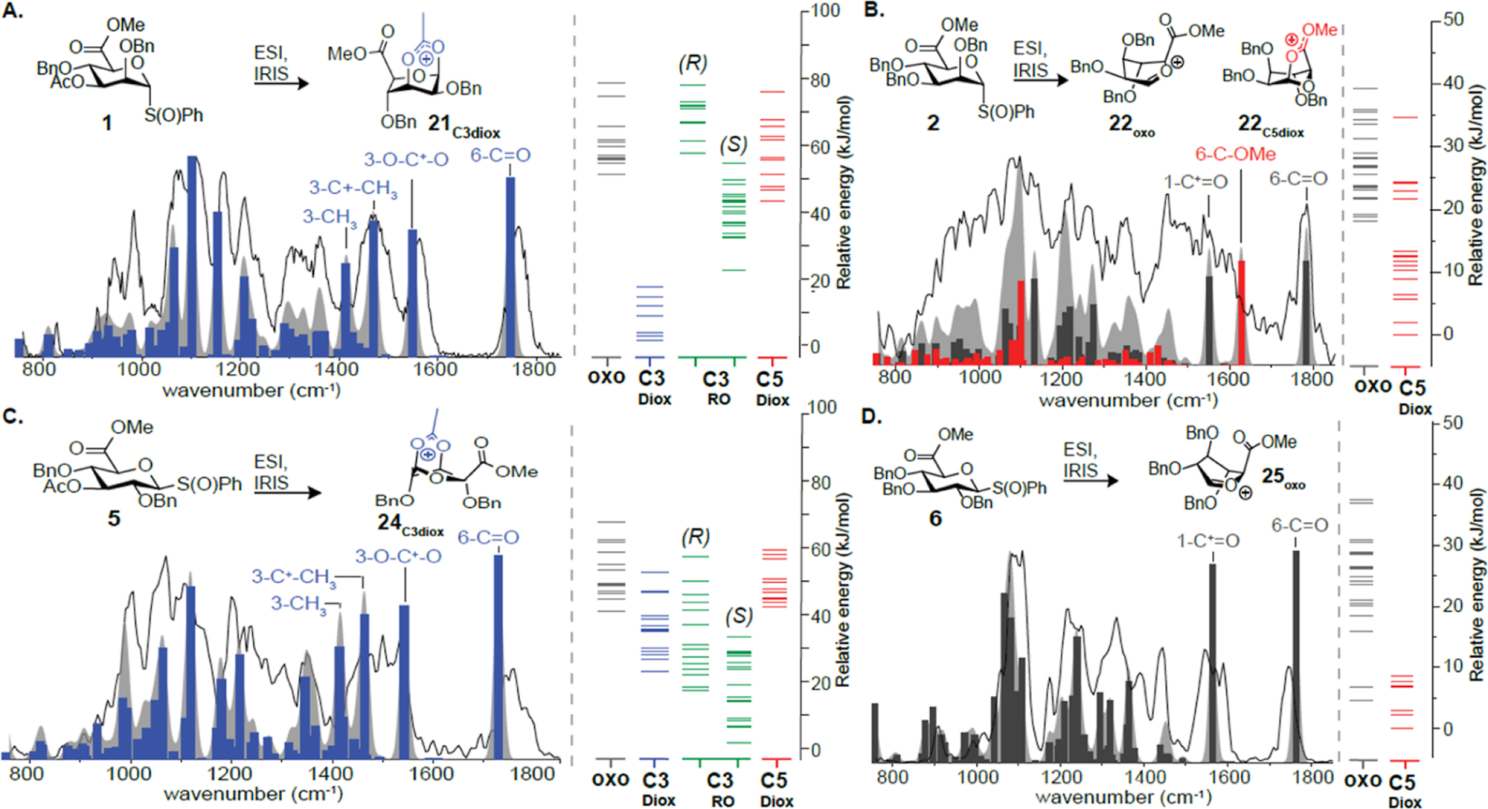
IR ion spectra of glycosyl cations formed from acetylated uronic acid donors **1** and **5** and perbenzylated donors **2** and **6**. Comparison of the measured IR ion spectrum (black line) with the calculated spectra (represented both as stick spectra and as convolved spectral profiles). IR ion spectra are accompanied by the relative energy hierarchies of the reoptimized structures for glucosyl cations of candidate structures.

The C-3 acetyl mannuronic acid donor **1** was ionized and fragmented to afford the glycosyl cation (Figure S28), and its IRIS spectrum was recorded (Figure 7A). The IR spectrum matched best with the DFT-calculated IR spectrum corresponding to C-3 dioxanium ion **21**_**C3diox**_. DFT calculations of the oxocabenium ion and C-5 dioxolanium ion indicated that these species were significantly higher in energy than the C-3 dioxanium ion and were also in poor agreement with the experimental spectrum (Figure S30). In contrast, the glycosyl cation (Figure S31) derived from perbenzylated mannuronic acid donor **2** showed a very broad and congested IR spectrum (Figure 7B). This was unexpected because we investigated the corresponding methylated derivative previously, which showed clear formation of the C-5 dioxolanium ion (Figure S32).^12^ Clearly, the introduction of the benzyl ethers led to the formation of a mix of isomers, which was also validated by DFT calculations (Figure 7B). In contrast, calculations of the methylated derivative reproduce the profound preference for the C-5 dioxolanium ion, indicating the impact of the protecting groups (Figure S33). To ensure that the broad peak pattern was not the result of overlapping fragmentation pathways, we performed high-resolution mass spectrometry, which validated the expected elemental composition (Figure S34). DFT calculations indicated only small differences in energy between the oxocarbenium and dioxolanium ions and mixing the calculated IR spectra (Figure S35) results in a reasonable overlap with the observed spectrum (Figure 7B). Hence, the mix of isomers is likely responsible for the broad peak pattern, and likely these intermediates are not relevant under solution-phase conditions. This is consistent with the NMR experiments, which showed a reduced triflate release rate for the perbenzyl compound **22**_**ax**_ (Figure 4).

In the case of the C-3 acetyl glucuronic acid donor **5**, in-source fragmentation provided the glycosyl cations (Figure S36), which was validated with high-resolution MS (Figure S36). The calculated IR spectrum of the C-3 dioxanium ion was in best agreement with the observed spectrum (Figure 7C, Figure S38). Moreover, its calculated energy was lower than the corresponding oxocarbenium ion, although the difference in energy was much smaller than in the mannuronic acid case. The broad peak pattern may also be the result of ring-opened structures and hence is likely due to a mix of isomers. Since the NMR experiments did not directly or indirectly indicate that the C-3 acetyl in glucuronic acid donors engages in NGP, dioxanium ion formation in the gas phase is likely the result of the extreme solvent- and counterion-free conditions and hence is not relevant under solution-phase conditions. Finally, we investigated perbenzylated glucuronic acid donor **6**. Its glycosyl cation could be generated via in-source fragmentation (Figure S39) and was validated at high resolution (Figure S40). The IR spectrum showed a clear carbonyl stretch at ∼1800 cm^–1^, which indicates that the C-5 NGP did not occur (Figure 7D). Indeed, the calculated IR spectrum of the 1,5-dioxolanium ion is in poor agreement with the experimental IR spectrum (Figure S41).

### Mechanistic Summary

**Figure 8:**
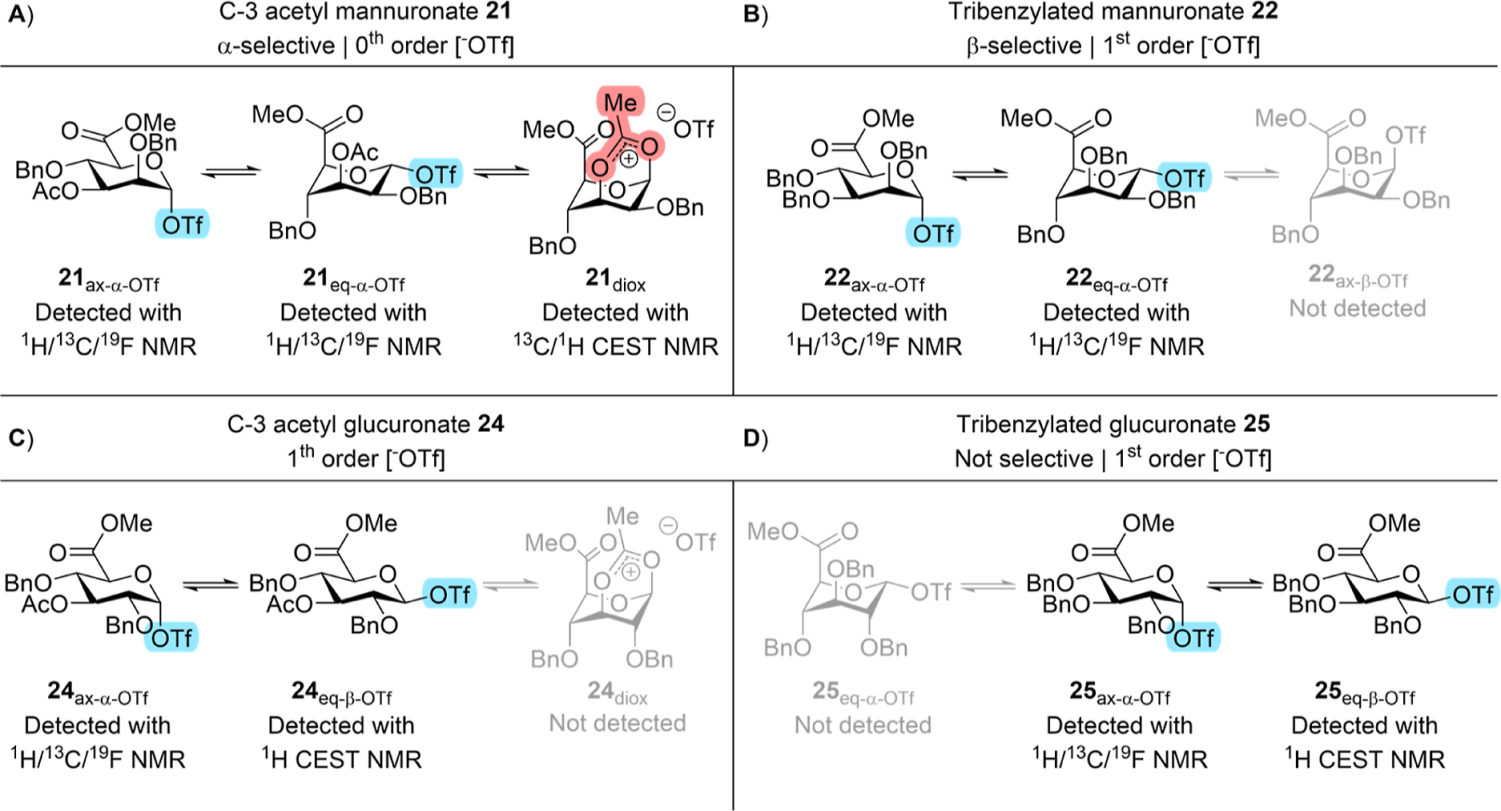
Schematic summary of uronic acid intermediates studied in this work.

By means of the direct and indirect detection and characterization of reaction intermediates derived from uronic acid glycosyl donors **1**, **2**, **5**, and **6**, a rationale for the stereoselectivity of their glycosylation reactions could now be established. We showed that mannuronic acid donors equipped with a C-3 acyl group engage in C-3 NGP in the gas phase. Moreover, solution-phase exchange NMR experiments demonstrated mixed unimolecular and bimolecular triflate release mechanisms. The former mechanism can directly be linked to dioxanium ion formation using ^13^C CEST NMR, which underlines C-3 acyl NGP. Hence, the highly α-selective glycosylation behavior first observed by Van den Bos et al.^24^ can be explained by a reaction pathway proceeding via a low-abundance 1,3-bridged dioxanium ion according to a Curtin–Hammett scenario (Figure 8A).

In the case of tri-*O*-benzylated mannuronic acid, gas-phase experiments revealed the formation of a mixture of an oxocarbenium ion and C-5 dioxolanium ion species. Exchange NMR experiments demonstrated the conformational interconversion of the α-glycosyl triflate and triflate release via bimolecular triflate exchange. However, we were unable to detect the presence of an β-glycosyl triflate. Hence, the β-selectivity associated with this donor type is likely the result of an S_N_2-like displacement of the α-glycosyl triflates as reported before (Figure 8B).^24,25,28^ In contrast, glucuronic acid donors are known to glycosylate with a much lower stereoselectivity.^43−45^ Since we observed both their α- and β-glycosyl triflate derivatives, the mixed outcome of the reaction can be explained by S_N_2-like substitution reactions on both rapidly equilibrating glycosyl triflate species according to a Curtin–Hammett (or Curtin–Hammett-like) scenario. In these situations, the product ratio is determined by the relative energy barriers in the product-forming step (Figure 8C and D).

## Conclusions

This study provides evidence on the mechanism of the C-3 acyl NGP in manno-type sugars using FELIX-based IR spectroscopy and NMR spectroscopy. We demonstrated that a conformational ring-flip is required prior to participation in mannuronic acids, mannose, and rhamnose. This orients the participating group in an axial position, which enables C-3 NGP. In addition, we showed that the introduction of the C-3 acyl group on mannuronic acids accelerates triflate dissociation as a result of NGP. The corresponding 1,3-bridged dioxanium ion was detected using ^13^C CEST NMR and gas-phase infrared multiple-photon dissociation (IRMPD) spectroscopy. Hence, this low-abundance species was assigned as the product-forming intermediate that afforded the α-glycoside product via a Curtin–Hammett scenario. Using the combination of IRIS and NMR experiments to elucidate the structure of reactive intermediates contributes to our understanding of glycosylation reactions and can drive the development of more effective glycosylation methodology.

## Supporting Information Available

Synthetic procedures for the synthesis of compounds **1–17** and their corresponding analytical data (NMR and MS), theoretical and practical description of the EXSY and CEST experiments, VT-NMR procedures, additional variable-temperature NMR spectra and raw ^19^F EXSY NMR data, ion spectroscopy, mass spectrometry data, and computational IR spectra (PDF)
