# Supplementary material for: Mechanism of C-3 Acyl Neighboring Group Participation in Mannuronic Acid Glycosyl Donors
Source: J Am Chem Soc. 2024 Dec 18;147(1):932–44. doi: 10.1021/jacs.4c13910 (PMC11726434; doi:10.1021/jacs.4c13910)
Supplement: Supplementary file 1 — ja4c13910_si_001.pdf [file ja4c13910_si_001.pdf]

The Mechanism of C-3 acyl Neighboring Group Participation in Mannuronic Acid Glycosyl Donors

Supporting information

Authors: Frank F. J. de Kleijne#[1], Peter H. Moons#[1], Floor ter Braak#[1], Hero R. Almizori[1], Luuk J. H. Jakobs[1], Kas J. Houthuijs[2], Giel Berden[2], Jonathan Martens[2], Jos Oomens[2], Floris P. J. T. Rutjes[1], Paul B. White*[1], Thomas J. Boltje*[1]

[1]Radboud University, Institute for Molecules and Materials (IMM), Synthetic Organic Chemistry, 6525 AJ, Nijmegen, The Netherlands

[2]Radboud University, Institute for Molecules and Materials, FELIX Laboratory, 6525 ED, Nijmegen, The Netherlands

F.F.J.d.K, P.H.M and F.t.B. contributed equally
*Corresponding authors: Email: Paul.White@ru.nl, Thomas.Boltje@ru.nl

Table of Contents

Theoretical background S2

  Initial rate approximation S2

  Chemical Exchange Saturation Transfer NMR (CEST NMR) kinetic experiments S4

  Standard deviations S6

NMR experimental details S7

  Instrumentation S7

  Chemical Exchange Saturation Transfer NMR (CEST NMR) spectroscopy S7

  Selective ^{19}F Exchange NMR (EXSY NMR) spectroscopy S7

  Sample preparation variable temperature NMR (VT NMR) S8

  EXSY Acquisition S8

  CEST Profile Acquisition S9

  CEST Kinetics Acquisition S9

General synthetic methods S10

Ion spectroscopy in a modified ion trap mass spectrometer S11

Generation of computational IR spectra S11

Chemical synthesis S12

Supporting characterization data S39

Supporting donor activation data S42

Supporting NMR figures S52

Rhamnose: assigning the minor resonance in 19F NMR as the equatorial α-triflate S56

Supporting IRIS figures S58

Supporting exchange rates S67

References S69

Data

  EXSY kinetic data at variable temperatures S70

  EXSY kinetic data at variable tetrabutylammonium triflate concentrations S76

  ^{19}F EXSY profiles raw data S93

  Synthetic spectra S98

  Coordinates S141

Theoretical background

Initial rate approximation: The *α*-triflates dissociate to form triflate anion in two possible mechanisms (Eq. S1 and S2). The rate law of the corresponding reactions are described in equation S3 and S4 for the intramolecular glycosyl stabilization and intermolecular glycosyl stabilization respectively. Both processes can proceed simultaneously, hence the overall rate law could be a combination of both (Eq. S5).

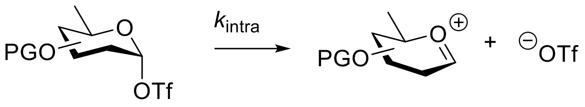

Eq. S1

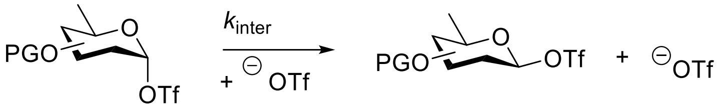

Eq. S2

$$\frac{d[OTf]}{dt} = -\frac{d[\alpha]}{dt} = R_{a\to OTf} = k_{intra}[\alpha] \quad \text{Eq. S3}$$

$$\frac{d[OTf]}{dt} = -\frac{d[\alpha]}{dt} = R_{a\to OTf} = k_{inter}[\alpha][^{-}OTf] \quad \text{Eq. S4}$$

$$\frac{d[OTf]}{dt} = -\frac{d[\alpha]}{dt} = R_{a\to OTf} = k_{intra}[\alpha] + k_{inter}[\alpha][^{-}OTf] \quad \text{Eq. S5}$$

Selective 1D ^{19}F EXSY NMR is a suitable method to study the exchange. By applying a selective excitation pulse on both the α-triflate resonances simultaneously, formation of triflate anion can be measured despite the high population triflate already present in the reaction mixture. This is possible for two main reasons: 1) the resonances of both the *α*-triflate and triflate anion are sufficiently separated to selectively excite the α-triflate in ^{19}F NMR, and, 2) only excited-state nuclei are detected in EXSY NMR. Varying the delay (mix time, τ_m) between the excitation of the *α*-triflate resonance and the spectrum acquisition allows to measure different degrees of conversion for the *α*-triflate into the triflate anion resonance. Eventually, by applying an 'infinitely long' mix time, the equilibrium ratio of *α*-triflate and triflate anion is obtained Figure S1).

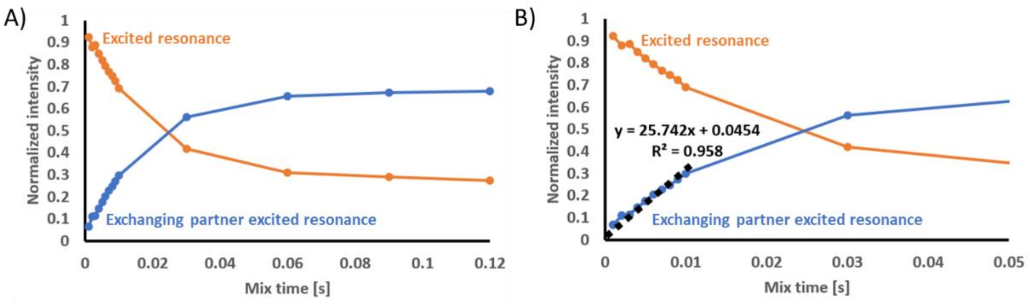

Figure S1: A) Correlation of EXSY mix time to intensity selected/excited resonance and its exchanging partner that is forming. B) Displaying the linear initial rate approximation.

The initial triflate formation is linear and kinetics could be described according to the initial rate approximation. Over the initial linear interval, the reaction rate is the α-triflate consumption and triflate anion formation (Eq. S6 and S7). Herein, $[\boldsymbol{\alpha}]_t$ = concentration excited state α-triflate at set mix time after applying the excitation pulse on the α-triflate resonance; $[\boldsymbol{\alpha}]_0$ = concentration excited state α-triflate directly after applying a selective excitation pulse on the α-triflate resonance; $[^{-}\mathbf{OTf}]_t$ = concentration excited state $^{-}$OTf at set mix time after applying the excitation pulse on the α-triflate resonance; $[^{-}\mathbf{OTf}]_0$ = concentration excited state $^{-}$OTf directly after applying a selective excitation pulse on the α-triflate resonance (hence, $[^{-}\mathbf{OTf}]_0 = 0$). Substituting equation S5 into equation S6 gives the concentration excited state α-triflate in terms of concentration and mix time (Eq. S8).

$$\frac{[\boldsymbol{\alpha}]_t - [\boldsymbol{\alpha}]_0}{\tau_m} = -R_{\alpha\rightarrow OTf} \qquad \text{Eq. S6}$$

$$\frac{[^{-}\boldsymbol{OTf}]_t - [^{-}\boldsymbol{OTf}]_0}{\tau_m} = R_{\alpha\rightarrow OTf} \qquad \text{Eq. S7}$$

$$[\boldsymbol{\alpha}]_t = [\boldsymbol{\alpha}]_0 - (k_{intra}[\boldsymbol{\alpha}] + k_{inter}[\boldsymbol{\alpha}][^{-}OTf]) \times \tau_m \qquad \text{Eq. S8}$$

At the very start of the reaction, the concentration *α*-triflate deviates only marginally compared to the starting concentration ($[\boldsymbol{\alpha}]_0$). In accordance to the initial rate approximation, equation S8 becomes equation S9. Additionally, within the initial rate approximation, only an α-triflate conversion of about 5-15% is recorded. Therefore, the concentration excited state triflate ($[^{-}\mathbf{OTf}]$) is sufficiently small (especially compared to the bulk concentration non-excited triflate anion ($[^{-}$OTf$]$)) such that the backwards reaction can be neglected. Subsequently, dividing the equation by $[\boldsymbol{\alpha}]_0$ simplifies the equation to S10.

$$[\boldsymbol{\alpha}]_t = [\boldsymbol{\alpha}]_0 - (k_{intra}[\boldsymbol{\alpha}]_0 + k_{inter}[\boldsymbol{\alpha}]_0[^{-}OTf]) \times \tau_m \qquad \text{Eq. S9}$$

$$\frac{[\boldsymbol{\alpha}]_t}{[\boldsymbol{\alpha}]_0} = 1 - (k_{intra} + k_{inter}[^{-}OTf]) \times \tau_m \qquad \text{Eq. S10}$$

Within NMR spectroscopy, the concentration is proportional (with a constant, c) related to the absolute integral ($\int I_x$) of the observed resonances (Eq. S11 and S12). Substituting equation S10 with S11 and S12 gives the absolute integral of the excited state *α*-triflate resonance as function of mix time (Eq. S13).

$$\int I_{\alpha,t} = c \times [\boldsymbol{\alpha}]_t \qquad \text{Eq. S11}$$

$$\int I_{\alpha,0} = c \times [\boldsymbol{\alpha}]_0 \qquad \text{Eq. S12}$$

$$\frac{\int I_{\alpha,t}}{\int I_{\alpha,0}} = 1 - (k_{intra} + k_{inter}[^{-}OTf]) \times \tau_m \qquad \text{Eq. S13}$$

One complication is that T_1 relaxation occurs during the mixing time, which will reduce the absolute integral or intensity of the selected and exchanged resonances over time and plotting S13 will result in a multiexponential decay process if $k \leq T_1^{-1}$. This can be easily taken into account if the T_1 is known for each in the absence of exchange. However,

if the T_1s for the species are very similar and thus experience similar rates of relaxation, then an approximation can be made where the integral of the α-triflate at t=0 ($I_{\alpha,0}$) is the sum of the integrals of the α-triflate and triflate anion at a given mix time (Eq. S14). This then allows Equation S13 to be rewritten in an internally-consistent manner where the decay is normalized by the measurable peaks for each given mix time. The slope of the plot (Eq. S16) is directly related to the rate constants of both exchange processes (Eq. S1 and S2).

$$\int I_{\alpha,0} = \int I_{\alpha,t} + \int I_{OTf,t} \qquad \text{Eq. S14}$$

$$\frac{\int I_{\alpha,t}}{\int I_{\alpha,t} + \int I_{OTf,t}} = 1 - (k_{intra} + k_{inter}[^{-}OTf]) \times \tau_m \qquad \text{Eq. S15}$$

$$Slope = k_{intra} + k_{inter}[^{-}OTf] \qquad \text{Eq. S16}$$

The rates measured and determined by selective ^{19}F EXSY spectroscopy is, as described above, directly the slope of the normalised absolute integral of the α-triflate ($\int I_{\alpha,t}$) versus mixing time. Hence, equation S16 is in the main text referred to as $R_{\alpha \rightarrow OTf, EXSY}$ (Eq. S17).

$$R_{a \rightarrow OTf,EXSY} = Slope = k_{intra} + k_{inter}[^{-}OTf] \qquad \text{Eq. S17}$$

Above is described how the rate of α-triflate dissociation is determined by applying a broad selection pulse that excites both the axial and the equatorial α-triflates simultaneously. A narrow selection pulse that only excites *one* selected resonance can also be applied. By varying the mixing time interconversion rates for the axial to equatorial α-triflates (and *vice versa*) can be determined. The approach as described above still hold. First determine the interconversion rate laws, then by going through the manipulations of equations S6 up to and including S16 the equivalent of equation S17 for interconversion is defined: $R_{ax \rightarrow eq, EXSY}$.

Chemical Exchange Saturation Transfer NMR (CEST NMR) kinetic experiments: Saturation transfer experiments to derive the exchange constant from the *α*-triflate to dioxanium ion were conducted as reported by Serianni et. al.[1] The system is considered as in Scheme S1, and their corresponding formation rates are described in eq. S18 and S19.

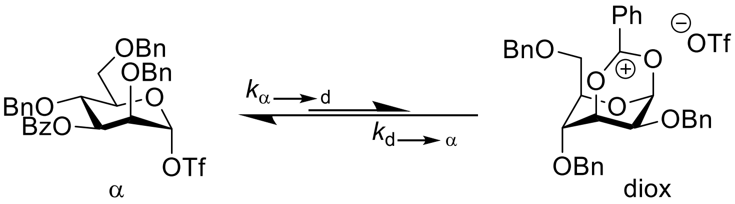

Scheme S1: Equilibrium considered for determining the reaction rate constant of dioxanium ion formation ($k_{\alpha \rightarrow d}$).

$$\frac{d[\alpha]}{dt} = R_{diox \rightarrow a} = k_{d \rightarrow \alpha}[diox][OTf] \qquad \text{Eq. S18}$$

$$\frac{d[diox]}{dt} = R_{a \to diox} = k_{\alpha \to d}[\alpha] \qquad \text{Eq. S19}$$

Equation S20 describes the modified Bloch equation that accounts for change in longitudinal magnetization (M_z) due to T_1 relaxation and chemical exchange as a function of time (t).

$$\frac{dM_z^{trif}(t)}{dt} = \frac{-\left(M_z^{trif}(t) - M_z^{trif}(0)\right)}{T_{1,trif}} - \frac{M_z^{trif}(t)}{\tau_{trif}} + \frac{M_z^{diox}(t)}{\tau_{diox}} \qquad \text{Eq. S20}$$

Herein: $T_{1,trif}$ is the spin-lattice relaxation time of the *α*-triflate resonance, τ_{trif} is the lifetime of the triflate, and τ_{diox} is the lifetime of the dioxanium ion. Due to saturation of the dioxanium resonance, the magnetization of the dioxanium (M_z^{diox}) becomes zero, and, as a result, M_z^{diox}/τ_{diox} also goes to zero. The resulting equation can then be integrated to yield equation S21.

$$M_z^{trif}(\tau) = M_z^{trif}(0) \times \left(\frac{\tau_{1,trif}}{\tau_{trif}} e^{\frac{-\tau}{\tau_{1,trif}}} + \frac{\tau_{1,trif}}{T_{1,trif}}\right) \qquad \text{Eq. S21}$$

Here, $M_z^{trif}(\tau)$ and $M_z^{trif}(0)$ are the intensities of the α-triflate resonance after applying a saturation pulse for the duration of τ at the dioxanium ion resonance ($M_z^{trif}(\tau)$), and the intensity of the α-triflate resonance after applying a saturation pulse for the duration of 0 seconds at the dioxanium ion resonance ($M_z^{trif}(0)$). Subtracting the peak intensity of the α-triflate with 'infinitely long' saturation at the dioxanium ion ($M_z^{trif}(\infty)$) from $M_z^{trif}(\tau)$ gives the net saturation effect on the peak decay. Typically, $M_z^{trif}(\infty)$ is determined by saturating the dioxanium ion for five times the relaxation time of the carbonyl. Plotting the natural logarithm of the net peak decay resulting from saturation ($\ln(M_z^{trif}(\tau) - M_z^{trif}(\infty))$) against the saturation time (τ) gives a linear correlation where the slope is related to $\tau_{1,trif}$ according to equation S22.

$$slope = \frac{-1}{\tau_{1,trif}} \qquad \text{Eq. S22}$$

The observed lifetime ($\frac{1}{\tau_{1,trif}}$, Equation S23) describes the relation between the α-triflate lifetime (τ_{trif}) and its T1. $T_{1,trif}$, can be determined from the acquired data according to equation S24.

$$\frac{1}{\tau_{1,trif}} = \frac{1}{\tau_{trif}} + \frac{1}{T_{1,trif}} \qquad \text{Eq. S23}$$

$$T_{1,trif} = \frac{M_z^{trif}(0)}{M_z^{trif}(\infty)} \times \frac{1}{\tau_{1,trif}} \qquad \text{Eq. S24}$$

Applying equations S22, S23, and S24 yields the lifetime of the α-triflate. As described by McConnell,[2] the lifetime is to be related to pseudo first-order rate constants. As a consequence, in the system as described by Scheme S1, the lifetimes of the α-triflate and

dioxanium ion are given by equations S25 and S26. Hence, the rate constant for the formation of dioxanium ion from the α-triflate ($k_{\alpha\to d}$) is directly obtained from τ_{trif} (Eq. 25).

$$\frac{1}{\tau_{trif}} = k_{\alpha\to d} \qquad \text{Eq. S25}$$

$$\frac{1}{\tau_{diox}} = k_{d\to\alpha}[^{-}OTf] \qquad \text{Eq. S26}$$

$R_{\alpha\to d,\,CEST}$ is used to describe equation S26. Hence, for unimolecular processes $R_{\alpha\to d,\,CEST}$ is described according to equation S27.

$$R_{\alpha\to d,CEST} = \frac{1}{\tau_{trif}} = k_{\alpha\to d} \qquad \text{Eq. S27}$$

This can also be conducted in other nuclei to determine for example α- to β-triflate exchange, or ring-flip kinetics.

Standard deviations: The rates determined by EXSY are directly the slope of the normalized α-triflate peak integral against mix time (Eq. S17). The corresponding graph is based on the measured data analysed with the least square regression method. Equations S28 is used to determine the error in the determined EXSY rate based on the measured input data.

$$s_x = \sqrt{\frac{\sum(X_i-\bar{X})^2}{n-1}} \qquad \text{Eq. S28}$$

Rates determined by CEST are based on a linear relationship between the natural logarithm of the net saturation effect versus saturation time. The slope represents ${\tau_{1trif}}^{-1}$ and the standard deviation is calculated using equation S28. The relative error of ${\tau_{1trif}}^{-1}$ is determined according to equation S29 and the resulting relative error is applied to the determined rate (Eq. S30).

$$Relative\ error\ slope = \frac{e_{Slope}}{Slope} \qquad \text{Eq. S29}$$

$$e_{R_{\alpha\to d,CEST}} = \frac{e_{Slope}}{Slope} \times R_{\alpha\to d,CEST} \qquad \text{Eq. S30}$$

NMR experimental details

Instrumentation: Variable temperature NMR (VT NMR) experiments were conducted on a Bruker 300 MHz Avance III HD nanobay equipped with a BBFO probe and on the JEOL 500 ECZ-R spectrometer equipped with a ROYAL-HFX or ROYAL probe. Low temperature VT operations were achieved with the aid of LN_2 evaporator to supply the cold gas, which the probe heated to the desired temperature. The temperature for VT experiments was calibrated against a pure MeOH standard to accurately determine the probe temperature.

Chemical Exchange Saturation Transfer NMR (CEST NMR) spectroscopy: CEST NMR was utilized in ^{1}H, ^{13}C, and ^{19}F NMR spectroscopy. CEST NMR spectra were recorded by incrementing the saturation over a domain of interest. For ^{1}H the domain of interest is typically between 9 and 4 ppm; ^{13}C CEST was typically performed in a window between 190 and 160 ppm or 130 to 90 ppm; and ^{19}F CEST was performed in a range of -72 to -80 ppm. Before each experiment, the 90-degree pulse was calibrated. A saturation field strength was chosen with respect to experimental duration, resolution, and signal intensity (eg. high resolution (small saturation field strengths) leads to weak signals and long experimental times, in contrast, low resolution (large saturation field strength) leads to stronger signals, faster experiments but low resolution). Typically, saturation field strengths were chosen to be 20 Hz (^{1}H CEST), 80 or 40 Hz (^{13}C CEST), and 30 Hz (^{19}F CEST). The saturation was achieved either by CW saturation (Bruker) or by pulsed saturation using laminar pulses (JEOL). Finally, typical saturation times were set to 2-3 seconds; the relaxation delay was set one second longer then the saturation time (3-4 seconds); number of scans were typically 2-4 per frequency; Two dummy scans (Bruker) or 2 (JEOL) were executed.

Selective ^{19}F Exchange NMR (EXSY NMR) spectroscopy: EXSY NMR was utilized in ^{19}F NMR spectroscopy. Before each experiment, the 90-degree pulse was calibrated and then the selective excitation offset was set to the resonance of interest. The selection pulse typically spanned either 0.2 or 0.6 ppm and was on-resonance with the (one of the/both) glycosyl *α*-triflates. The power levels of the excitation pulse were calculated against the actual 90-degree pulse and the selection window. Relaxation delays were typically set to 5xT_1 of the triflates, and the number of scans were set to 8 with 2 dummy scans. The longest mix times were determined empirically so that they fit within the initial rate approximation (~10% conversion). For experiments where the shortest mix time was > 60 ms, a pulse sequence that contains a Z-gradient element to crush zero-quantum magnetization and clean up artifacts was used (Bruker: selnogp, JEOL: noesy_1d). However, frequently the shortest mix time was << 60 ms, therefore the versions of the above experiments without the Z-gradient element were utilized.

Sample preparation variable temperature NMR (VT NMR): Glycosyl sulfoxide donor (1.0 eq., typically 15 mg) and TTBP (2.5 eq.) were dissolved in dried DCM-d_2 (600 μL). Two spherical molecular sieves (4 or 5 Á) were added to the NMR tube and the tube was transferred to an analytical scale where internal standard (trimethyl(4-trifluoromethylphenyl)silane) was added. A stock solution of Tf_2O was prepared in DCM-d_2 such that upon addition of stock solution (50 μL), the desired amount Tf_2O (1.3 eq.) could be added. When the NMR sample and Tf_2O stock solution were ready, the NMR tube was cooled to -80 °C (dry ice/acetone bath) and to the cold tube was added the freshly prepared Tf_2O stock solution (50 μL). The solution generally did not change colour or became (light) yellow or blue/purple upon addition of Tf_2O, was shaken quickly (3x) and was carefully transferred to the NMR. In the probe, the temperature was heated to -60 °C for 1h. The temperature was decreased to -80 °C and characterization/kinetic experiments were performed at variable temperatures.

For experiments starting with glycosyl thioethers, Ph_2SO (1.1 eq.) was weight to the donor solution. No TTBP was used for these experiments.

For experiments at varying concentrations tetra butyl ammonium triflate (TBAT), typically the Bruker 300 MHz NMR was used and the sample was dissolved in 500 μL DCM-d_2. A 1.0 M solution of tetra butyl ammonium triflate (TBAT) was prepared in DCM-d_2. To the solution was added activated molecular sieves (4 Á) and the solution was stored under argon at -80 °C. This solution was removed from the -80 °C fridge 1h before the NMR experiment.

NMR experiments at various concentrations triflate anion were executed as described above with respect to sample preparation. After activation at the desired temperature, the probe was heated to the temperature where the sample displayed an exchange ($R_{\alpha \rightarrow OTf, EXSY}$) of about 0.2 s^{-1} to allow sufficient exchange at the lowest concentration and sufficient opportunity to increase as a consequence of the increased triflate concentration before falling out the window of EXSY NMR (see main text). After recording the triflate dissociation under standard conditions, the sample was removed from the probe, quickly stored in a dry ice/acetone bath (-80 °C) and the TBAT solution was added (20 μL). The sample was quickly shaken to homogenize the solution (3x) and was carefully transferred to the probe. The sample was locked to DCM-d_2, tuned, and shimmed before performing NMR experiments. After finishing the EXSY experiment, the cycle was repeated for two more time (by adding 30 μL and 50 μL TBAT solution). In the data workup, the internal standard was used to accurately correct the concentration to volume and TBAT added.

EXSY Acquisition: ^{19}F EXSY rates were determined according to kinetics from the initial rates approximation. Typically 8-10 EXSY spectra were recorded with varying mixing times between the selection/excitation pulse and the acquisition. Typically a maximum conversion for the selected resonance (*α*-triflate) to the newly formed species (^{-}OTf) was between 10-15%. All acquired spectra were phased and baseline correction was applied. The peak areas from both the *α*-triflate resonance and triflate anion were normalized

against the sum of the integrals for their corresponding spectrum. The normalized intensities were then plotted against the mixing time to yield a linear plot where the absolute slope of each graph represents the rate of *α*-triflate being converted to the $^{-}$OTf resonance.

CEST Profile Acquisition: CEST profiles were constructed using either 1D ^{13}C, ^{1}H or ^{19}F spectra with saturation at variable positions. A spectrum with no saturation or with off-resonance saturation to both the major and minor observable signal is required as reference named $M_z(0)$ (typically a 1D spectrum with saturation at δ_C = 190 ppm; δ_H = 9.0 ppm; or δ_F = -72 ppm). The peak intensity for the *α*-triflate (^{13}C and ^{1}H CEST) and $^{-}$OTf (^{19}F) resonances were determined for all individual spectra after phasing and applying baseline correction. The peak intensity of every spectrum was divided by the peak intensity of the unaltered spectrum ($M_z(0)$) to obtain the relative peak intensity of the main observable species as function of saturation frequency. Plotting the relative intensity of the *α*-triflate as function of the saturation frequency gives the CEST spectrum.

CEST Kinetics Acquisition: Dioxanium ion formation rates ($R_{\alpha \rightarrow d, CEST,}$ Scheme S1, page S4) were determined by using saturation-transfer. The saturation frequency offset was set to be on-resonance with the minor exchangeable species (e.g. the dioxanium C=O 13C resonance) as discovered by first obtaining a CEST profile. The saturation offset frequency was held constant while the saturation duration was varied. Deriving the exchange rate required a spectrum having no saturation ($M_z(0)$), a spectrum where the exchange and relaxation rates are in equilibrium ($M_z(inf)$), and a number of spectra where a decay of the main observable is visible as function of saturation time ($M_z(\tau)$). Typically the saturation times were set to 0 seconds, 0.1 seconds incrementing to 3 seconds, and 5 seconds. The resulting 1D ^{13}C spectra were phased and baseline correction was applied. The maximum peak intensity was determined for all individual 1D ^{13}C NMR spectra and was plotted against the saturation time to yield the kinetic build-up curves. The crude 1D ^{13}C peak intensities were converted as described above (page S4-5) to yield the exchange rate. This method was also applied in ^{19}F CEST.

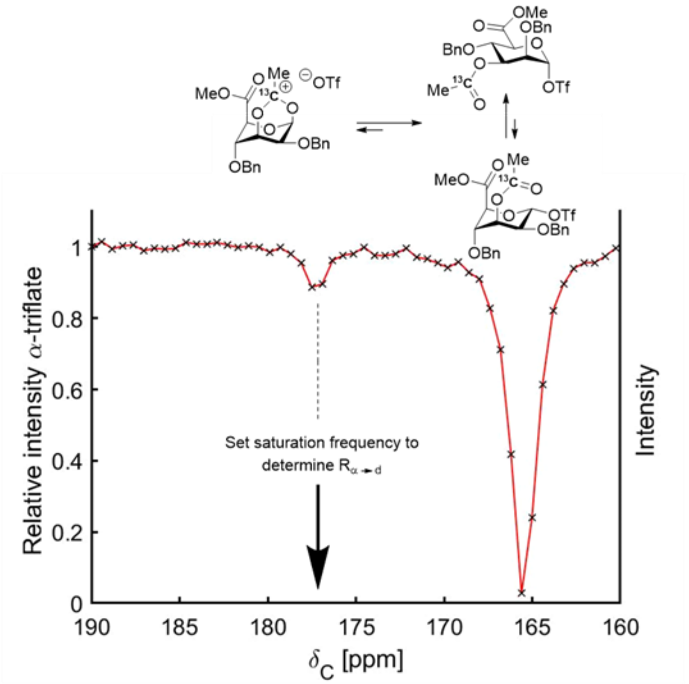

Figure S2: Rate of α-triflate becoming dioxanium ion is determined by setting the saturation frequency to the minimum of the (minor) dioxanium ion signal in 13C CEST NMR and subsequently applying variable saturation times on-resonance.

General synthetic methods: ^{1}H, ^{13}C{^{1}H, COSY, ^{1}H-^{13}C HSQC (coupled and decoupled), and ^{1}H-^{13}C HMBC spectra were acquired on either a Bruker 500 MHz AVANCE III spectrometer, or JEOL 500 ECZ-R spectrometers. The Bruker 500 MHz Avance III spectrometer is equipped with a Prodigy BB cryoprobe. The JEOL 500 ECZ-R spectrometers were equipped with either a SuperCOOL broadband probe, ROYAL broadband probe, or ROYAL HFX broadband probe. Chemical shifts are reported in parts per million (ppm) relative to tetramethylsilane (TMS), or residual solvents as the internal standard. NMR data is presented as follows: chemical shift, multiplicity (s = singlet, d = doublet, t = triplet, dd = doublet of doublets, m = multiplet and/or multiple resonances), coupling constant (J) in hertz (Hz), integration. All NMR signals were assigned on the basis of 1H NMR, 13C NMR, COSY, HSQC, and TOCSY experiments.

Mass spectra were recorded on an JEOL AccuTOF CS JMST100CS mass spectrometer. Automatic flash column chromatography was performed using Biotage Isolera Spektra One, using SNAP cartridges (Biotage, 30− 100 µm, 60 Å), 10−50 g. TLC analysis was conducted on silica gel F254 (Merck KGaA) with detection by UV absorption (254 nm) where applicable; by spraying with 10% sulfuric acid in methanol followed by charring at ≈300 °C or by spraying with $KMnO_4$ stain consisting of (0.06M $KMnO_4$, 0.5M K_2CO_3 and 0.02M NaOH in water) after gently heating of the plate.

All reagents were commercially purchased unless stated differently. All reagents were used without purification apart for dry DCM, THF, and toluene which were freshly distilled. DCM-d_2 was dried by adding activated molecular sieves (4 Å) directly to the bottle as

obtained from Deutero GmbH. Molecular sieves (3 Å, 4 Å, or 5 Å) were flame-activated under a vacuum prior to use. All dry reactions were carried out under an argon atmosphere using flame-dried flasks.

Ion spectroscopy in a modified ion trap mass spectrometer

The experimental apparatus is based on a modified 3D quadrupole ion trap mass spectrometer (Bruker, AmaZon Speed ETD) that has been coupled to the beamline of the FELIX infrared free electron laser (IR-FEL).[3] : For each compound protonated, sodiated and ammonium adduct ions were generated by electrospray ionization from solutions of 10^{-6} M (in 50:50 acetonitrile:water for compound 2 and 50:50 methanol:water for all other measured compounds) containing 2% ammonium acetate and introduced at 2 µl min^{-1}. Source conditions were optimized to maximize the production of the glycosyl cations of interest from their triflate precursors. These fragment ions were subsequently mass isolated in the ion trap and an additional MS/MS stage was used to irradiate the ions with a single macropulse from the mid-infrared FEL (750-1850 cm^{-1}). The FEL was tuned to provide 5 µs optical pulses at 10 Hz repetition rate having a pulse energy of 30–60 mJ over the tuning range (bandwidth ~0.4% of the centre frequency). The pulse energy used for measurements was appropriately attenuated in order to avoid excessive depletion of ions (saturation). Upon resonance between the laser frequency and an absorption band of the ions, multiple IR photons are absorbed during a single FEL macropulse, and unimolecular dissociation occurs. Parent and fragment ion intensities are recorded in the mass spectrometer as function of the IR frequency (3 cm^{-1} step size). Relating the parent ion intensity to the total fragmentation intensity in the observed mass spectra (yield=ln(ΣI(fragment+parent ions)/I(parent ions)) then generates an infrared vibrational spectrum.[4] The yield is obtained from several averaged mass spectra and is linearly corrected for laser pulse energy; the IR frequency is calibrated using a grating spectrometer.

Generation of computational IR spectra

Vibrational spectra of the candidate geometries were predicted using a previously reported computational workflow.[5-6] A SMILES code for the oxocarbenium, C-1,C-3 dioxanium, C-1,C-5 dioxolanium and ring-opened ions served as input for the cheminformatics toolbox RDkit. For each ion, 500 random conformations were generated using the distance geometry algorithm, which were minimized using the MMFF94 classical forcefield. The 40 most distinct geometries were selected based on the root-mean-squared distance between them. The geometries served as input for semi-empirical PM6 minimization and vibrational analysis with Gaussian16 Rev. C.01.[7] The resulting geometries were filtered for duplicates and subsequently minimized at the B3LYP/6-31++G(d,p) level, followed by vibrational analysis. More accurate enthalpies were obtained with single-point calculations at the MP2/6-31++G(d,p) level. The harmonic vibrational line spectra were frequency scaled by 0.975 and broadened using a Gaussian function with a full-width at half-maximum of 25 cm^{-1}, such as to match the experimental peak widths.

Chemical Synthesis:

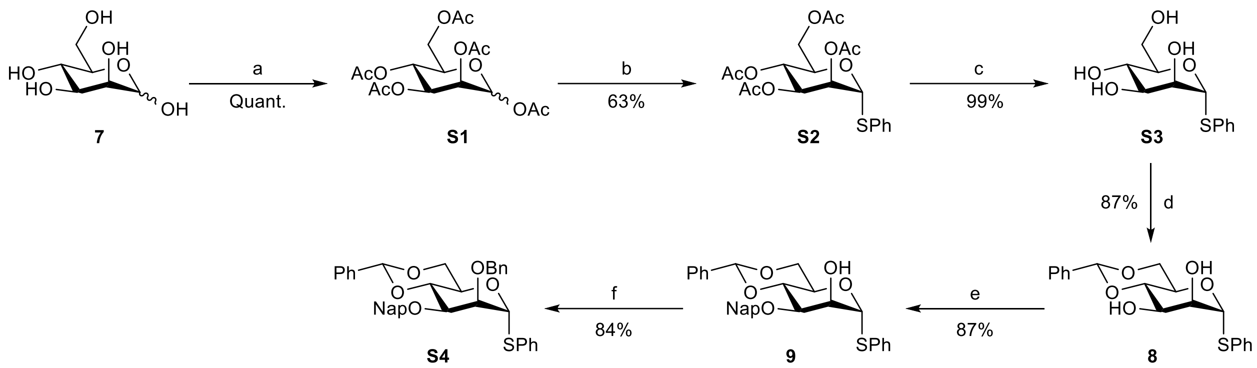

Scheme S2: Monosaccharide **7**-**9** and **S1**-**S4** synthesis. (a) Ac_2O, pyridine; (b) HSPh, $BF_3 \cdot OEt_2$, DCM; (c) NaOMe, MeOH; (d) BDA, $NaHSO_4 \cdot SiO_2$, ACN; (e) Bu_2SnO, Tol; NapBr, CsF, DMF; (f) BnBr, NaH, DMF.

1,2,3,4,6-Penta-*O*-acetyl-D-mannopyranoside (S1)

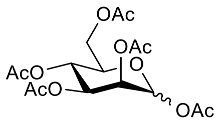

S1

Pyridine (300 ml) was added to a solution of **7** (25.0 g, 0.141 mol, 1.00 eq) in Ac_2O (150 ml, 1.59 mol, 11 eq) at 0°C. The reaction mixture was stirred at 0°C R.T. for 17 hrs. The solution was evaporated *in vacuo*. The residue was dissolved in EtOAc (150 ml). The solution was washed with 1.0 M aq. HCl (150 ml), H_2O (150 ml), aq. $NaHCO_3$ (sat.) (150 ml) and brine (150 ml), respectively. The organic layer was dried with $MgSO_4$, filtered and evaporated *in vacuo*, yielding monosaccharide **S1** as a pale yellow oil (55.2 g, 0.141 mol, quantitative, α:β = 2.2:1).

α-Anomer: **TLC**: (EtOAc/Hept, 60:40, v/v): R_f = 0.55; **^{1}H NMR** (500 MHz, $CDCl_3$): δ 6.08 (d, *J* = 2.0 Hz, 1H, ***H-1***), 5.37 – 5.33 (m, 2H, ***H-3***, ***H-4***), 5.26 (t, *J* = 2.3 Hz, 1H, ***H-2***), 4.34 – 4.24 (m, 1H, ***H-6a***), 4.17 – 4.04 (m, 2H, ***H-5***, ***H-6b***), 2.18 (s, 3H, C***H3***, OAc), 2.18 (s, 3Hz, C***H3***, OAc), 2.09 (s, 3H, C***H3***, OAc), 2.06 (s, 3H, C***H3***, OAc), 2.01 (s, 3H, C***H3***, OAc); **^{13}C NMR** (126 MHz, $CDCl_3$): δ 170.56 (***C***=O**,** OAc), 169.94 (***C***=O**,** OAc), 169.71 (***C***=O**,** OAc), 169.53 (***C***=O**,** OAc), 168.06 (***C***=O**,** OAc), 90.61(***C-1***), 70.62 (***C-5***), 68.78 (***C-3***), 68.36 (***C-2***), 65.54 (***C-4***), 62.10 (***C-6***), 20.82 (***C***H3, OAc), 20.74 (***C***H3, OAc), 20.68 (***C***H3, OAc), 20.64 (***C***H3, OAc), 20.61 (***C***H3, OAc); **β-Anomer:** **TLC**: (EtOAc/Hept, 60:40, v/v): R_f = 0.55; **^{1}H NMR** (500 MHz, $CDCl_3$): δ 5.90 (d, *J* = 1.2 Hz, 1H, ***H-1***), 5.49 (dd, *J* = 3.4, 1.2 Hz, 1H, ***H-2***), 5.29 (t, J = 9.9 Hz, 1H, ***H-4***), 5.18 (dd, *J* = 10.0, 3.3 Hz, 1H, ***H-3***), 4.34 – 4.24 (m, 1H, ***H-6a***), 4.17 – 4.04 (m, 1H, ***H-6b***), 3.85 (ddd, *J* = 9.9, 5.3, 2.4 Hz, 1H, ***H-5***), 2.21 (s, 3H, C***H3***, OAc), 2.10 (s, 3H, C***H3***, OAc), 2.06 (s, 3H, C***H3***, OAc), 2.04 (s, C***H3***, OAc), 2.00 (s, C***H3***, OAc); **^{13}C NMR** (126 MHz, $CDCl_3$): δ 170.59 (***C***=O**,** OAc), 170.17(***C***=O**,** OAc), 169.75 (***C***=O**,** OAc), 169.59 (***C***=O**,** OAc), 168.35 (***C***=O**,** OAc), 90.45 (***C-1***), 73.20 (***C-5***), 70.65 (***C-3***), 68.27 (***C-2***), 65.48 (***C-4***), 62.10 (***C-6***), 20.75 (***C***H3, OAc), 20.72 (***C***H3, OAc), 20.70 (***C***H3, OAc), 20.65 (***C***H3, OAc), 20.51 (***C***H3, OAc); **HR-ESI-TOF/Ms** (m/z): [M+Na]+ calcd for $C_{16}H_{22}O_{11}Na$, 413.10598; found, 413.10521.

Phenyl 2,3,4,6-tetra-O-acetyl-1-thio-α-D-mannopyranoside (S2)

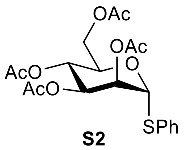

S2

Monosaccharide **S1** (34.6 g, 88.6 mmol, 1.00 eq) was dissolved in anh. DCM (180 ml). The solution was cooled down to 0°C. Thiophenol (11.8 ml, 115 mmol, 1.3 eq) was added. Next, $BF_3·OEt_2$ (38.9 ml, 310 mmol, 3.5 eq) was carefully added, after which the mixture was stirred at 0°C – R.T. for 48 hrs, turning purple over time. The solution was poured in ice-cold water (500 mL). The organic layer was extracted and subsequently washed with aq. NaHCO3 (sat.) (3x 100 ml) and brine (3x 100 ml), respectively. The organic layer was dried over $MgSO_4$, filtered and evaporated *in vacuo*. The residue was recrystallized in Et_2O/cyclohexane, yielding thioglycoside **S2** as white crystals (39.0 g, 89.5 mmol, 63.3%).

TLC: (EtOAc/Hept, 60:40, v/v): R_f = 0.67; **<u>^{1}H NMR</u>** (500 MHz, $CDCl_3$): δ 7.51 – 7.47 (m, 2H, 2x Ar***H***, SPh), 7.34 – 7.29 (m, 2H, 3x Ar***H***, SPh), 5.53 – 5.47 (m, 2H, ***H-1***, ***H-2***), 5.38 – 5.26 (m, 2H, ***H-3***, ***H-4***), 4.60 – 4.51 (m, 1H, ***H-5***), 4.31 (dd, *J* = 12.2, 5.9 Hz, 1H, ***H-6a***), 4.11 (dd, *J* = 12.2, 2.4 Hz, 1H, ***H-6b***), 2.15 (s, 3H, C***H3***, OAc), 2.08 (s, 3H, C***H3***, OAc), 2.05 (s, 3H, C***H3***, OAc), 2.02 (s, 3H, C***H3***, OAc); **<u>^{13}C NMR</u>** (126 MHz, $CDCl_3$): δ 170.56 (***C***=O**,** OAc), 169.93 (***C***=O**,** OAc), 169.84 (***C***=O**,** OAc), 169.76 (***C***=O**,** OAc), 132.63 (Ar***C***S, SPh), [132.07, 129.19, 128.13 (SPh)], 85.70 (***C-1***), 70.91 (***C-2***), 69.52 (***C-5***), 69.38 (***C-3***), 66.38 (***C-4***), 62.45 (***C-6***), 20.89 (***C***H_3, OAc), 20.72 (***C***H_3, OAc), 20.70 (***C***H_3, OAc), 20.65 (***C***H_3, OAc); **<u>HR-ESI-TOF/Ms</u>** (m/z): [M+Na]+ calcd for $C_{20}H_{24}O_9SNa$, 463.10387; found, 463.10232.

Phenyl 1-thio-α-D-mannopyranoside (S3)

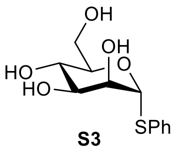

S3

Thioglycoside **S2** (39.0 g, 88.5 mmol, 1.00 eq) was dissolved in anh. MeOH (443 ml). 5.4 M NaOMe in MeOH (6.56 mL, 35.4 mmol, 0.40 eq) was added. The mixture was stirred at R.T. for 21 hrs. Dowex® ion exchange(H+) resin (2.0 g) was added, after which the mixture was stirred for an additional 20 min. The suspension was filtered and evaporated *in vacuo*, yielding monosaccharide **S3** as a white foam (23.9 g, 87.8 mmol, 99.1%).

<u>TLC:</u> (MeOH/DCM, 10:90, v/v): R_f = 0.10; **<u>^{1}H NMR</u>** (500 MHz, DMSO-d_6): δ 7.53 –7.26 (m, 5H, Ar***H***, SPh), 5.34 (d, *J* = 1.5 Hz, 1H, ***H-1***), 5.13 (d, *J* = 4.2 Hz, 1H, 2-O***H***), 4.88 (d, *J* = 5.2 Hz, 1H, 4-O***H***), 4.79 (d, *J* = 5.2 Hz, 1H, 3-O***H***), 4.50 (t, *J* = 6.0 Hz, 1H, 6-O***H***), 3.91 – 3.85 (ddd, *J* = 4.4, 2.9, 1.6 Hz, 1H, ***H-2***), 3.77 (ddd, *J* = 8.6, 6.1, 2.1 Hz, 1H, ***H-5***), 3.66 (ddd, *J* = 11.8, 5.7, 2.2 Hz, 1H, ***H-6a***), 3.55 – 3.42 (m, 3H, ***H-3***, ***H-4***, ***H-6b***); **<u>^{13}C NMR</u>** (126 MHz, DMSO-d_6; solvent peak ref'd to 39.52): δ 134.91 (Ar***C***S, SPh), [131.04, 129.02, 127.05 (SPh)], 88.87 (***C-1***), 75.39 (***C-5***), 71.92 (***C-2***), 71.53 (***C-3***), 67.00 (***C-4***), 60.89 (***C-6***); **<u>HR-ESI-TOF/Ms</u>** (m/z): [M+Na]+ calcd for $C_{12}H_{16}O_5SNa$, 295.06161; found, 295.06176.

Phenyl 4,6-O-benzylidene-1-thio-α-D-mannopyranoside (8)

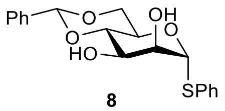

8

Thioglycoside **S3** (23.7 g, 87.0 mmol, 1.0 eq) was suspended in anh. ACN (396 ml). Benzaldehyde dimethyl acetal (14.4 ml, 95.7 mmol, 1.1 eq) and $NaHSO_4 \cdot SiO_2$ (7.47 g, 42% Wt, 17.4 mmol, 0.20 eq) were added. The mixture was stirred at R.T. for 45 min. The reaction mixture was neutralized with TEA. The suspension was filtered and washed with ACN. The residue was partially dissolved in DCM (2.0 L) and filtered. The remaining residue was dissolved in hot ethanol and filtered. The combined filtrates were evaporated *in vacuo*, yielding monosaccharide **8** as a white solid (27.3 g, 75.7 mmol, 87.0%).

TLC: (EtOAc/Hept, 50:50 v/v): R_f = 0.35; **^{1}H NMR** (500 MHz, DMSO-d_6): δ 7.51 – 7.44 (m, 4H, 2x Ar***H***, SPh; 2x Ar***H***, $PhCHO_2$), 7.41 – 7.29 (m, 6H, 3x Ar***H***, SPh; 3x Ar***H***, $PhCHO_2$), 5.63 (s, 1H, PhC***H***O_2), 5.54 (d, *J* = 4.1 Hz, 1H, 2-O***H***), 5.46 (d, *J* = 1.4 Hz, 1H, ***H-1***), 5.21 (d, *J* = 6.0 Hz, 1H, 3-O***H***), 4.06 (m, 2H, ***H-5***, ***H-6a***), 4.02 – 3.99 (m, 1H, ***H***-2), 3.95 (t, *J* = 9.3 Hz, 1H, ***H-4***), 3.82 – 3.71 (m, 2H, ***H***-3, ***H***-***6b***); **^{13}C NMR** (126 MHz, DMSO-d_6; solvent peak ref'd to 39.52): δ 137.83 (Ar***C***CHO_2), 133.65 (Ar***C***S, SPh), 131.30 (SPh), [129.26, 128.85, 128.01 (SPh; $PhCHO_2$)], 127.45 (SPh), 126.38 ($PhCHO_2$), 101.17 (Ph***C***HO_2), 89.26 (***C-1***), 78.45 (***C-4***), 72.40 (***C-2***), 68.05 (***C-3***), 67.58 (***C-6***), 65.28 (***C-5***); **HR-ESI-TOF/Ms** (m/z): [M+Na]+ calcd for $C_{19}H_{20}O_5SNa$, 383.09291; found, 383.09402.

Phenyl 4,6-O-benzylidene-3-O-(naphthalene-2-ylmethyl)-1-thio-α-D-mannopyranoside (9)

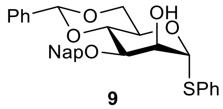

9

Thioglycoside **8** (25.4 g, 70.5 mmol, 1.00 eq) was dissolved in toluene (2.0 L). Bu_2SnO (26.3 g, 106 mmol, 1.5 eq) was added, after which the mixture was refluxed for 4 hours using a dean stark apparatus. The clear solution was concentrated *in vacuo* and resuspended in anh. DMF (250 ml). CsF (21.4 g, 141 mmol, 2.0 eq) and 2-bromomethylnaphthalene (23.4 g, 106 mmol, 1.5 eq) were added. The mixture was heated to 95°C and stirred for 18 hrs, after which the mixture was evaporated *in vacuo*. The residue was purified using silica-flash column chromatography (20 - 50% EtOAc in Hept), yielding monosaccharide **9** (30.5 g, 60.9 mmol, 86.5%).

TLC: (EtOAc/Hept, 25:75, v/v): R_f = 0.66; **^{1}H NMR** (500 MHz, $CDCl_3$): δ 7.83 – 7.80 (m, 3H, 3x Ar***H***, ONap), 7.77 – 7.72 (m, 1H, Ar***H***, ONap), 7.55 – 7.36 (m, 10H, 3x Ar***H***, ONap; 2x ArH, SPh; 5x Ar***H***, $PhCHO_2$), 7.33 – 7.24 (m, 3H, 3x Ar***H***, SPh); 5.64 (s, 1H, PhC***H***O_2), 5.60 (d, *J* = 1.2 Hz, 1H, ***H-1***), 5.03 (d, *J* = 12.1 Hz, 1H, ArC***H***$_a$$H_b$, ONap), 4.92 (d, *J* = 12.1 Hz, 1H, ArCH_a***H***$_b$, ONap), 4.37 – 4.30 (m, 2H, ***H-2***, ***H-5***), 4.25 – 4.18 (m, 2H, ***H-6a***, ***H-4***), 4.02 (dd, *J* = 9.5, 3.4 Hz, 1H, ***H-3***), 3.86 (t, *J* = 10.3 Hz, 1H, ***H-6b***); **^{13}C NMR** (126 MHz, $CDCl_3$): δ 137.48 (Ar***C***CHO_2), 135.14 (Ar***C***CH_2, ONap) , 133.27 (Ar***C***S, SPh), 133.25 (Ar***C***C_2, ONap), 133.12 (Ar***C***C_2, ONap), 131.78 (SPh), 129.16 (SPh), [129.04, 128.38, 128.30, 127.99, 127.73 (SPh; $PhCHO_2$; ONap)] , 126.71 (ONap), [126.24, 126.17, 126.11 ($PhCHO_2$; ONap)], 125.67 (ONap), 101.76 (Ph***C***HO_2), 87.81 (***C-1***), 78.99 (***C-4***), 75.71 (***C-***

3), 73.13 (Ar***C***H_2, ONap), 71.39 (***C-2***), 68.56 (***C-6***), 64.63 (***C-5***); **HR-ESI-TOF/Ms** (m/z): [M+Na]+ calcd for $C_{30}H_{28}O_5SNa$, 523.15551; found, 523.15561.

Phenyl 2-O-benzyl-4,6-O-benzylidene-3-O-(naphthalene-2-ylmethyl)-1-thio-α-D-mannopyranoside (S4)

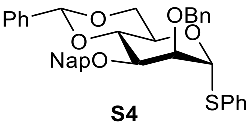

S4

Thioglycoside **9** (27.9 g, 57.3, 1.0 eq) was dissolved in anh. DMF (441 ml). The solution was cooled down to 0°C. NaH (4.59 g, 60% Wt, 115 mmol, 2.0 eq) was added. The mixture was stirred for 45 min, after which BnBr (8.18 ml, 68.8 mmol, 1.2 eq) was carefully added. The mixture was stirred at 0°C – R.T. for 48 hrs, turning orange over time. The mixture was quenched with aq. NH_4Cl and concentrated *in vacuo*. The residue was resuspended in DCM (400 mL) and washed with H_2O (400 ml) and brine (400 ml), respectively. The organic layer was dried over $MgSO_4$, filtered and evaporated *in vacuo*. The residue was purified using silica-flash column chromatography (0 – 30% EtOAc in Hept), yielding monosaccharide **S4** (27.9 g, 48.4 mmol, 84.4%) as a white foam.

TLC: (EtOAc:Hept, 25:75 v/v): R_f = 0.76; **^{1}H NMR** (500 MHz, DMSO-d_6) δ 7.94 – 7.84 (m, 3H, 3x Ar***H***, ONap), 7.77 – 7.71 (m, 1H, Ar***H***, ONap), 7.53 – 7.28 (m, 18H, 3x Ar***H***, ONap; 5x ArH, SPh; 5x Ar***H***, OBn; 5x ArH, $PhCHO_2$), 5.80 – 5.76 (m, 2H, ***H-1***, PhC***H***O_2), 4.84 – 4.77 (m, 3H, ArC***H_2***, ONap; PhC***H_a***H_b, OBn), 4.69 (d, *J* = 11.8 Hz, 1H, $PhCH_a$***H_b***, OBn), 4.28 – 4.19 (m, 2H, ***H-2***, ***H-4***), 4.16 – 4.05 (m, 2H, ***H-5***, ***H-6a***), 3.91 (dd, *J* = 10.0, 3.2 Hz, 1H, ***H-3***), 3.85 (t, *J* = 10.0 Hz, 1H, ***H-6b***); **^{13}C NMR** (126 MHz, DMSO-d_6) δ 138.17 (Ar***C***CH_2, OBn), 137.76 (Ar***C***CHO_2), 135.06 (Ar***C***CH_2, ONap), 133.03 (Ar***C***S, SPh), 132.77 (Ar***C***C_2, ONap), 132.43 (Ar***C***C_2, ONap), 131.47 (SPh), [129.24, 128.84, 128.23, 128.12, 127.94, 127.79, 127.64, 127.62, 127.59 (SPh; ONap; OBn; $PhCHO_2$)], 126.16 ($PhCHO_2$), [125.87, 125.75 , 125.72 (ONap)], 100.73 (Ph***C***HO_2), 85.75 (***C-1***), 77.78 (***C-4***), 77.12 (***C-2***), 75.60 (***C-3***), 72.03 (Ph***C***H_2, OBn), 70.87 (Ph***C***H_2, ONap), 67.45 (***C-6***), 65.28 (***C-5***); **HR-ESI-TOF/Ms** (m/z): [M+Na]+ calcd for $C_{37}H_{34}O_5S$, 613.20246; found, 613.20264.

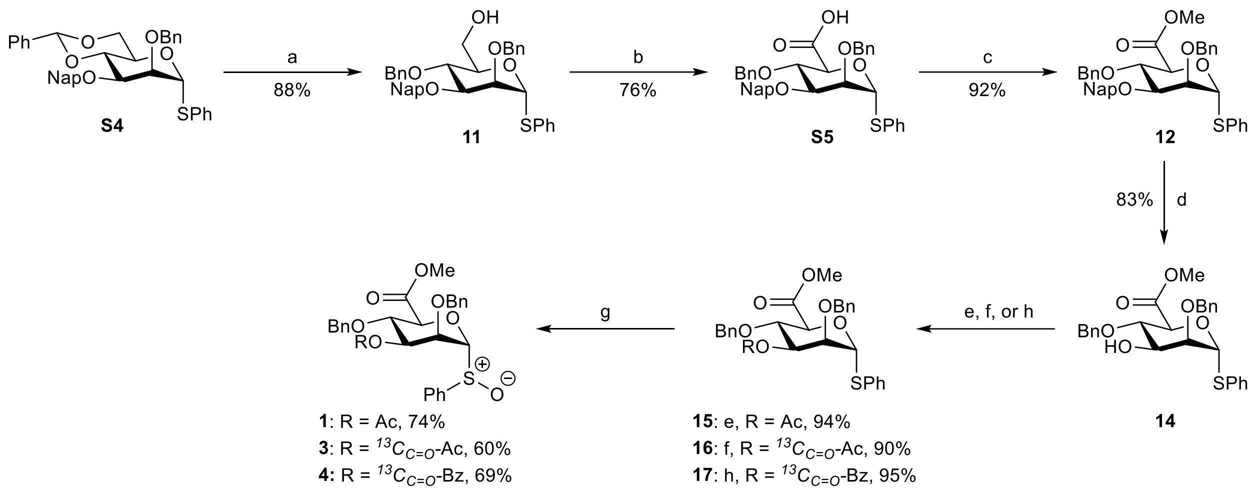

Scheme S3: Monosaccharide **S5** – **1**, **3**, **4** and **11-17** synthesis. (a) $PhBCl_2$, TES, DCM; (b) TEMPO, BAIB, DCM, H_2O; (c) MeI, K_2CO_3, DMF; (d) DDQ, DCM, H_2O; (e) Ac_2O, pyridine; (f) $^{13}C_{C=O}$-AcOH, DMAP, DIC, DCM; (g) mCPBA, DCM; (h) $^{13}C_{C=O}$-BzOH, DMAP, DIC, DCM.

Phenyl 2,4-di-*O*-benzyl-3-*O*-(naphthalene-2-ylmethyl)-1-thio-α-D-mannopyranoside (11)

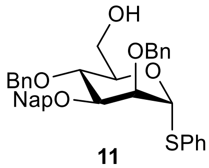

11

Thioglycoside **S4** (3.07 g, 5.20 mmol, 1.0 eq) was dissolved in anh. DCM (50 mL). The solution was cooled down to 0°C. M.S. (4Å) were added, after which the solution was stirred for 60 min. The solution was cooled down to -78°C. TES (1.30 mL, 7.98 mmol, 1.5 eq) and $PhBCl_2$ (1.25 mL, 9.38 mol, 1.8 eq) were added. The solution was stirred at -78°C for 50 min. It was then quenched with MeOH:TEA (6.0 mL, 1:1 v/v), warmed up to R.T. and filtered. The filtrate was diluted with DCM (50 mL) and washed with aq. $NaHCO_3$ (sat.) (50 mL). The organic layer was dried with $MgSO_4$, filtered and evaporated *in vacuo*. The residue was purified using silica-flash column chromatography (0 – 30% EtOAc in Hept), yielding monosacccharide **11** as a white foam (2.70 g, 4.56 mmol, 88%).

TLC: (EtOAc:Hept, 50:50 v/v): R_f = 0.44; **^{1}H NMR** (500 MHz, $CDCl_3$): δ 7.86 – 7.74 (m, 4H, 4x Ar***H***, ONap), 7.50 – 7.45 (m, 3H, 3x Ar***H***, ONap), 7.39 – 7.24 (m, 15H, 5x Ar***H***, SPh; 10x Ar***H***, OBn), 5.51 (d, *J* = 1.8 Hz, 1H, ***H-1***), 4.99 (d, *J* = 10.9 Hz, 1H, PhC$\boldsymbol{H_a}$$H_b$, 4-OBn), 4.80 (d, *J* = 12.0 Hz, 1H, ArC$\boldsymbol{H_a}$$H_b$, ONap), 4.77 (d, *J* = 12.0 Hz, 1H, ArC$H_a$$\boldsymbol{H_b}$, ONap), 4.73 – 4.66 (m, 3H, PhC$H_a$$\boldsymbol{H_b}$, 4-OBn; PhC$\boldsymbol{H_2}$, 2-OBn;), 4.13 (ddd, *J* = 9.7, 4.4, 2.8 Hz, 1H, ***H-5***), 4.07 (t, *J* = 9.4 Hz, 1H, ***H-4***), 4.02 (dd, *J* = 3.0, 1.8 Hz, 1H, ***H-2***), 3.96 (dd, *J* = 9.0, 3.0 Hz, 1H, ***H-3***), 3.87 – 3.77 (m, 2H, ***H-6a***, ***H-6b***), 1.82 (dd, *J* = 7.4, 5.8 Hz, 1H, 6-O***H***); **^{13}C NMR** (126 MHz, $CDCl_3$): 138.32 (Ar***C***CH_2, 4-OBn), 137.85 (Ar***C***CH_2, 2-OBn), 135.59 (Ar***C***CH_2, ONap), 133.91 (Ar***C***S, SPh), 133.31 (Ar***C***C_2, ONap), 133.02 (Ar***C***C_2, ONap), 131.88 (SPh), [129.12, 128.47, 128.22, 128.04, 127.97, 127.93, 127.85, 127.79, 127.72, 127.67 (SPh; OBn; ONap)], [126.56, 126.15, 125.95, 125.85 (ONap)], 86.01 (***C-1***), 80.13 (***C-3***), 76.44 (***C-2***), 75.33 (Ph***C***H_2, 4-OBn), 74.79 (***C-4***), 73.28 (***C-5***), 72.34 (Ph***C***H_2, 2-OBn), 72.32 (Ar***C***H_2, ONap), 62.25 (***C-6***); **HR-ESI-TOF/MS (m/z):** $[M+Na]^+$ calcd for $C_{37}H_{36}O_5SNa$, 615.21811; found, 615.21592.

Phenyl 2,4-di-*O*-benzyl-3-*O*-(naphthalene-2-ylmethyl)-1-thio-α-D-mannopyranosiduronic acid (S4)

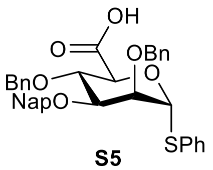

S5

Thioglycoside **11** (3.51 g, 5.92 mmol, 1.0 eq) was dissolved in DCM:H_2O (60 mL, 9:1 v/v). BAIB (5.75 g, 17.9 mmol, 3.0 eq) and TEMPO (207 mg, 1.32 mmol, 0.22 eq) were added, after which the solution was stirred at R.T. for 150 min. The reaction was quenched with 10% aq. $Na_2S_2O_3$ (80 mL) and stirred for an additional 10 min. The mixture was acidified to pH <3 with citric acid and extracted with EtOAc (3x 120 mL). The combined organic layers were dried with $MgSO_4$, filtered and evaporated *in vacuo*. The residue was purified using silica-flash column chromatography (0 – 60% EtOAc in Hept + 2% AcOH), yielding uronic acid **S5** as a pale yellow oil (2.72 g, 4.50 mmol, 76%).

TLC: (EtOAc:Hept:AcOH, 40:58:2 v/v): R_f = 0.21; **^{1}H NMR** (500 MHz, $CDCl_3$; measured at 50°C): δ 7.81 – 7.77 (m, 1H, Ar***H***, ONap), 7.75 – 7.70 (m, 2H, 2x Ar***H***, ONap), 7.69 (s, 1H, Ar***H***, ONap), 7.51 – 7.47 (m, 2H, 2x Ar***H***, SPh), 7.46 – 7.42 (m, 2H, 2x ArH, ONap), 7.37 (dd, *J* = 8.4, 1.7 Hz, 1H, ArH, ONap), 7.30 – 7.20 (m, 13H, 3x Ar***H***, SPh; 10x Ar***H***, OBn), 5.66 (d, *J* = 5.1 Hz, 1H, ***H-1***), 4.73 – 4.62 (m, 5H, PhC$\boldsymbol{H_a}H_b$, 2-OBn; PhC$\boldsymbol{H_2}$, 4-OBn; PhC$\boldsymbol{H_2}$, ONap), 4.57 (d, *J* = 6.5 Hz, 1H, ***H-5***), 4.52 (d, *J* = 12.0 Hz, 1H, PhC$H_a\boldsymbol{H_b}$, 2-OBn), 4.22 (t, *J* = 6.8 Hz, 1H, ***H-4***), 3.92 (dd, *J* = 5.2, 2.9 Hz, 1H, ***H-2***), 3.87 (dd, *J* = 7.0, 2.8 Hz, 1H, ***H-3***); **^{13}C NMR** (126 MHz, $CDCl_3$; measured at 50°C): δ 172.20 (***C-6***), 137.87 (Ar***C***CH_2, 2-OBn), 137.68 (Ar***C***CH_2, 4-OBn), 135.28 (Ar***C***CH_2, ONap), 133.64 (Ar***C***S, SPh), 133.38 (Ar***C***C_2, ONap), 133.18 (Ar***C***C_2, ONap), 131.75 (SPh), [129.08, 128.48, 128.45, 128.27, 128.06, 128.04, 128.01, 127.95, 127.86, 127.77, 127.57 (SPh; OBn; ONap)], [126.68, 126.20, 126.03, 125.86 (ONap)], 84.58 (***C-1***), 77.30 (***C-3***), 75.93 (***C-4***), 75.36 (***C-2***), 74.07 (Ph***C***H_2, 4-OBn), [72.66, 72.62 (Ph***C***H_2, 2-OBn; Ph***C***H_2, ONap)], 72.22 (***C-5***). **HR-ESI-TOF/MS (m/z):** $[M+Na]^+$ calcd for $C_{37}H_{34}O_6SNa$, 629.19738; found, 629.19513.

Methyl (phenyl 2,4-di-*O*-benzyl-3-*O*-(naphthalene-2-ylmethyl)-1-thio-α-D-mannopyranosyl uronate) (12)

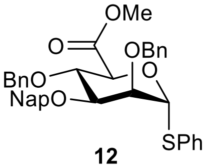

12

Thioglycoside **S5** (1.2 g, 2.0 mmol, 1.0 eq) was dissolved in anh. DMF (11 mL). MeI (0.25 mL, 4.0 mmol, 2.0 eq) and K_2CO_3 (0.57 g, 4.1 mmol, 2.1 eq) were added, after which the solution was stirred at R.T. for 66 hrs. The reaction was quenched with MeOH (1.0 mL). The solution was diluted with EtOAc (50 mL) and washed with H_2O (50 mL) and brine (50 mL), respectively. The combined organic layers were dried with $MgSO_4$, filtered and evaporated *in vacuo*. The residue was purified using silica-flash column chromatography (5 – 30% EtOAc in Hept), yielding monosaccharide **12** as a colourless oil (1.1 g, 1.8 mmol, 92%).

TLC: (EtOAc:Hept, 40:60 v/v): R_f = 0.48; **^{1}H NMR** (500 MHz, $CDCl_3$; measured at 50°C): δ 7.83 – 7.78 (m, 1H, Ar***H***, ONap), 7.78 – 7.69 (m, 3H, 3x Ar***H***, ONap), 7.54 – 7.48 (m, 2H, 2x Ar***H***, SPh), 7.48 – 7.43 (m, 2H, 2x Ar***H***, ONap), 7.39 (dd, *J* = 8.4, 1.7 Hz, 1H, Ar***H***,

ONap), 7.32 – 7.20 (m, 13H, 3x Ar***H***, SPh; 10x Ar***H***, OBn), 5.67 (d, *J* = 5.4 Hz, 1H, ***H-1***), 4.73 – 4.62 (m, 4H, PhC$\boldsymbol{H_2}$, ONap; PhC$\boldsymbol{H_a}$H$_b$, 2-OBn; PhC$\boldsymbol{H_2}$, 4-OBn), 4.61 (d, *J* = 6.2 Hz, 1H, ***H-5***), 4.53 (d, *J* = 12.0 Hz, 1H, PhCH$_a$$\boldsymbol{H_b}$, 2-OBn), 4.29 (t, *J* = 6.6 Hz, 1H, ***H-4***), 3.92 (dd, *J* = 5.5, 2.9 Hz, 1H, ***H-2***), 3.87 (dd, *J* = 7.0, 2.9 Hz, 1H, ***H-3***), 3.63 (s, 3H, C$\boldsymbol{H_3}$, OMe); **<u>^{13}C NMR</u>** (126 MHz, $CDCl_3$; measured at 50°C): 169.68 (***C-6***), 138.13 (Ar***C***CH_2, OBn), 138.03 (Ar***C***CH_2, OBn), 135.48 (Ar***C***CH_2, ONap), 134.04 (Ar***C***S, SPh), 133.39 (Ar***C***C_2, ONap), 133.17 (Ar***C***C_2, ONap), 131.60 (SPh), [128.94, 128.41, 128.18, 128.05, 127.99, 127.83, 127.78, 127.75, 127.32 (SPh; OBn; ONap)], [126.64, 126.16, 125.98, 125.91 (ONap)], 84.49 (***C-1***), 77.36 (***C-3***), 76.30 (***C-4***), 75.42 (***C-2***), 73.80 (Ph***C***H_2, 4-OBn), 73.22 (***C-5***), 72.74 (Ph***C***H_2, ONap), 72.60 (Ph***C***H_2, 2-OBn), 52.14 (***C***H_3, OMe); **<u>HR-ESI-TOF/MS (m/z)</u>:** $[M+Na]^+$ calcd for $C_{38}H_{36}O_6SNa$, 643.21303; found, 643.21278.

Methyl (phenyl 2,4-di-*O*-benzyl-1-thio-α-D-mannopyranosyl uronate) (14)

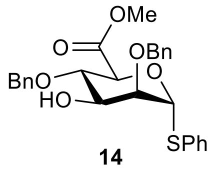

14

Thioglycoside **12** (1.12 g, 1.80 mmol, 1.0 eq) was dissolved in DCM:H_2O (25 mL, 9:1 v/v). DDQ (758 mg, 3.34 mmol, 1.6 eq) was added. The solution was vigorously stirred under exclusion of light for 3 hrs. The mixture was diluted with DCM (25 mL), quenched with DDQ mixture (50 mL) and stirred for an additional 10 min. The organic layer was extracted and subsequently washed with DDQ mixture (50 mL) and aq. $NaHCO_3$ (sat.) (50 mL), respectively. The combined organic layers were dried with $MgSO_4$, filtered and evaporated *in vacuo*. The residue was purified using silica-flash column chromatography (0 – 10% EtOAc in Tol), yielding monosaccharide **14** as a colourless oil (717 mg, 1.49 mmol, 83%).

<u>TLC</u>: (EtOAc:Tol, 14:86 v/v): R_f = 0.44; **<u>^{1}H NMR</u>** (500 MHz, $CDCl_3$): δ 7.55 – 7.52 (m, 2H, 2x Ar***H***, SPh), 7.37 – 7.24 (m, 13H, 3x Ar***H***, SPh; 10x Ar***H***, OBn), 5.65 (d, *J* = 4.7 Hz, 1H, ***H-1***), 4.75 (d, *J* = 11.5 Hz, 1H, PhC$\boldsymbol{H_a}$H$_b$, 2-OBn), 4.72 (d, *J* = 11.4 Hz, 1H, PhC$\boldsymbol{H_a}$H$_b$, 4-OBn), 4.66 (d, *J* = 11.3 Hz, 1H, PhCH$_a$$\boldsymbol{H_b}$, 4-OBn), 4.64 (d, *J* = 6.5 Hz, 1H, ***H-5***), 4.52 (d, *J* = 11.5 Hz, 1H, PhCH$_a$$\boldsymbol{H_b}$, 2-OBn), 4.06 (t, *J* = 6.9 Hz, 1H, ***H-4***), 4.00 (ddd, *J* = 7.3, 6.2, 3.3 Hz, 1H, ***H-3***), 3.92 (dd, *J* = 4.7, 3.3 Hz, 1H, ***H-2***), 3.74 (s, 3H, C$\boldsymbol{H_3}$, OMe), 2.42 (d, *J* = 6.1 Hz, 1H, 3-O***H***); **<u>^{13}C NMR</u>** (126 MHz, $CDCl_3$): δ 169.68 (***C-6***), 137.90 (Ar***C***CH_2, 4-OBn), 137.25 (Ar***C***CH_2, 2-OBn), 133.65 (Ar***C***S, SPh), 131.53 (SPh), [129.03, 128.63, 128.44, 128.21, 128.12, 127.87, 127.48 (SPh; OBn)], 83.60 (***C-1***), 77.52 (***C-4***), 77.15 (***C-2***), 73.88 (Ph***C***H_2, 4-OBn), 72.67 (Ph***C***H_2, 2-OBn), 72.13 (***C-5***), 70.26 (***C-3***), 52.31 (***C***H_3, OMe); **<u>HR-ESI-TOF/MS (m/z)</u>:** $[M+Na]^+$ calcd for $C_{27}H_{28}O_6SNa$, 503.15043; found, 503.14883.

Methyl (phenyl 3-*O*-acetyl-2,4-di-*O*-benzyl-1-thio-α-D-mannopyranosyl uronate) (15)

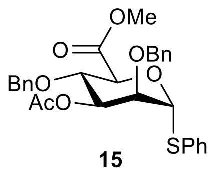

15

Thioglycoside **14** (0.25 g, 0.51 mmol, 1.0 eq) was dissolved in pyridine (4.0 mL). Ac_2O (2.0 mL, 21 mmol, 42 eq) was added, after which the solution was stirred at R.T. for 40 hrs. The solution was evaporated *in vacuo*. The residue was dissolved in EtOAc (30 mL). The solution was washed with H_2O (15 mL), aq. $NaHCO_3$ (sat.) (15 mL) and brine (15 mL), respectively. The organic layer was dried with $MgSO_4$, filtered and evaporated *in vacuo*. The residue was purified using silica-flash column chromatography (0 – 8% EtOAc in Tol), yielding monosaccharide **15** as a colourless oil (0.25 g, 0.48 mmol, 94%).

TLC: (EtOAc:Tol, 10:90 v/v): R_f = 0.40; **^{1}H NMR** (500 MHz, $CDCl_3$; measured at 50°C): δ 7.57 – 7.52 (m, 2H, 2x Ar***H***, SPh), 7.35 – 7.23 (m, 13H, 3x Ar***H***, SPh; 10x Ar***H***, OBn), 5.55 (d, *J* = 5.1 Hz, 1H, ***H-1***), 5.25 (dd, *J* = 7.3, 3.1 Hz, 1H, ***H-3***), 4.69 – 4.64 (m, 2H, ***H-5***, PhC$\boldsymbol{H_a}$$H_b$, 4-OBn), 4.64 – 4.57 (m, 2H, PhC$\boldsymbol{H_a}$$H_b$, 2-OBn, PhC$H_a$$\boldsymbol{H_b}$, 4-OBn), 4.49 (d, *J* = 11.9 Hz, 1H, PhC$H_a$$\boldsymbol{H_b}$, 2-OBn), 4.24 (t, *J* = 6.9 Hz, 1H, ***H-4***), 4.00 (dd, *J* = 5.1, 3.1 Hz, 1H, ***H-2***), 3.73 (s, 3H, C$\boldsymbol{H_3}$, OMe), 1.96 (s, 3H, C$\boldsymbol{H_3}$, OAc); **^{13}C NMR** (126 MHz, $CDCl_3$; measured at 50°C): δ 169.78 (***C***=O, OAc), 169.44 (***C-6***), 137.69 (Ar***C***CH_2, 4-OBn), 137.33 (Ar***C***CH_2, 2-OBn), 133.43 (Ar***C***S, SPh), 131.81 (SPh), [129.05, 128.98, 128.45, 128.12, 128.00, 127.89, 127.69, 127.56 (SPh; OBn)], 84.15 (***C-1***), 75.06 (***C-4***), 74.63 (***C-2***), 73.84 (Ph***C***H_2, 4-OBn), 72.72 (***C-5***), 72.38 (Ph***C***H_2, 2-OBn), 70.96 (***C-3***), 52.41 (***C***H_3, OMe), 20.88 (***C***H_3, OAc); **HR-ESI-TOF/MS (m/z):** $[M+Na]^+$ calcd for $C_{29}H_{30}O_7SNa$, 545.16099; found, 545.15969.

Methyl (phenyl 3-*O*-$^{13}C_{C=O}$-acetyl-2,4-di-*O*-benzyl-1-thio-α-D-mannopyranosyl uronate) (16)

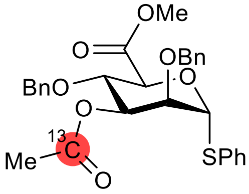

16

Thioglycoside **14** (0.15 g, 0.32 mmol, 1.0 eq) was dissolved in DCM (5.0 mL). $^{13}C_{C=O}$-AcOH (28 µL, 0.50 mmol, 1.6 eq), DMAP (5.0 mg, 41 µmol, 0.13 eq) and DIC (0.10 mL, 0.65 mmol, 2.0 eq) were added, after which the solution was stirred at R.T. for 2.5 hrs. The solution was cooled down to -20˚C, after which the precipitate was filtered. The filtrate was evaporated *in vacuo*. The residue was purified using silica-flash column chromatography (0 – 8% EtOAc in Tol), yielding monosaccharide **16** as a colourless oil (0.15 g, 0.29 mmol, 90%).

TLC: (EtOAc:Tol, 10:90 v/v): R_f = 0.42; **^{1}H NMR** (500 MHz, $CDCl_3$; measured at 50°C): δ 7.58 – 7.51 (m, 2H, 2x Ar***H***, SPh), 7.35 – 7.22 (m, 13H, 3x Ar***H***, SPh; 10x Ar***H***, OBn), 5.55 (d, *J* = 5.0 Hz, 1H, ***H-1***), 5.25 (dt, *J* = 6.9, 3.2 Hz, 1H, ***H-3***), 4.69 – 4.64 (m, 2H, ***H-5***, PhC$\boldsymbol{H_a}$$H_b$, 4-OBn), 4.64 – 4.57 (m, 2H, PhC$\boldsymbol{H_a}$$H_b$, 2-OBn, PhC$H_a$$\boldsymbol{H_b}$, 4-OBn), 4.49 (d, *J* = 11.8 Hz, 1H, PhC$H_a$$\boldsymbol{H_b}$, 2-OBn), 4.24 (t, *J* = 7.0 Hz, 1H, ***H-4***), 4.00 (dd, *J* = 5.0, 3.2 Hz, 1H, ***H-2***), 3.73 (s, 3H, C$\boldsymbol{H_3}$, OMe), 1.95 (d, *J* = 6.9 Hz, 3H, C$\boldsymbol{H_3}$, OAc); **^{13}C NMR** (126 MHz, $CDCl_3$; solvent peak ref'd to 77.16; measured at 50°C): δ 169.83 (***^{13}C***=O, OAc),

169.55 (***C-6***), 138.01 (Ar***C***CH_2, 4-OBn), 137.65 (Ar***C***CH_2, 2-OBn), 133.71 (Ar***C***S, SPh), 132.10 (SPh), [129.11, 128.58, 128.57, 128.22, 128.10, 127.98, 127.83, 127.70 (SPh; OBn)], 84.55 (***C-1***), 75.37 (***C-4***), 75.11 (***C-2***), 73.99 (Ph***C***H_2, 4-OBn), 73.02 (***C-5***), 72.65 (Ph***C***H_2, 2-OBn), 71.30 (d, *J* = 1.6 Hz, ***C-3***), 52.39 (***C***H_3, OMe), 20.92 (d, *J* = 59.9 Hz, ***C***H_3, OAc); **HR-ESI-TOF/MS (m/z):** $[M+Na]^+$ calcd for $C_{28}{}^{13}CH_{30}O_7SNa$, 546.16435; found, 546.16242.

Methyl (phenyl 3-*O*-acetyl-2,4-di-*O*-benzyl-1-sulfinyl-α-D-mannopyranosyl uronate) (1)

Thioglycoside **15** (205 mg, 392 µmol, 1.0 eq) was dissolved in DCM (10 mL). The solution was cooled down to -78°C and purged with Argon. mCPBA (96.0 mg, 428 µmol, 1.1 eq) was added, after which the solution was stirred at -78°C for 6 hrs. The solution was then stirred at -78°C – R.T. for an additional 14 hrs. The solution was diluted with DCM (10 mL), quenched by the addition of 10% aq. $Na_2S_2O_3$ (20 mL) and stirred for an additional 10 min. The organic layer was extracted and subsequently washed with aq. $NaHCO_3$ (sat.) (2x 20 mL). The organic layer was dried with $MgSO_4$, filtered and evaporated *in vacuo*. The residue was purified using silica-flash column chromatography (0 – 30% EtOAc in toluene), yielding sulfoxide **1** as a colourless oil (157 mg, 291 µmol, 74%).

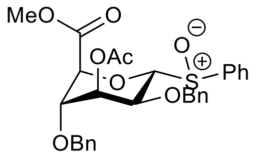

1_{eq}

Diastereomer A | TLC: (EtOAc:Tol, 30:70 v/v): R_f = 0.37; **^{1}H NMR** (500 MHz, $CDCl_3$): δ 7.72 – 7.69 (m, 2H, 2x Ar***H***, SOPh), 7.55 – 7.47 (m, 3H, 3x Ar***H***, SOPh), 7.38 – 7.29 (m, 10H, 10x Ar***H***, OBn), 5.46 (dd, *J* = 4.1, 2.9 Hz, 1H, ***H-3***), 4.86 (d, *J* = 9.5 Hz, 1H, ***H-1***), 4.69 – 4.64 (m, 2H, PhC$\boldsymbol{H_a}$$H_b$, 2-OBn; PhC$\boldsymbol{H_a}$$H_b$, 4-OBn), 4.62 (d, *J* = 10.8 Hz, 1H, PhC$H_a$$\boldsymbol{H_b}$, 2-OBn), 4.56 (d, *J* = 12.2 Hz, 1H, PhC$H_a$$\boldsymbol{H_b}$, 4-OBn), 4.51 (d, *J* = 2.4 Hz, 1H, ***H-5***), 4.41 (dd, *J* = 9.5, 2.9 Hz, 1H, ***H-2***), 4.07 (dd, *J* = 4.1, 2.5 Hz, 1H, ***H-4***), 3.57 (s, 3H, C$\boldsymbol{H_3}$, OMe), 1.97 (s, 3H, C$\boldsymbol{H_3}$, OAc); **^{13}C NMR** (126 MHz, $CDCl_3$): δ 169.26 (***C***=O, OAc), 168.86 (***C-6***), 139.65 (Ar***C***SO), 137.13 (Ar***C***CH_2, 4-OBn), 136.84 (Ar***C***CH_2, 2-OBn), 130.74 (SOPh), 128.69 (SOPh), [128.61, 128.56, 128.25, 128.11, 127.88 (OBn)], 125.22 (SOPh), 87.76 (***C-1***), 75.32 (***C-4***), 74.93 (***C-5***), [72.55, 72.51 (Ph***C***H_2, 2-OBn; Ph***C***H_2, 4-OBn)], 69.44 (***C-2***), 68.04 (***C-3***), 52.03 (***C***H_3, OMe), 20.75 (***C***H_3, OAc);

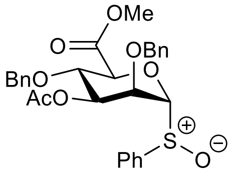

1_{ax}

Diastereomer B | TLC: (EtOAc:Tol, 30:70 v/v): R_f = 0.26; **^{1}H NMR** (500 MHz, $CDCl_3$): δ 7.62 – 7.58 (m, 2H, 2x Ar***H***, SOPh), 7.47 – 7.42 (m, 3H, 3x Ar***H***, SOPh), 7.37 – 7.29 (m, 3H, 3x Ar***H***, OBn), 7.29 – 7.25 (m, 3H, 3x Ar***H***, OBn), 7.24 – 7.20 (m, 2H, 2x Ar***H***, OBn), 7.13 – 7.09 (m, 2H, 2x Ar***H***, OBn), 5.43 (dd, *J* = 5.7, 3.2 Hz, 1H, ***H-3***), 4.99 (d, *J* = 6.8 Hz, 1H, ***H-1***), 4.63 – 4.60 (m, 2H, ***H-5***, PhC$\boldsymbol{H_a}$$H_b$, 4-OBn), 4.57 (d, *J* = 11.8 Hz, 1H, PhC$H_a$$\boldsymbol{H_b}$, 4-OBn), 4.37 (s, 2H, PhC$\boldsymbol{H_2}$, 2-OBn), 4.19 (dd, *J* = 6.8, 3.2 Hz, 1H, ***H-2***), 4.15 (dd, *J* = 5.7, 4.4 Hz, 1H, ***H-4***), 3.73 (s, 3H, C$\boldsymbol{H_3}$, OMe), 1.94 (s, 3H, C$\boldsymbol{H_3}$, OAc); **^{13}C NMR** (126 MHz, $CDCl_3$): δ 169.39 (***C***=O, OAc), 168.97 (***C-6***), 140.40 (Ar***C***SO), 137.33 (Ar***C***CH_2, 4-OBn), 136.91 (Ar***C***CH_2, 2-OBn), 131.12 (SOPh), 128.97 (SOPh), [128.48, 128.34, 128.12, 127.97, 127.93, 127.62 (OBn)],

125.06 (SOPh), 91.73 (***C-1***), 75.09 (***C-5***), 75.04 (***C-4***), 72.90 (Ph***C***H_2, 4-OBn), 71.33 (Ph***C***H_2, 2-OBn), 69.69 (***C-2***), 68.83 (***C-3***), 52.46 (***C***H_3, OMe), 20.78 (***C***H_3, OAc); **HR-ESI-TOF/MS (m/z):** $[M+Na]^+$ calcd for $C_{29}H_{30}O_8SNa$, 561.15591; found, 561.15356.

Methyl (phenyl 3-*O*-$^{13}C_{C=O}$-acetyl-2,4-di-*O*-benzyl-1-sulfinyl-α-D-mannopyranosyl uronate) (3)

Thioglycoside **16** (0.13 g, 0.25 mmol, 1.0 eq) was dissolved in DCM (10 mL). The solution was cooled down to -78°C and purged with Argon. mCPBA (57 mg, 0.25 mmol, 1.0 eq) was added, after which the solution was stirred at -78°C for 6 hrs. The solution was then stirred at -78°C – R.T. for an additional 18 hrs. The solution was diluted with DCM (10 mL), quenched by the addition of 10% aq. $Na_2S_2O_3$ (20 mL) and stirred for an additional 10 min. The organic layer was extracted and subsequently washed with aq. $NaHCO_3$ (sat.) (20 mL). The organic layer was dried with $MgSO_4$, filtered and evaporated *in vacuo*. The residue was purified using silica-flash column chromatography (0 – 30% EtOAc in Tol), yielding sulfoxide **3** as a colourless oil (81 mg, 0.15 mmol, 60%).

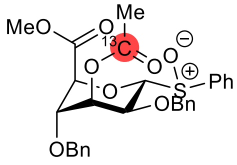

3_{eq}

Diastereomer A | TLC: (EtOAc:Tol, 30:70 v/v): R_f = 0.37; **^{1}H NMR** (500 MHz, $CDCl_3$): δ 7.74 – 7.69 (m, 2H, 2x Ar***H***, SOPh), 7.56 – 7.47 (m, 3H, 3x Ar***H***, SOPh), 7.39 – 7.29 (m, 10H, 10x Ar***H***, OBn), 5.46 (q, *J* = 3.6 Hz, 1H, ***H-3***), 4.86 (d, *J* = 9.5 Hz, 1H, ***H-1***), 4.69 – 4.64 (m, 2H, PhC$\boldsymbol{H_a}$$H_b$, 2-OBn; PhC$\boldsymbol{H_a}$$H_b$, 4-OBn), 4.62 (d, *J* = 10.8 Hz, 1H, PhC$H_a$$\boldsymbol{H_b}$, 2-OBn), 4.56 (d, *J* = 12.2 Hz, 1H, PhC$H_a$$\boldsymbol{H_b}$, 4-OBn), 4.51 (d, *J* = 2.4 Hz, 1H, ***H-5***), 4.41 (dd, *J* = 9.5, 2.9 Hz, 1H, ***H-2***), 4.07 (dd, *J* = 4.0, 2.4 Hz, 1H, ***H-4***), 3.57 (s, 3H, C$\boldsymbol{H_3}$, OMe), 1.97 (d, *J* = 6.9 Hz, 3H, C$\boldsymbol{H_3}$, OAc); **^{13}C NMR** (126 MHz, $CDCl_3$; solvent peak ref'd to 77.16): δ 169.39 (***C***=O, OAc), 168.99 (***C-6***), 139.76 (Ar***C***SO), 137.26 (Ar***C***CH_2, 4-OBn), 136.97 (Ar***C***CH_2, 2-OBn), 130.87 (SOPh), 128.82 (SOPh), [128.74, 128.69, 128.38, 128.24, 128.01 (OBn)], 125.35 (SOPh), 87.88 (***C-1***), 75.45 (***C-4***), 75.06 (***C-5***), [72.68, 72.64 (Ph***C***H_2, 2-OBn; Ph***C***H_2, 4-OBn)], 69.57 (***C-2***), 68.17 (d, *J* = 2.6 Hz, ***C-3***), 52.16 (***C***H_3, OMe), 20.87 (d, *J* = 60.4 Hz, ***C***H_3, OAc);

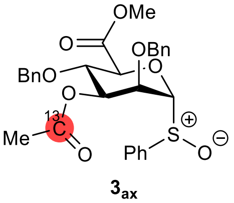

3_{ax}

Diastereomer B | TLC: (EtOAc:Tol, 30:70 v/v): R_f = 0.27; **^{1}H NMR** (500 MHz, $CDCl_3$): δ 7.62 – 7.58 (m, 2H, 2x Ar***H***, SOPh), 7.47 – 7.42 (m, 3H, 3x Ar***H***, SOPh), 7.37 – 7.29 (m, 3H, 3x Ar***H***, OBn), 7.29 – 7.25 (m, 3H, 3x Ar***H***, OBn), 7.24 – 7.20 (m, 2H, 2x Ar***H***, OBn), 7.13 – 7.09 (m, 2H, 2x Ar***H***, OBn), 5.43 (dt, *J* = 5.7, 3.4 Hz, 1H, ***H-3***), 4.99 (d, *J* = 6.8 Hz, 1H, ***H-1***), 4.64 – 4.60 (m, 2H, ***H-5***, PhC$\boldsymbol{H_a}$$H_b$, 4-OBn), 4.57 (d, *J* = 11.8 Hz, 1H, PhC$H_a$$\boldsymbol{H_b}$, 4-OBn), 4.37 (s, 2H, PhC$\boldsymbol{H_2}$, 2-OBn), 4.19 (dd, *J* = 6.8, 3.2 Hz, 1H, ***H-2***), 4.15 (dd, *J* = 5.7, 4.4 Hz, 1H, ***H-4***), 3.73 (s, 3H, C$\boldsymbol{H_3}$, OMe), 1.94 (d, *J* = 6.9 Hz, 3H, C$\boldsymbol{H_3}$, OAc); **^{13}C NMR** (126 MHz, $CDCl_3$): δ 169.38 (***C***=O, OAc), 168.96 (***C-6***), 140.39 (Ar***C***SO), 137.32 (Ar***C***CH_2, 4-OBn), 136.90 (Ar***C***CH_2, 2-OBn), 131.12 (SOPh), 128.96 (SOPh), [128.47, 128.33, 128.11, 127.96, 127.92, 127.61 (OBn)], 125.05 (SOPh), 91.73 (***C-1***), 75.08 (***C-5***), 75.04 (***C-4***), 72.90 (Ph***C***H_2, 4-OBn), 71.33 (Ph***C***H_2, 2-OBn), 69.69 (***C-2***), 68.82 (***C-3***), 52.46 (***C***H_3, OMe), 20.77 (d, *J* = 60.0 Hz, ***C***H_3,

OAc); **HR-ESI-TOF/MS (m/z):** $[M+Na]^+$ calcd for $C_{28}{}^{13}CH_{30}O_8SNa$, 562.15926; found, 562.15676.

Methyl (phenyl 3-*O*-$^{13}C_{C=O}$-benzoyl-2,4-di-*O*-benzyl-1-thio-α-D-mannopyranosyl uronate) (17)

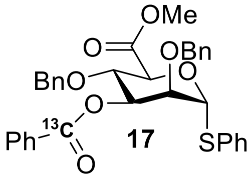

Thioglycoside **14** (95 mg, 0.20 mmol, 1.0 eq) was dissolved in DCM (2.0 mL). $^{13}C_{C=O}$-BzOH (39 mg, 0.32 mmol, 1.6 eq), DMAP (3 mg, 26 µmol, 0.13 eq) and DIC (62 µL, 0.20 mmol, 2.0 eq) were added, after which the solution was stirred at R.T. for 12 hrs. The solution was evaporated *in vacuo*. The residue was purified using silica-flash column chromatography (0 – 20% EtOAc in heptane), yielding monosaccharide **17** as a white waxy solid (110 mg, 188 µmol, 95%).

TLC: (EtOAc/Hept, 50:50, v/v): R_f = 0.76; **^{1}H NMR** (500 MHz, CHLOROFORM-*D*) δ 8.09 – 7.95 (m, 2H, 2x Ar***H***, OBz), 7.65 – 7.54 (m, 3H, 1x Ar***H***, OBz, 2x Ar***H***, SPh), 7.49 – 7.39 (m, 2H, 2x Ar***H***, OBz), 7.35 – 7.29 (m, 3H, 3x Ar***H***, SPh), 7.29 – 7.14 (m, 10H, 10x Ar***H***, OBn), 5.72 (d, *J* = 5.4 Hz, 1H, ***H-1***), 5.52 (dt, *J* = 6.8, 3.2 Hz, 1H, ***H-3***), 4.74 (d, *J* = 6.2 Hz, 1H, ***H-5***), 4.70 (d, *J* = 11.3 Hz, 1H, PhC$\boldsymbol{H_a}$H$_b$, 4-OBn), 4.66 (d, *J* = 11.3 Hz, 1H, PhCH$_a$$\boldsymbol{H_b}$, 4-OBn), 4.60 (d, *J* = 11.9 Hz, 1H, PhC$\boldsymbol{H_a}$H$_b$, 2-OBn), 4.51 (d, *J* = 11.9 Hz, 1H, PhC$\boldsymbol{H_a}$H$_b$, 2-OBn), 4.41 (t, *J* = 6.6 Hz, 1H, ***H-4***), 4.11 (dd, *J* = 5.4, 3.2 Hz, 1H, ***H-2***), 3.60 (s, 3H, C$\boldsymbol{H_3}$, OMe). **^{13}C NMR** (126 MHz, CHLOROFORM-*D*) δ 169.33 (***C-6***), 165.42 (***^{13}C***=O, OBz), 137.50 (Ar***C***CH$_2$, 4-OBn), 137.27 (Ar***C***CH$_2$, 2-OBn), 133.50 (Ar***C***S, SPh), [133.43, 131.66, 129.88, 129.86, 129.69, 129.10 (Ar***^{13}C***CH$_2$, OBz), 128.54, 128.50, 128.41, 128.35, 128.01, 127.89, 127.85, 127.81, 127.46 (SPh; OBn)], 83.87 (***C-1)***, 75.11 (***C-4***), 74.48 (***C-2***), 73.84 (Ph***C***H$_2$, 4-OBn), 72.86 (***C-5***), 72.36 (Ph***C***H$_2$, 2-OBn), 71.35 (***C-3***), 52.38 (***C***H$_3$, OMe). **HR-ESI-TOF/MS (m/z):** $[M+H]^+$ calcd for $C_{33}{}^{13}CH_{32}O_7SH$, 586.1975; found, 586.1976.

Methyl (phenyl 3-*O*-$^{13}C_{C=O}$-benzoyl-2,4-di-*O*-benzyl-1-thiosulfinyl-α-D-mannopyranosyl uronate) (4)

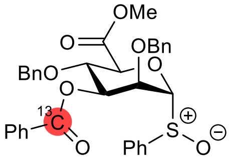

4_{ax}

Thioglycoside **17** (70 mg, 120 µmol, 1.0 eq) was dissolved in DCM (1.2 mL) under argon atmosphere. The solution was cooled down to -78°C. mCPBA (23 mg, 132 µmol, 1.1 eq) was carefully added, after which the solution was stirred for 2.5 hrs. The mixture was diluted with DCM (10 mL) and quenched by the addition of 10% aq. $Na_2S_2O_3$ (10 mL). The organic layer was extracted and subsequently washed with aq. $NaHCO_3$ (sat.) (10 mL). The organic layer was dried with $MgSO_4$, filtered and evaporated *in vacuo*. The residue was purified using silica-flash column chromatography (0 – 30% EtOAc in Tol), yielding sulfoxide **4** as a white waxy solid (50 mg, 83 µmol, 69%).

Diastereomer A | TLC: (EtOAc/Hept, 50:50, v/v): R_f = 0.56; **^{1}H NMR** (500 MHz, CHLOROFORM-*D*) δ 7.94 (m, 2H, 2x Ar***H***, OBz), 7.84 – 7.70 (m, 2H, 2x Ar***H***, SOPh), 7.60 – 7.39 (m, 6H, 3x Ar***H***, SOPh, 3x Ar***H***, SOPh), 7.39 – 7.21 (m, 10H, Ar***H*** OBn), 5.73 (q, *J* = 3.4 Hz, 1H, ***H-3***), 5.11 (d, *J* = 9.6 Hz, 1H, ***H-1***), 4.71 (d, *J* = 12.2 Hz, 1H, PhC$\boldsymbol{H_a}$$H_b$, 4-OBn), 4.69 (s, 2H, PhC$\boldsymbol{H_2}$, 2-OBn), 4.61 (d, *J* = 12.2 Hz, 1H, PhC$H_a$$\boldsymbol{H_b}$, 4-OBn), 4.54 – 4.52 (m, 2H, ***H-2***; ***H-5***), 4.20 (dd, *J* = 4.0, 2.1 Hz, 1H, ***H-4***), 3.31 (s, 3H, C$\boldsymbol{H_3}$, OMe).**^{13}C NMR** (126 MHz, CHLOROFORM-*D*) δ 168.73 (***C-6***), 165.12 (***^{13}C***=O, OBz), 139.41 (Ar***C***S, SOPh), 137.16 (Ar***C***CH_2, 4-OBn), 136.91 (Ar***C***CH_2, 2-OBn), [133.71, 133.62, 131.07 (Ar***^{13}C***CH_2, OBz), 130.25, 129.90 (Ar***C***H, OBz), 129.88 (Ar***C***H, OBz), 129.83, 128.75, 128.58, 128.55, 128.52, 128.46, 128.29, 128.12, 128.10, 127.87 (Ar***C***H; SPh; OBn, OBz)], 125.36 (Ar***C***H, SOPh), 87.41 (***C-1***), 75.45 (***C-4***), 74.83 (***C-5***), 72.58 (***C***H_2; 4-OBn), 72,51 (***C***H_2; 2-OBn), 69.55 (***C-2***), 68.31 (***C-3***), 52.06 (***C***H_3, OMe).

Diastereomer B | TLC: (EtOAc/Hept, 50:50, v/v): R_f = 0.48; **^{1}H NMR** (500 MHz, CHLOROFORM-*D*) δ 7.96 – 7.90 (m, 2H, 2x Ar***H***, OBz), 7.63 (dd, *J* = 6.6, 2.7 Hz, 2H, 2x Ar***H***, SOPh), 7.60 – 7.55 (m, 1H, 1x Ar***H***, OBz), 7.50 – 7.38 (m, 5H, 2x Ar***H***, OBz, 3x Ar***H***, SOPh), 7.36 – 6.96 (m, 10H, 10x Ar***H***, OBn), 5.79 – 5.59 (m, 1H, ***H-3***), 5.21 (d, *J* = 7.1 Hz, 1H, ***H-1***), 4.70 – 4.58 (m, 3H, ***H-5***, PhC$\boldsymbol{H_2}$, 4-OBn), 4.44 (d, *J* = 11.6 Hz, 1H, PhC$\boldsymbol{H_a}$$H_b$, 2-OBn), 4.38 (d, *J* = 11.6 Hz, 1H, PhC$H_a$$\boldsymbol{H_b}$, 4-OBn), 4.32 – 4.24 (m, 2H, ***H-2***; ***H-4***), 3.51 (s, 3H, C$\boldsymbol{H_3}$, OMe). **^{13}C NMR** (126 MHz, CHLOROFORM-*D*) δ 168.81 (***C-6***), 165.14 (***^{13}C***=O, OBz), 140.30 (Ar***C***S, SOPh), 137.26 (Ar***C***CH_2, 4-OBn), 136.92 (Ar***C***CH_2, 2-OBn), 133.51 (Ar***C***H, OBz), 131.08 (Ar***C***H, SOPh), 129.88 (Ar***C***H, OBz), 129.86 (Ar***C***H, OBz), [128.92, 128.52, 128.48, 128.25, 127.97, 127.78, 127.66 (Ar***C***H, SOPh, OBn, OBz)], 125.14 (Ar***C***H, SOPh), 91.55 (***C-1***), 75.13 (***C-4***), 75.04 (***C-5***), 72.90 (***C***H_2; 2-OBn), 71.29 (***C***H_2; 4-OBn), 69.80 (***C-2***), 69.12 (***C-3***), 52.42 (***C***H_3, OMe).**HR-ESI-TOF/MS (m/z):** $[M+Na]^+$ calcd for $C_{33}{}^{13}CH_{32}O_7SNa$, 624.1744; found, 624.1732.

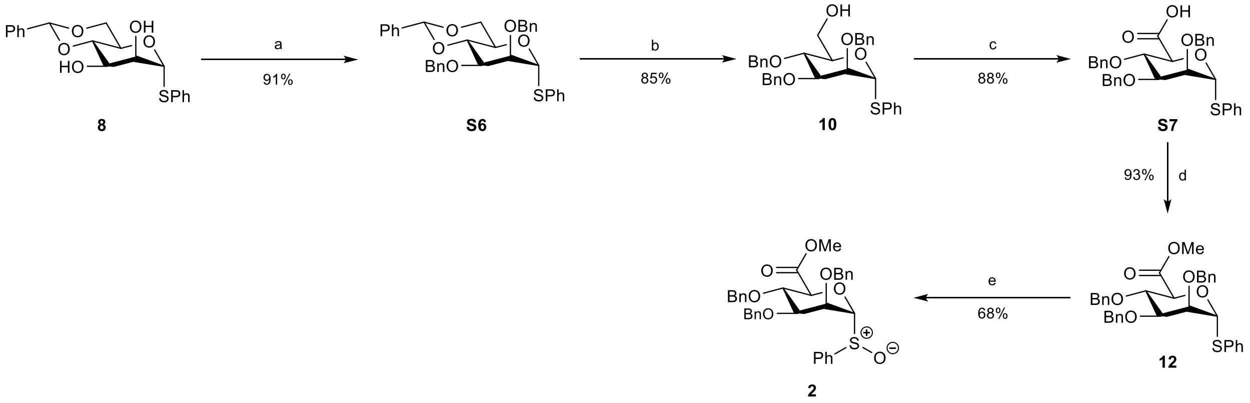

Scheme S4: Monosaccharide **2**, **10**, and **12**, **S6** and **S7** synthesis. (a) BnBr, NaH, DMF; (b) $PhBCl_2$, TES, DCM; (c) TEMPO, BAIB, DCM, H_2O; (d) MeI, K_2CO_3, DMF; (e) mCPBA, DCM.

Phenyl 2,3-di-*O*-benzyl-4,6-*O*-benzylidene-1-thio-α-D-mannopyranoside (S6)

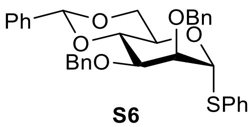

S6

Thioglycoside **8** (1.4 g, 3.9 mmol, 1.0 eq) was dissolved in anh. DMF (39 mL). The solution was cooled down to 0°C. NaH (0.48 g, 60% Wt, 12 mmol, 3.1 eq) and BnBr (1.4 mL, 12 mmol, 3.0 eq) were added, after which the solution was stirred at 0°C – R.T. for 16 hrs. The reaction was quenched with MeOH (3.0 mL) and stirred for an additional 15 min. The solution was evaporated *in vacuo*. The residue was resuspended in EtOAc (60 mL) and washed with H_2O (50 mL), aq. $NaHCO_3$ (sat.) (50 mL) and brine (50 mL), respectively. The organic layer was dried with $MgSO_4$, filtered and evaporated *in vacuo*. The residue was purified using silica-flash column chromatography (0 – 60% EtOAc in Hept), yielding monosaccharide **S6** a pale yellow oil (1.7 g, 3.9 mmol, 81%).

<u>TLC</u>: (EtOAc:Hept, 50:50 v/v): R_f = 0.59; **<u>^{1}H NMR</u>** (500 MHz, $CDCl_3$): δ 7.53 – 7.50 (m, 2H,), 7.41 – 7.25 (m, 18H,), 5.65 (s, 1H, PhC***H***O_2), 5.51 (d, *J* = 1.5 Hz, 1H, ***H-1***), 4.82 (d, *J* = 12.2 Hz, 1H, PhC***H$_a$***H_b, 3-OBn), 4.72 (s, 2H, PhC***H$_2$***, 2-OBn), 4.65 (d, *J* = 12.2 Hz, 1H, PhCH_a***H$_b$***, 3-OBn), 4.35 – 4.25 (m, 2H, ***H-4***, ***H-5***), 4.22 (dd, *J* = 10.2, 4.4 Hz, 1H, ***H-6$_a$***), 4.04 (dd, *J* = 3.3, 1.4 Hz, 1H, ***H-2***), 3.97 (dd, *J* = 9.4, 3.2 Hz, 1H, ***H-3***), 3.88 (t, *J* = 9.9 Hz, 1H, ***H-6$_b$***); **<u>^{13}C NMR</u>** (126 MHz, $CDCl_3$): δ 138.38 (Ar***C***CH_2, 3-OBn), 137.74 (Ar***C***CH_2, 2-OBn), 137.60 (Ar***C***CHO_2), 133.78 (Ar***C***S, SPh), [131.64, 129.14, 128.88, 128.44, 128.37, 128.20, 128.11, 127.87, 127.66, 127.63 (SPh; OBn; PhCHO_2)], 126.10 (PhCHO_2), 101.51 (Ph***C***HO_2), 87.13 (***C-1***), 79.10 (***C-4***), 78.09 (***C-2***), 76.23 (***C-3***), [73.10, 73.06 (Ph***C***H_2, 2-OBn; Ph***C***H_2, 3-OBn)], 68.52 (***C-6***), 65.45 (***C-5***); **<u>HR-ESI-TOF/MS (m/z)</u>:** $[M+Na]^+$ calcd for $C_{33}H_{32}O_5SNa$, 563.18681; found, 563.18428.

Phenyl 2,3,4-tri-*O*-benzyl-1-thio-α-D-mannopyranoside (10)

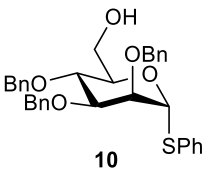

10

Thioglycoside **S6** (1.4 g, 2.5 mmol, 1.0 eq) was dissolved in anh. DCM (25 mL). The solution was cooled down to 0°C. M.S. (4Å) were Added, after which the mixture was stirred for 2 hrs. The mixture was cooled down to -78°C. TES (0.61 mL, 3.8 mmol, 1.5 eq) and PhBCl2 (0.60 mL, 4.5 mmol, 1.8 eq) were added. The reaction was stirred for 70 min, after which it was quenched by the addition of MeOH:TEA (4.0 mL, 1:1 v/v). The mixture was warmed up to R.T. and filtered. The filtrate was diluted with DCM (25 mL) and subsequently washed with aq. $NaHCO_3$ (sat.) (25 mL). The organic layer was dried with $MgSO_4$, filtered and evaporated *in vacuo*. The residue was purified using silica-flash column chromatography (0 – 40% EtOAc in Hept), yielding monosaccharide **10** as a colourless oil (1.2 g, 2.1 mmol, 85%).

<u>TLC</u>: (EtOAc:Hept, 50:50 v/v): R_f = 0.47; **<u>^{1}H NMR</u>** (500 MHz, $CDCl_3$): δ 7.40 – 7.24 (m, 20H, 15x Ar***H***, OBn; 5x Ar***H***, SPh), 5.51 (d, *J* = 1.6 Hz, 1H, ***H-1***), 4.95 (d, *J* = 10.9 Hz, 1H, PhC***H$_a$***H_b, 4-OBn), 4.68 (s, 2H, PhC***H$_2$***, 2-OBn), 4.67 – 4.63 (m, 2H, PhC***H$_a$***H_b, 3-OBn; PhCH_a***H$_b$***, 4-OBn), 4.61 (d, *J* = 11.7 Hz, 1H, PhCH_a***H$_b$***, 3-OBn), 4.12 (ddd, *J* = 9.7, 4.5, 2.8 Hz, 1H, ***H-5***), 4.03 (t, *J* = 9.4 Hz, 1H, ***H-4***), 3.99 (dd, *J* = 3.0, 1.8 Hz, 1H, ***H-2***), 3.89 (dd, *J* = 9.2, 3.0 Hz, 1H, ***H-3***), 3.85 – 3.76 (m, 2H, ***H-6$_a$***, ***H-6$_b$***), 1.81 (dd, *J* = 7.4, 5.8 Hz,

1H, 6-O***H***); **^{13}C NMR** (126 MHz, $CDCl_3$): δ 138.32 (Ar***C***CH_2, 4-OBn), 138.13 (Ar***C***CH_2, 3-OBn), 137.88 (Ar***C***CH_2, 2-OBn), 133.95 (Ar***C***S), 131.87 (SPh), [129.12, 128.46, 128.08, 127.93, 127.84, 127.80, 127.77, 127.66 (SPh; OBn)], 86.05 (***C-1***), 80.12 (***C-3***), 76.43 (***C-2***), 75.30 (Ph***C***H_2, 4-OBn), 74.81 (***C-4***), 73.24 (***C-5***), 72.35 (Ph***C***H_2, 2-OBn), 72.25 (Ph***C***H_2, 3-OBn), 62.25 (***C-6***); **HR-ESI-TOF/MS (m/z):** $[M+Na]^+$ calcd for $C_{33}H_{34}O_5SNa$, 565.20246; found, 565.19987.

Phenyl 2,3,4-tri-*O*-benzyl-1-thio-α-D-mannopyranosiduronic acid (S7)

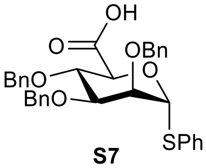

S7

Thioglycoside **10** (1.12 g, 2.06 ml, 1.0 eq) was dissolved in DCM:H_2O (20 mL, 9:1 v/v). BAIB (2.05 g, 6.24 mmol, 3.0 eq) and TEMPO (159 mg, 1.02 mmol, 0.49 eq) were added, after which the solution was stirred at R.T. for 2 hrs. The solution was diluted with DCM (20 mL), quenched by the addition of 10% aq. $Na_2S_2O_3$ (40 mL) and stirred for an additional 15 min. The mixture was acidified to pH <3 with citric acid. The organic layer was extracted and the aqueous layer was extracted with EtOAc (3x 40 mL). The combined organic layers were dried with $MgSO_4$, filtered and evaporated *in vacuo*. The residue was purified using silica-flash column chromatography (5 – 40% EtOAc in Hept + 2% AcOH), yielding mannuronic acid **S7** as a white foam (1.01 g, 1.82 mmol, 88%).

TLC: (EtOAc:Hept:AcOH, 48:52:2 v/v): R_f = 0.27; **^{1}H NMR** (500 MHz, $CDCl_3$; measured at 50°C): δ 7.51 – 7.47 (m, 2H, 2x Ar***H***, SPh), 7.33 – 7.22 (m, 18H, 3x Ar***H***, SPh; 15x Ar***H***, OBn), 5.64 (d, *J* = 4.9 Hz, 1H, ***H-1***), 4.73 – 4.60 (m, 4H, ***H-5***, PhC***H**a*** H_b, 2-OBn; PhC***H₂*** , 4-OBn), 4.60 – 4.52 (m, 3H, PhCH_a***H*b**, 2-OBn; PhC***H_2***, 3-OBn), 4.21 (t, *J* = 7.0 Hz, 1H, ***H-4***), 3.91 (dd, *J* = 4.9, 2.8 Hz, 1H, ***H-2***), 3.83 (dd, *J* = 7.2, 2.8 Hz, 1H, ***H-3***); **^{13}C NMR** (126 MHz, $CDCl_3$; measured at 50°C): δ 172.06 (***C-6***), 137.87 (Ar***C***CH_2, OBn), 137.83 (Ar***C***CH_2, OBn), 137.71 (Ar***C***CH_2, OBn), 133.63 (Ar***C***S, SPh), 131.77 (SPh), [129.10, 128.49, 128.48, 128.46, 128.09, 128.06, 127.97, 127.90, 127.87, 127.61 (SPh; OBn)], 84.74 (***C-1***), 77.49 (***C-3***), 75.96 (***C-4***), 75.39 (***C-2***), 74.20 (Ph***C***H_2, 4-OBn), [72.60 , 72.58 (Ph***C***H_2, 2-OBn; Ph***C***H_2, 3-OBn)], 72.20 (***C-5***); **HR-ESI-TOF/MS (m/z):** $[M+Na]^+$ calcd for $C_{33}H_{32}O_6SNa$, 579.18173; found, 579.18093.

Methyl (phenyl 2,3,4-tri-*O*-benzyl-1-thio-α-D-mannopyranosyl uronate) (12)

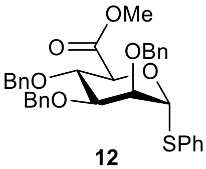

12

Thioglycoside **S7** (0.52 g, 0.93 mmol, 1.0 eq) was dissolved in anh. DMF (5.0 mL). MeI (0.12 mL, 1.9 mmol, 2.1 eq) and K_2CO_3 (0.27 g, 1.9 mmol, 2.1 eq) were added, after which the mixture was stirred at R.T. for 64 hrs. The reaction was quenched with MeOH (1.0 mL). The mixture was diluted with EtOAc (25 mL) and washed with H_2O (25 mL) and brine (25 mL), respectively. The organic layer was dried with $MgSO_4$, filtered and evaporated *in vacuo*. The residue was purified using silica-flash column chromatography (5 – 30% EtOAc in Hept), yielding monosaccharide **12** as a colourless oil (0.49 g, 0.87 mmol, 93%).

TLC: (EtOAc:Hept, 50:50 v/v): R_f = 0.56; **^{1}H NMR** (500 MHz, $CDCl_3$; measured at 50°C): δ 7.55 – 7.50 (m, 2H, 2x Ar***H***, SPh), 7.34 – 7.21 (m, 18H, 3x Ar***H***, SPh; 15x Ar***H***, OBn), 5.65 (d, *J* = 5.3 Hz, 1H, ***H-1***), 4.69 – 4.62 (m, 3H, PhC$\boldsymbol{H_a}$$H_b$, 2-OBn; PhC$\boldsymbol{H_2}$, 4-OBn), 4.60 (d, *J* = 6.3 Hz, 1H, ***H-5***), 4.56 (s, 2H, PhC$\boldsymbol{H_2}$, 3-OBn), 4.53 (d, *J* = 12.0 Hz, 1H, PhC$H_a$$\boldsymbol{H_b}$, 2-OBn), 4.25 (t, *J* = 6.7 Hz, 1H, ***H-4***), 3.90 (dd, *J* = 5.3, 2.9 Hz, 1H, ***H-2***), 3.82 (dd, *J* = 7.1, 2.9 Hz, 1H, ***H-3***), 3.63 (s, 3H, C$\boldsymbol{H_3}$, OMe); **^{13}C NMR** (126 MHz, $CDCl_3$; measured at 50°C): δ 169.66 (***C-6***), 138.15 (Ar***C***CH_2, OBn), 138.03 (Ar***C***CH_2, OBn), 134.06 (Ar***C***S, SPh), 131.60 (SPh), [128.94, 128.55, 128.41, 128.06, 127.91, 127.86, 127.78 (SPh; OBn)], 127.32 (SPh), 84.51 (***C-1***), 77.30 (***C-3***), 76.31 (***C-4***), 75.40 (***C-2***), 73.82 (Ph***C***H_2, 4-OBn), 73.17 (***C-5***), 72.66 (Ph***C***H_2, 3-OBn), 72.55 (Ph***C***H_2, 2-OBn), 52.11 (***C***H_3, OMe); **HR-ESI-TOF/MS (m/z):** $[M+Na]^+$ calcd for $C_{34}H_{34}O_6SNa$, 593.19738; found, 593.19440.

Methyl (phenyl 2,3,4-tri-*O*-benzyl-1-sulfinyl-α-D-mannopyranosyl uronate) (2)

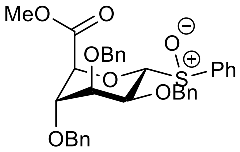

2_{eq}

Thioglycoside **12** (464 mg, 813 µmol, 1.0 eq) was dissolved in DCM (15 mL) under argon atmosphere. The solution was cooled down to -78°C. mCPBA (154 mg, 892 µmol, 1.1 eq) in DCM (2.0 mL) was carefully added, after which the solution was stirred for 3 hrs. The mixture was diluted with DCM (10 mL) and quenched by the addition of 10% aq. $Na_2S_2O_3$ (25 mL). The organic layer was extracted and subsequently washed with aq. $NaHCO_3$ (sat.) (25 mL). The organic layer was dried with $MgSO_4$, filtered and evaporated *in vacuo*. The residue was purified using silica-flash column chromatography (0 – 30% EtOAc in Toluene), yielding sulfoxide **2** as a colourless oil (322 mg, 549 µmol, 68%).

Diastereomer A | TLC: (EtOAc:Tol, 40:60 v/v): R_f = 0.49; **^{1}H NMR** (500 MHz, $CDCl_3$; measured at 50°C): δ 7.72 – 7.67 (m, 2H, 2x Ar***H***, SOPh), 7.51 – 7.12 (m, 18H, 3x Ar***H***, SOPh; 15x Ar***H***, OBn), 5.01 (d, *J* = 9.6 Hz, 1H, ***H-1***), 4.75 (d, *J* = 11.4 Hz, 1H, PhC$\boldsymbol{H_a}$$H_b$, 2-OBn), 4.61 (d, *J* = 12.3 Hz, 1H, PhC$\boldsymbol{H_a}$$H_b$, 4-OBn), 4.55 (d, *J* = 11.4 Hz, 1H, PhC$H_a$$\boldsymbol{H_b}$, 2-OBn), 4.46 – 4.38 (m, 4H, ***H-5***, PhC$\boldsymbol{H_2}$, 3-OBn, PhC$H_a$$\boldsymbol{H_b}$, 4-OBn), 4.34 (dd, *J* = 9.6, 2.6 Hz, 1H, ***H-2***), 4.12 (dd, *J* = 4.2, 1.9 Hz, 1H, ***H-4***), 3.83 (dd, *J* = 4.2, 2.6 Hz, 1H, ***H-3***), 3.37 (s, 3H, C$\boldsymbol{H_3}$, OMe); **^{13}C NMR** (126 MHz, $CDCl_3$; measured at 50°C): δ 169.15 (***C-6***), 140.25 (Ar***C***SO), 137.73 (Ar***C***CH_2, OBn), 137.59 (Ar***C***CH_2, OBn) , 137.57 (Ar***C***CH_2, OBn), 130.42 (SOPh), [128.98, 128.54, 128.49, 128.45, 128.40, 128.37, 128.29, 128.00, 127.97, 127.91, 127.88, 127.84, 127.78, 127.75, 127.69 (SOPh; OBn; *peaks of both diastereomers*)], 125.34 (SOPh), 87.85 (***C-1***), 75.82 (***C-4***), 74.82 (***C-5***), 74.37 (***C-3***), 72.84 (Ph***C***H_2, 2-OBn), 72.77 (Ph***C***H_2, 3-OBn), 72.21 (Ph***C***H_2, 4-OBn), 71.54 (***C-2***), 52.20 (***C***H_3, OMe);

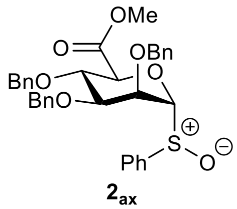

<u>Diastereomer B | TLC</u>: (EtOAc:Tol, 40:60 v/v): R_f = 0.49; **<u>^{1}H NMR</u>** (500 MHz, $CDCl_3$; measured at 50°C): δ 7.60 – 7.55 (m, 2H, 2x Ar***H***, SOPh), 7.51 – 7.12 (m, 18H, 3x Ar***H***, SOPh; 15x Ar***H***, OBn), 5.04 (d, *J* = 5.7 Hz, 1H, ***H-1***), 4.61 (s, 2H, PhC***H₂***, 4-OBn), 4.54 (d, *J* = 4.9 Hz, 1H, ***H-5***), 4.51 (s, 2H, PhC***H₂***, 3-OBn), 4.46 – 4.38 (m, 2H, PhC***H₂***, 2-OBn), 4.23 (dd, J = 6.7, 4.9 Hz, 1H, ***H-4***), 4.21 (dd, *J* = 5.5, 2.7 Hz, 1H, ***H-2***), 3.98 (dd, *J* = 6.5, 2.8 Hz, 1H, ***H-3***), 3.57 (s, 3H, C***H₃***, OMe); **<u>^{13}C NMR</u>** (126 MHz, $CDCl_3$; measured at 50°C): δ 169.09 (***C-6***), 141.36 (Ar***C***SO), 137.88 (Ar***C***CH_2, OBn), 137.86 (Ar***C***CH_2, OBn), 137.65 (Ar***C***CH_2, OBn), 130.96 (SOPh), [128.98, 128.54, 128.49, 128.45, 128.40, 128.37, 128.29, 128.00, 127.97, 127.91, 127.88, 127.84, 127.78, 127.75, 127.69 (SOPh; OBn; *peaks of both diastereomers*)], 124.89 (SOPh), 93.20 (***C-1***), 76.23 (***C-3***), 76.18 (***C-4***), 75.82 (***C-5***), 73.26 (Ph***C***H_2, 4-OBn), 72.74 (Ph***C***H_2, 3-OBn), 71.75 (Ph***C***H_2, 2-OBn), 71.32 (***C-2***), 51.71 (***C***H_3, OMe); **<u>HR-ESI-TOF/MS (m/z)</u>:** $[M+Na]^+$ calcd for $C_{34}H_{34}O_7SNa$, 609.19229; found, 609.18961.

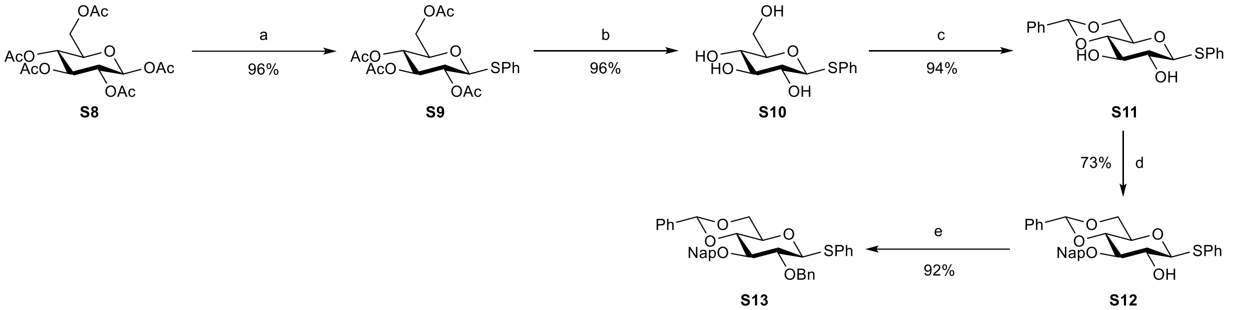

Scheme S 5: Monosaccharide **S8** – **S13** synthesis. (a) HSPh, $BF_3{\cdot}OEt_2$, DCM; (b) NaOMe, MeOH; (c) BDA, $NaHSO_4{\cdot}SiO_2$, ACN; (d) Bu_2SnO, Tol; NapBr, CsF, DMF; (e) BnBr, NaH, DMF.

Phenyl 2,3,4,6-tetra-*O*-acetate-1-thio-β-D-glucopyranoside (S9)

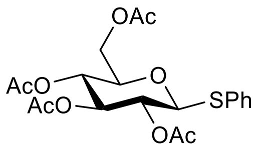

β-D-Glucose pentaacetate (**S8**, 50.0 g, 128 mmol, 1.0 eq) was dissolved in anhydrous DCM (250 mL). The solution was cooled down to 0°C. Thiophenol (33.0 mL, 324 mmol, 2.5 eq) and $BF_3{\cdot}OEt_2$ (48.0 mL, 384 mmol, 3.0 eq) were added. The solution was stirred at R.T. for 41 hrs, turning pink over time. The reaction mixture was poured on ice (250 mL). The organic layer was washed with 1.0 M aq. NaOH (100 mL), aq. $NaHCO_3$ (sat.) (100 mL). The organic layer was dried with $MgSO_4$, filtered and evaporated *in vacuo*. The residue was recrystallized from EtOH, yielding thioglycoside **S9** as white crystals (54.0 g, 128 mmol, 96%).

<u>TLC:</u> (EtOAc:Hept, 50:50 v/v): R_f = 0.40; **<u>^{1}H NMR</u>** (500 MHz, $CDCl_3$): δ 7.52 – 7.47 (m, 2H, 2x Ar***H***, SPh), 7.35 – 7.29 (m, 3H, 3x Ar***H***, SPh), 5.23 (t, *J* = 9.4 Hz, 1H, ***H-3***), 5.04 (t, *J* = 9.8 Hz, 1H, ***H-4***), 4.98 (t, *J* = 9.7 Hz, 1H, ***H-2***), 4.71 (d, *J* = 10.1 Hz, 1H, ***H-1***), 4.24 – 4.17 (m, 2H, ***H-6a***, ***H-6b***), 3.73 (ddd, *J* = 10.1, 5.1, 2.6 Hz, 1H, ***H-5***), 2.09 (s, 3H, C***H₃***, OAc), 2.08 (s, 3H, C***H₃***, OAc), 2.02 (s, 3H, C***H₃***, OAc), 1.99 (s, 3H, C***H₃***, OAc); **<u>^{13}C NMR</u>** (126 MHz, $CDCl_3$): δ 170.59 (***C***=O, OAc), 170.20 (***C***=O, OAc), 169.41 (***C***=O), 169.27 (***C***=O), 133.14 (SPh), 131.65 (Ar***C***S), 128.95 (SPh), 128.44 (SPh), 85.76 (***C-1***), 75.81

(***C-5***), 73.97 (***C-4***), 69.95 (***C-2***), 68.22 (***C-3***), 62.16 (***C-6***) , 20.76 (***C***H_3, OAc), 20.74 (***C***H_3, OAc), 20.60 (***C***H_3, OAc), 20.59 (***C***H_3, OAc); **HR-ESI-TOF/Ms(m/z):** $[M+Na]^+$ calcd for $C_{20}H_{24}O_9SNa$, 463.1033; found 463.10387.

Phenyl 1-thio-β-D-glucopyranoside (S10)

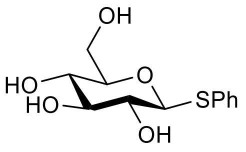

S10

Thioglycoside **S9** (51.0 g, 116 mmol, 1.0 eq) was dissolved in MeOH (1.10 L). K_2CO_3 (3.30 g, 23.9 mmol, 0.20 eq) was added. The mixture was stirred at R.T. for 66 hrs. DOWEX® 50WX8(H^+) resin was added, after which the mixture was stirred for an additional 30 min. The mixture was filtered and evaporated *in vacuo*, yielding monosaccharide **S10** as a white solid (30.0 g, 116 mmol, 96.4%).

<u>TLC:</u> (MeOH:DCM, 20:80 v/v): $R_f = 0.60$; **<u>^{1}H NMR</u>** (500 MHz, MeOD-d_4; solvent peak ref'd to 3.31)**;** δ 7.59 – 7.52 (m, 2H, 2x Ar***H***, SPh), 7.34 – 7.21 (m, 3H, 3x Ar***H***, SPh), 4.60 (dd, *J* = 9.7, 2.4 Hz, 1H, ***H-1***), 3.87 (dt, *J* = 12.2, 2.2 Hz, 1H, ***H-6a***), 3.67 (ddd, *J* = 12.1, 5.4, 2.2 Hz 1H, ***H-6b***), 3.39 (td, *J* = 8.7, 2.2 Hz, 1H, ***H-3***), 3.34 – 3.28 (m, 2H, ***H-4***, ***H-5***), 3.22 (ddd, *J* = 9.5, 8.5, 2.3 Hz, 1H, ***H-2***); **<u>^{13}C NMR</u>** (126 MHz, MeOD-d_4; solvent peak ref'd to 49.00): δ 135.28 (Ar***C***S), [132.70, 129.87, 128.31 (SPh)], 89.56 (***C-1***), 82.19(***C-5***), 79.84 (***C-4***), 73.92 (***C-2***), 71.50 (***C-3***), 63.02 (***C-6***); **HR-ESI-TOF/Ms(m/z):** $[M+Na]^+$ calcd for $C_{12}H_{16}O_5SNa$, 295.06065; found, 295.06161.

Phenyl 4,6-*O*-benzylidene-1-thio-β-D-glucopyranoside (S11)

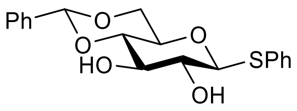

S11

Thioglycoside **S10** (20 g, 73 mmol, 1.0 eq) added to anh. ACN (0.75 L). Benzaldehyde dimethyl acetal (13 mL, 90 mmol, 1.2 eq) and $NaHSO_4 \cdot SiO_2$ (42% wt, 4.0 g, 9.3 mmol 0.08 eq) were added, The mixture was stirred at R.T. for 90 min, after which it was quenched with TEA (35 mL). The catalyst was removed by filtration over celite. The filtrate was evaporated *in vacuo*. The residue was dissolved in EtOAc (300 mL). The solution washed with aq. $NaHCO_3$ (sat.) (100 mL) and brine (100 mL), respectively. The organic layer was dried with $MgSO_4$, filtered and evaporated *in vacuo*, yielding monosaccharide **S11** as a white solid (25g, 69 mmol, 94%).

<u>TLC:</u> (EtOAc:Tol, 40:60 v/v): $R_f = 0.50$; **<u>^{1}H NMR</u>** (500 MHz, $CDCl_3$): δ 7.58 – 7.51 (m, 2H, 2x Ar***H***, SPh), 7.50 – 7.44 (m, 2H, 2x Ar***H***, $PhCHO_2$), 7.37 – 7.33 (m, 6H, 3x Ar***H***, SPh; 3x Ar***H***, $PhCHO_2$), 5.52 (s, 1H, PhC***H***O_2), 4.62 (d, J = 9.8 Hz, 1H, ***H-1***), 4.41 – 4.34 (m, 1H, 1H, ***H-6a***), 3.86 – 3.73 (m, 2H, ***H-3***, ***H-6b***), 3.54 – 3.48 (m, 2H, ***H-4***, ***H-5***), 3.45 (t, *J* = 9.1 Hz, 1H, ***H-2***), 2.93 (bs, 1H, O***H***), 2.76 (bs, 1H, O***H***); **<u>^{13}C NMR</u>** (126 MHz, $CDCl_3$): δ 136.86 (Ar***C***CHO_2), 133.07 (SPh), 131.31 (Ar***C***S, SPh), 129.36 ($PhCHO_2$), [129.15, 128.49, 128.39 (SPh; $PhCHO_2$), 126.30 ($PhCHO_2$), 101.95 (Ph***C***HO_2), 88.60 (***C-1***), 80.20 (***C-4***), 74.58 (***C-3***), 72.60 (***C-2***), 70.54 (***C-5***), 68.58 (***C-6***); **HR-ESI-TOF/Ms(m/z):** [M+Na]+calcd for $C_{19}H_{20}O_5Sna$, 383.09291; found 383.09425.

Phenyl 3-*O*-(naphthalene-2-ylmethyl)-4,6-benzylidene-1-thio-β-D-glucopyranoside (S12)

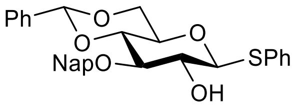

S12

Thioglycoside **S11** (19.7 g, 54.6 mmol, 1.0 eq) and Bu_2SnO (14.9 g, 60.1 mmol, 1.1 eq) were suspended in anh. toluene (150 mL). The suspension was refluxed for 41 hrs, after which the resulting clear solution was concentrated *via* distillation. The solution was cooled down to R.T. and diluted with anh. DMF (200 mL). CsF (14.4 g, 95.3 mmol, 1.7eq) and NapBr (18.1g, 81.9mmo, 1.5 eq) were added. The mixture was stirred for 3 hrs. The mixture was concentrated *in vacuo* and then diluted with DCM (1.00 L). Next, it was washed with water (400 mL) and $NaHCO_3$ (sat.) (400 mL). The organic layer was dried with Na_2SO_4, filtered and evaporated *in vacuo*. The residue was recrystallized from EtOH, yielding monosaccharide **S12** as white crystals (20.0 g, 40.0 mmol, 73%).

TLC: (EtOAc:Heptane, 50:50 v/v): R_f = 0.53; **^{1}H NMR** (500 MHz, $CDCl_3$)**:** δ 7.83 – 7.77 (m, 3H, 3x Ar***H***, ONap), 7.75 – 7.71 (m, 1H, Ar***H***, ONap), 7.56 – 7.51 (m, 2H, 2x Ar***H***, SPh), 7.51 – 7.43 (5H, 5x Ar***H***), 7.40 – 7.36 (m, 3H, 3x Ar***H***), 7.32 – 7.31 (m, 3H, Ar***H***), 5.59 (s, 1H, PhC***H***O_2) 5.10 (d, *J* = 11.8 Hz, 1H, PhC$\boldsymbol{H_a}$$H_b$, ONap), 4.96 (d, *J* = 11.8 Hz, 1H, PhC$H_a$$\boldsymbol{H_b}$, ONap), 4.63 (d, *J* = 9.8 Hz, 1H, ***H-1***), 4.39 (dd, *J* = 10.5, 5.0 Hz, 1H, $\boldsymbol{H\text{-}6_a}$), 3.80 (t, *J* = 10.3 Hz, 1H, $\boldsymbol{H\text{-}6_b}$), 3.77 – 3.66 (m, 2H, ***H-3***, ***H-4***), 3.59 – 3.49 (m, 2H, ***H-2***, ***H-5***), (d, *J* = 2.1 Hz, 1H, 2-O***H***); **^{13}C NMR** (126 MHz, $CDCl_3$): δ 137.20 (Ar***C***CHO_2), 135.59 (Ar***C***CH_2, ONap), 133.27 (Ar***C***C_2, ONap), 133.20 (SPh), 133.07 (Ar***C***C_2, ONap), 131.28 (Ar***C***S, SPh), [129.06, 128.40, 128.31, 128.28, 127.95, 127.68, 126.96, 126.07 (SPh, PhCHO_2, ONap)], 126.01 (ONap), 125.94 (ONap), 101.38 (Ph***C***HO_2), 88.56 (***C-1***), 81.46 (***C-3***), 81.10 (***C-4***), 74.82 (Ar***C***H_2, ONap), 72.38 (***C-2***), 70.79 (***C-5***), 68.66 (***C-6***); **HR-ESI-TOF/Ms(m/z):** $[M+Na]^+$ calcd for $C_{30}H_{28}O_5SNa$, 523.1551; found, 523.15373.

Phenyl 2-*O*-benzyl-3-*O*-(naphthalene-2-ylmethyl)-4,6-O-benzylidene-1-thio-β-D-glucopyranoside (S13)

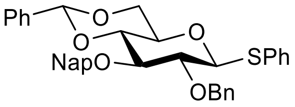

S13

Compound **S12** (5.0 g, 9.9 mmol, 1,0 eq) was dissolved in anh. DMF (150 mL). NaH (1.0 g, 60% wt, 25 mmol, 2.5 eq) added at 0°C. BnBr (1.5 mL, 12 mmol, 1.2 eq) was added. The mixture was stirred at 0°C R.T. for 63 hrs. The reaction was quenched with aq. NH_4Cl (90 mL) and concentrated *in vacuo*. The residue was resuspended in DCM (200 mL). The solution washed with water (100 mL) and brine (100 mL), respectively. The organic layer dried with Na_2SO_4, filtered and evaporated *in vacuo*. The residue was recrystallized from EtOH, yielding monosaccharide **S13** as white crystals (5.4 g, 9.1 mmol, 92%).

TLC: (EtOAc: Hep, 25:75, v/v): R_f = 0.55; **^{1}H NMR** (500 MHz, $CDCl_3$): δ 7.82 – 7.78 (m, 1H, Ar***H***, ONap), 7.77 – 7.72 (m, 2H, 2x Ar***H***, ONap), 7.70 – 7.65 (m, 1H, Ar***H***, ONap), 7.56 – 7.51 (m, 2H, 2x Ar***H***, SPh), 7.51 – 7.41 (m, 5H, 5x Ar***H***, 3x Ar***H***, ONap; 2x Ar***H***, PhCHO_2), 7.41 – 7.28 (m, 11H, 11x Ar***H***, 3x Ar***H***, SPh; 3x Ar***H***, PhCHO_2, 5x Ar***H***, OBn), 5.61 (s, 1H, PhC***H***O_2), 5.08 (d, *J* = 11.4 Hz, 1H, ArC$\boldsymbol{H_a}$$H_b$, ONap), 4.94 (d, *J* = 11.4 Hz,

1H, ArCH$_a$***H$_b$***, ONap), 4.89 (d, *J* = 10.3 Hz, 1H, PhC***H$_a$***H$_b$, OBn), 4.89 (d, *J* = 10.3 Hz, 1H, PhCH$_a$***H$_b$***, OBn), 4.77 (d, *J* = 9.7 Hz, 1H, ***H-1***), 4.39 (dd, *J* = 10.5, 5.0 Hz, 1H, ***H-6a***), 3.89 (dd, *J* = 9.4, 8.3 Hz, 1H, ***H-3***), 3.82 (t, *J* = 10.3 Hz, 1H, ***H-6b***), 3.74 (t, *J* = 9.4 Hz, 1H, ***H-4***), 3.54 (dd, *J* = 9.8, 8.3 Hz, 1H, ***H-2***), 3.48 (td, *J* = 9.7, 5.0 Hz, 1H, ***H-5***); **^{13}C NMR** (126 MHz, $CDCl_3$): δ 138.03 (Ar***C***CH_2, OBn), 137.27 (Ar***C***CHO_2), 135.75 (Ar***C***CH_2, ONap), 133.29 (Ar***C***S, SPh), 133.14 (Ar***C***C_2, ONap), 133.04 (Ar***C***C_2, ONap), 132.34 (SPh), [129.04, 128.42, 128.30, 128.20, 128.14, 127.96, 127.88, 127.67 (SPh; ONap; OBn; $PhCHO_2$)], 126.87 (ONap), 126.22 (ONap), 126.06 ($PhCHO_2$), 126.01 (ONap), 125.88 (ONap), 101.25 (Ph***C***HO_2), 88.35 (***C-1***), 82.94 (***C-3***), 81.47 (***C-4***), 80.56 (***C-2***), 75.92 (Ph***C***H_2, OBn), 75.34 (Ar***C***H_2, ONap), 70.28 (***C-5***), 68.73 (***C-6***); **HR-ESI-TOF/Ms(m/z):** $[M+Na]^+$ calcd for $C_{37}H_{34}O_5SNa$, 613.18871; found, 613.18863.

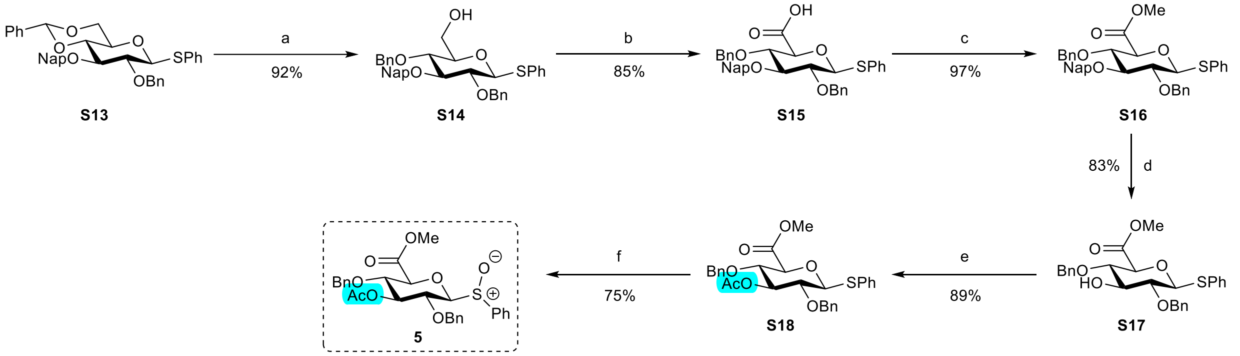

Scheme S6: Monosaccharide **5, S14** – **S18** synthesis. (a) TES, $PhBCl_2$, DCM; (b) TEMPO, BAIB, DCM, H_2O; (c) MeI, K_2CO_3, DMF; (d) DDQ, DCM, H_2O; (e) Ac_2O, pyridine; (f) mCPBA, DCM.

Phenyl 2,4-di-*O*-benzyl-3-*O*-(naphthalene-2-ylmethyl)-1-thio-β-D-glucopyranoside (S14)

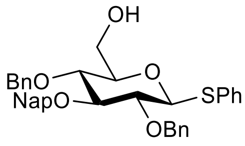

Thioglycoside **S13** (2.4 g, 4.1 mmol, 1.0 eq) was dissolved in anh. DCM (40 mL). The solution was cooled down to 0°C. M.S. (4Å) were added, after which the solution was stirred for 90 min. The solution was cooled down to -78°C. TES (0.95 mL, 6.0 mmol, 1.5 eq) and $PhBCl_2$ (0.95 mL, 7.3 mmol, 1.8 eq) were added. The solution was stirred at -78°C for 45 min. It was then quenched with MeOH:TEA (6.0 mL, 1:1 v/v), warmed up to R.T. and filtered. The filtrate was diluted with DCM (20 mL) and washed with aq. $NaHCO_3$ (sat.) (30 mL). The organic layer was dried with $MgSO_4$, filtered and evaporated *in vacuo*. The residue was purified using silica-flash column chromatography (5 - 40% EtOAc in Hept), yielding monosacccharide **S14** as a white foam (2.2 g, 3.7 mmol, 92%).

TLC: (EtOAc:Hept, 50:50 v/v): R_f = 0.49; **^{1}H NMR** (500 MHz, $CDCl_3$): δ 7.83 – 7.69 (m, 4H, 4x Ar***H***, ONap), 7.53 – 7.50 (m, 2H, 2x Ar***H***, SPh), 7.47 – 7.44 (m, 2H, 2x Ar***H***, ONap), 7.41 (dd, *J* = 8.4, 1.7 Hz, 1H, Ar***H***, ONap), 7.39 – 7.35 (m, 2H, 2x Ar***H***, 2-OBn), 7.33 – 7.23 (m, 11H, 3x Ar***H***, SPh; 8x Ar***H***, OBn), 5.06 (d, *J* = 11.2 Hz, 1H, ArC***H$_a$***H$_b$, ONap), 5.01 (d, *J* = 11.2 Hz, 1H, ArCH$_a$***H$_b$***, ONap), 4.94 (d, *J* = 10.3 Hz, 1H, PhC***H$_a$***H$_b$, 2-OBn), 4.87 (d, *J* = 11.0 Hz, 1H, PhC***H$_a$***H$_b$, 4-OBn), 4.78 (d, *J* = 10.3 Hz, 1H, PhCH$_a$***H$_b$***, 2-OBn),

4.74 (d, J = 9.8 Hz, 1H, ***H-1***), 4.68 (d, J = 11.0 Hz, 1H, PhCH$_a$***H$_b$***, 4-OBn), 3.89 (ddd, J = 12.0, 6.2, 2.7 Hz, 1H, ***H-6$_a$***), 3.78 (t, J = 9.0 Hz, 1H, ***H-3***), 3.71 (ddd, J = 12.1, 7.3, 4.9 Hz, 1H, ***H-6$_b$***), 3.61 (t, J = 9.4 Hz, 1H, ***H-4***), 3.52 (dd, J = 9.8, 8.8 Hz, 1H, ***H-2***), 3.41 (ddd, J = 9.8, 4.8, 2.7 Hz, 1H, ***H-5***), 1.98 (t, J = 6.8 Hz, 1H, 6-O***H***); **^{13}C NMR** (126 MHz, $CDCl_3$; solvent peak ref'd to 77.16): δ 138.06 (Ar***C***CH_2, 2-OBn), 137.99 (Ar***C***CH_2, 4-OBn), 135.93 (Ar***C***CH_2, ONap), 133.66 (Ar***C***S), 133.45 (Ar***C***C_2, ONap), 133.11 (Ar***C***C_2, ONap), 131.95 (SPh), [129.19, 128.64, 128.57, 128.31, 128.11, 128.07, 128.06, 128.02, 127.82, 127.80 (SPh; OBn; ONap)], [126.61, 126.22, 126.03, 125.95 (ONap)], 87.71 (***C-1***), 86.67 (***C-3***), 81.28 (***C-2***), 79.49 (***C-5***), 77.74 (***C-4***), 75.97 (Ar***C***H_2, ONap, 75.68 (Ph***C***H_2, 2-OBn), 75.25 (Ph***C***H_2, 4-OBn), 62.25 (***C-6***); **HR-ESI-TOF/MS (m/z):** $[M+Na]^+$ calcd for $C_{37}H_{36}O_5SNa$, 615.21811; found, 615.21556.

Phenyl 2,4-di-*O*-benzyl-3-*O*-(naphthalene-2-ylmethyl)-1-thio-β-D-glucopyranosiduronic acid (S15)

Thioglycoside **S14** (2.15 g, 3.63 mmol, 1.0 eq) was dissolved in DCM:H_2O (40 mL, 9:1 v/v). BAIB (3.63 g, 11.3 mmol, 3.0 eq) and TEMPO (283 mg, 1.81 mmol, 0.50 eq) were added, after which the solution was stirred at R.T. for 2 hrs. The solution was diluted with DCM (40 mL), quenched with 10% aq. $Na_2S_2O_3$ (60 mL) and stirred for an additional 10 min. The mixture was acidified to pH <3 with citric acid and extracted with EtOAc (3x 60 mL). The combined organic layers were dried with $MgSO_4$, filtered and evaporated *in vacuo*. The residue was purified using silica-flash column chromatography (40% EtOAc in Hept + 2% AcOH), yielding glucuronic acid **S15** as a white solid (1.86 g, 3.07 mmol, 85%).

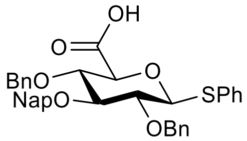

S15

TLC: (EtOAc:Hept:AcOH, 50:48:2 v/v): R_f = 0.31; **^{1}H NMR** (500 MHz, $CDCl_3$): δ 7.83 – 7.72 (m, 3H, 3x ArH, ONap), 7.68 (bs, 1H, Ar***H***, ONap), 7.58 – 7.54 (m, 2H, 2x Ar***H***, SPh), 7.49 – 7.44 (m, 2H, 2x Ar***H***, ONap), 7.38 (dd, J = 8.4, 1.7 Hz, 1H, Ar***H***, ONap), 7.37 – 7.34 (m, 2H, 2x Ar***H***, 2-OBn), 7.33 – 7.26 (m, 6H, 3x Ar***H***, SPh; 3x Ar***H***, OBn), 7.24 – 7.18 (m, 5H, 5x Ar***H***, OBn), 4.99 (d, J = 11.2 Hz, 1H, ArC***H$_a$***H$_b$, ONap), 4.96 (d, J = 11.2 Hz, 1H, ArCH$_a$***H$_b$***, ONap), 4.92 (d, J = 10.4 Hz, 1H, PhC***H$_a$***H$_b$, 2-OBn), 4.80 – 4.73 (m, 3H, ***H-1***, PhCH$_a$***H$_b$***, 2-OBn; PhC***H$_a$***H$_b$, 4-OBn), 4.66 (d, J = 10.8 Hz, 1H, PhCH$_a$***H$_b$***, 4-OBn), 3.99 (d, J = 9.2 Hz, 1H, ***H-5***), 3.86 (t, J = 9.0 Hz, 1H, ***H-4***), 3.78 (t, J = 8.5 Hz, 1H, ***H-3***), 3.57 (dd, J = 9.7, 8.3 Hz, 1H, ***H-2***); **^{13}C NMR** (126 MHz, $CDCl_3$; solvent peak ref'd to 77.16): δ 172.92 (***C-6***), 137.88 (Ar***C***CH_2, 2-OBn), 137.40 (Ar***C***CH_2, 4-OBn), 135.56 (Ar***C***CH_2, ONap), 133.42 (Ar***C***C_2, ONap), [133.15, 133.13 (Ar***C***C_2, ONap; Ar***C***S, SPh)], 132.51 (SPh), [129.25, 128.62, 128.59, 128.57, 128.37, 128.32, 128.28, 128.24, 128.18, 128.14, 128.10, 128.07, 127.83 (SPh; OBn; ONap)], [126.72, 126.28, 126.11, 125.92 (ONap)], 88.31 (***C-1***), 85.47 (***C-3***), 80.42 (***C-2***), 78.77 (***C-4***), 77.53 (***C-5***), 75.92 (Ar***C***H_2, ONap), 75.58 (Ph***C***H_2, 2-OBn), 75.20 (Ph***C***H_2, 4-OBn); **HR-ESI-TOF/MS (m/z):** $[M+Na]^+$ calcd for $C_{37}H_{34}O_6SNa$, 629.19738; found, 629.19465.

Methyl (phenyl 2,4-di-*O*-benzyl-3-*O*-(naphthalene-2-ylmethyl)-1-thio-β-D-glucopyranosyl uronate) (S16)

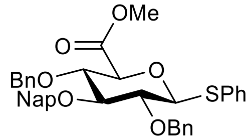

S16

Thioglycoside **S15** (1.6 g, 2.6 mmol, 1.0 eq) was dissolved in anh. DMF (20 mL). MeI (0.33 mL, 5.3 mmol, 2.0 eq) and K_2CO_3 (0.74 g, 5.4 mmol, 2.0 eq) were added, after which the solution was stirred at R.T. for 18 hrs. The reaction was quenched with MeOH (2.0 mL). The solution was diluted with EtOAc (100 mL) and washed with H_2O (100 mL) and brine (100 mL), respectively. The combined organic layers were dried with $MgSO_4$, filtered and evaporated *in vacuo*. The residue was purified using silica-flash column chromatography (30% EtOAc in Hept), yielding monosaccharide **S16** as an off-white solid (1.59 g, 2.6 mmol, 97%).

TLC: (EtOAc:Hept, 50:50 v/v): R_f = 0.62; **^{1}H NMR** (500 MHz, $CDCl_3$): δ 7.81 (dd, *J* = 6.1, 3.4 Hz, 1H, Ar***H***, ONap), 7.77 (d, *J* = 8.5 Hz, 1H, Ar***H***, ONap), 7.73 (dt, *J* = 7.0, 3.4 Hz, 1H, Ar***H***, ONap), 7.69 (bs, 1H, Ar***H***, ONap), 7.57 – 7.53 (m, 2H, 2x Ar***H***, SPh), 7.48 – 7.44 (m, 2H, 2x Ar***H***, ONap), 7.39 (dd, *J* = 8.4, 1.7 Hz, 1H, Ar***H***, ONap), 7.37 – 7.34 (m, 2H, 2x Ar***H***, OBn), 7.33 – 7.19 (m, 11H, 3x Ar***H***, SPh; 8x Ar***H***, OBn), 5.02 (d, *J* = 11.2 Hz, 1H, ArC$\boldsymbol{H_a}H_b$, ONap), 4.99 (d, *J* = 11.2 Hz, 1H, ArC$H_a\boldsymbol{H_b}$, ONap), 4.91 (d, *J* = 10.3 Hz, 1H, PhC$\boldsymbol{H_a}H_b$, 2-OBn), 4.80 (d, *J* = 10.9 Hz, 1H, PhC$\boldsymbol{H_a}H_b$, 4-OBn), 4.75 (d, *J* = 10.3 Hz, 1H, PhC$H_a\boldsymbol{H_b}$, 2-OBn), 4.70 (d, *J* = 9.8 Hz, 1H, ***H-1***), 4.63 (d, *J* = 10.9 Hz, 1H, PhC$H_a\boldsymbol{H_b}$, 4-OBn), 3.94 (d, *J* = 9.6 Hz, 1H, ***H-5***), 3.87 (t, *J* = 9.4 Hz, 1H, ***H-4***), 3.77 (t, *J* = 8.9 Hz, 1H, ***H-3***), 3.74 (s, 3H, C$\boldsymbol{H_3}$, OMe), 3.56 (dd, *J* = 9.7, 8.7 Hz, 1H, ***H-2***); **^{13}C NMR** (126 MHz, $CDCl_3$): δ 168.70 (***C-6***), 137.86 (Ar***C***CH_2, 2-OBn), 137.71 (Ar***C***CH_2, 4-OBn), 135.60 (Ar***C***CH_2, ONap), 133.30 (Ar***C***C_2, ONap), 133.21 (Ar***C***S, SPh), 133.00 (Ar***C***C_2, ONap), 132.21 (SPh), [129.05, 128.45, 128.43, 128.21, 128.17, 127.94, 127.90, 127.83, 127.70 (SPh; OBn; ONap)], [126.55, 126.12, 125.95, 125.82 (ONap)], 88.38 (***C-1***), 85.85 (***C-3***), 80.35 (***C-2***), 79.29 (***C-4***), 78.01 (***C-5***), 75.92 (Ar***C***H2, ONap), 75.53 (Ph***C***H_2, 2-OBn), 75.13 (Ph***C***H_2, 4-OBn), 52.53 (***C***H_3, OMe); **HR-ESI-TOF/MS (m/z):** $[M+Na]^+$ calcd for $C_{38}H_{36}O_6SNa$, 643.21303; found, 643.21007.

Methyl (phenyl 2,4-di-*O*-benzyl-1-thio-β-D-glucopyranosyl uronate) (S17)

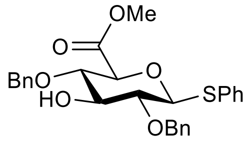

S17

Thioglycoside **S16** (1.55 g, 2.50 mmol, 1.0 eq) was dissolved in DCM:H_2O (40 mL, 9:1 v/v). DDQ (859 mg, 3.78 mmol, 1.5 eq) was added. The solution was vigorously stirred under exclusion of light for 3 hrs. The mixture was diluted with DCM (40 mL), quenched with DDQ mixture (60 mL) and stirred for an additional 10 min. The organic layer was extracted and subsequently washed with DDQ mixture (60 mL) and aq. $NaHCO_3$ (sat.) (60 mL), respectively. The combined organic layers were dried with $MgSO_4$, filtered and evaporated *in vacuo*. The residue was purified using silica-flash column chromatography (0 – 10% EtOAc in Toluene), yielding monosaccharide **S17** as a colourless oil (1.11 g, 2.30 mmol, 92%).

TLC: (EtOAc:Tol, 10:90 v/v): R_f = 0.36; **^{1}H NMR** (500 MHz, $CDCl_3$): δ 7.56 – 7.53 (m, 2H, 2x Ar***H***, SPh), 7.40 – 7.26 (m, 13H, 3x Ar***H***, SPh; 10x Ar***H***, OBn), 4.95 (d, *J* = 11.0 Hz, 1H, PhC***Ha***Hb, 2-OBn), 4.74 (d, *J* = 11.3 Hz, 1H, PhC***Ha***Hb, 4-OBn), 4.70 – 4.62 (m, 3H, ***H-1***, PhCHa***Hb***, 2-OBn; PhCHa***Hb***, 4-OBn), 3.90 (d, *J* = 9.4 Hz, 1H, ***H-5***), 3.80 – 3.70 (m, 5H, ***H-3***, ***H-4***), 3.39 (dd, *J* = 9.8, 8.4 Hz, 1H, ***H-2***), 2.44 – 2.39 (m, 1H, 3-O***H***); **^{13}C NMR** (126 MHz, $CDCl_3$): δ 168.72 (***C-6***), 137.91 (Ar***C***CH_2, OBn), 137.88 (Ar***C***CH_2, OBn), 133.18 (Ar***C***S, SPh), 132.09 (SPh), [129.08, 128.65, 128.52, 128.23, 128.16, 128.00, 127.85 (SPh; OBn)], 87.94 (***C-1***), 80.06 (***C-2***), 78.85 (***C-4***), 78.01 (***C-3***), 77.78 (***C-5***), 75.28 (Ph***C***H_2, 2-OBn), 74.77 (Ph***C***H_2, 4-OBn), 52.57 (***C***H_3, OMe); **HR-ESI-TOF/MS (m/z):** $[M+Na]^+$ calcd for $C_{27}H_{28}O_6SNa$, 503.15043; found, 503.14937.

Methyl (phenyl 3-*O*-acetyl-2,4-di-*O*-benzyl-1-thio-β-D-glucopyranosyl uronate) (S18)

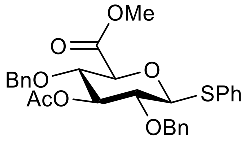

S18

Thioglycoside **S17** (0.23 g, 0.47 mmol, 1.0 eq) was dissolved in pyridine (4.0 mL). Ac_2O (2.0 mL, 21 mmol, 45 eq) was added, after which the solution was stirred at R.T. for 16 hrs. The solution was evaporated *in vacuo*. The residue was dissolved in EtOAc (20 mL). The solution was washed with H_2O (20 mL), aq. $NaHCO_3$ (sat.) (20 mL) and brine (20 mL), respectively. The organic layer was dried with $MgSO_4$, filtered and evaporated *in vacuo*. The residue was purified using silica-flash column chromatography (0 – 5% EtOAc in Tol), yielding monosaccharide **S18** as a colourless oil (0.22 g, 0.42 mmol, 89%).

TLC: (EtOAc:Tol, 10:90 v/v): R_f = 0.49; **^{1}H NMR** (500 MHz, $CDCl_3$): δ 7.57 – 7.52 (m, 2H, 2x Ar***H***, SPh), 7.38 – 7.18 (m, 13H, 3x Ar***H***, SPh; 10x Ar***H***, OBn), 5.30 (t, *J* = 9.2 Hz, 1H, ***H-3***), 4.85 (d, *J* = 10.9 Hz, 1H, PhC***Ha***Hb, 2-OBn), 4.74 (d, *J* = 9.7 Hz, 1H, ***H-1***), 4.59 – 4.52 (m, 2H, PhCHa***Hb***, 2-OBn; PhC***Ha***Hb, 4-OBn), 4.49 (d, *J* = 11.3 Hz, 1H, PhCHa***Hb***, 4-OBn), 3.98 (d, *J* = 9.5 Hz, 1H, ***H-5***), 3.84 (t, *J* = 9.5 Hz, 1H, ***H-4***), 3.75 (s, 3H, C***H_3***, OMe), 3.49 (t, *J* = 9.4 Hz, 1H, ***H-2***), 1.83 (s, 3H, C***H_3***, OAc); **^{13}C NMR** (126 MHz, $CDCl_3$): δ 169.69 (***C***=O, OAc), 168.50 (***C-6***), 137.57 (Ar***C***CH_2, OBn), 137.46 (Ar***C***CH_2, OBn), 132.93 (Ar***C***S, SPh), 132.29 (SPh), [129.12, 128.46, 128.12, 127.99, 127.95, 127.93, 127.89 (SPh; OBn)], 88.29 (***C-1***), 78.36 (***C-2***), 77.75 (***C-5***), 77.60 (***C-4***), 76.17 (***C-3***), 74.85 (Ph***C***H_2, 2-OBn), 74.46 (Ph***C***H_2, 4-OBn), 52.66 (***C***H_3, OMe), 20.88 (***C***H_3, OAc); **HR-ESI-TOF/MS (m/z):** $[M+Na]^+$ calcd for $C_{29}H_{30}O_7SNa$, 545.16099; found, 545.15977.

Methyl (phenyl 3-*O*-acetyl-2,4-di-*O*-benzyl-1-sulfinyl-β-D-glucopyranosyl uronate) (5)

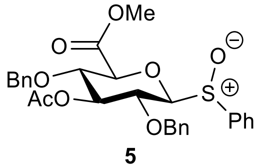

5

Thioglycoside **S18** (161 mg, 308 µmol, 1.0 eq) was dissolved in DCM (10 mL). The solution was cooled down to -78°C and purged with Argon. mCPBA (72.0 mg, 321 µmol, 1.0 eq) was added, after which the solution was stirred at -78°C for 5 hrs. The solution was then stirred at -78°C – R.T. for an additional 16 hrs. The solution was diluted with DCM (10 mL), quenched by the addition of 10% aq.

$Na_2S_2O_3$ (20 mL) and stirred for an additional 10 min. The organic layer was extracted and subsequently washed with aq. $NaHCO_3$ (sat.) (2x 20 mL). The organic layer was dried with $MgSO_4$, filtered and evaporated *in vacuo*. The residue was purified using silica-flash column chromatography (0 – 25% EtOAc in Tol), yielding sulfoxide **5** as a colourless oil (124 mg, 230 µmol, 75%).

Diastereomer A: **TLC:** (EtOAc:Tol, 30:70 v/v): R_f = 0.50; **^{1}H NMR** (500 MHz, $CDCl_3$): δ 7.64 – 7.60 (m, 2H, 2x Ar***H***, SOPh), 7.43 – 7.21 (m, 11H, 3x Ar***H***, SOPh; 8x Ar***H***, OBn), 7.15 – 7.11 (m, 2H, 2x Ar***H***, OBn), 5.38 (t, *J* = 7.2 Hz, 1H, ***H-3***), 4.63 (d, *J* = 11.2 Hz, 1H, PhC***H***$_a$H$_b$, 2-OBn), 4.60 – 4.52 (m, 4H, ***H-1***, PhCH$_a$***H***$_b$, 2-OBn; PhC***H***$_2$, 4-OBn), 4.20 (d, *J* = 9.0 Hz, 1H, ***H-5***), 4.06 (t, *J* = 6.7 Hz, 1H, ***H-2***), 3.92 (dd, *J* = 9.0, 7.5 Hz, 1H, ***H-4***), 3.76 (s, 3H, C***H***$_3$, OMe 1.92 (s, 3H, C***H***$_3$, OAc); **^{13}C NMR** (126 MHz, $CDCl_3$): δ 169.74 (***C***=O, OAc), 168.54 (***C-6***), 140.52 (Ar***C***SO), 137.24 (Ar***C***CH$_2$, OBn), 137.22 (Ar***C***CH$_2$, OBn), 131.18 (SOPh), [128.94, 128.47, 128.30, 128.03, 127.93, 127.75, 127.63 (SOPh; OBn)], 124.96 (SOPh), 95.67 (***C-1***), 76.77 (***C-4***), 76.69 (***C-5***), 74.74 (***C-3***), 73.97 (Ph***C***H$_2$, 4-OBn), 73.71 (***C-2***), 73.29 (Ph***C***H$_2$, 2-OBn), 52.67 (***C***H$_3$, OMe), 20.91 (***C***H$_3$, OAc).

Diastereomer B: **TLC:** (EtOAc:Tol, 30:70 v/v): R_f = 0.46; **^{1}H NMR** (500 MHz, $CDCl_3$): δ 7.65 – 7.60 (m, 2H, 2x Ar***H***, SOPh), 7.53 – 7.48 (m, 3H, 3x Ar***H***, SOPh), 7.38 – 7.24 (m, 8H, 8x Ar***H***, OBn), 7.20 – 7.17 (m, 2H, 2x Ar***H***, OBn), 5.37 (t, *J* = 9.1 Hz, 1H, ***H-3***), 4.94 (d, *J* = 10.9 Hz, 1H, PhC***H***$_a$H$_b$, 2-OBn), 4.73 (d, *J* = 11.0 Hz, 1H, PhCH$_a$***H***$_b$, 2-OBn), 4.57 (d, *J* = 11.3 Hz, 1H, PhC***H***$_a$H$_b$, 4-OBn), 4.48 (d, *J* = 11.3 Hz, 1H, PhCH$_a$***H***$_b$, 4-OBn), 4.10 (t, *J* = 9.4 Hz, 1H, ***H-2***), 4.00 (d, *J* = 9.6 Hz, 1H, ***H-1***), 3.86 (t, *J* = 9.3 Hz, 1H, ***H-4***), 3.72 (d, *J* = 9.5 Hz, 1H, ***H-5***), 3.62 (s, 3H, C***H***$_3$, OMe), 1.88 (s, 3H, C***H***$_3$, OAc); **^{13}C NMR** (126 MHz, $CDCl_3$): δ 169.81 (***C***=O, OAc), 167.39 (***C-6***), 138.73 (Ar***C***SO), 137.26 (Ar***C***CH$_2$, 4-OBn), 137.09 (Ar***C***CH$_2$, 2-OBn), 131.45 (SOPh), [128.86, 128.60, 128.47, 128.30, 128.21, 128.01, 127.99 (SOPh; OBn)], 125.53 (SOPh), 92.88 (***C-1***), 77.98 (***C-5***), 76.96 (***C-4***), 76.28 (***C-3***), 75.50 (Ph***C***H$_2$, 2-OBn), 74.74 (***C-2***), 74.64 (Ph***C***H$_2$, 4-OBn), 52.48 (***C***H$_3$, OMe), 20.91 (***C***H$_3$, OAc).
HR-ESI-TOF/MS (m/z): $[M+Na]^+$ calcd for $C_{29}H_{30}O_8SNa$, 561.15591; found, 561.15380.

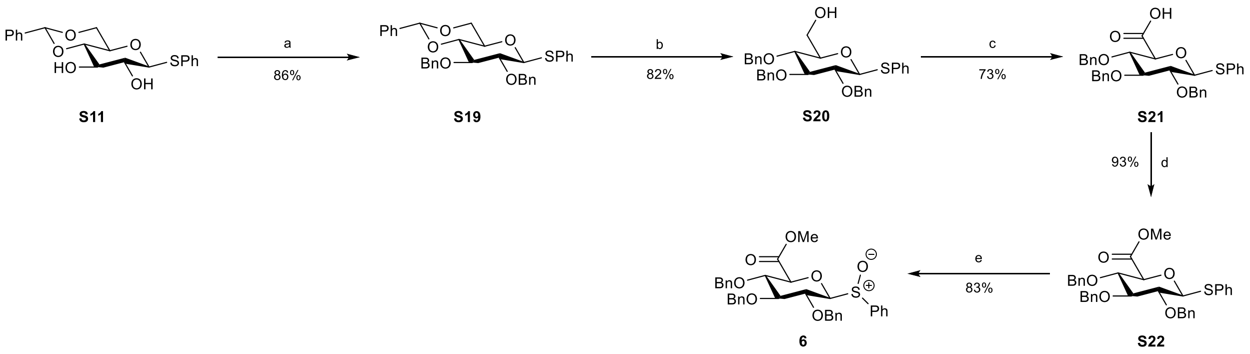

Scheme S7: Monosaccharide **6**, and **S19** – **S22** synthesis. (a) BnBr, NaH, DMF; (b) $PhBCl_2$, TES, DCM; (c) TEMPO, BAIB, DCM, H_2O; (d) MeI, K_2CO_3, DMF; (e) mCPBA, DCM.

Phenyl 4,6-*O*-benzylidene-1-thio-β-D-glucopyranoside (S19)

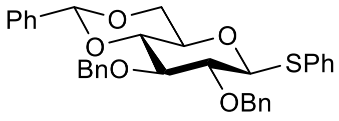

S19

Thioglycoside **S11** (4.8g, 13 mmol, 1.0 eq) was dissolved in anh. DMF (0.10 L). The solution was cooled down to 0°C. NaH (1.6 g, 60% Wt, 40 mmol, 3.1 eq) and BnBr (4.2 mL, 35 mmol, 2.7 eq) added. The mixture was stirred at 0°C – R.T. for 17 hrs. The mixture was diluted with water and extracted with DCM. The organic layer was washed with brine, dried with $MgSO_4$ and evaporated *in vacuo*. The residue was recrystallized from EtOAc/Hept, yielding monosaccharide **S19** (6.3 g, 12 mmol, 86%) as a white solid.

TLC: (50% EtOAc/Hep, 50:50, v/v): R_f = 0.75; **^{1}H NMR** (500 MHz, $CDCl_3$): δ 7.56 – 7.51 (m, 2H, 2x Ar***H***, SPh), 7.51 – 7.46 (m, 2H, 2x Ar***H***, $PhCHO_2$), 7.42 – 7.26 (m, 16H, 3x Ar***H***, SPh; 3x Ar***H***, $PhCHO_2$; 10x Ar***H***, OBn), 5.59 (s, 1H, $PhC\boldsymbol{H}O_2$), 4.94 (d, *J* = 11.2 Hz, 1H, $PhC\boldsymbol{H_a}H_b$, 3-OBn), 4.86 (d, *J* = 10.3 Hz, 1H, $PhC\boldsymbol{H_a}H_b$, 2-OBn), 4.81 (d, *J* = 10.3 Hz, 1H, $PhCH_a\boldsymbol{H_b}$, 2-OBn), 4.80 – 4.74 (m, 2H, ***H-1***, $PhCH_a\boldsymbol{H_b}$, 3-OBn), 4.38 (dd, *J* = 10.5, 5.0 Hz, 1H, ***H-6a***), 3.86 – 3.78 (m, 2H, ***H-3***, ***H-6b***), 3.70 (t, *J* = 9.4 Hz, 1H, ***H-4***), 3.54 – 3.44 (m, 2H, ***H-2***, ***H-5***); **^{13}C NMR** (126 MHz, $CDCl_3$; solvent peak ref'd to 77.16): δ 138.42 (Ar***C***CH_2, 3-OBn), 138.16 (Ar***C***CH_2, 2-OBn), 137.39 (Ar***C***CHO_2), 133.24 (Ar***C***S), 132.49 (SPh), [129.15, 129.12, 128.54, 128.53, 128.40, 128.36, 128.02, 128.01, 127.91, (SPh, OBn, $PhCHO_2$)], 126.13 ($PhCHO_2$), 101.28 (Ph***C***HO_2), 88.43 (***C-1***), 83.15 (***C-3***), 81.61 (***C-4***), 80.60 (***C-2***), 76.04 (Ph***C***H_2, 2-OBn), 75.46 (Ph***C***H_2, 3-OBn), 70.39 (***C-5***), 68.85 (***C-6***); **HR-ESI-TOF/Ms(m/z):** $[M+Na]^+$ calcd for $C_{33}H_{32}O_5SNa^+$ 563.18591; found 563.18681

Phenyl 2,3,4-O-benzyl-1-thio-β-D-glucopyranoside (S20)

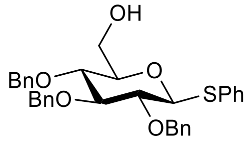

S20

Thioglycoside **S19** (3.0 g, 5.2 mmol, 1.0 eq) was dissolved in anh. DCM (21 mL). The solution cooled down to -78°C and molecular sieves (4Å) were added. The solution was stirred for 30 min. TES (1.3 mL, 7.8 mmol, 1.5 eq) and $PhBCl_2$ (1.2 mL, 8.2 mmol, 1.6 eq) were added. The reaction allowed to warm up to R.T and quenched with MeOH/TEA (6.0 mL, 1:1 v/v). The mixture was diluted with DCM and washed with $NaHCO_3$ (sat.) (2x 80 mL). The organic layer was dried with $MgSO_4$, filtered and evaporated *in vacuo*. The residue was purified using silica-flash column chromatography (10 – 30% EtOAc in Hept), yielding monosaccharide **S20** as a white solid. (2.4 g, 4.5 mmol, 82%).

TLC: (EtOAc:Hept, 50:50 v/v): R_f = 0.65; **^{1}H NMR** (500 MHz, $CDCl_3$): δ 7.53 – 7.49 (m, 2H, 2x Ar***H***, SPh), 7.41 – 7.26 (m, 18H, 3x Ar***H***, SPh; 15x Ar***H***, OBn), 4.93 – 4.89 (m, 2H, $PhC\boldsymbol{H_a}H_b$, 2-OBn; $PhC\boldsymbol{H_a}H_b$, 3-OBn), 4.89 – 4.83 (m, 2H, $PhCH_a\boldsymbol{H_b}$, 3-OBn; $PhC\boldsymbol{H_a}H_b$, 4-OBn), 4.76(d, *J* = 10.2 Hz, 1H, $PhCH_a\boldsymbol{H_b}$, 2-OBn), 4.72 (d, *J* = 9.8 Hz, 1H, ***H-1***), 4.65 (d, *J* = 10.9 Hz, 1H, $PhCH_a\boldsymbol{H_b}$, 2-OBn), 3.87 (ddd, *J* = 12.0, 6.4, 2.7 Hz, 1H, ***H-6a***), 3.76 – 3.66 (m, 2H, ***H-3***, ***H-6b***), 3.57 (t, *J* = 9.4 Hz, 1H, ***H-4***), 3.49 (dd, *J* = 9.8, 8.8 Hz, 1H, ***H-2***), 3.39 (ddd, *J* = 9.7, 4.9, 2.7 Hz, 1H, ***H-5***), 1.89 (t, *J* = 6.9 Hz, 1H, 6-O***H***); **^{13}C NMR** (126 MHz, $CDCl_3$; solvent peak ref'd to 77.16): δ 138.42 (Ar***C***CH_2, OBn), 138.07 (Ar***C***CH_2,

OBn), 137.97 (Ar***C***CH_2, OBn), 133.62 (Ar***C***S, SPh), 132.01 (SPh), [129.20, 128.67, 128.62, 128.59, 128.36, 128.18, 128.06, 127.90, 127.84 (SPh, OBn)], 87.69 (***C-1***), 86.70 (***C-3***), 81.26 (***C-2***), 79.47 (***C-5***), 77.75 (***C-4***), 75.97 (Ph***C***H_2, 3-OBn), 75.69 (Ph***C***H_2, 2-OBn), 75.26 (Ph***C***H_2, 4-OBn), 62.30 (***C-6***); **HR-ESI-TOF/Ms(m/z):** $[M+Na]^+$ calcd for $C_{33}H_{34}O_5SNa$, 565.20246; found, 565.20042.

Phenyl 2,3,4-O-benzyl-1-thio-β-D-glucopyranosiduronic acid (S21)

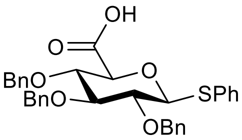

S21

Thioglycoside **S20** (3.2 g, 9.9 mmol, 2.0 eq) was dissolved in DCM:H_2O (30 mL, 9:1 v/v). BAIB (3.2 g, 4.9 mmol, 2.0 eq) and TEMPO (0.20 g, 1.5 mmol, 0.30 eq) were added, after which the solution was stirred at R.T. for 80 min. The reaction was quenched with 10% aq. $Na_2S_2O_3$ (60 mL) and stirred for an additional 10 min. The mixture was acidified to pH <3 with citric acid and extracted with EtOAc (60 mL). The organic layer was dried with $MgSO_4$, filtered and evaporated *in vacuo*. The residue was purified using silica-flash column chromatography (20 – 60% EtOAc in Hept + 2% AcOH), yielding uronic acid **S21** as a white solid (1.9 g, 3.3 mmol, 73%).

TLC: (EtOAc/Hept/AcOH, 60:38:2 v/v): R_f = 0.50; **^{1}H NMR** (500 MHz, $CDCl_3$): δ 7.56 – 7.53 (m, 2H, Ar***H***, SPh), 7.39 – 7.21 (m, 18H, 3x Ar***H***, SPh; 15x Ar***H***, OBn), 4.88 (d, *J* = 10.4 Hz, 1H, PhC***H_a***Hb, 2-OBn), 4.82 (d, *J* = 11.1 Hz, 1H, PhC***Ha***Hb, 3-OBn), 4.79 (d, *J* = 11.1 Hz, 1H, PhCHa***Hb***, 3-OBn), 4.78 – 4.75 (m, 2H, ***H-1***, PhC***Ha***Hb, 4-OBn), 4.72 (d, *J* = 10.4 Hz, 1H, PhCHa***Hb***, 2-OBn), 4.66 (d, *J* = 10.7 Hz, 1H, PhCHa***Hb***, 4-OBn), 4.00 (d, *J* = 8.9 Hz, 1H, ***H-5***), 3.83 (t, *J* = 8.8 Hz, 1H, ***H-4***), 3.73 (t, *J* = 8.3 Hz, 1H, ***H-3***), 3.54 (dd, *J* = 9.7, 8.0 Hz, 1H, ***H-2***); **^{13}C NMR** (126 MHz, $CDCl_3$; solvent peak ref'd to 77.16): δ 171.23 (***C-6***), 138.06 (Ar***C***CH_2, OBn), 137.85 (Ar***C***CH_2, OBn), 137.41 (Ar***C***CH_2, OBn), 133.05 (Ar***C***S, SPh), 132.60 (SPh), [129.29, 128.66, 128.63, 128.62, 128.37, 128.34, 128.23, 128.15, 128.04, 127.98 (SPh, OBn)], 88.04 (***C-1***), 85.09 (***C-3***), 80.34 (***C-2***), 78.64 (***C-4***), 77.29 (***C-5***), 75.74 (Ph***C***H_2, 3-OBn), 75.47 (Ph***C***H_2, 2-OBn), 75.14 (Ph***C***H_2, 4-OBn); **HR-ESI-TOF/Ms(m/z):** $[M+Na]^+$ calcd for $C_{33}H_{32}O_6SNa$, 579.1812; found, 579.1804.

Methyl (phenyl 2,3,4-O-benzyl-1-thio-β-D-glucopyranosyl uronate) (S22)

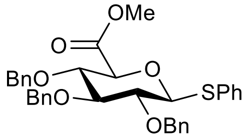

S22

Thioglycoside **S21** (1.8 g, 3.3 mmol, 1.0 eq) was dissolved in anh. DMF (17 mL). K_2CO_3 (1.4 g, 10 mmol, 3.0 eq) and MeI (0.57 mL, 9.7 mmol, 3.0 eq) were added. The mixture was stirred for 30 min, after which it was quenched with MeOH (5.0 mL). The mixture was extracted with DCM (50 mL) and washed with water (2x 50 mL) and brine (2x 50 mL), respectively. The organic layer was dried with $MgSO_4$, filtered and evaporated *in vacuo*. The residue was purified using silica-flash column chromatography (20 – 40 % EtOAc in Hept), yielding monosaccharide **S22** as a white solid (1.8 g, 3.1 mmol, 93%).

TLC: (EtOAc:Hept, 50:50 v/v): R_f = 0.75; **^{1}H NMR** (500 Mz, $CDCl_3$): δ 7.57 – 7.53 (m, 2H, 2x Ar***H***, SPh), 7.40 – 7.20 (m, 18H, 3x Ar***H***, SPh; 15x Ar***H***, OBn), 4.91 – 4.86 (m, 2H,

PhC***H***$_a$H$_b$, 2-OBn; PhC***H***$_a$H$_b$, 3-OBn), 4.84 (d, *J* = 11.0, 1H, PhCH$_a$***H***$_b$, 3-OBn), 4.77 (d, *J* = 10.8 Hz, 1H, PhC***H***$_a$H$_b$, 4-OBn), 4.72 (d, *J* = 10.2 Hz, 1H, PhCH$_a$***H***$_b$, 2-OBn), 4.68 (d, *J* = 9.7 Hz, 1H, ***H-1***), 4.61 (d, *J* = 10.8 Hz, 1H, PhCH$_a$***H***$_b$, 4-OBn), 3.92 (d, *J* = 9.7 Hz, 1H, ***H-5***), 3.84 (t, *J* = 9.4 Hz, 1H, ***H-4***), 3.75 – 3.68 (m, 4H, ***H-3***, C***H***$_3$, OMe), 3.52 (dd, *J* = 9.8, 8.7 Hz, 1H, ***H-2***); **^{13}C NMR** (126 MHz, $CDCl_3$): δ 168.82 (***C-6***), 138.27 (Ar***C***CH_2, OBn), 137.98 (Ar***C***CH_2, OBn), 137.84 (Ar***C***CH_2, OBn), 133.33 (Ar***C***S, SPh), 132.35 (SPh), [129.18, 128.61, 128.58, 128.57, 128.33, 128.11, 128.11, 128.07, 128.05, 127.96 (SPh; OBn)], 88.49 (***C-1***), 86.02 (***C-3***), 80.45 (***C-2***), 79.40 (***C-4***), 78.14 (***C-5***), 76.03 (Ph***C***H_2, 3-OBn), 75.65 (Ph***C***H_2, 2-OBn), 75.25 (Ph***C***H_2, 4-OBn), 52.66 (***C***H_3); **HR-ESI-TOF/Ms(m/z):** $[M+Na]^+$ calcd for $C_{34}H_{24}O_6SNa$, 593.19738; found, 593.19452.

Methyl (phenyl 2,3,4-tri-*O*-benzyl-1-sulfinyl-β-D-glucopyranosyl uronate) (6)

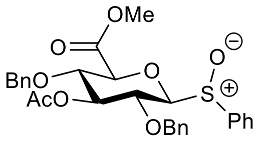
6

Thioglycoside **S22** (305 mg, 534 µmol, 1.0 eq) was dissolved in DCM (15 mL). The solution was cooled down to -78°C and purged with Argon. mCPBA (127 mg, 567 µmol, 1.1 eq) was added, after which the solution was stirred at -78°C for 3 hrs. The solution was then stirred at -78°C – R.T. for an additional 18 hrs. The solution was diluted with DCM (15 mL), quenched by the addition of 10% aq. $Na_2S_2O_3$ (30 mL) and stirred for an additional 10 min. The organic layer was extracted and subsequently washed with aq. $NaHCO_3$ (sat.) (30 mL). The organic layer was dried with $MgSO_4$, filtered and evaporated *in vacuo*. The residue was purified using silica-flash column chromatography (0 – 20% EtOAc in Toluene), yielding sulfoxide **6** as a colourless oil (260 mg, 443 µmol, 83%).

Diastereomer A | TLC: (EtOAc:Tol, 30:70 v/v): R_f = 0.50; **^{1}H NMR** (500 MHz, $CDCl_3$): δ 7.66 – 7.61 (m, 2H, 2x Ar***H***, SOPh), 7.46 – 7.38 (m, 3H, 3x Ar***H***, SOPh), 7.33 – 7.23 (m, 11H, 11x Ar***H***, OBn), 7.22 – 7.19 (m, 2H, 2x Ar***H***, OBn), 7.15 – 7.11 (m, 2H, 2x Ar***H***, OBn), 4.77 – 4.72 (m, 4H, PhC***H***$_2$, 2-OBn; PhC***H***$_2$, 3-OBn), 4.69 (d, *J* = 10.9 Hz, 1H, PhC***H***$_a$H$_b$, 4-OBn), 4.55 (d, *J* = 11.0 Hz, 1H, PhCH$_a$***H***$_b$, 4-OBn), 4.52 (d, *J* = 7.7 Hz, 1H, ***H-1***), 4.12 – 4.07 (m, 1H, ***H-5***), 3.97 – 3.92 (m, 1H, ***H-2***), 3.86 – 3.80 (m, 2H, ***H-3***, ***H-4***), 3.71 (s, 3H, C***H***$_3$, OMe); **^{13}C NMR** (126 MHz, $CDCl_3$): δ 168.62 (***C-6***), 140.37 (Ar***C***SO), 137.64 (Ar***C***CH_2, OBn), 137.48 (Ar***C***CH_2, OBn), 137.45 (Ar***C***CH_2, OBn), 131.23 (SOPh), [128.86, 128.50, 128.45, 128.30, 127.98, 127.94, 127.91, 127.82, 127.73, 127.61 (SOPh; OBn)], 125.49 (SOPh), 95.46 (***C-1***), 84.26 (***C-3***), 78.61 (***C-4***), 77.12 (***C-5***), 75.12 (***C-2***), 74.92 (Ph***C***H_2, 3-OBn), 74.62 (Ph***C***H_2, 4-OBn), 73.68 (Ph***C***H_2, 2-OBn), 52.51 (***C***H_3, OMe).

Diastereomer B: TLC: (EtOAc:Tol, 30:70 v/v): R_f = 0.46; **^{1}H NMR** (500 MHz, $CDCl_3$): δ 7.66 – 7.61 (m, 2H, 2x Ar***H***, SOPh), 7.51 – 7.47 (m, 3H, 3x Ar***H***, SOPh), 7.43 – 7.17 (m, 15H, 15x Ar***H***, OBn), 5.00 (d, *J* = 10.3 Hz, 1H, PhC***H***$_a$H$_b$, 2-OBn), 4.97 – 4.88 (m, 3H, PhCH$_a$***H***$_b$, 2-OBn; PhC***H***$_2$, 3-OBn), 4.74 (d, *J* = 10.9 Hz, 1H, PhC***H***$_a$H$_b$, 4-OBn), 4.61 (d, *J* = 10.8 Hz, 1H, PhCH$_a$***H***$_b$, 4-OBn), 4.12 (t, *J* = 9.4 Hz, 1H, ***H-2***), 3.96 (d, *J* = 9.8 Hz, 1H, ***H-1***), 3.86 (t, *J* = 9.2 Hz, 1H, ***H-4***), 3.79 (t, *J* = 8.9 Hz, 1H, ***H-3***), 3.67 (d, *J* = 9.5 Hz, 1H, ***H-5***), 3.60 (s, 3H, C***H***$_3$, OMe); **^{13}C NMR** (126 MHz, $CDCl_3$): δ 167.64 (***C-6***), 139.08 (Ar***C***SO), 137.99 (Ar***C***CH_2, OBn), 137.47 (Ar***C***CH_2, OBn), 137.37 (Ar***C***CH_2, OBn), 131.29

(SOPh), [128.80, 128.56, 128.53, 128.42, 128.37, 128.16, 128.08, 127.96, 127.86, 127.67 (SOPh; OBn), 125.52 (SOPh), 93.15 (***C-1***), 85.67 (***C-3***), 78.65 (***C-4***), 78.23 (***C-5***), 76.29 (***C-2***), 75.84 (Ph***C***H$_2$, 2-OBn), 75.69 (Ph***C***H$_2$, 3-OBn), 75.11 (Ph***C***H$_2$, 4-OBn), 52.36 (***C***H$_3$, OMe).
HR-ESI-TOF/MS (m/z): $[M+Na]^+$ calcd for $C_{34}H_{34}O_7SNa$, 609.19229; found, 609.18951.

Mannosyl[8] and Rhamnosyl[9] donors **19** and **20** where prepared as previously described by us.

Supporting characterization data

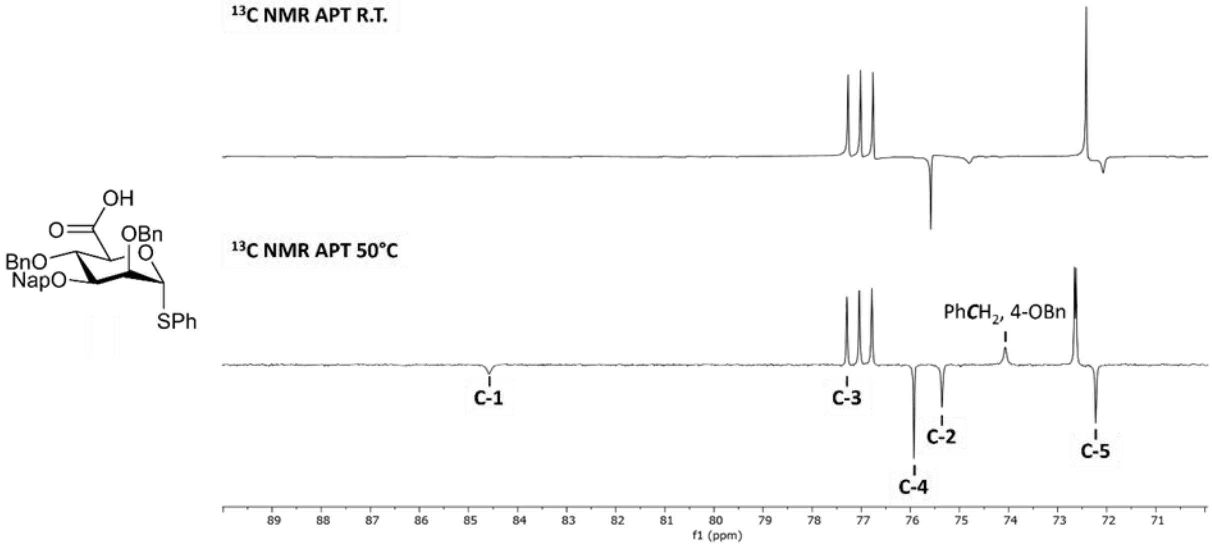

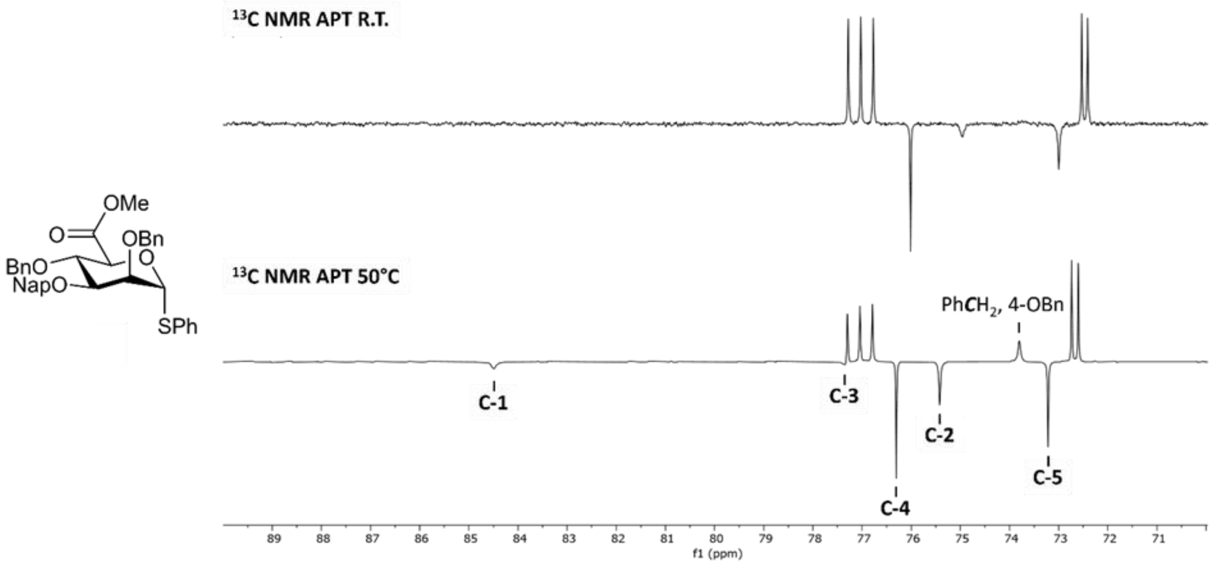

Figure S3: Stacked ^{13}C 1D spectra of C-2,4 benzyl-protected and C-3 naphthyl-protected mannuronic acid derivatives measured at room temperature (top) and 50°C (bottom). Resonances have been assigned for the elevated temperature measurements using COSY, HSQC and HMBC NMR.

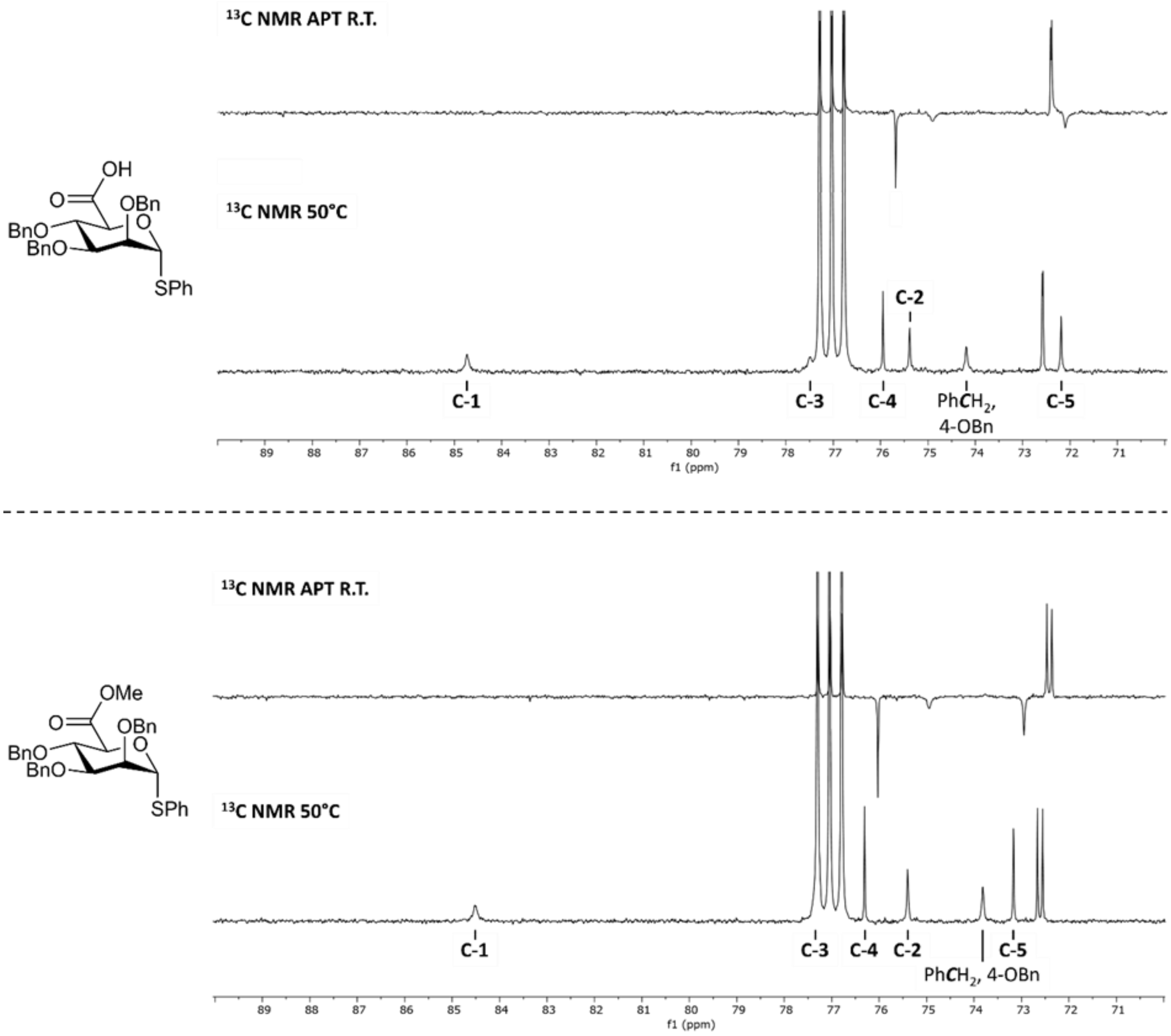

Figure S4: Stacked ^{13}C 1D spectra of C-2,3,4 benzyl-protected mannuronic acid derivatives measured at room temperature (top) and 50°C (bottom). Resonances have been assigned for the elevated temperature measurements using COSY, HSQC and HMBC NMR.

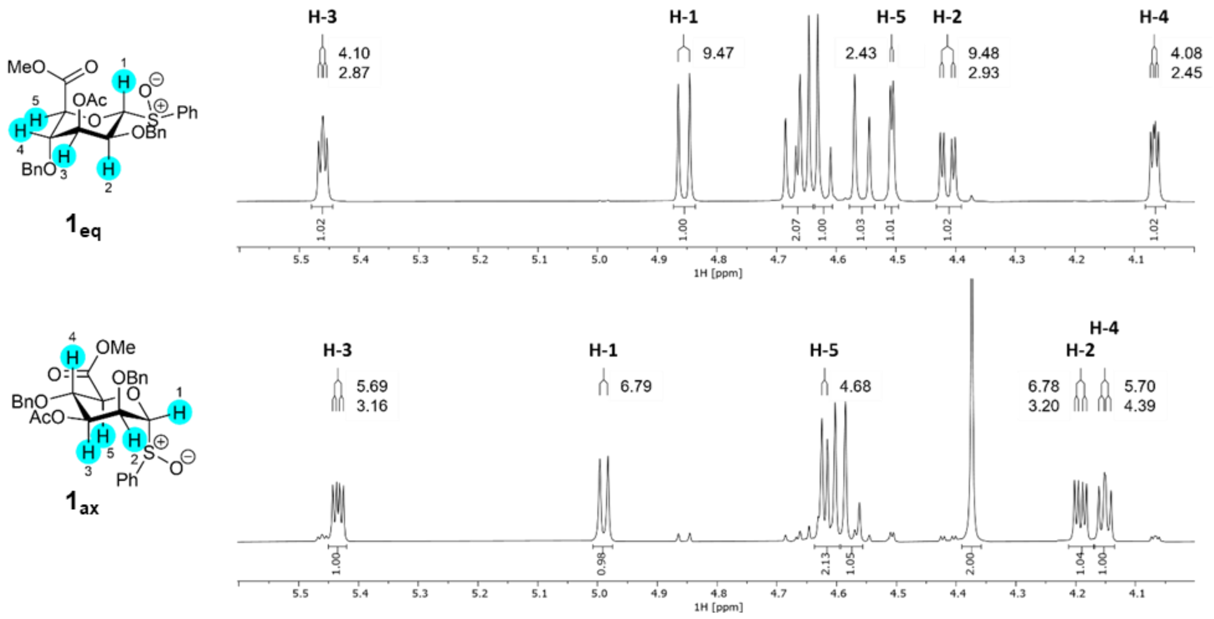

Figure S5: Stacked ^{1}H 1D spectra of two diastereomers of C-3 acetyl-substituted derivative **1**, $^{3}J_{H\text{-}H}$-coupling suggests that $\mathbf{1_{eq}}$ adopts a $^{1}C_4$-chair conformation while $\mathbf{1_{ax}}$ adopts a $^{4}C_1$-chair conformation.

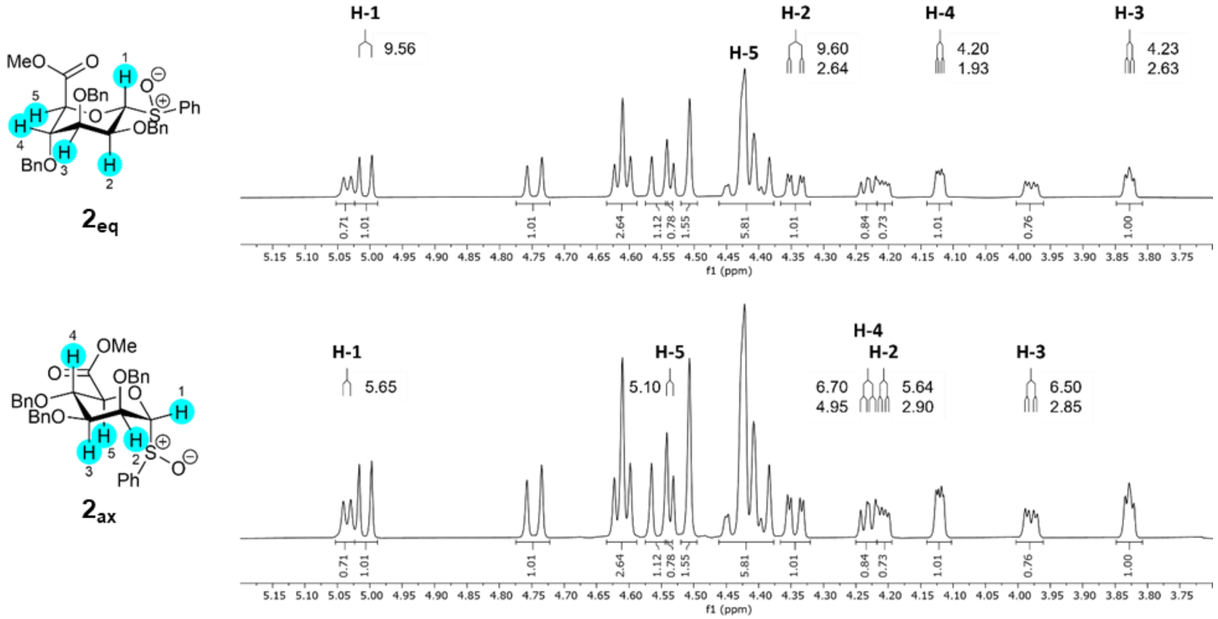

Figure S6: ^{1}H 1D spectrum of two diastereomers of C-3 benzyl-substituted derivative **2**, $^{3}J_{H\text{-}H}$-coupling suggests that $\mathbf{2_{eq}}$ adopts a $^{1}C_4$-chair conformation while $\mathbf{2_{ax}}$ adopts a $^{4}C_1$-chair conformation.

Supporting donor activation data

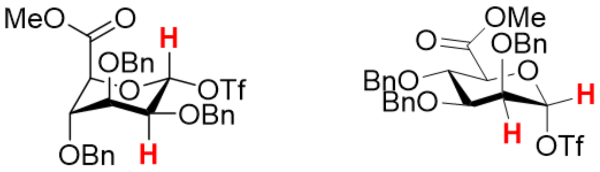

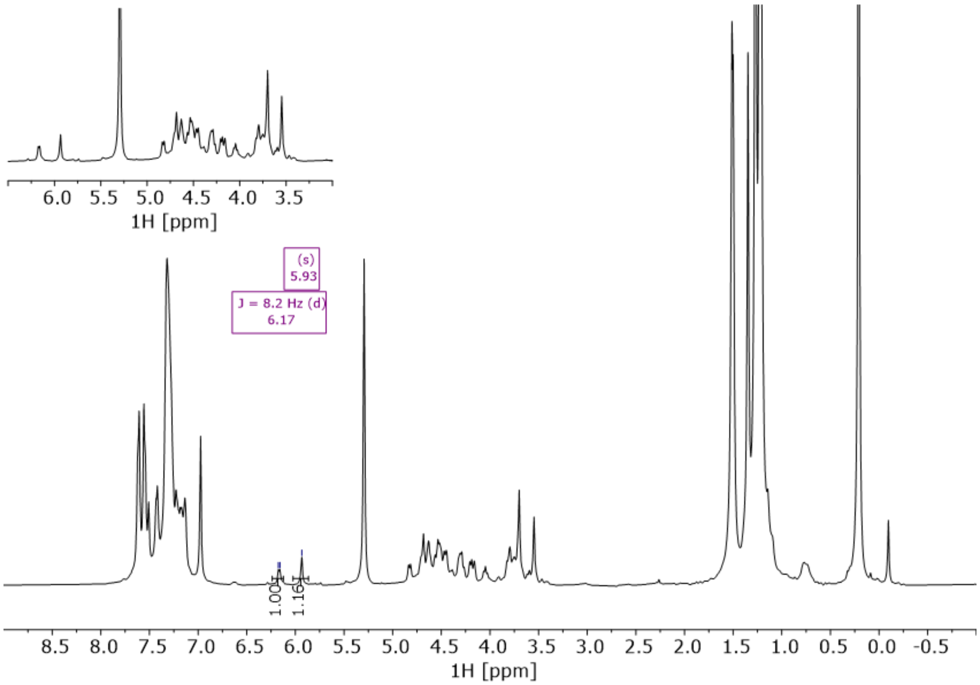

Figure S7: ^{1}H NMR spectrum of activated benzylated mannuronic acid donor.

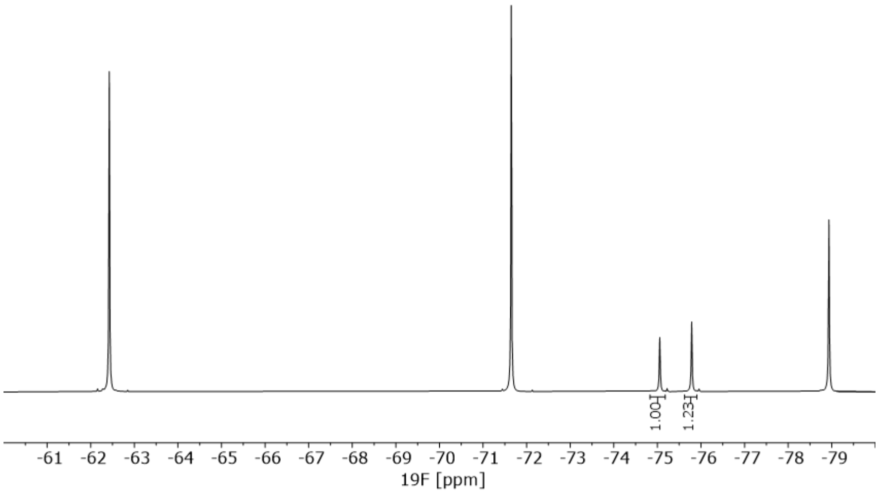

Figure S8: ^{19}F NMR spectrum of activated benzylated mannuronic acid donor.

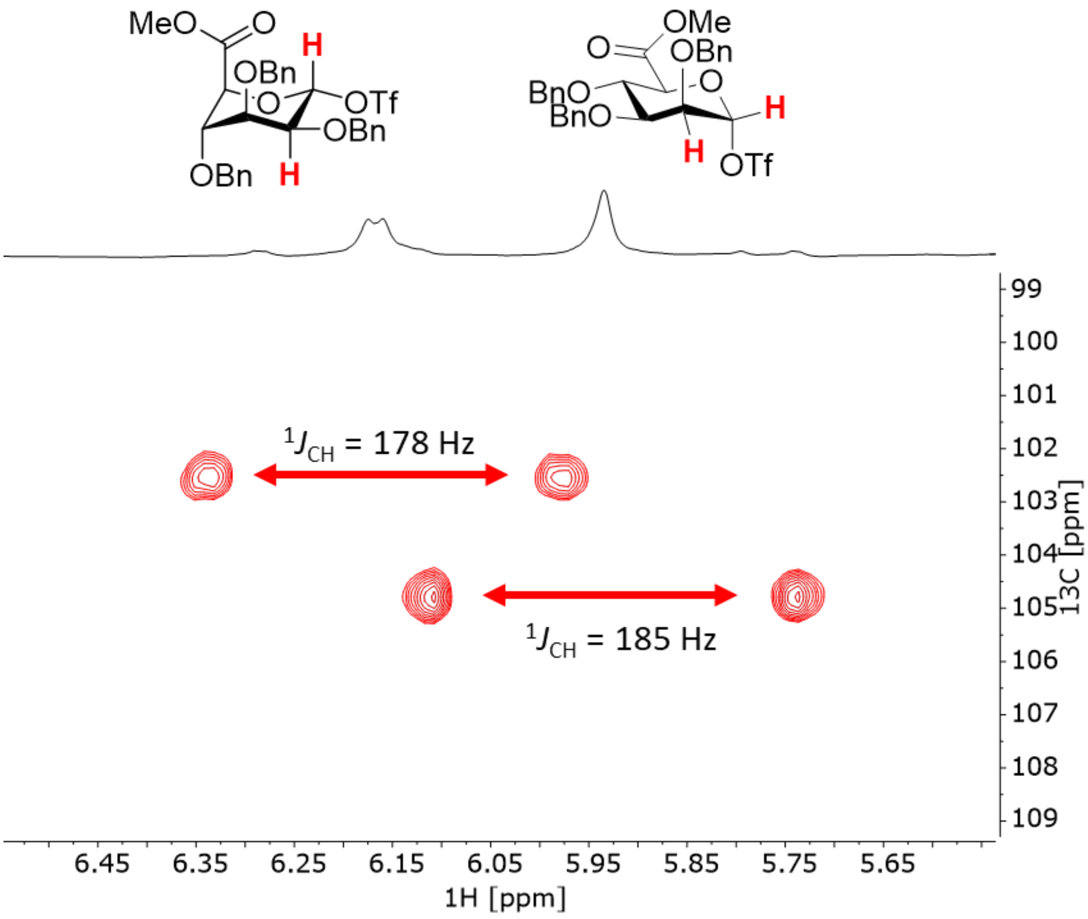

Figure S9: HSQC NMR spectrum of activated benzylated mannuronic acid donor.

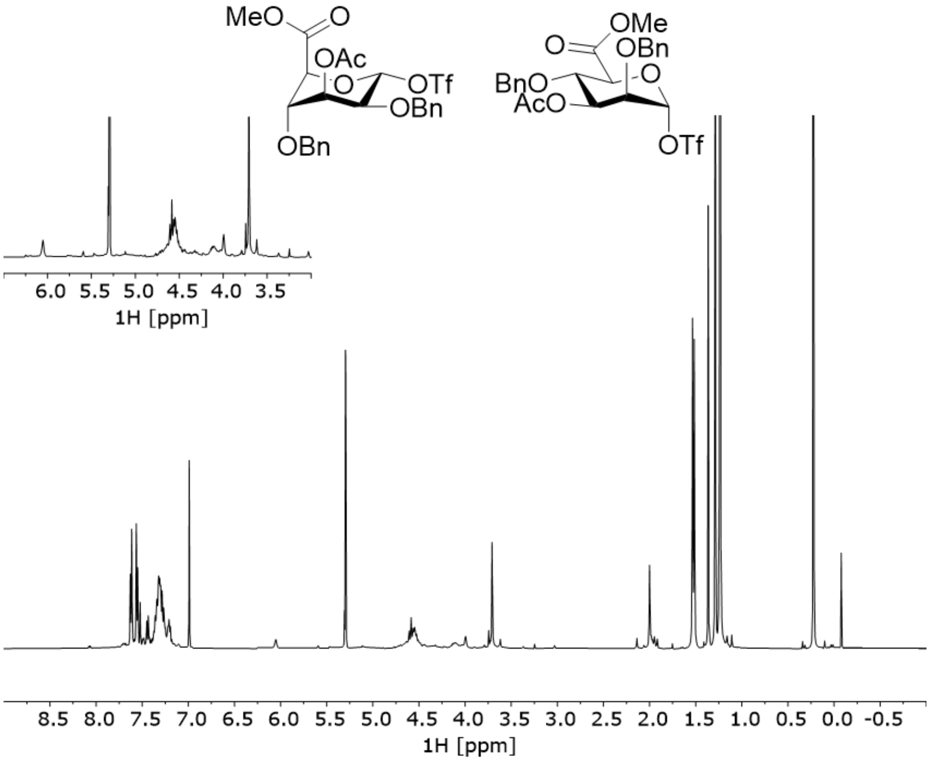

Figure S10: ^{1}H NMR spectrum of activated benzylated mannuronic acid donor equipped with a C-3 acetyl.

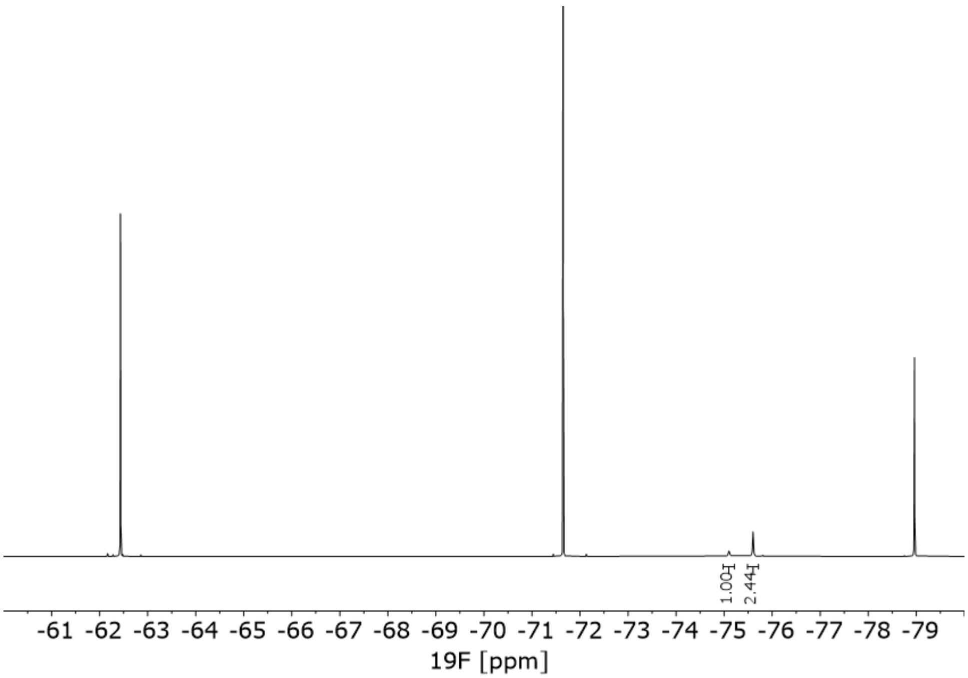

Figure S11: ^{19}F NMR spectrum of activated benzylated mannuronic acid donor equipped with a C-3 acetyl.

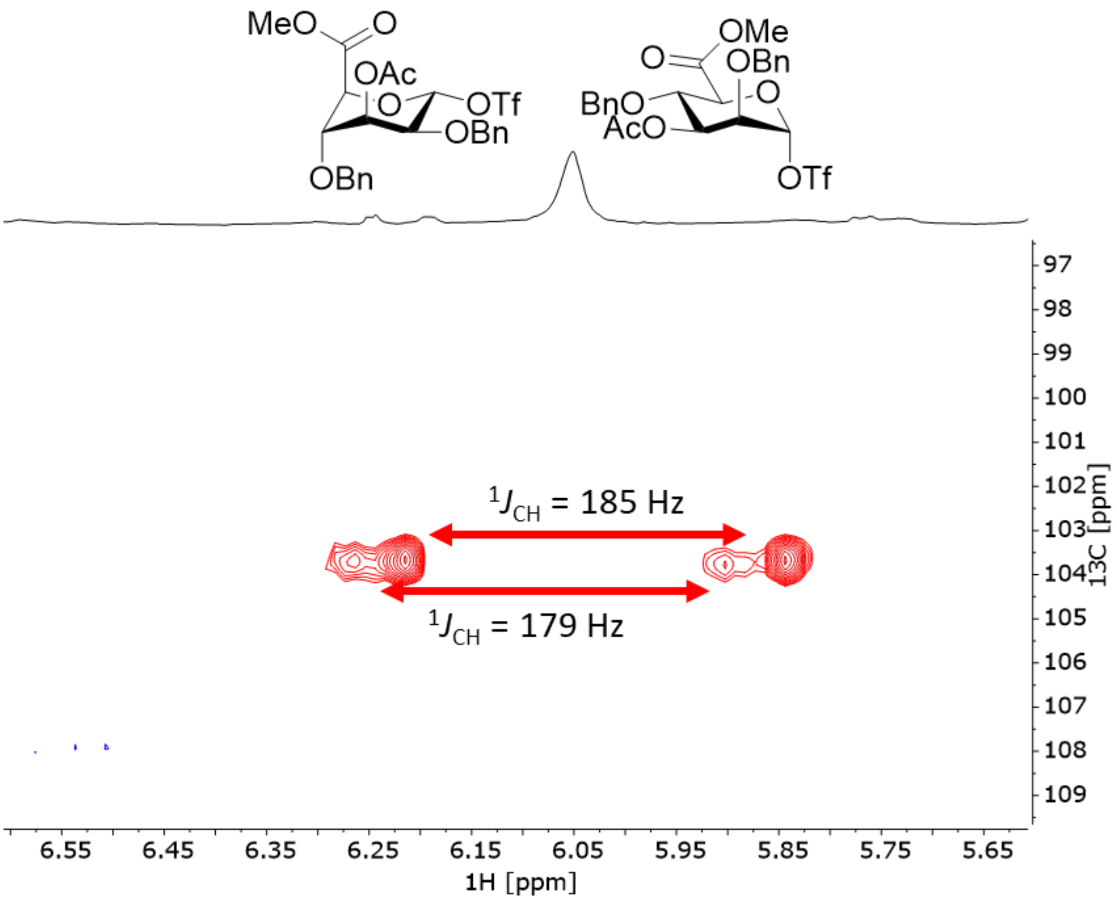

Figure S12: HSQC NMR spectrum of activated benzylated mannuronic acid donor equipped with a C-3 acetyl.

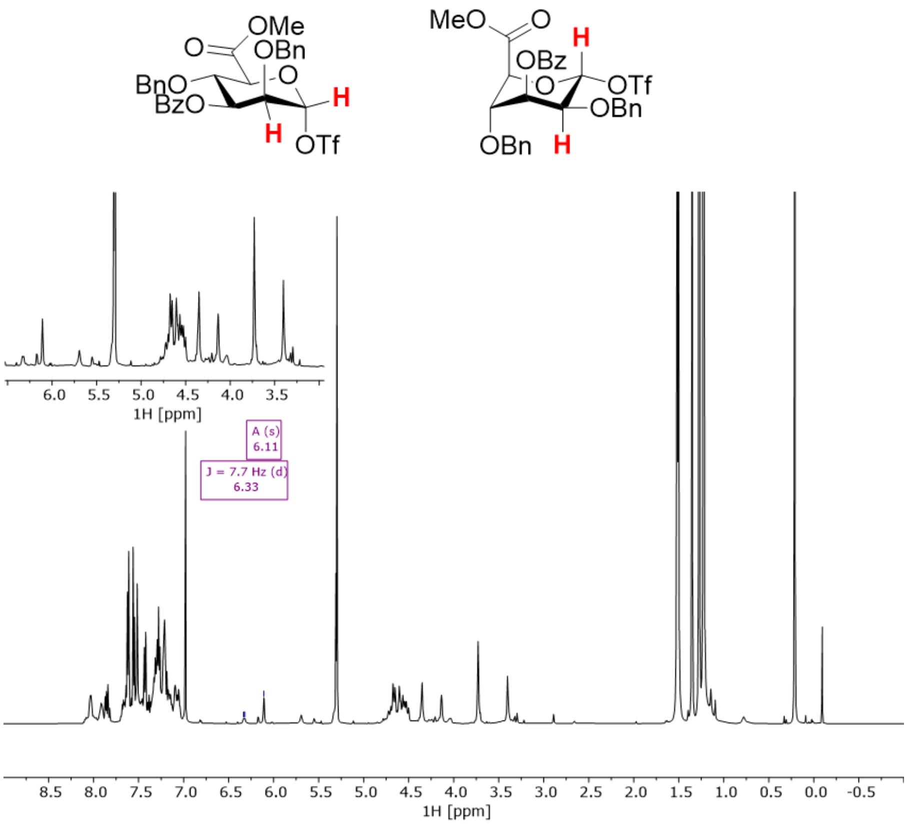

Figure S13: ^{1}H NMR spectrum of activated benzylated mannuronic acid donor equipped with a C-3 benzoyl.

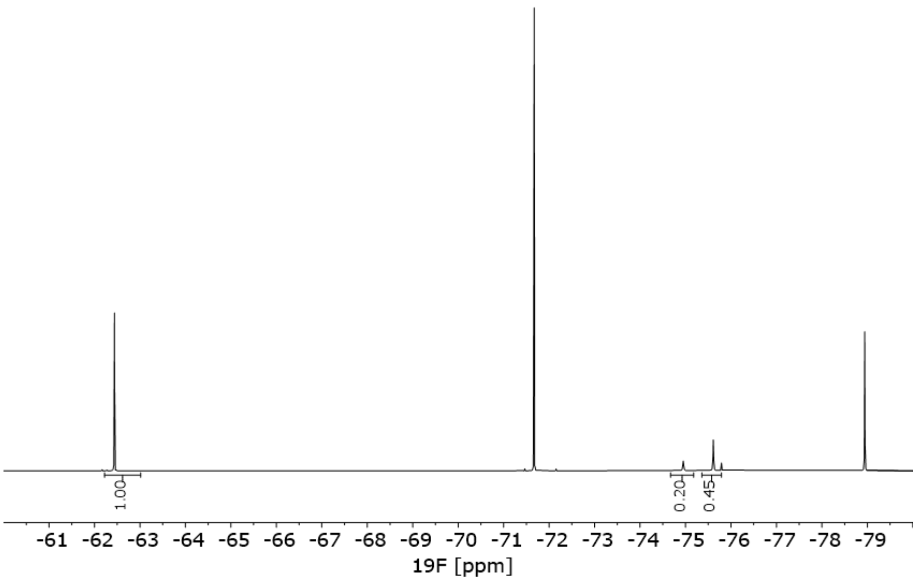

Figure S14: ^{19}F NMR spectrum of activated benzylated mannuronic acid donor equipped with a C-3 benzoyl.

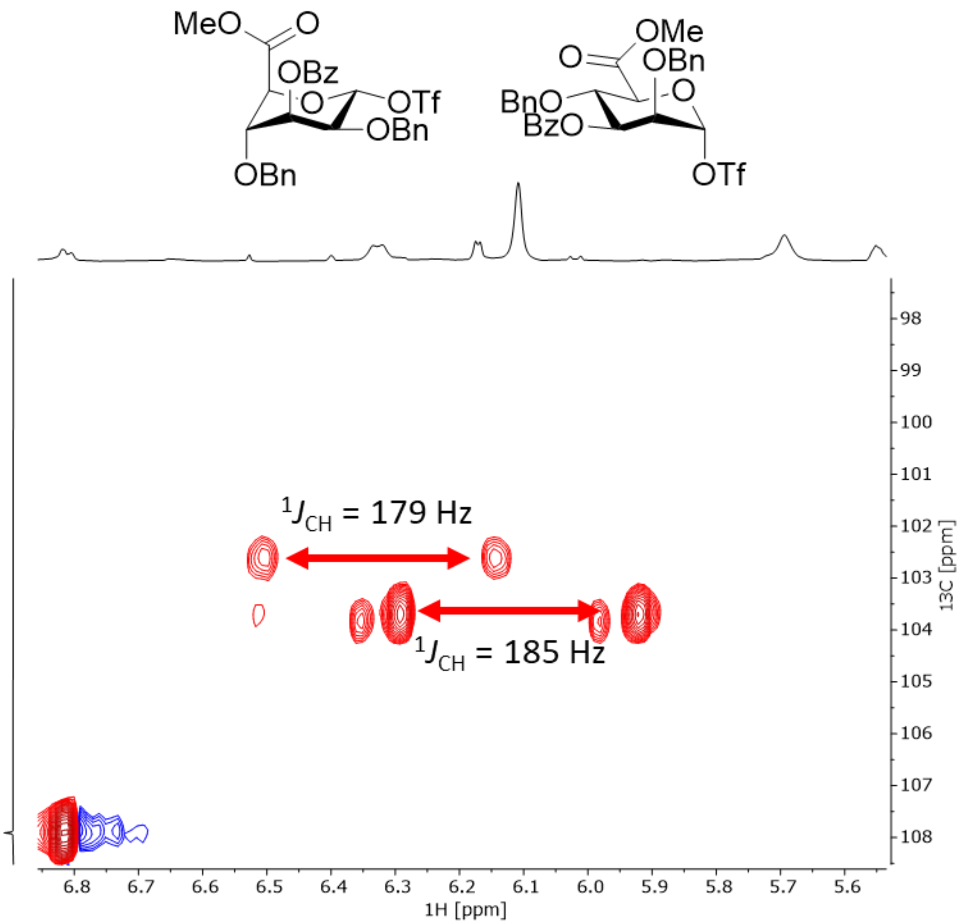

Figure S15: HSQC NMR spectrum of activated benzylated mannuronic acid donor equipped with a C-3 benzoyl.

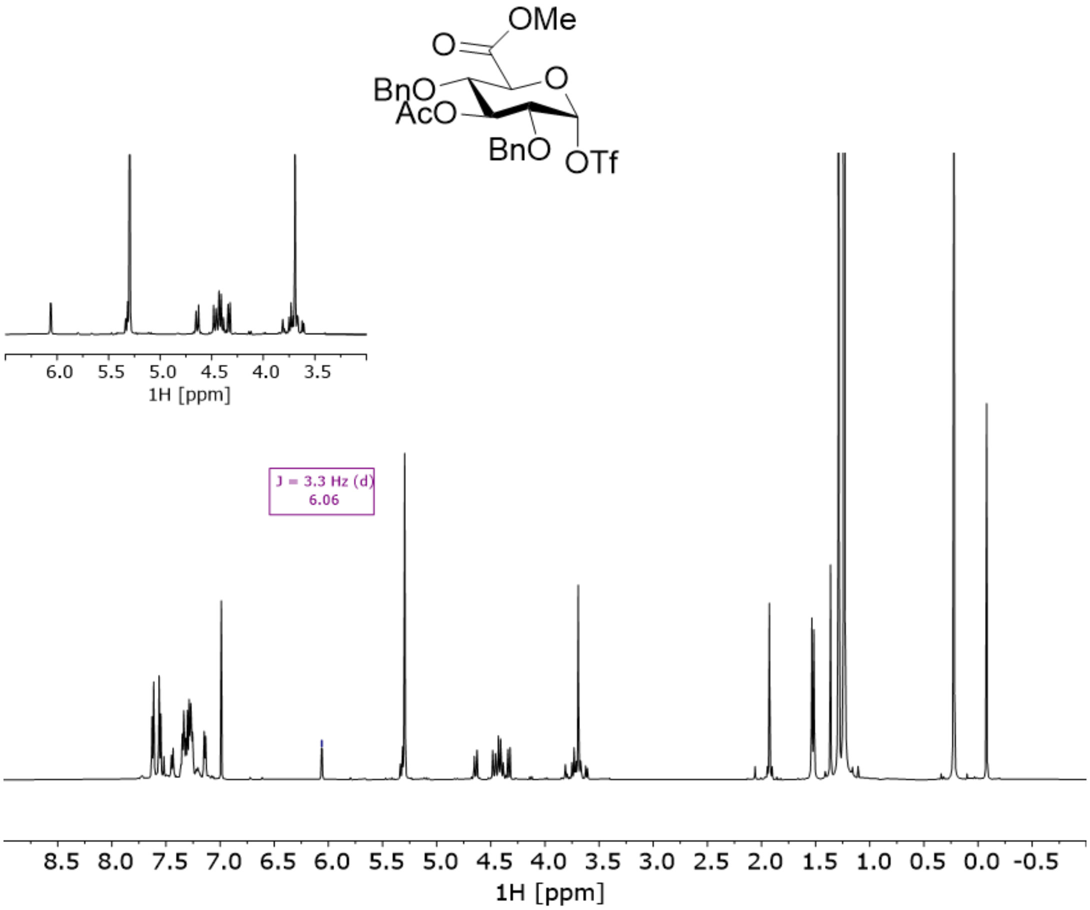

Figure S16: ^{1}H NMR spectrum of activated benzylated glucuronic acid donor equipped with a C-3 acetyl.

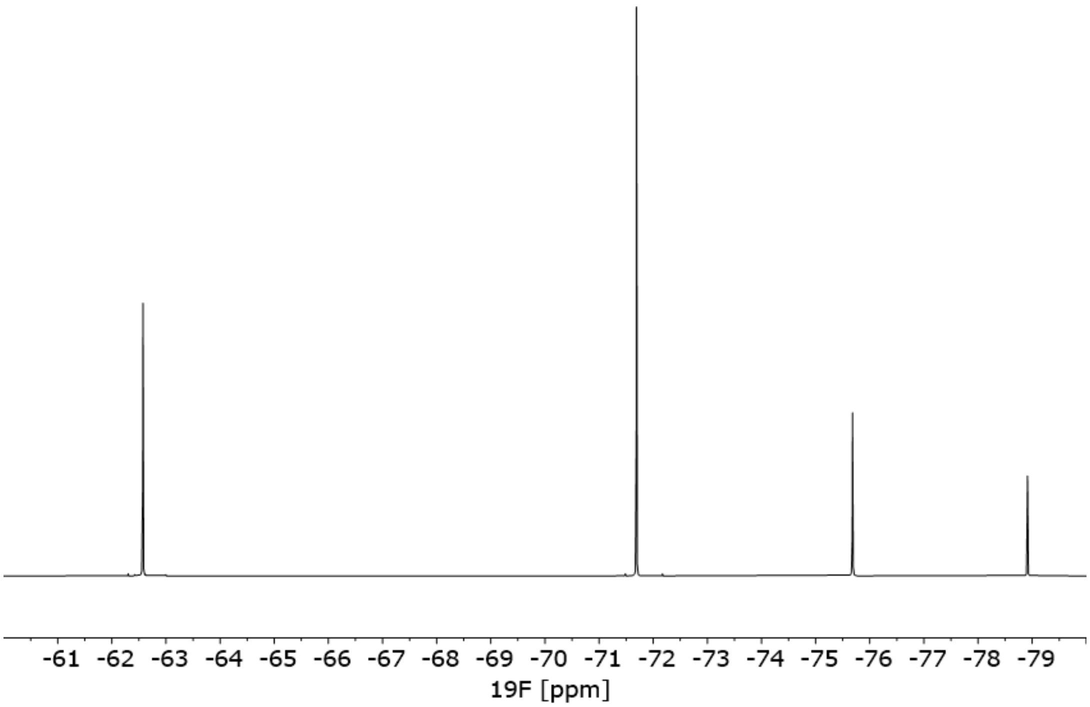

Figure S17: ^{19}F NMR spectrum of activated benzylated glucuronic acid donor equipped with a C-3 acetyl.

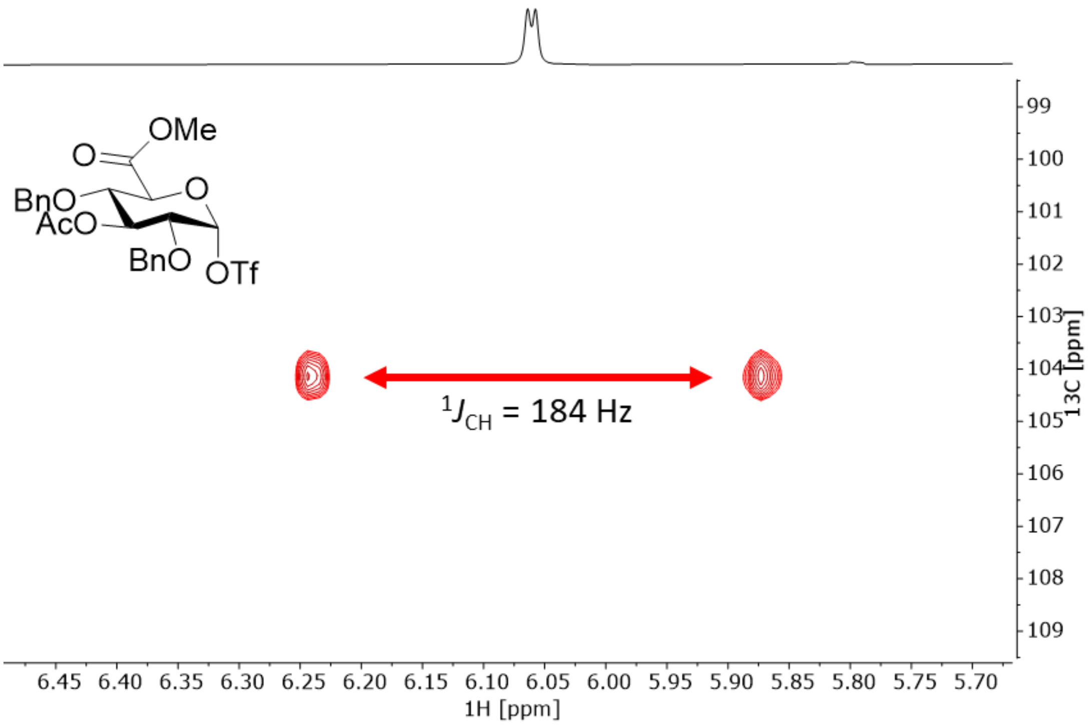

Figure S18: HSQC NMR spectrum of activated benzylated glucuronic acid donor equipped with a C-3 acetyl.

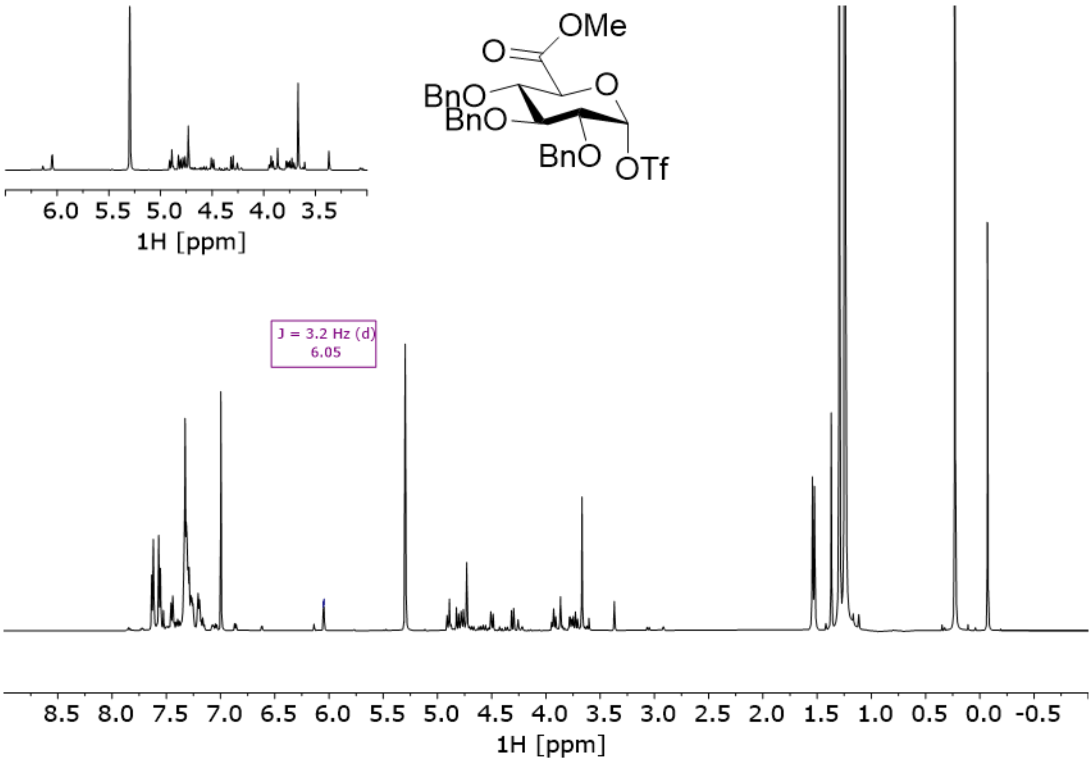

Figure S19: ^{1}H NMR spectrum of activated benzylated glucuronic acid donor.

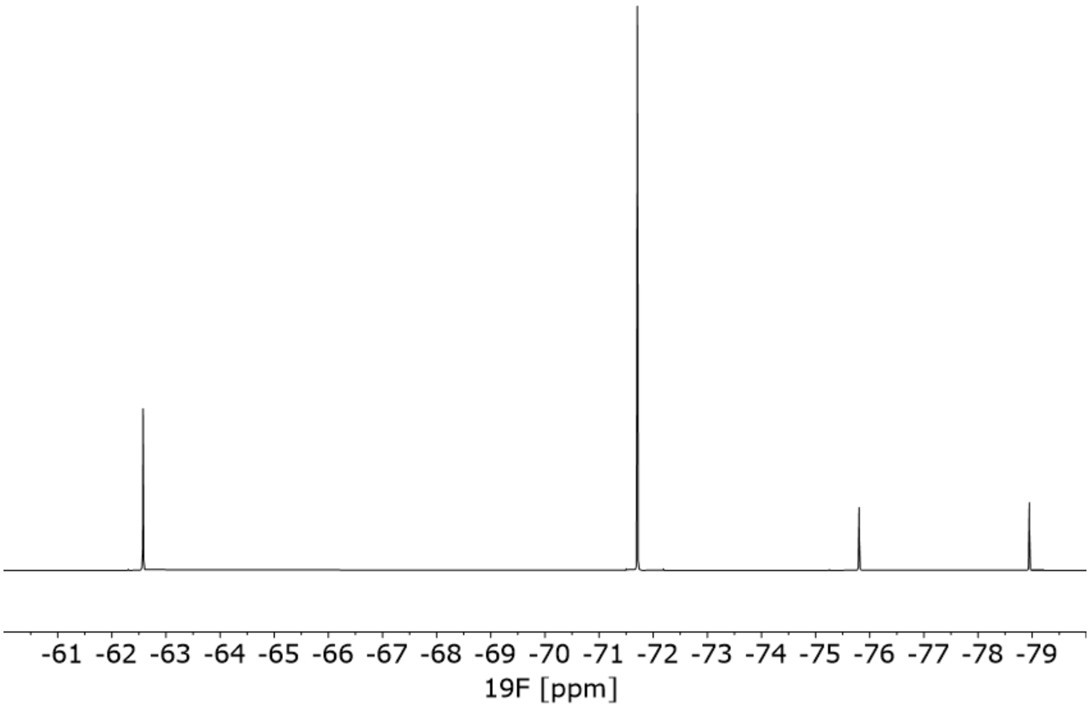

Figure S20: ^{19}F NMR spectrum of activated benzylated glucuronic acid donor.

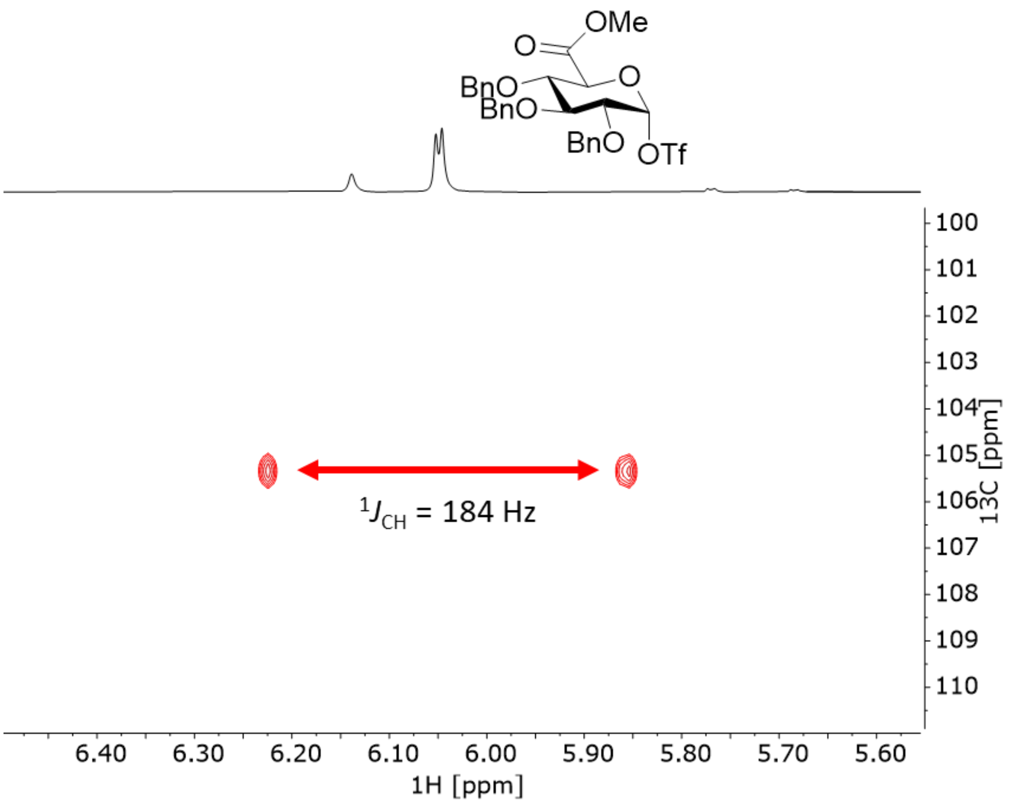

Figure S21: HSQC NMR spectrum of activated benzylated glucuronic acid donor.

Supporting NMR figures

A) System studied

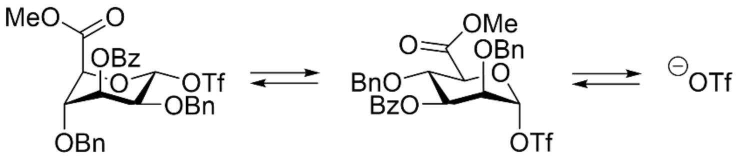

B) Graphical visualization of ^{19}F EXSY experiment to track OTf formation

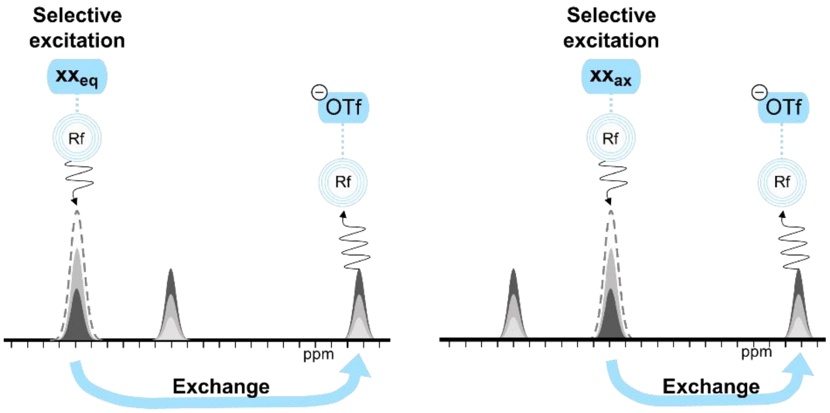

C) OTf formation upon selection of either equatorial or axial α-triflate

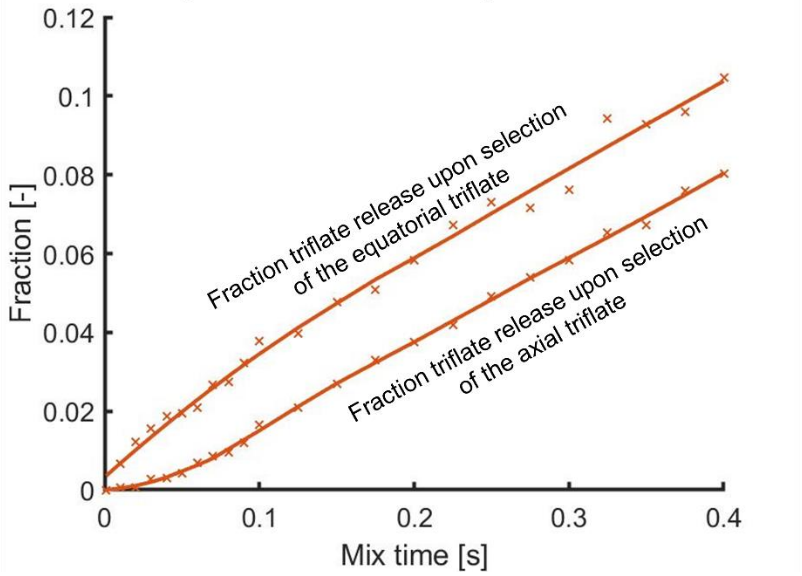

Figure S22: Comparing $^{-}$OTf resonance formation using ^{19}F EXSY upon selecting either the equatorial or axial α-triflate demonstrating that the equatorial α-triflate releases OTf faster compared to the axial α-triflate.

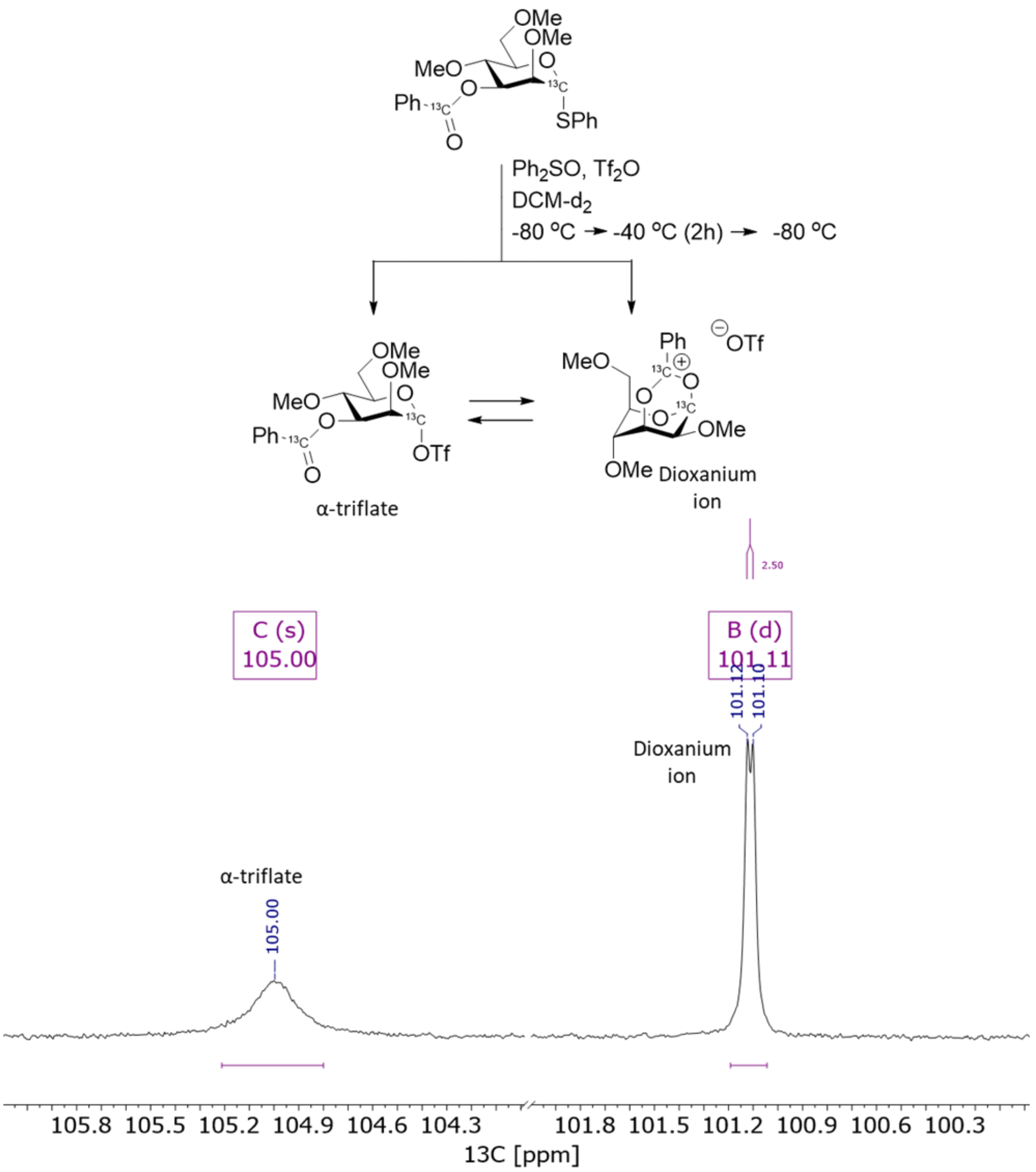

Figure S23: ^{13}C NMR spectrum after activation and equilibration for 2h at -40 °C, sample cooled to -80 °C at which temperature this spectrum was recorded.

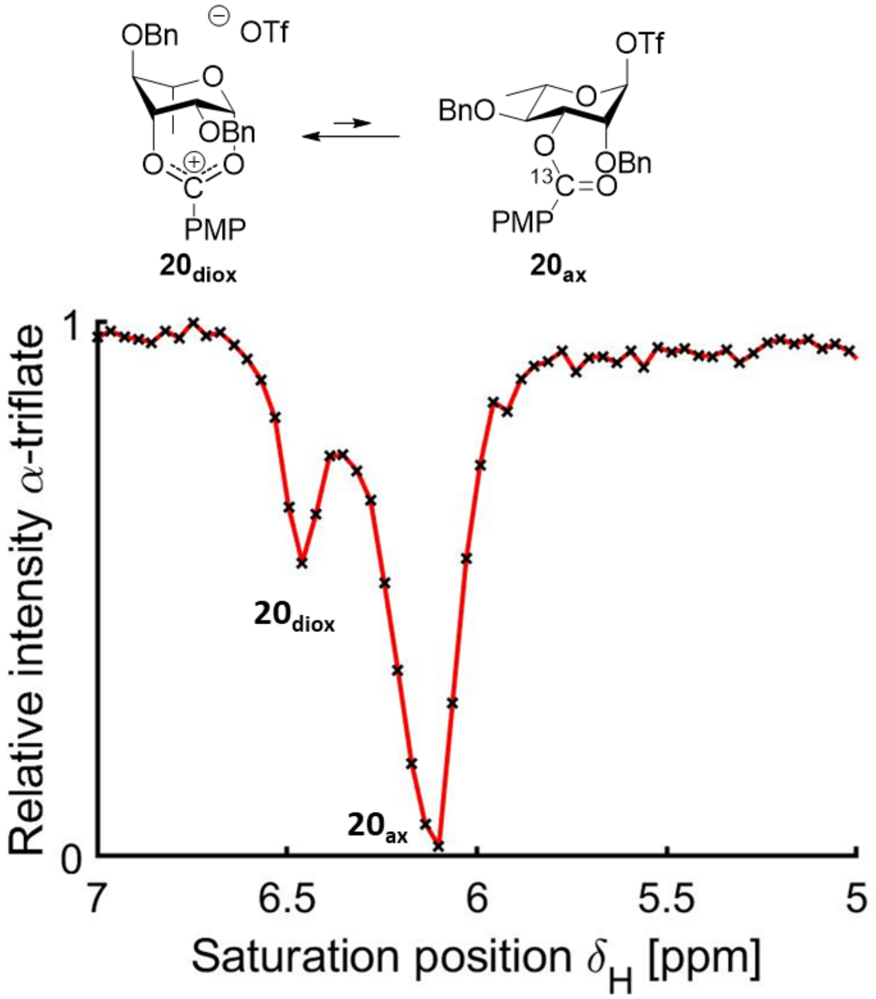

Figure S24: ^{1}H CEST profile on rhamnosyl α-triflate 20_{ax} displaying it being in chemical exchange with a species at δ_H = 6.46 ppm which is in good agreement to the chemical shift of the anomeric proton of the dioxanium ion as previously observed.[9] Hence, no evidence for a ring-flipped rhamnosyl α-triflate was observed.

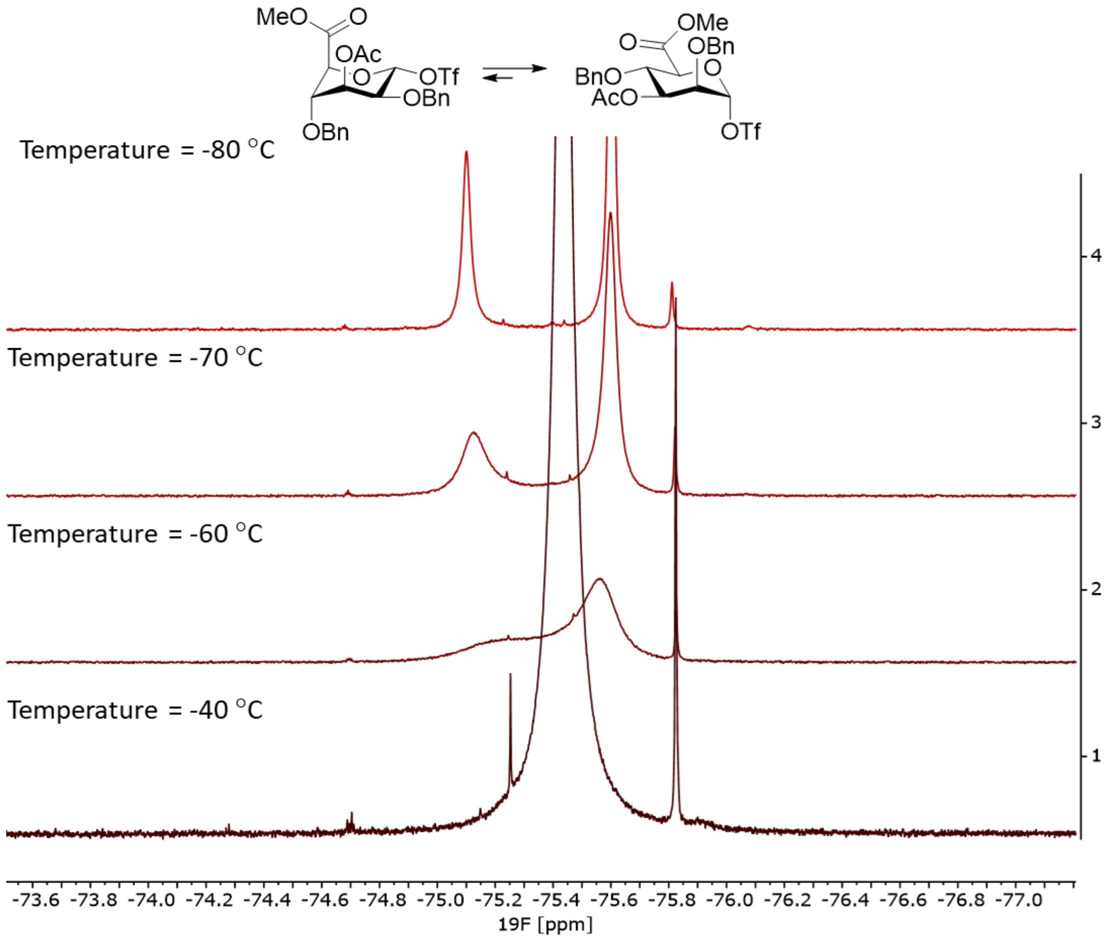

Figure S25: Mannuronic acid α-triflates equipped with a C-3 acetyl protecting group, ^{19}F spectra at varying temperatures.

Rhamnose: Assigning the minor resonance in 19F NMR as the equatorial α-triflate

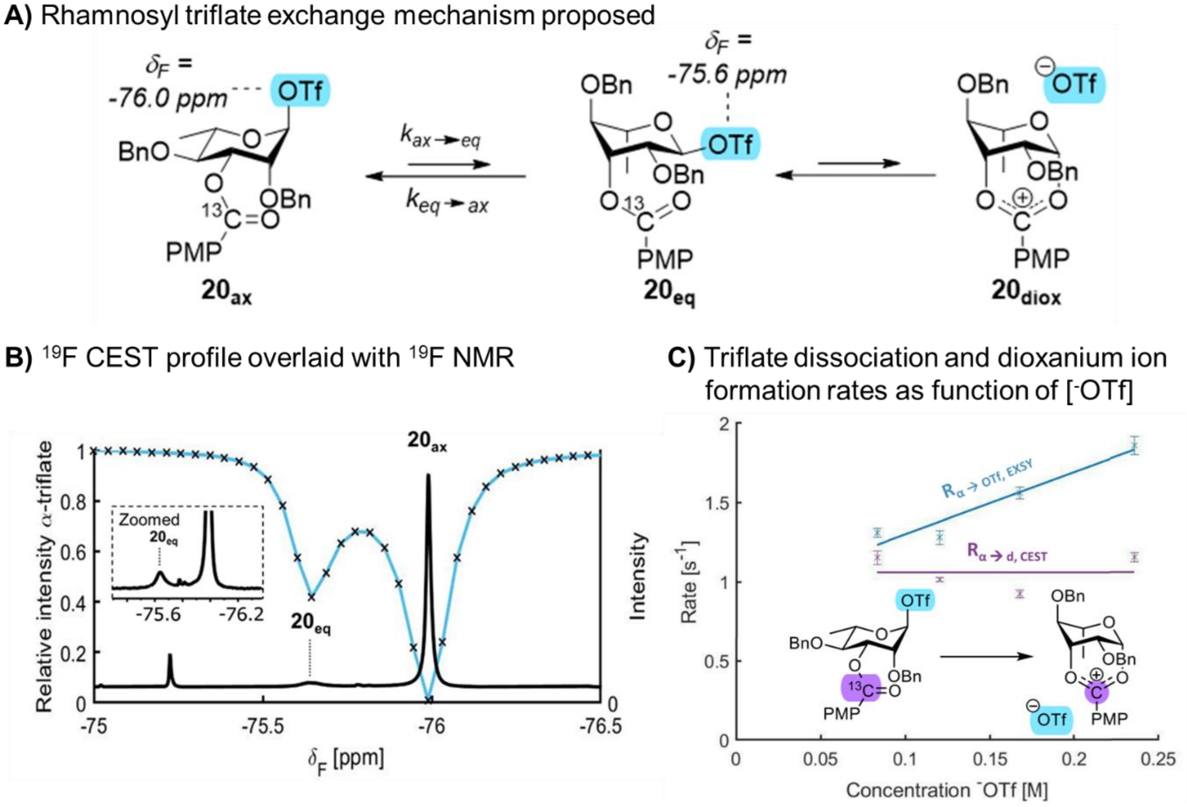

Figure S26: A) Rhamnosyl triflate exchange mechanism proposed; B) ^{19}F CEST profile overlaid with ^{19}F NMR; C) Triflate dissociation and dioxanium ion formation rates as function of [$^{-}$OTf] as previously reported.[9]

CEST NMR is a powerful tool to detect low abundant reaction intermediates and study what it is in chemical exchange with. However, the technique typically only provides a chemical shift which is related to the molecular structure of the molecule. The information is thus rather limited. Therefore assigning CEST signals in the CEST profile should proceed with caution. In the context of glycosylation, ring-flipping, and axial α- to equatorial β-triflate formation it is particularly difficult.

In order to rationalize the peak at δ_F = -75.6 ppm to be the equatorial α-triflate, the rate at which the axial α-triflate is becoming this resonance was determined using ^{19}F CEST NMR. The resonance at δ_F = -75.6 ppm was saturated by varying the saturation duration (see pages S4-9). The rate determined using CEST then gives the rate at which the axial α-triflate (δ_F = -76.0 ppm) becomes the unknown species at δ_F = -75.6 ppm (pages S4-9).

The rate determined by ^{19}F EXSY was 4.2 s^{-1} (Figure S89). Integration of the rhamnosyl triflate two resonances yields a ratio of 10:1 which suggests that the rate of the resonance at δ_F = -75.6 ppm becoming the axial α-triflate is about 43 s^{-1}.

Previously we reported axial α-triflate dissociation to be about 0.15 s^{-1} faster than dioxanium ion formation under standard activation conditions. (Figure S26). The rate of

axial α-triflate dissociation increased when tetrabutyl ammonium triflate was added, suggesting formation of the equatorial β-triflate. Hence, the resonance at δ_F = -75.6 ppm could also be the equatorial β-triflate. Since the axial α-triflate is becoming the resonance at δ_F = -75.6 ppm much faster compared dissociation of the axial α-triflate, this resonance (at δ_F = -75.6 ppm) cannot be the equatorial β-triflate. We therefore assigned the resonance to the equatorial α-triflate rather than the equatorial β-triflate.

A final notable effect is observed when changing the readout resonance of the ^{19}F CEST experiment (Figure S27C). Only a decay in the free triflate resonance ($^{-}$OTf) of ≈15% is observed when saturating the unknown resonance at δ_F = -75.6 ppm. In contrast, having the axial α-triflate as read-out, a 60% reduction of the axial α-triflate resonance is observed upon saturation of the resonance at δ_F = -75.6 ppm (Figure S26B). This suggests a direct correlation (Figure S27A). If the resonance at δ_F = -75.6 ppm is the equatorial β-triflate, then it should first dissociate followed by multiple attacks at the rhamnosyl triflate to affect the peak intensity of the α-triflate (Figure S27B). With so much $^{-}$OTf ions in solution it is unlikely that the singly saturated $^{-}$OTf ion coming from a low populated equatorial β-triflate can cause such effect.

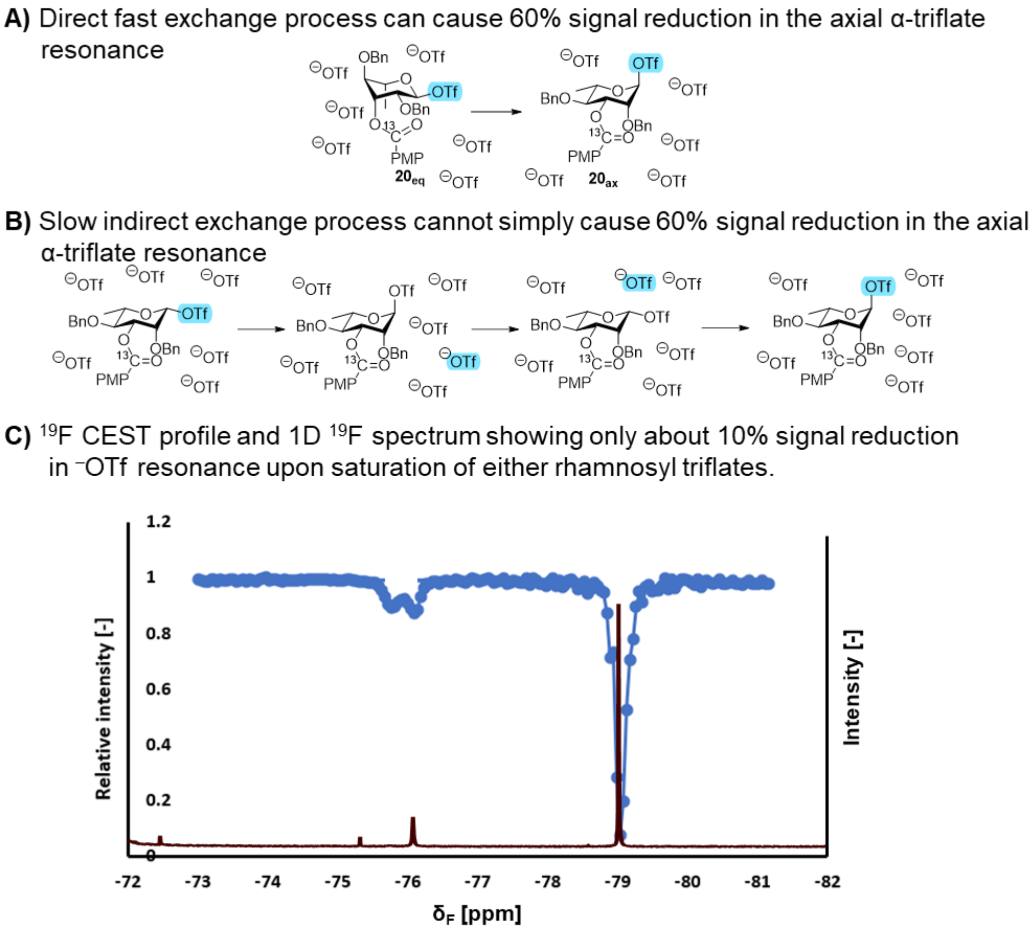

Figure S27: A+B) Mechanisms of saturation transfer to affect the axial a-triflate; C) ^{19}F CEST profile and ^{19}F spectrum to demonstrate the effect of saturation on the rhamnosyl triflates on the $^{-}$OTf resonance.

Supporting IRIS figures

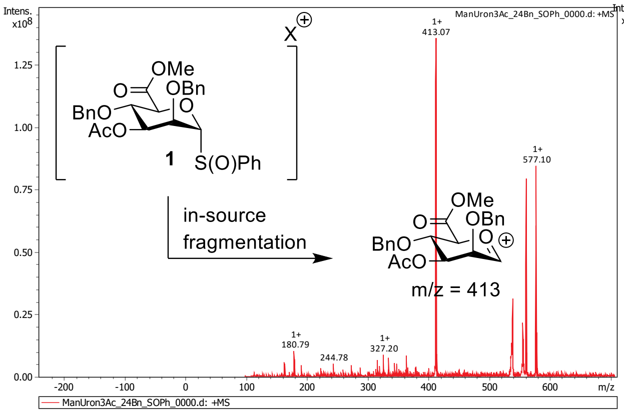

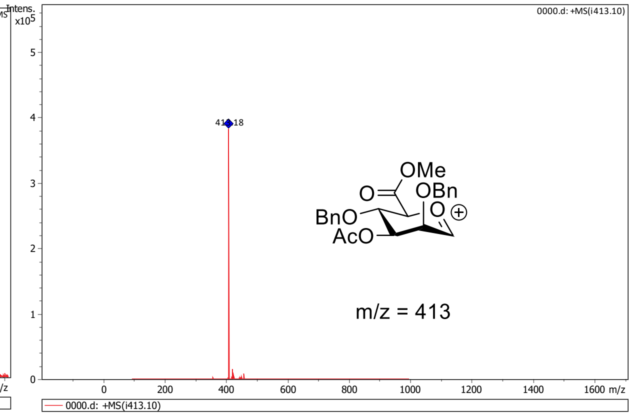

Figure S28: MS-spectrum of compound **1** (left) and isolation of in-source fragment m/z = 413 (right).

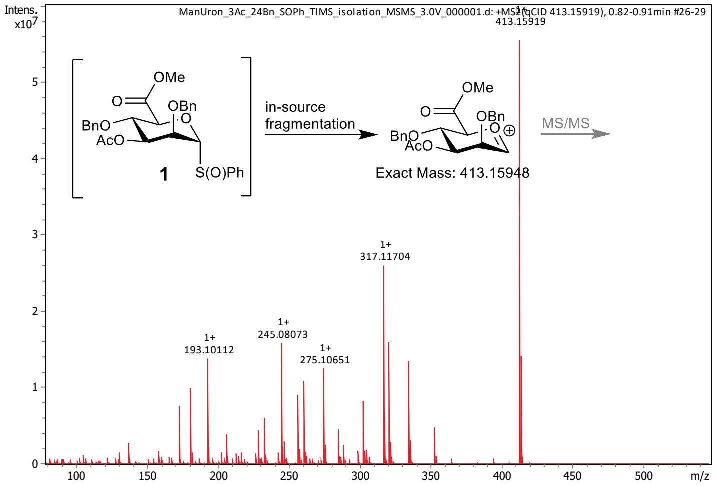

Figure S29: FTICR MS spectrum of in-source fragment with m/z = 461.19585 and its subsequent MS/MS fragmentation.

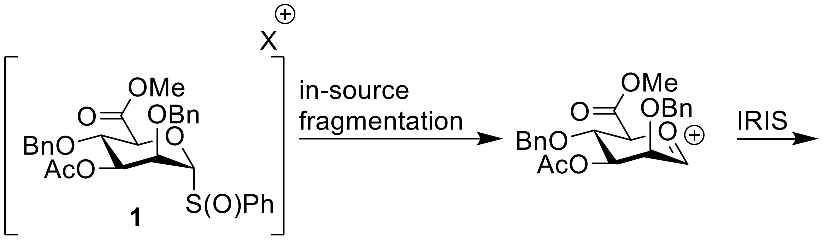

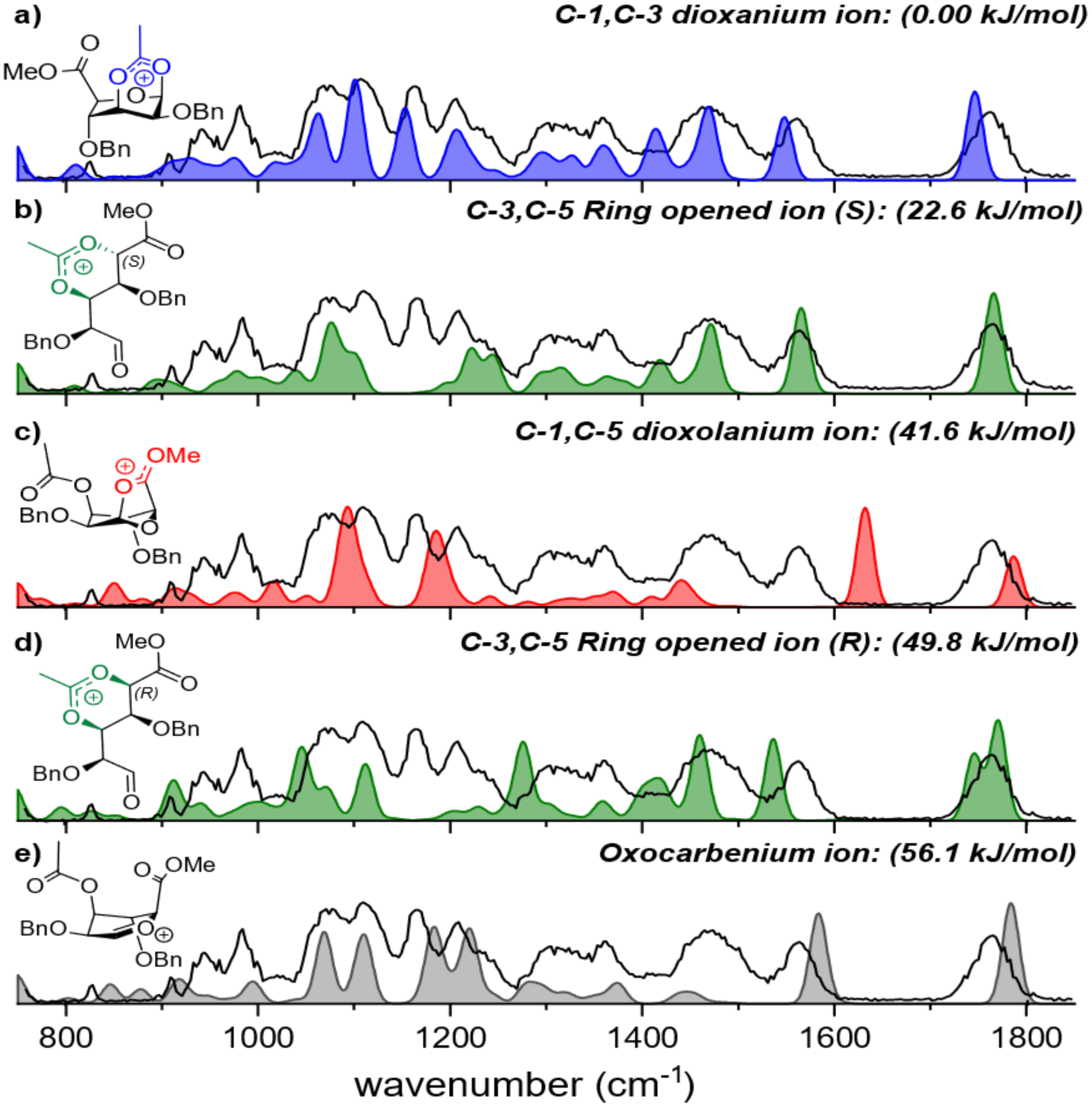

Figure S30: Comparison of the experimental IR spectrum of the mannuronic acid cation of 1 with m/z 413 (black) to the calculated spectra (coloured) of the C-1,C-3-dioxanium ion (blue, a), the ring opened C-3,C-5 (*S*) ion (green, b), the C1,C-5-dioxolanium ion (red, c), the ring opened C-3,C-5 (*R*) ion (green, d) and the oxocarbenium ion (grey, e). Relative free energies are given. Exact coordinates of the depicted 3D structures can be found below.

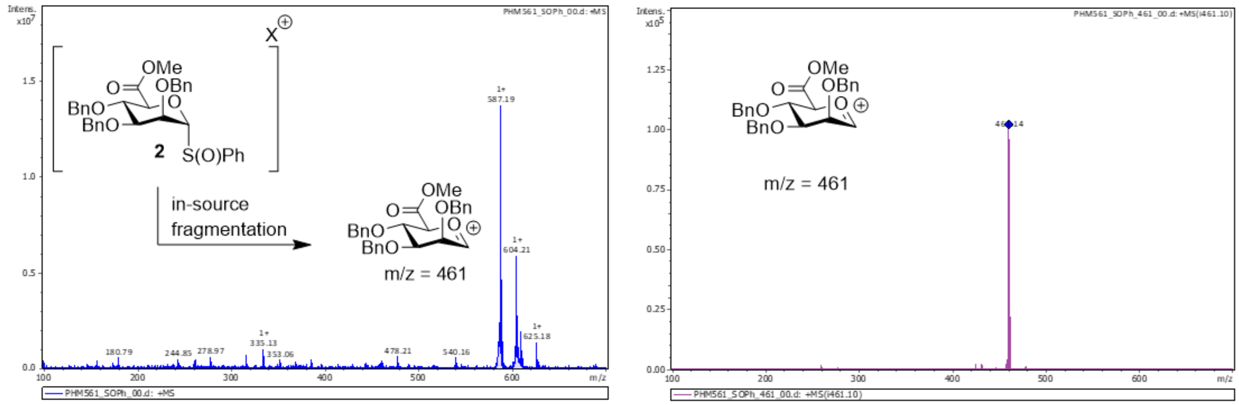

Figure S31: MS-spectrum of compound **2** (left) and isolation of in-source fragment m/z = 461 (right).

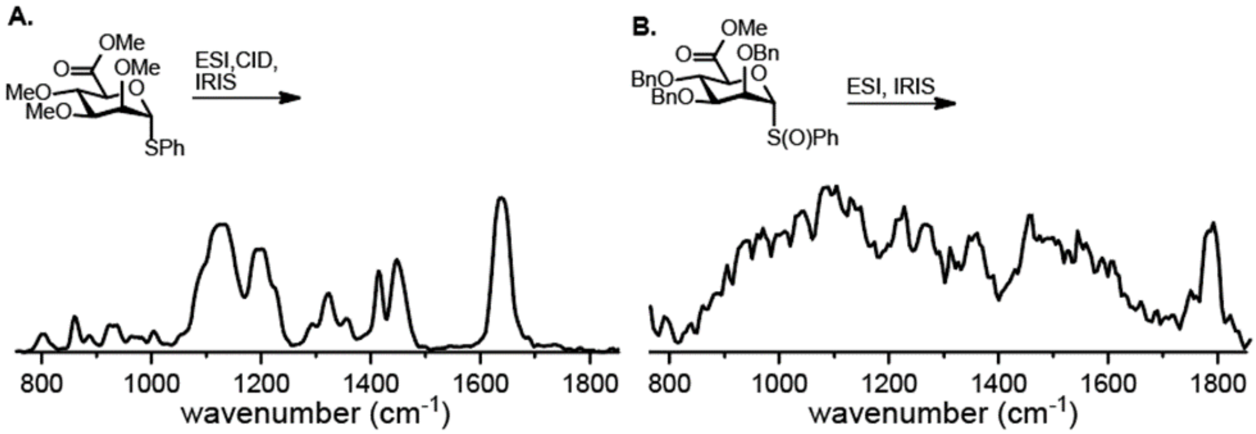

Figure S32: Comparison of the IR ion spectra of cations resulting from the permethylated Mannuronic acid donor (left) that was previously measured and the perbenzylated Mannuronic acid donor (right) used in this work. Panel A is adapted from Elferink et al.[10]

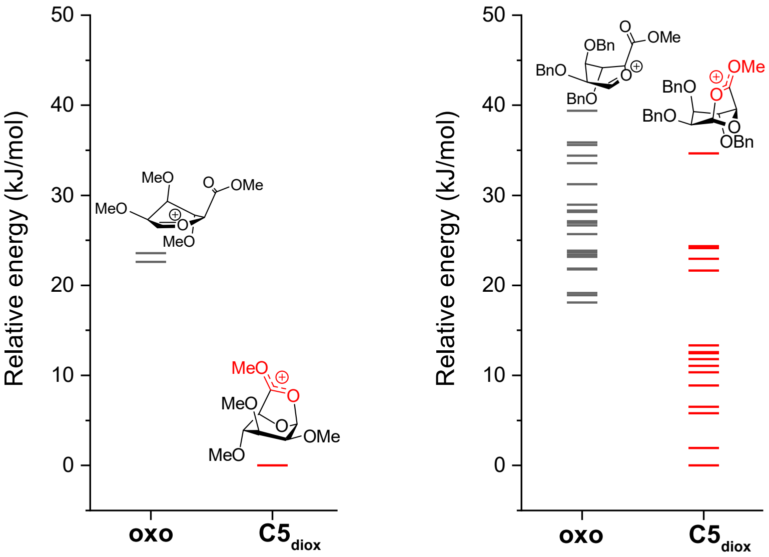

Figure S33: Energy hierarchies of the reoptimized structures for permethylated Mannuronic acid cations (left) and perbenzylated Mannuronic acid cations (right). The relative Gibbs free energies were calculated combining the electronic energies from MP2/6-31++G(d,p) single point calculations of the B3LYP optimized geometries with the thermal correction resulting from the B3LYP vibrational analysis (at 298K).

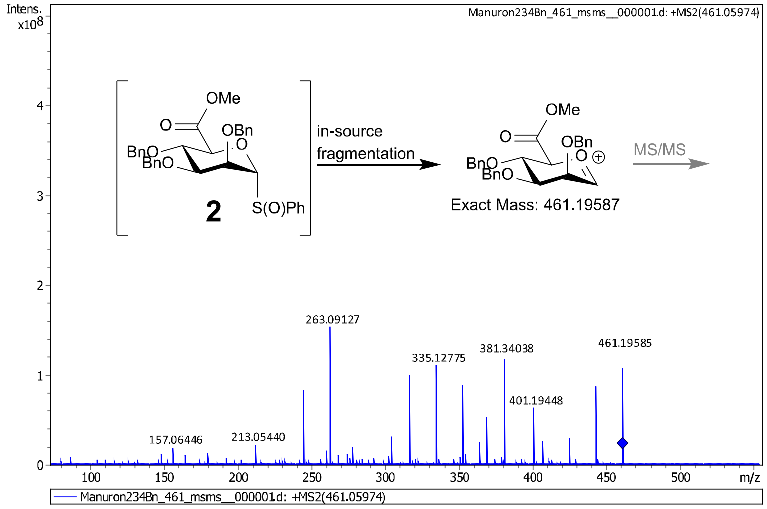

Figure S34: FTICR MS spectrum of in-source fragment with m/z = 461.19585 and its subsequent MS/MS fragmentation.

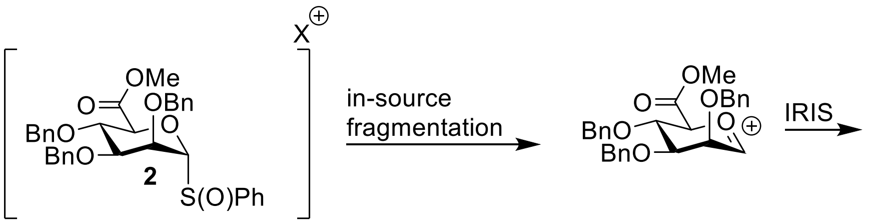

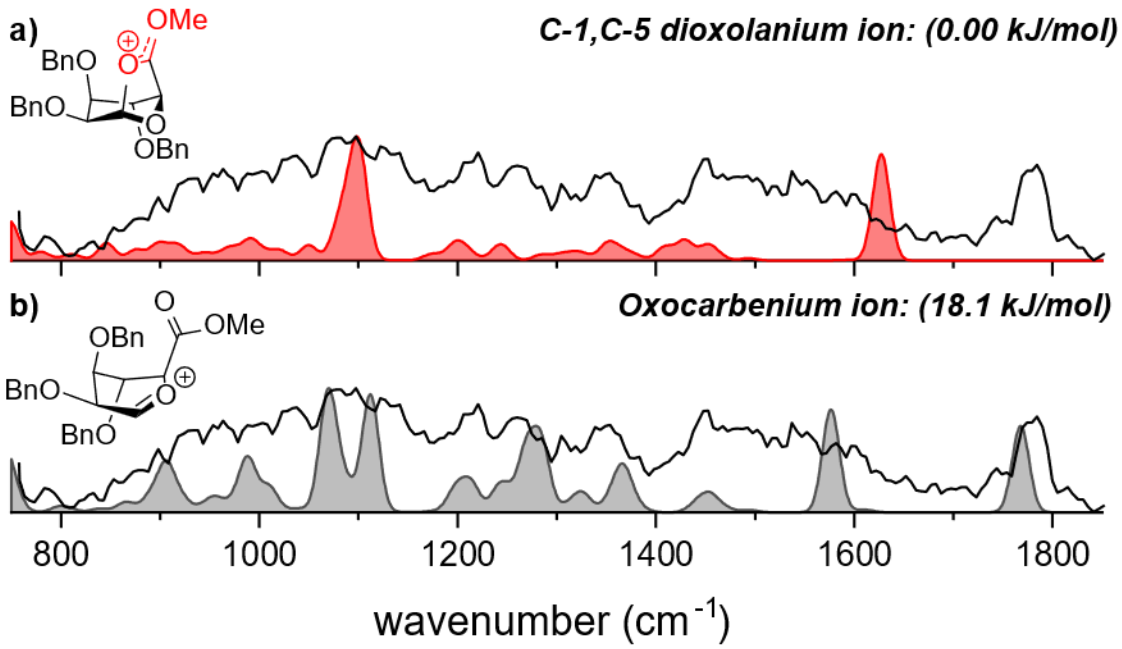

Figure S35: Comparison of the experimental IR spectrum of the mannuronic acid cation of 2 with m/z 461 (black) to the calculated spectra (coloured) of the C1,C-5-dioxolanium ion (red, a) and the oxocarbenium ion (grey, b). Relative free energies are given. Exact coordinates of the depicted 3D structures can be found below.

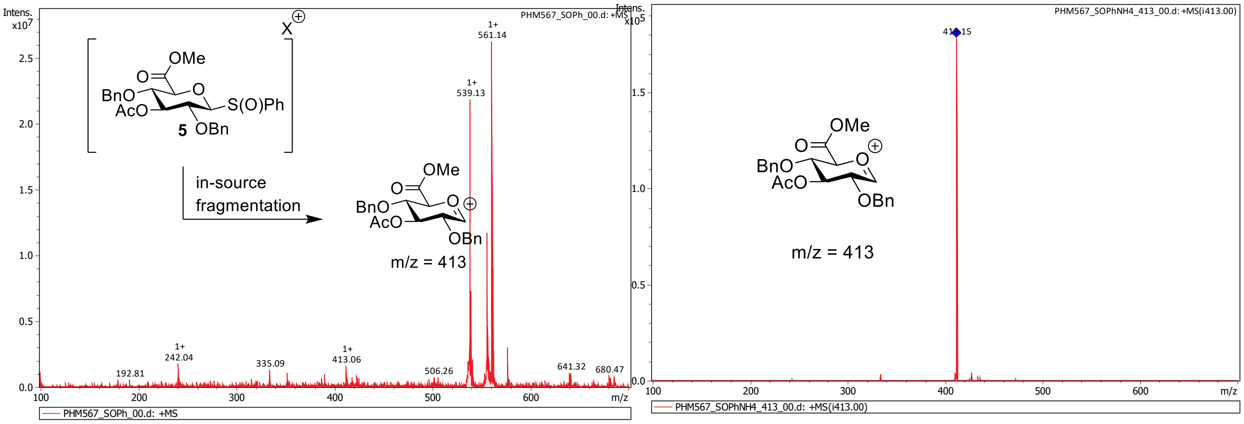

Figure S36: MS-spectrum of compound **5** (left) and isolation of in-source fragment m/z = 413 (right).

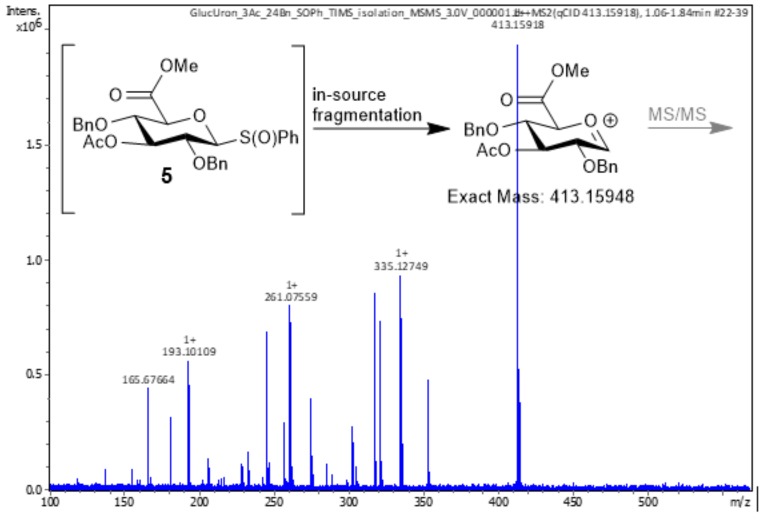

Figure S37: FTICR MS spectrum of in-source fragment with m/z = 413.15948 and its subsequent MS/MS fragmentation.

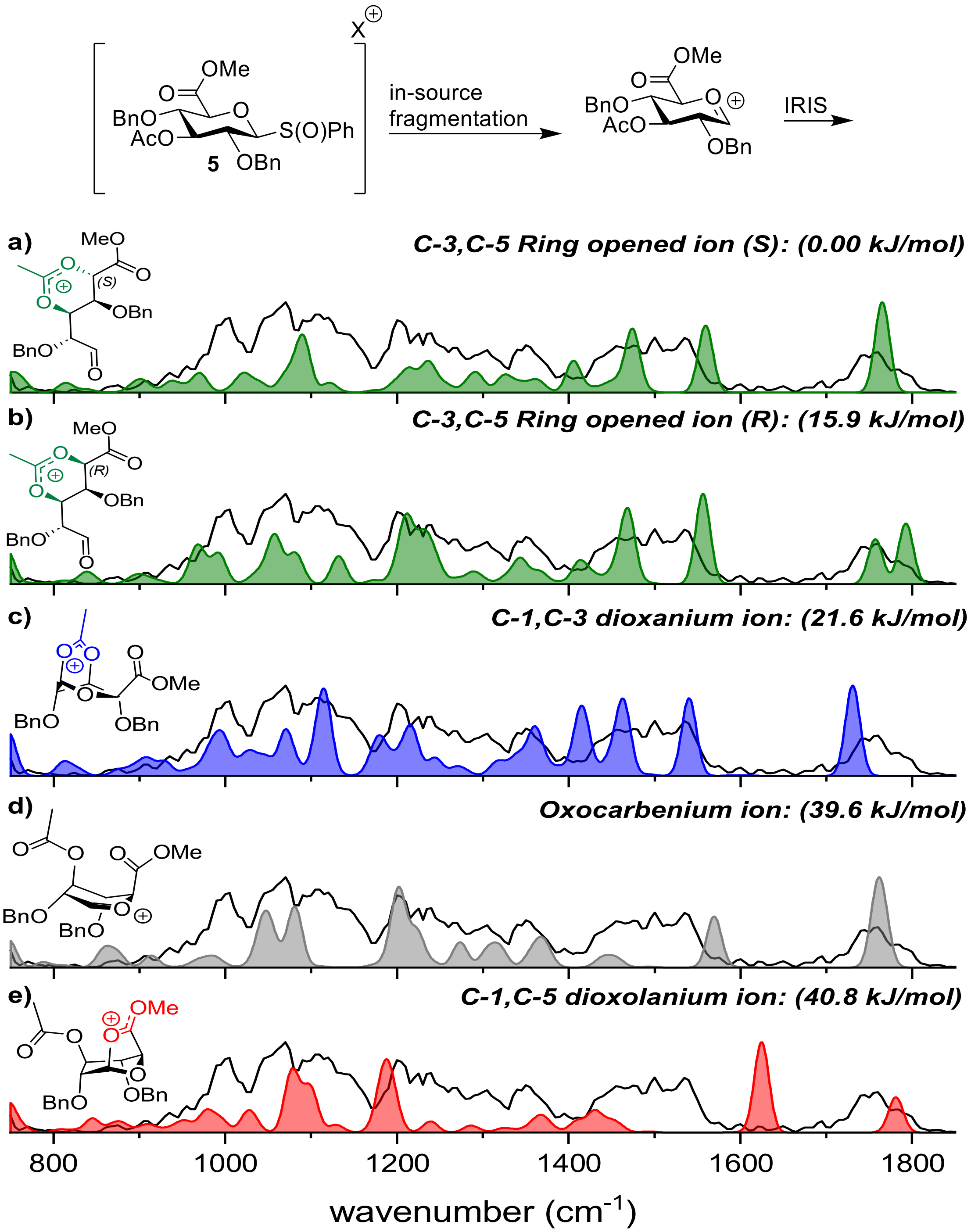

Figure S38: Comparison of the experimental IR spectrum of the glucuronic acid cation of 5 with m/z 413 (black) to the calculated spectra (coloured) of the ring opened C-3,C-5 (S) ion (green, a), the ring opened C-3,C-5 (R) ion (green, b), the C-1,C-3-dioxanium ion (blue, c), oxocarbenium ion (grey, d) and the C1,C-5-dioxolanium ion (red, e). Relative free energies are given. Exact coordinates of the depicted 3D structures can be found below.

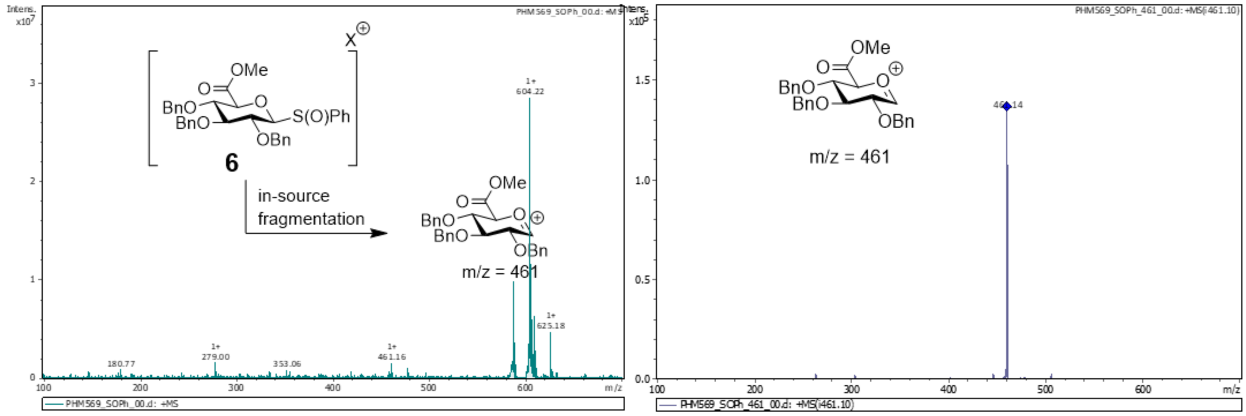

Figure S39: MS-spectrum of compound 6 (left) and isolation of in-source fragment m/z = 461 (right).

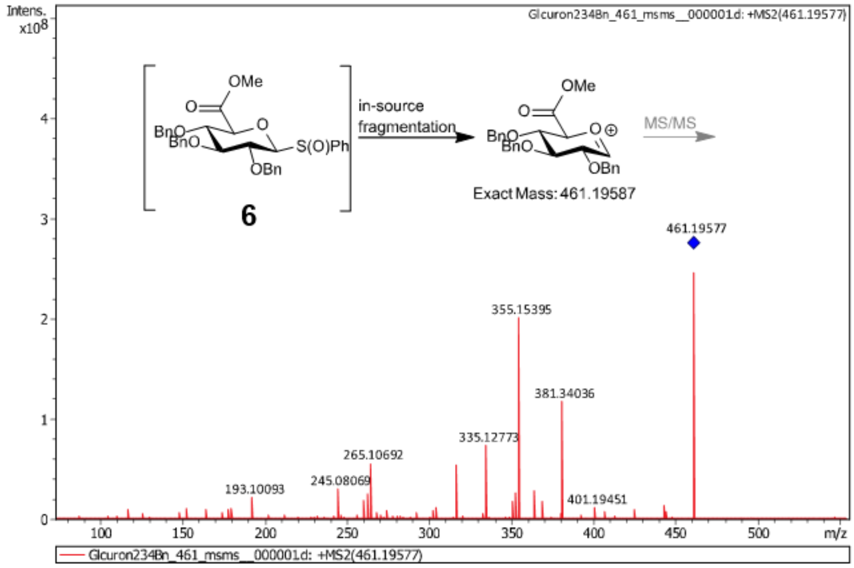

Figure S40: FTICR MS spectrum of in-source fragment with m/z = 461.19577 and its subsequent MS/MS fragmentation.

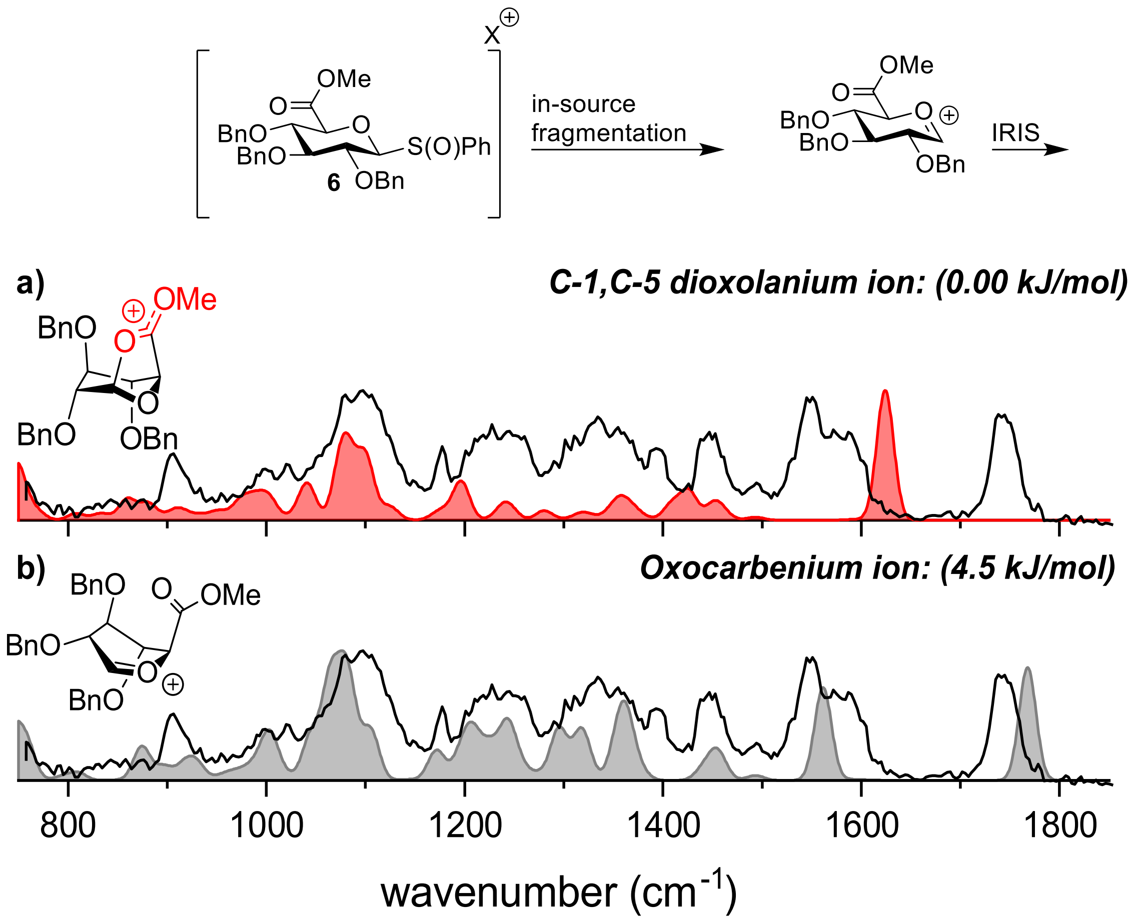

Figure S41: Comparison of the experimental IR spectrum of the glucuronic acid cation of 6 with m/z 461 (black) to the calculated spectra (coloured) of the C1,C-5-dioxolanium ion (red, a) and the oxocarbenium ion (grey, b). Relative free energies are given. Exact coordinates of the depicted 3D structures can be found below.

Supporting exchange rates

Table S1: Supporting rates Figure 6.

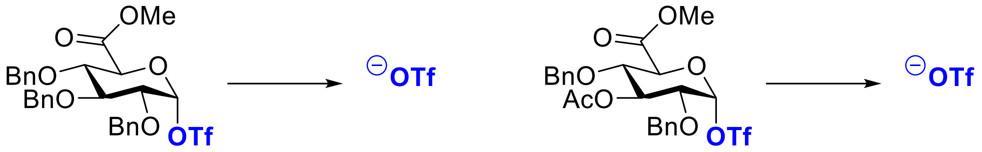

Conc. $^{-}$OTf [M]	$R_{\alpha \rightarrow OTf, EXSY}$ [s^{-1}]	Conc. $^{-}$OTf [M]	$R_{\alpha \rightarrow OTf, EXSY}$ [s^{-1}]
0.026437	0.0881 ± 0.005	0.034294	0.0945 ± 0.004
0.06322	0.211 ± 0.004	0.082156	0.228 ± 0.004
0.118011	0.409 ± 0.01	0.1355	0.358 ± 0.007
0.190633	0.653 ± 0.02	0.217134	0.580 ± 0.02

Table S2: Supporting rates Figure 6 and ring flip interconversion rates.

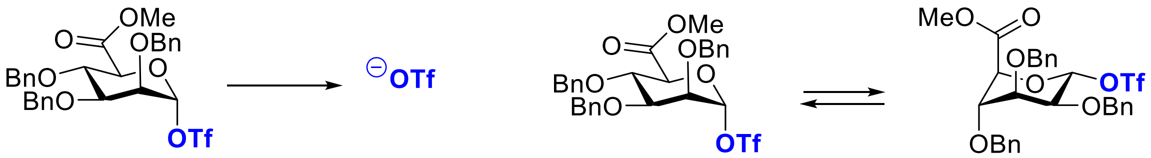

Conc. $^{-}$OTf [M]	$R_{\alpha \rightarrow OTf, EXSY}$ [s^{-1}]	$R_{ax \rightarrow eq, EXSY}$ [s^{-1}]	$R_{eq \rightarrow ax, EXSY}$ [s^{-1}]
0.0667	0.0880 ± 0.005	21.7 ± 1.2	22.4 ± 1.8
0.123	0.161 ± 0.01	24.1 ± 1.7	23.6 ± 1.7
0.164	0.185 ± 0.01	19.7 ± 1.2	20.9 ± 0.6
0.205	-	17.7 ± 1.3	21.5 ± 2.0

Table S3: Supporting rates Figure 6 and ring flip interconversion rates.

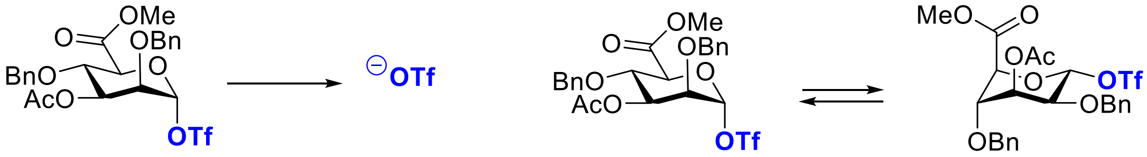

Conc. $^{-}$OTf [M]	$R_{\alpha \rightarrow OTf, EXSY}$ [s^{-1}]	$R_{ax \rightarrow eq, EXSY}$ [s^{-1}]	$R_{eq \rightarrow ax, EXSY}$ [s^{-1}]
0.0456	0.376 ± 0.007	10.7 ± 0.8	24.1 ± 3.4
0.0843	0.465 ± 0.01	10.8 ± 1.0	26.5 ± 1.0
0.145	0.520 ± 0.02	11.2 ± 0.9	30.7 ± 1.3
0.223	-	11.9 ± 1.0	27.2 ± 1.9

Table S4: Supporting rates Figure 6.

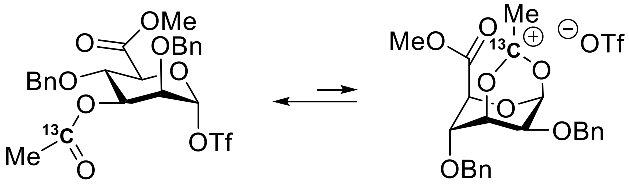

Conc. $^{-}$OTf [M]	$R_{\alpha \rightarrow d, CEST}$ [s^{-1}]
0.0456	0.261 ± 0.03
0.0843	0.202 ± 0.01
0.145	0.261 ± 0.03
0.223	0.230 ± 0.04

The linear range between 0.1 and 1 seconds saturation time was used to determine $R_{\alpha \rightarrow d, CEST}$.

References

1. Serianni, A. S.; Pierce, J.; Huang, S. G.; Barker, R., Anomerization of furanose sugars: kinetics of ring-opening reactions by proton and carbon-13 saturation-transfer NMR spectroscopy. *Journal of the American Chemical Society* **1982,** *104* (15), 4037-4044.
2. McConnell, H. M., Reaction rates by nuclear magnetic resonance. *The Journal of chemical physics* **1958,** *28* (3), 430-431.
3. Martens, J.; Berden, G.; Gebhardt, C. R.; Oomens, J., Infrared ion spectroscopy in a modified quadrupole ion trap mass spectrometer at the FELIX free electron laser laboratory. *Review of Scientific Instruments* **2016,** *87* (10).
4. Berden, G.; Derksen, M.; Houthuijs, K. J.; Martens, J.; Oomens, J., An automatic variable laser attenuator for IRMPD spectroscopy and analysis of power-dependence in fragmentation spectra. *International Journal of Mass Spectrometry* **2019,** *443*, 1-8.
5. Hansen, T.; Elferink, H.; van Hengst, J. M.; Houthuijs, K. J.; Remmerswaal, W. A.; Kromm, A.; Berden, G.; van der Vorm, S.; Rijs, A. M.; Overkleeft, H. S.; Codée, J. D.; Boltje, T. J., Characterization of glycosyl dioxolenium ions and their role in glycosylation reactions. *Nature Communications* **2020,** *11* (1), 2664.
6. van Outersterp, R. E.; Houthuijs, K. J.; Berden, G.; Engelke, U. F.; Kluijtmans, L. A.; Wevers, R. A.; Coene, K. L.; Oomens, J.; Martens, J., Reference-standard free metabolite identification using infrared ion spectroscopy. *International Journal of Mass Spectrometry* **2019,** *443*, 77-85.
7. M.J. Frisch, G. W. T., H.B. Schlegel, G.E. Scuseria, M.A. Robb, J.R. Cheeseman, G. Scalmani, V. Barone, G. A. Petersson, H. Nakatsuji, X. Li, M. Caricato, A.V. Marenich, J. Bloino, B.G. Janesko, R. Gomperts, B. Mennucci, H.P. Hratchian, J.V. Ortiz, A.F. Izmaylov, J.L. Sonnenberg, D. Williams-Young, F. Ding, F. Lipparini, F. Egidi, J. Goings, B. Peng, A. Petrone, T. Henderson, D. Ranasinghe, V.G. Zakrzewski, J. Gao, N. Rega, G. Zheng, W. Liang, M. Hada, M. Ehara, K. Toyota, R. Fukuda, J. Hasegawa, M. Ishida, T. Nakajima, Y. Honda, O. Kitao, H. Nakai, T. Vreven, K. Throssell, J.A. Montgomery, Jr., J.E. Peralta, F. Ogliaro, M.J. Bearpark, J.J. Heyd, E.N. Brothers, K.N. Kudin, V.N. Staroverov, T.A. Keith, R. Kobayashi, J. Normand, K. Raghavachari, A.P. Rendell, J.C. Burant, S.S. Iyengar, J. Tomasi, M. Cossi, J.M. Millam, M. Klene, C. Adamo, R. Cammi, J.W. Ochterski, R.L. Martin, K. Morokuma, O. Farkas, J.B. Foresman, and D.J. Fox, 1, mj frisch, gw trucks, hb schlegel, ge scuseria, ma robb, jr cheeseman, g. Scalmani, v. Barone, b. Mennucci, ga petersson et al., gaussian. *Inc., Wallingford CT* **2009,** *121*, 150-166.
8. de Kleijne, F. F.; Elferink, H.; Moons, S. J.; White, P. B.; Boltje, T. J., Characterization of Mannosyl Dioxanium Ions in Solution Using Chemical Exchange Saturation Transfer NMR Spectroscopy. *Angewandte Chemie International Edition* **2022,** *61* (6), e202109874.
9. Moons, P. H.; Ter Braak, F.; de Kleijne, F. F.; Bijleveld, B.; Corver, S. J.; Houthuijs, K. J.; Almizori, H. R.; Berden, G.; Martens, J.; Oomens, J.; White, P. B.; Boltje, T. J., Characterization of elusive rhamnosyl dioxanium ions and their application in complex oligosaccharide synthesis. *Nature Communications* **2024,** *15* (1), 2257.
10. Elferink, H.; Severijnen, M. E.; Martens, J.; Mensink, R. A.; Berden, G.; Oomens, J.; Rutjes, F. P.; Rijs, A. M.; Boltje, T. J., Direct experimental characterization of glycosyl cations by infrared ion spectroscopy. *Journal of the American Chemical Society* **2018,** *140* (19), 6034-6038.

Data

Kinetic data - Temperature

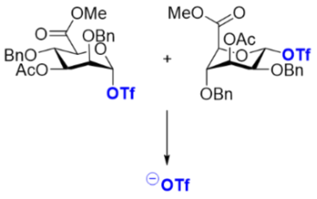

Spectrum number	Mixing Time [s]	Equatorial α-triflate resonance intensity	Axial α-triflate resonance intensity	⁻OTf resonance intensity
1	0.03	1.52051	3.70793	0.044737
2	0.06	1.3847	3.29292	0.077445
3	0.09	1.24449	2.96791	0.106492
4	0.12	1.13445	2.66579	0.128569
5	0.15	1.02209	2.41562	0.149615
6	0.18	0.921873	2.18137	0.165797
7	0.21	0.836154	1.96059	0.17868
8	0.24	0.761809	1.79404	0.182187
9	0.27	0.684122	1.6148	0.197205
10	0.3	0.619335	1.46213	0.200561

Temperature = -80 °C

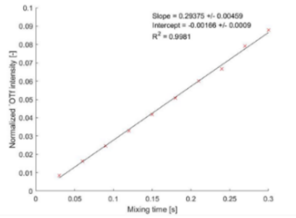

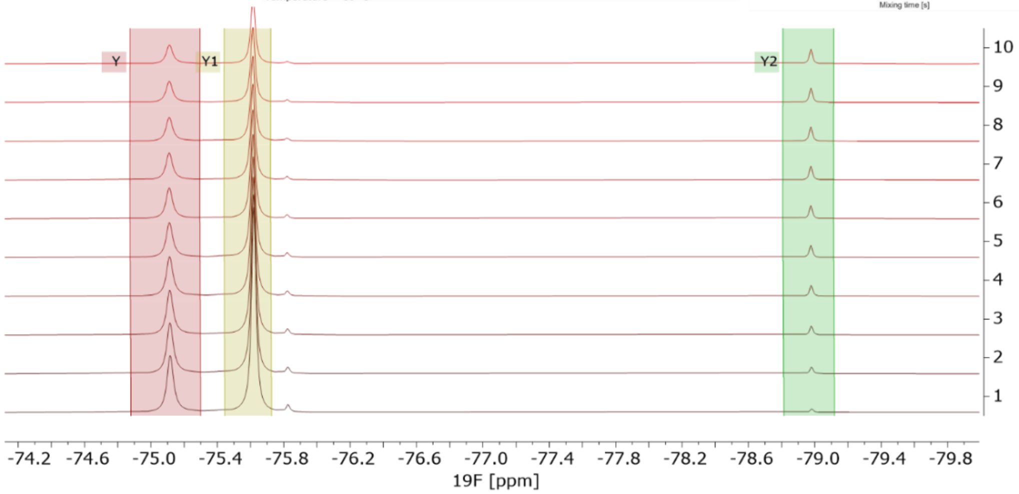

Figure S42: ^{19}F EXSY data.

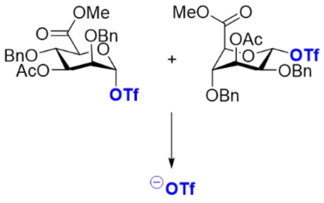

Spectrum number	Mixing Time [s]	Equatorial α-triflate resonance intensity	Axial α-triflate resonance intensity	⁻OTf resonance intensity
1	0.01	0.922021	2.16843	0.027876
2	0.02	0.947966	2.02341	0.056575
3	0.03	0.908528	1.93857	0.083138
4	0.04	0.883128	1.86149	0.108573
5	0.05	0.84258	1.79721	0.133032
6	0.06	0.821093	1.71112	0.152823
7	0.07	0.792103	1.65395	0.173427
8	0.08	0.740756	1.5905	0.19672
9	0.09	0.720655	1.52389	0.211944
10	0.1	0.68986	1.47303	0.228941

Temperature = -70 °C

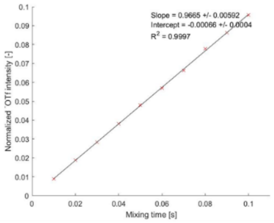

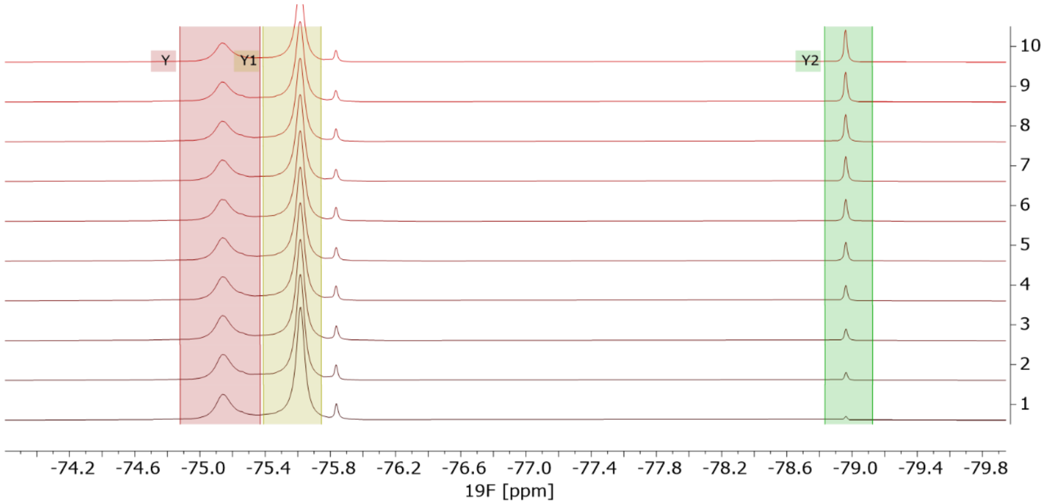

Figure S43: ^{19}F EXSY data.

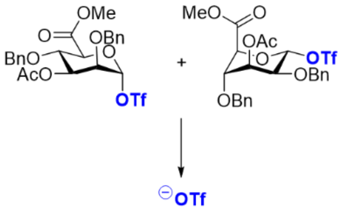

Spectrum number	Mixing Time [s]	Merged α-triflate resonances intensity	⁻OTf resonance intensity
1	0.0023	0.357402	0.006598
2	0.0046	0.355447	0.017489
3	0.0069	0.348058	0.018885
4	0.0092	0.351077	0.02771
5	0.0115	0.338809	0.035946
6	0.0138	0.335542	0.038868
7	0.0161	0.324205	0.043078
8	0.0184	0.327711	0.048573
9	0.0207	0.318122	0.051301
10	0.023	0.309491	0.056947

Temperature = -50 °C

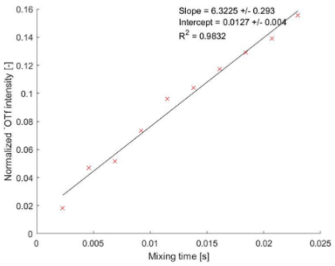

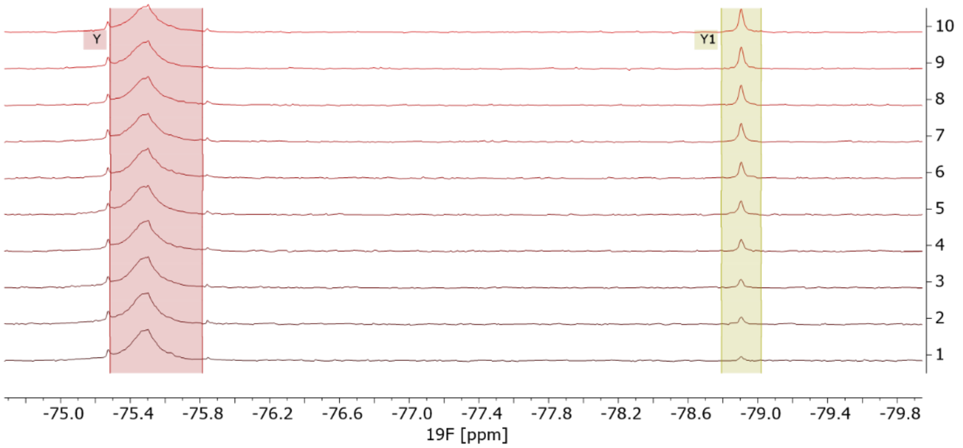

Figure S44: ^{19}F EXSY data.

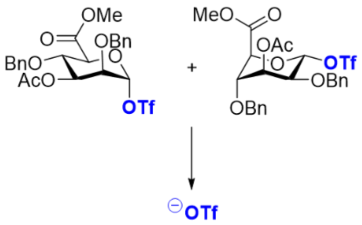

Spectrum number	Mixing Time [s]	Merged α-triflate resonances intensity	⁻OTf resonance intensity
1	0.001	5.13702	0.127596
2	0.002	4.8446	0.243686
3	0.003	4.78619	0.352252
4	0.004	4.70458	0.456825
5	0.005	4.4969	0.560015
6	0.006	4.50437	0.650922
7	0.007	4.31103	0.742958
8	0.008	4.34417	0.822753
9	0.009	4.12553	0.904047
10	0.01	4.11444	0.981682

Temperature = -40 °C

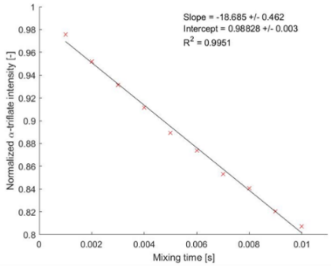

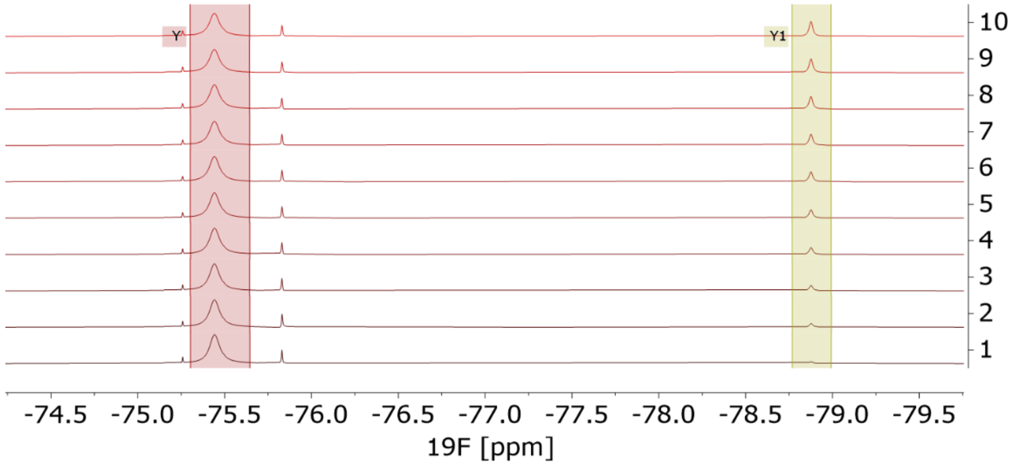

Figure S45: ^{19}F EXSY data.

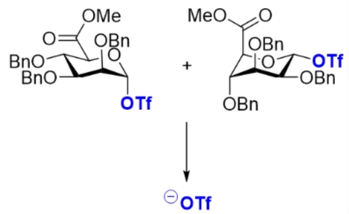

Spectrum number	Mixing Time [s]	Equatorial α-triflate resonance intensity	Axial α-triflate resonance intensity	$^{-}$OTf (-78.99 ppm) resonance intensity
1	0.08	7676.95	9765.02	211.6
2	0.16	6420.61	7768.91	388.39
3	0.24	5302.94	6793.99	377.49
4	0.32	3767.58	5924.99	516.88
5	0.4	3563.81	5159.47	510.11
6	0.48	3138.46	4044.44	517.71
7	0.56	2130.75	3430.2	497.91

Temperature = -50 °C

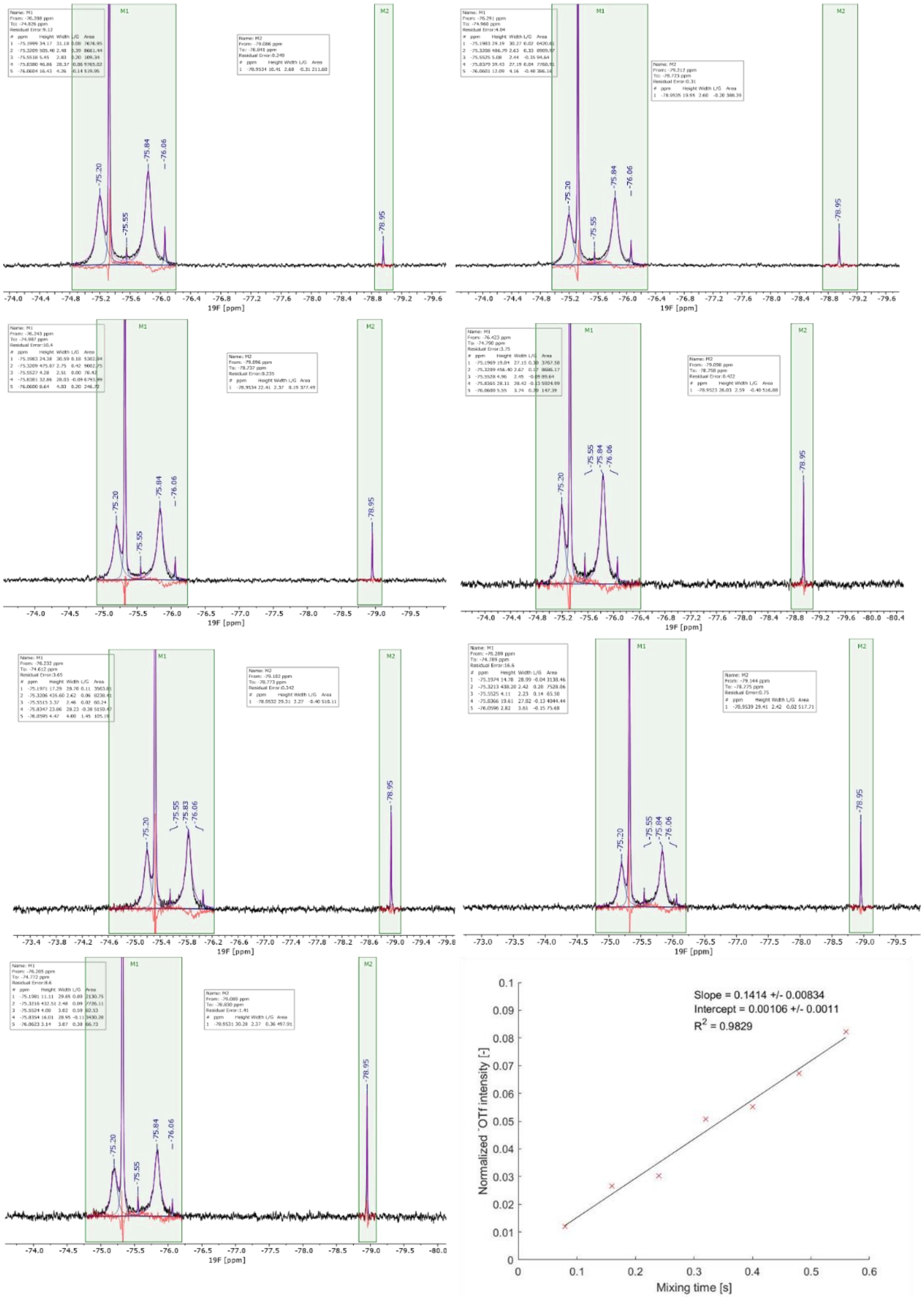

Figure S46: ^{19}F EXSY data.

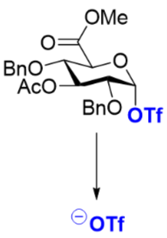

Spectrum number	Mixing Time [s]	α-triflate (-75.64 ppm) resonance intensity	⁻OTf (-78.93 ppm) resonance intensity
1	0.05	7.13463	0.06149
2	0.1	6.23724	0.109039
3	0.15	5.43504	0.148648
4	0.2	4.75721	0.182881
5	0.25	4.1218	0.20846
6	0.3	3.58064	0.224771
7	0.35	3.08464	0.24384
8	0.4	2.66909	0.250463
9	0.45	2.30622	0.258918
10	0.5	2.00231	0.25967

Temperature = -30 °C

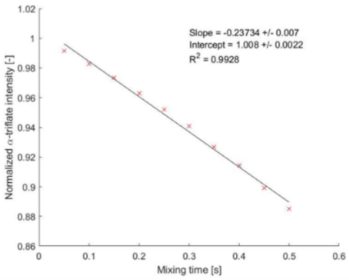

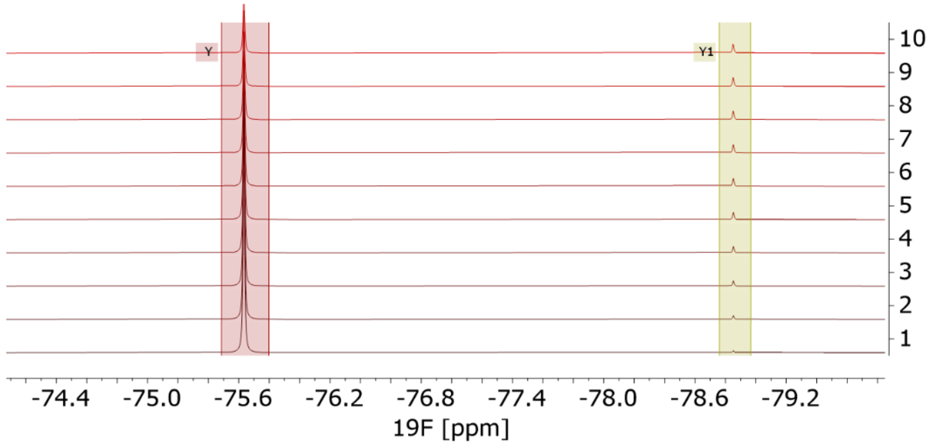

Figure S47: [19]F EXSY data.

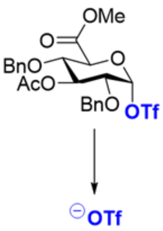

Spectrum number	Mixing Time [s]	α-triflate (-75.64 ppm) resonance intensity	⁻OTf (-78.93 ppm) resonance intensity
1	0.15	8.97677	0.048947
2	0.3	6.24135	0.075544
3	0.45	4.36199	0.085604
4	0.6	3.02852	0.088606
5	0.75	2.12172	0.08447
6	0.9	1.49476	0.082417
7	1.05	1.0481	0.072047
8	1.2	0.739729	0.065022
9	1.35	0.523901	0.060473
10	1.5	0.370873	0.0533

Temperature = -40 °C

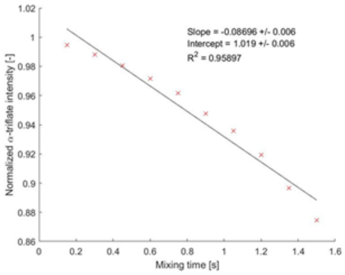

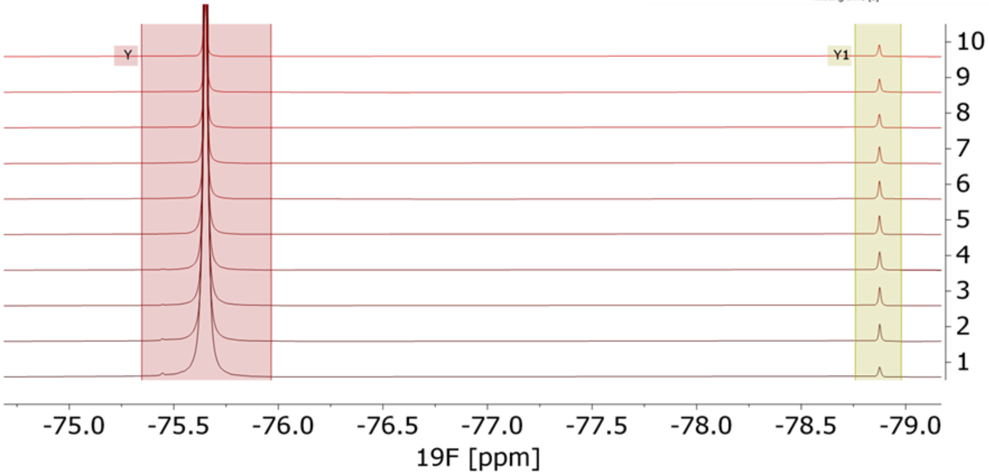

Figure S48: [19]F EXSY data.

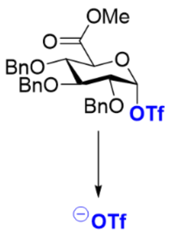

Spectrum number	Mixing Time [s]	α-triflate (-75.64 ppm) resonance intensity	$^{-}$OTf (-78.93 ppm) resonance intensity
1	0.1	7.54538	0.037068
2	0.2	5.68127	0.0598
3	0.3	4.30144	0.07227
4	0.4	3.22803	0.078843
5	0.5	2.44367	0.084884
6	0.6	1.85956	0.081595
7	0.7	1.40481	0.077368
8	0.8	1.06568	0.076312
9	0.9	0.813956	0.067124
10	1	0.620667	0.062807

Temperature = -50 °C

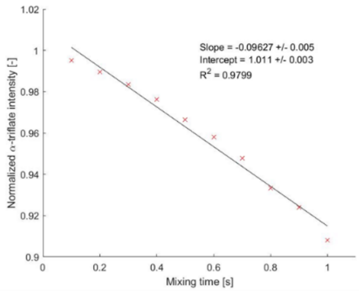

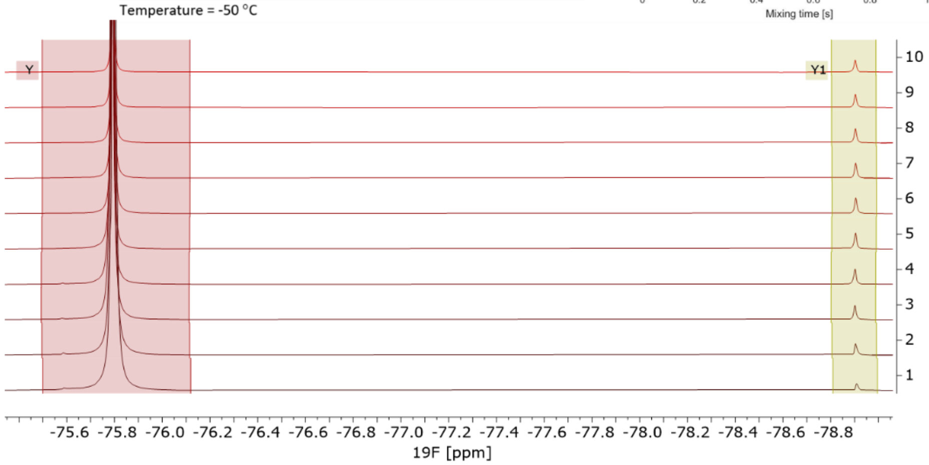

Figure S49: ^{19}F EXSY data.

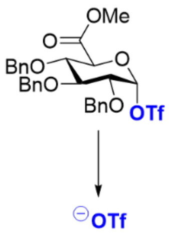

Spectrum number	Mixing Time [s]	α-triflate (-75.64 ppm) resonance intensity	$^{-}$OTf (-78.93 ppm) resonance intensity
1	0.1	7.54538	0.037068
2	0.2	5.68127	0.0598
3	0.3	4.30144	0.07227
4	0.4	3.22803	0.078843
5	0.5	2.44367	0.084884
6	0.6	1.85956	0.081595
7	0.7	1.40481	0.077368
8	0.8	1.06568	0.076312
9	0.9	0.813956	0.067124
10	1	0.620667	0.062807

Temperature = -50 °C

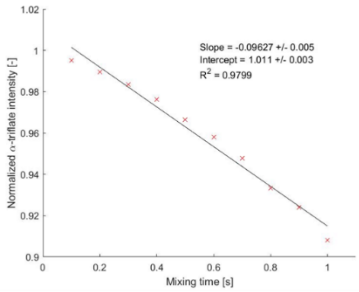

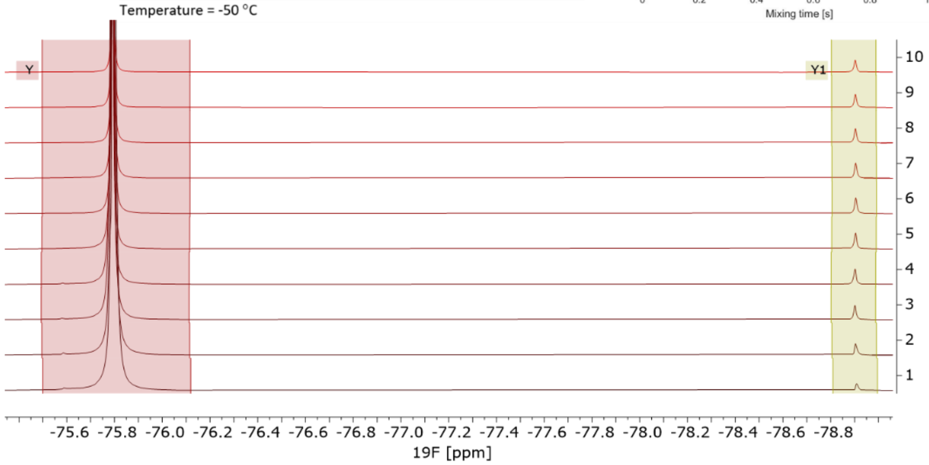

Figure S50: ^{19}F EXSY data.

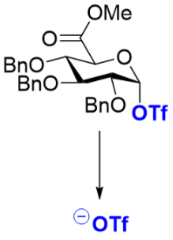

Spectrum number	Mixing Time [s]	α-triflate (-75.64 ppm) resonance intensity	⁻OTf (-78.93 ppm) resonance intensity
1	0.015	1.21114	0.014015
2	0.03	1.12769	0.028024
3	0.045	0.997258	0.031594
4	0.06	0.904386	0.036745
5	0.075	0.837854	0.040979
6	0.09	0.761313	0.044361
7	0.105	0.689298	0.051768
8	0.12	0.632521	0.05558
9	0.135	0.581841	0.059918
10	0.15	0.533626	0.059695

Temperature = -30 °C

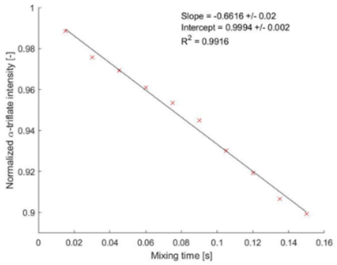

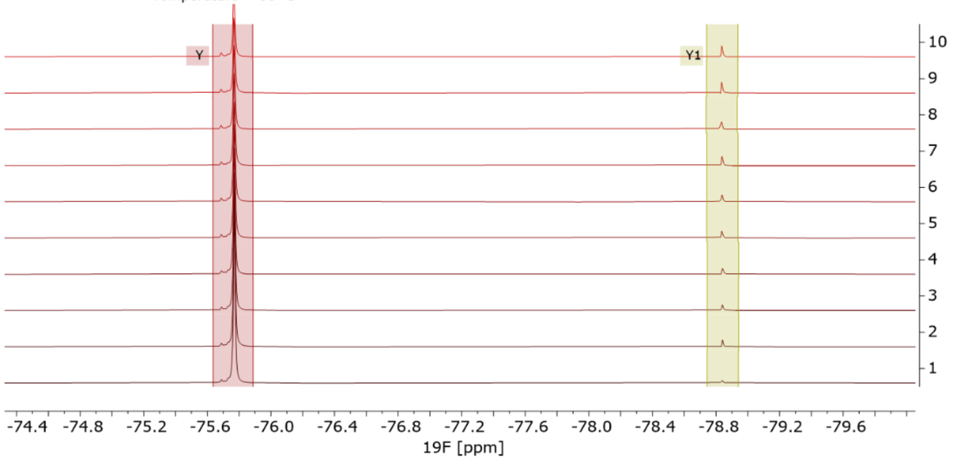

Figure S51: ^{19}F EXSY data.

Kinetic data – Tetrabutyl ammonium triflate

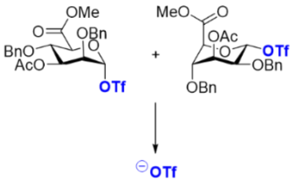

Spectrum number	Mixing Time [s]	Axial α-triflate resonance intensity	Equatorial α-triflate resonance intensity	⁻OTf resonance intensity
1	0.025	4264.73	10610.6	60.5838
2	0.05	4087.58	9725.72	222.986
3	0.075	3860.13	8988.56	303.316
4	0.1	3545.38	8304.44	449.57
5	0.125	3286.7	7609.53	498.888
6	0.15	3022.99	7036.8	567.439
7	0.175	2823.62	6518.96	596.674
8	0.2	2607.77	6071.37	657.395
9	0.225	2385.98	5565.3	700.172
10	0.25	2265.53	5141.97	738.469

Temperature = -80 °C
[OTf] = 45 mM

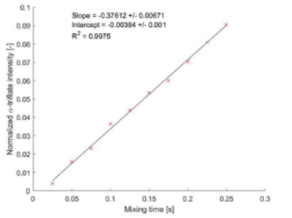

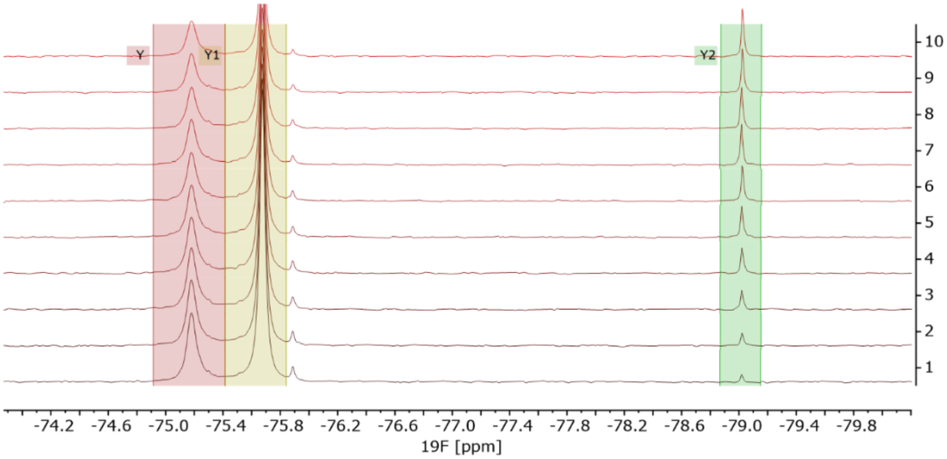

Figure S52: ^{19}F EXSY data.

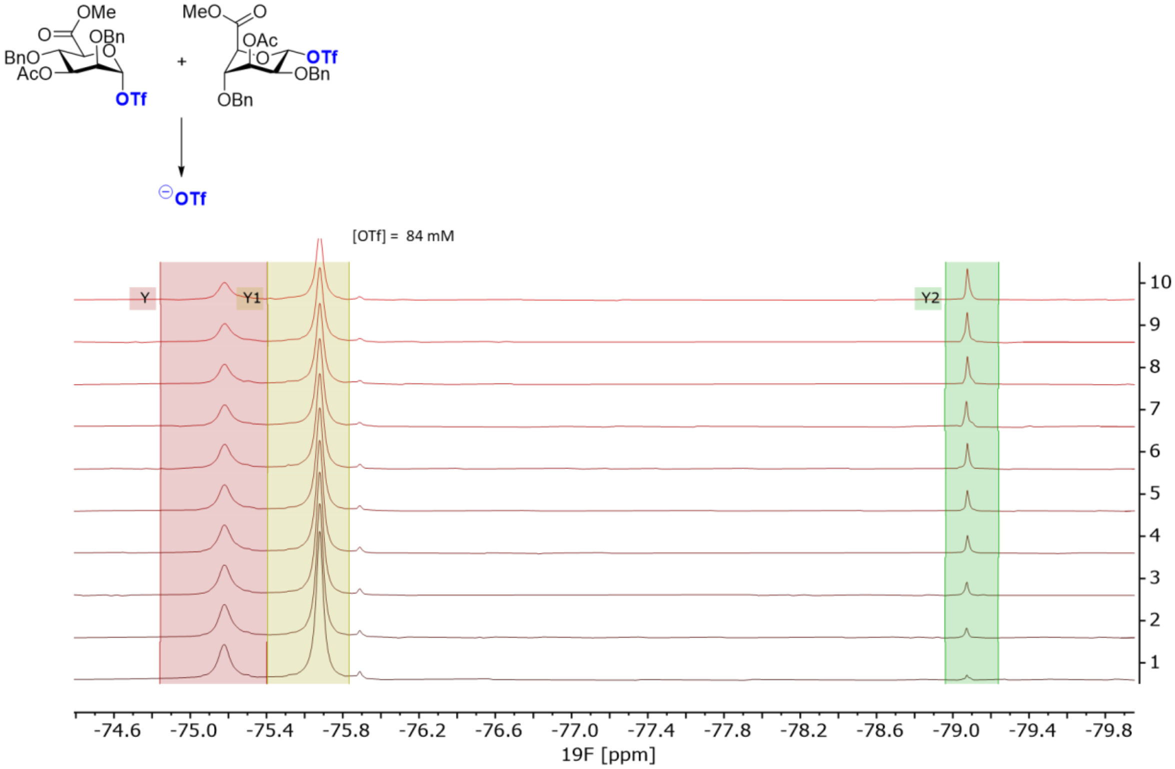

Figure S53: ^{19}F EXSY data.

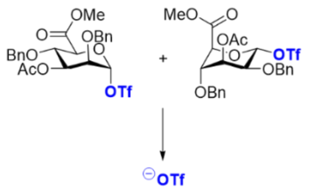

Spectrum number	Mixing Time [s]	Axial α-triflate resonance intensity	Equatorial α-triflate resonance intensity	⁻OTf resonance intensity
1	0.025	2680.83	6897.03	113.337
2	0.05	2615.16	6143.1	208.298
3	0.075	2330.45	5664.47	324.604
4	0.1	2143.65	5166.78	363.056
5	0.125	1854.29	4664.56	451.442
6	0.15	1792.48	4331.88	443.624
7	0.175	1617.87	3944.48	517.413
8	0.2	1683.72	3684.24	602.163
9	0.225	1488.95	3430.37	662.087
10	0.25	1286.69	3121.31	652.8

Temperature = -80 °C
[OTf] = 145 mM

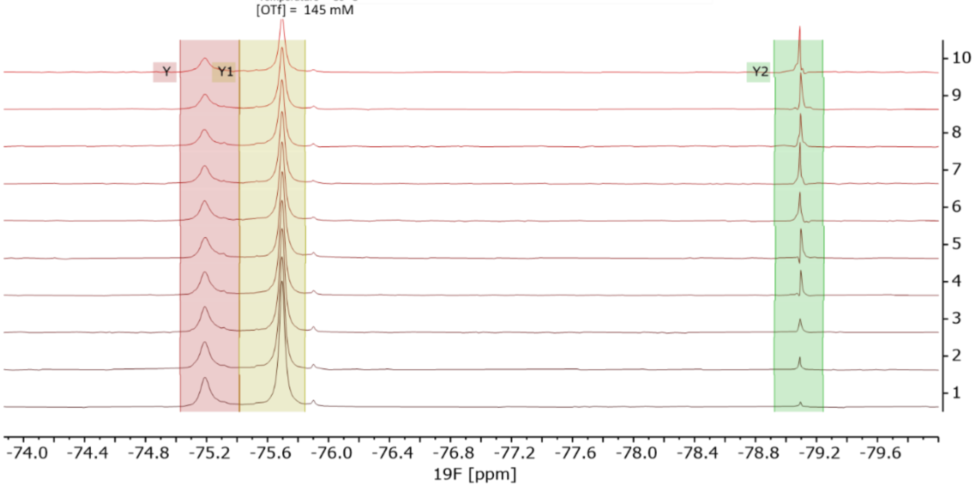

Figure S54: ^{19}F EXSY data.

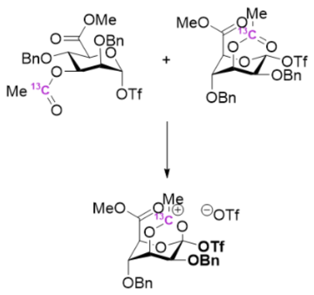

Saturation time [s]	Carbonyl intensity [-]
0	2214.17
0.1	2150.86
0.2	2128.13
0.3	2094.31
0.4	2044.34
0.5	2032.61
0.6	2035.6
0.7	1893.08
0.8	1945.52
0.9	1872.38
1	1857.92
5	1657.15

Temperature = -80 °C
[OTf] = 46 mM

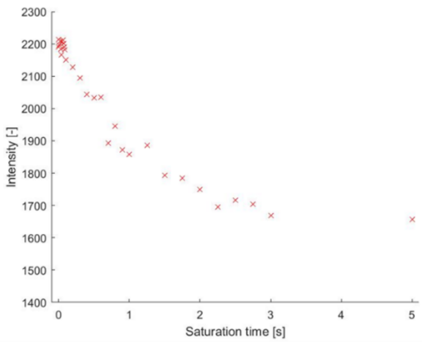

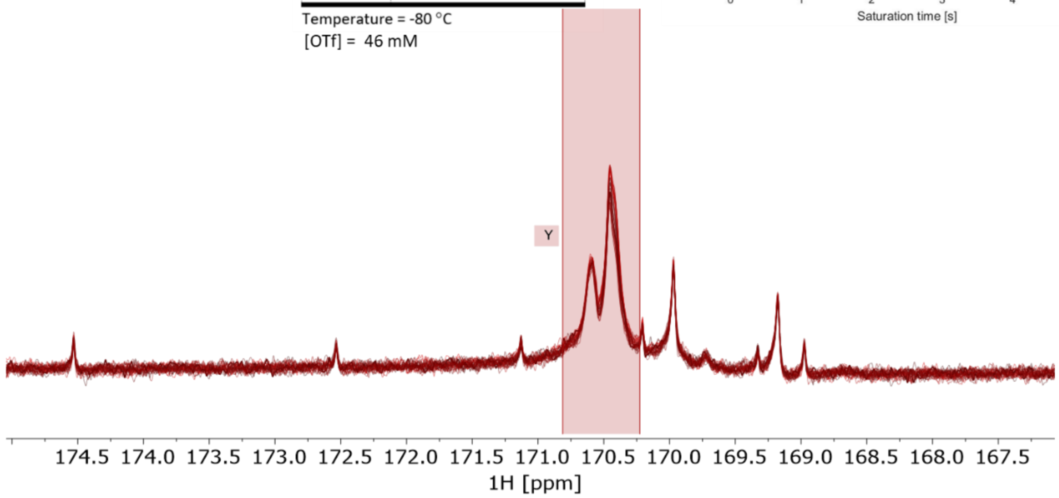

Figure S55: ^{13}C CEST data.

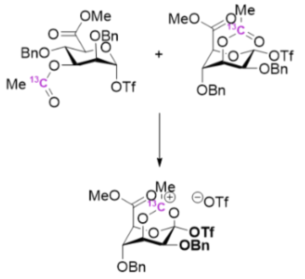

Saturation time [s]	Carbonyl intensity [-]
0	3414.77
0.1	3492.17
0.2	3443.18
0.3	3290.08
0.4	3291.24
0.5	3183.71
0.6	3193.98
0.7	3148.21
0.8	3084.37
0.9	3062.34
1	3053.71
5	2767.88

Temperature = -80 °C
[OTf] = 84 mM

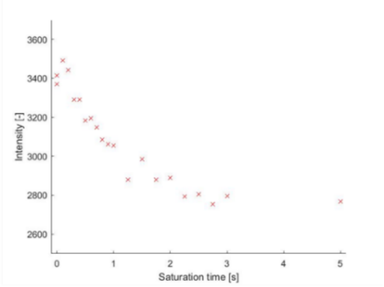

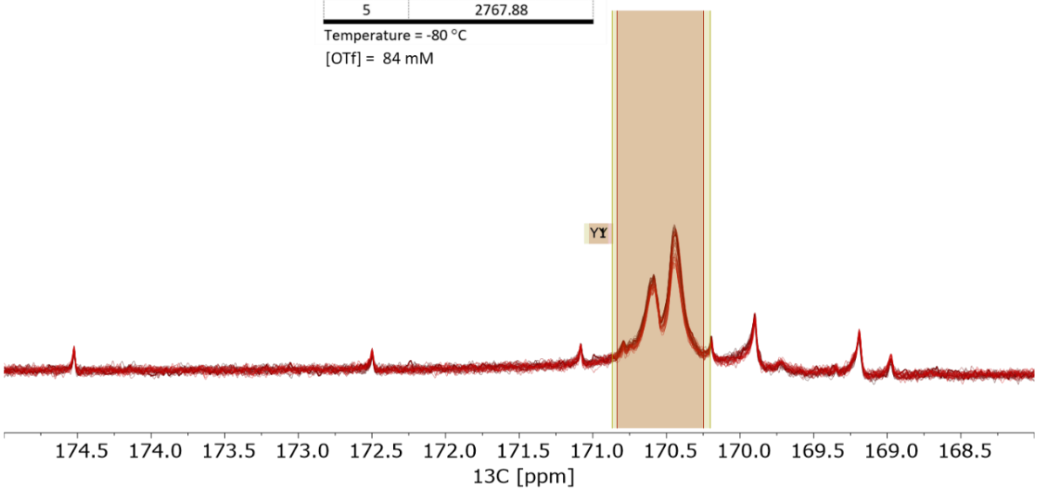

Figure S56: ^{13}C CEST data.

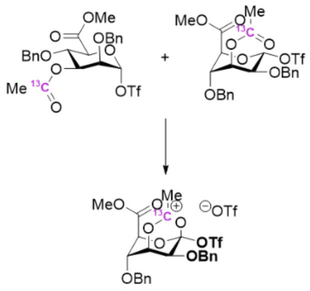

Saturation time [s]	Carbonyl intensity [-]
0	3413.02
0.1	3293.18
0.2	3152.51
0.3	3142.35
0.4	3033.61
0.5	2992.81
0.6	2941.64
0.7	2999.46
0.8	2904.99
0.9	2819.86
1	2881.81
5	2611.72

Temperature = -80 °C
[OTf] = 145 mM

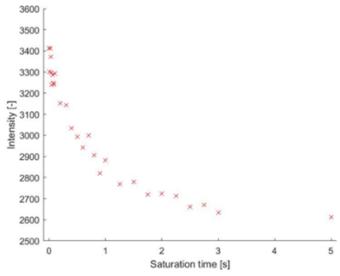

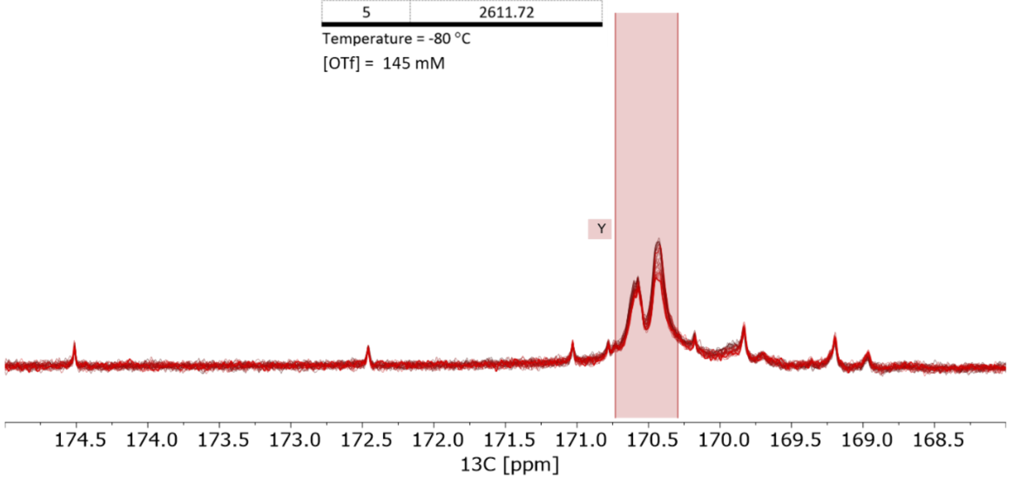

Figure S57: ^{13}C CEST data.

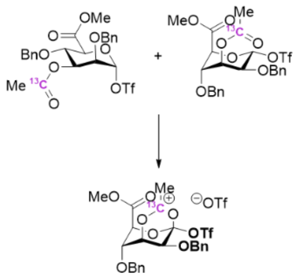

Saturation time [s]	Carbonyl intensity [-]
0	3097.09
0.1	3022.83
0.2	2943.12
0.3	2981.9
0.4	2784.95
0.5	2901.95
0.6	2841.89
0.7	2752.41
0.8	2722.08
0.9	2750.81
1	2674.1
5	2540.26

Temperature = -80 °C
[OTf] = 223 mM

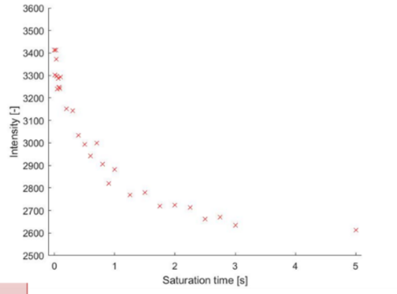

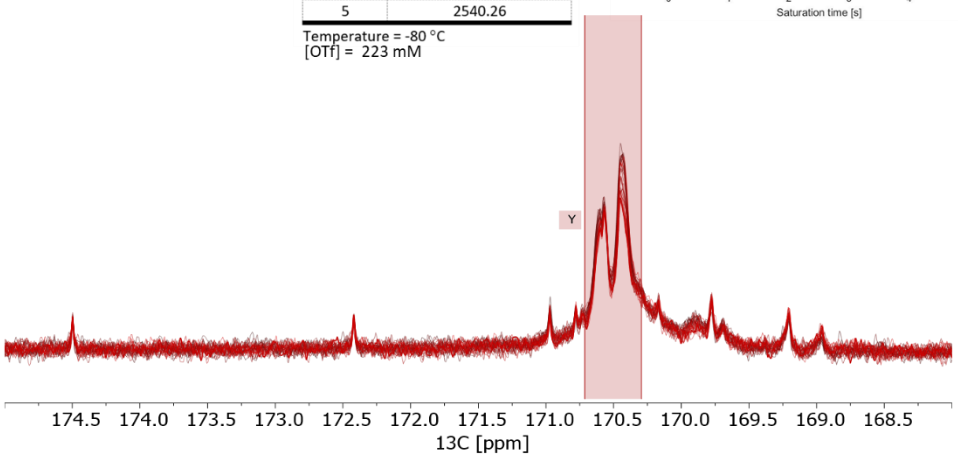

Figure S58: ^{13}C CEST data.

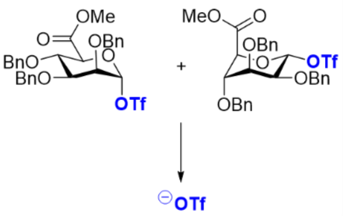

Spectrum number	Mixing Time [s]	Axial α-triflate resonance intensity	Equatorial α-triflate resonance intensity	$^-$OTf resonance intensity
1	0.05	7497.69	9113.99	72.1546
2	0.1	6575.02	7961.96	138.346
3	0.15	5861.78	6960.84	148.436
4	0.2	5059.66	6186.13	202.115
5	0.25	4676.62	5304.44	178.603
6	0.3	4025.73	4730.65	198.627
7	0.35	3611.87	4158.89	214.475
8	0.4	3109.26	3608.81	236.812
9	0.45	2817.62	3204.91	238.945
10	0.5	2498.84	2827.35	259.973

Temperature = -60 °C [OTf] = 66 mM

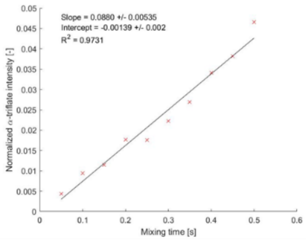

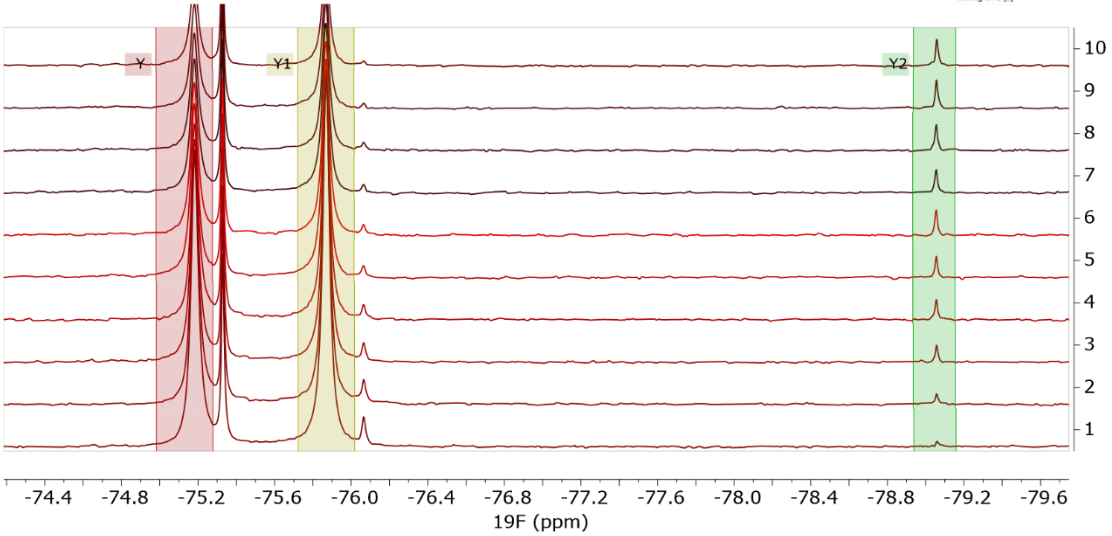

Figure S59: ^{19}F EXSY data.

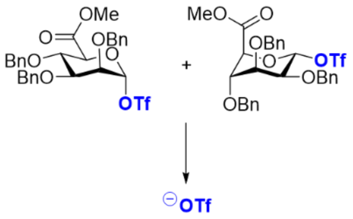

Spectrum number	Mixing Time [s]	Axial α-triflate resonance intensity	Equatorial α-triflate resonance intensity	⁻OTf resonance intensity
1	0.06	5287.19	6259.19	1.57187
2	0.12	4687.54	5187.35	183.636
3	0.18	3993.7	4410.23	207.207
4	0.24	3289.53	3768.77	245.622
5	0.3	2944.58	3255.8	269.803
6	0.36	2451.69	2704.86	294.613
7	0.42	2075.2	2326.41	336.116
8	0.48	1926.08	2065.31	267.701
9	0.54	1611.83	1684.2	270.299
10	0.6	1360.16	1397.65	297.365

Temperature = -60 °C [OTf] = 123 mM

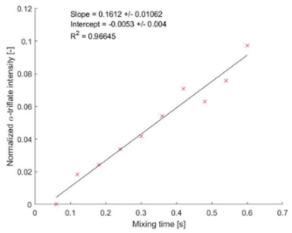

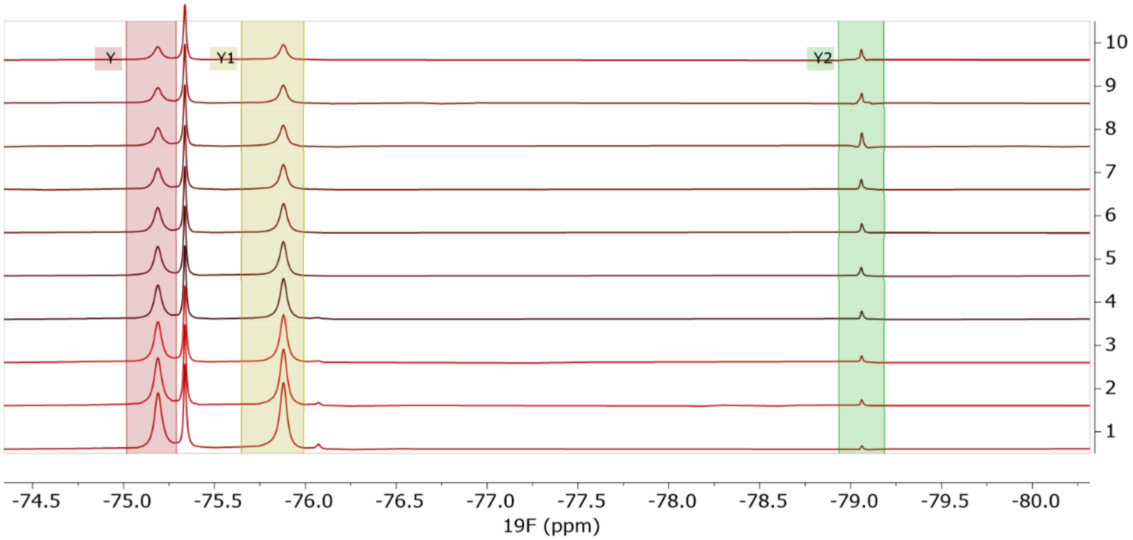

Figure S60: ^{19}F EXSY data.

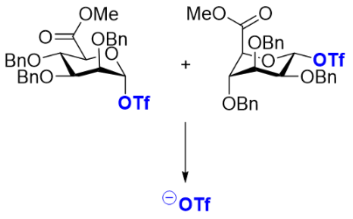

Spectrum number	Mixing Time [s]	Axial α-triflate resonance intensity	Equatorial α-triflate resonance intensity	⁻OTf resonance intensity
1	0.03	4464.76	4949.56	62.121
2	0.06	4191.27	4558.63	83.5328
3	0.09	3838.28	4210.24	94.5102
4	0.12	3703.36	3831.96	195.928
5	0.15	3229.24	3555.42	183.094
6	0.18	2976.6	3234.29	224.599
7	0.21	2765.91	2997.33	209.143
8	0.24	2578.95	2708.76	259.165
9	0.27	2321.85	2514.3	238.628
10	0.3	2278.76	2285.78	267.936

Temperature = -60 °C [OTf] = 164 mM

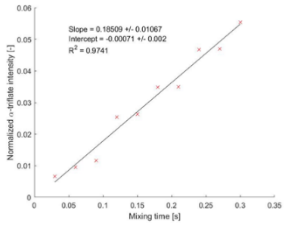

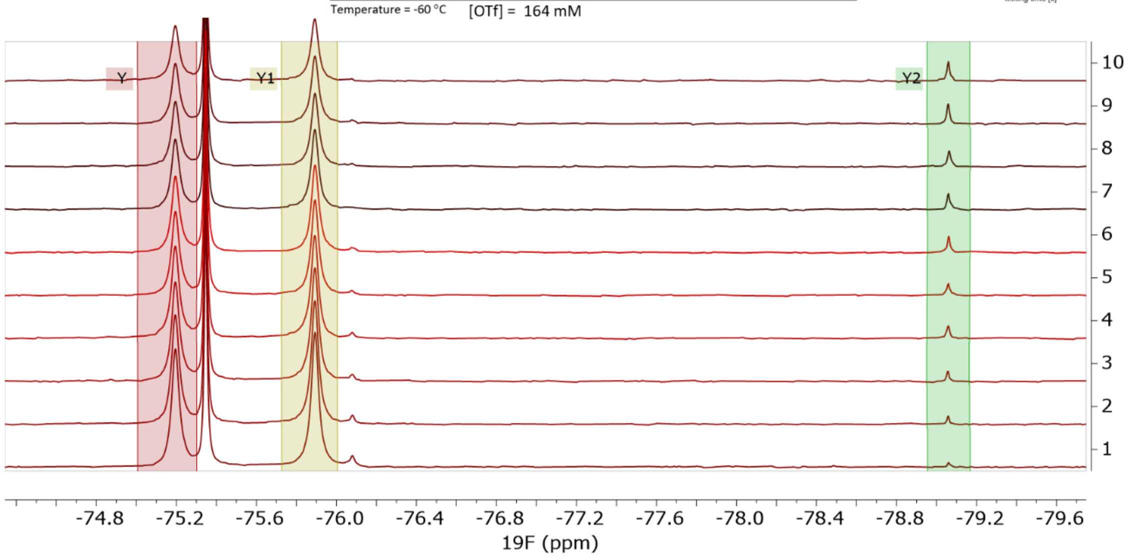

Figure S61: ^{19}F EXSY data.

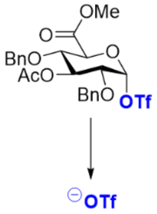

Spectrum number	Mixing Time [s]	α-triflate (-75.72 ppm) resonance intensity	⁻OTf (-78.93 ppm) resonance intensity
1	0.12	39138	254.596
2	0.24	31716.9	484.694
3	0.36	25767.8	638.279
4	0.48	21041.6	722.396
5	0.6	17222.7	785.92
6	0.72	14135.6	784.449
7	0.84	11632.2	817.314
8	0.96	9523.82	808.514
9	1.08	7851.23	802.94
10	1.2	6560.22	828.243

Temperature = -40 °C [OTf] = 34 mM

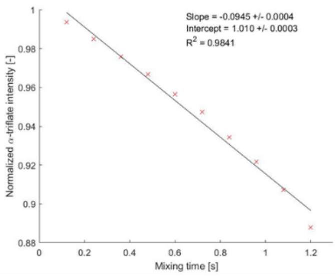

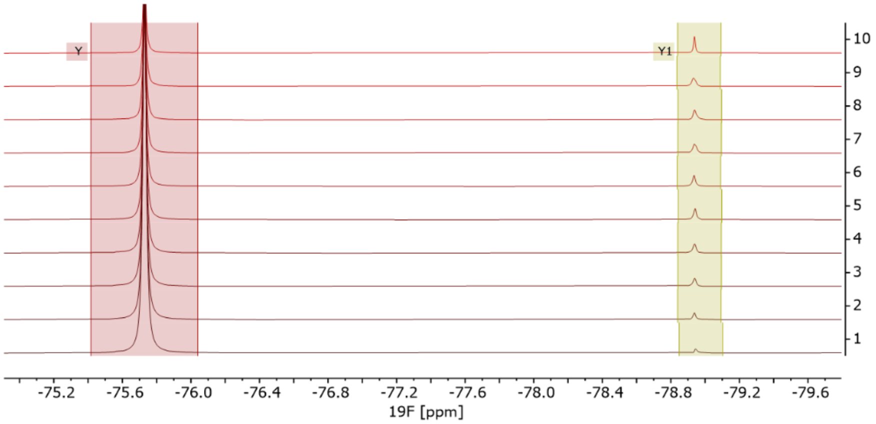

Figure S62: ^{19}F EXSY data.

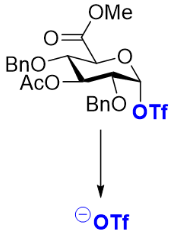

Spectrum number	Mixing Time [s]	α-triflate (-75.72 ppm) resonance intensity	⁻OTf (-78.93 ppm) resonance intensity
1	0.05	27888	230.986
2	0.1	24692	440.563
3	0.15	22396.1	627.902
4	0.2	20082.5	829.828
5	0.25	18166.4	950.83
6	0.3	16336	1052.04
7	0.35	14432.9	1123.57
8	0.4	13410.2	1261.45
9	0.45	12183.1	1335.95
10	0.5	11009.6	1353.86

Temperature = -40 °C [OTf] = 82 mM

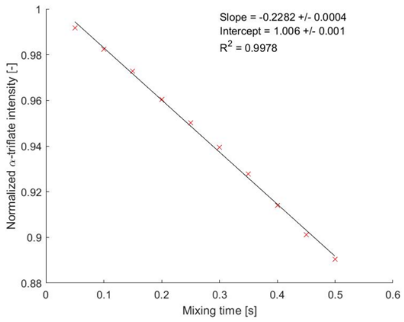

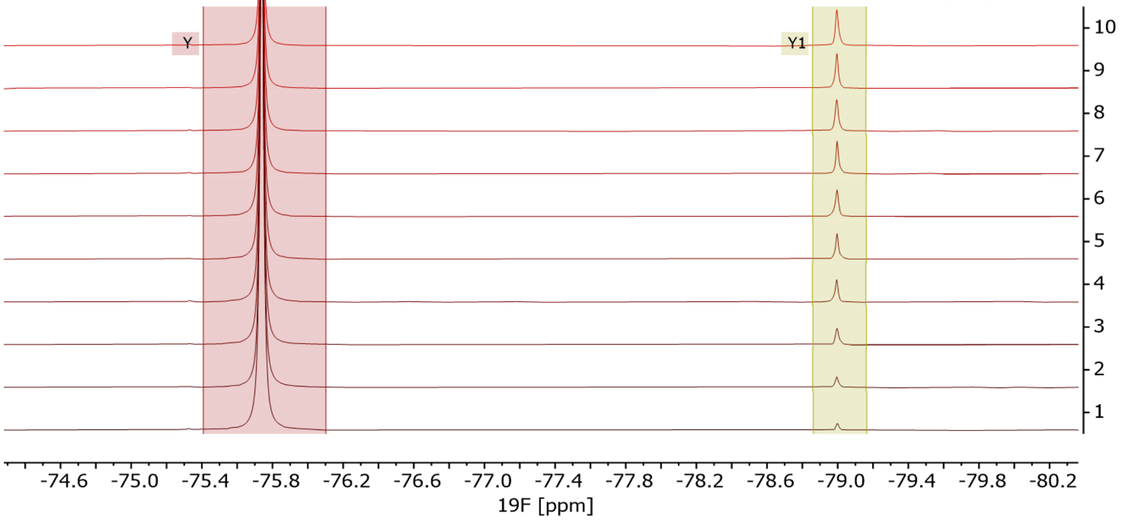

Figure S63: ^{19}F EXSY data.

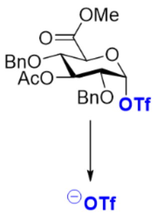

Spectrum number	Mixing Time [s]	α-triflate (-75.72 ppm) resonance intensity	⁻OTf (-78.93 ppm) resonance intensity
1	0.03	18882.6	209.299
2	0.06	17337.2	375.172
3	0.09	16014.4	535.075
4	0.12	14940.4	674.613
5	0.15	13872.2	714.726
6	0.18	12948.2	848.036
7	0.21	12025.3	996.197
8	0.24	11181.9	1039.21
9	0.27	10420.7	1117.49
10	0.3	9705.95	1165.64

Temperature = -40 °C [OTf] = 135 mM

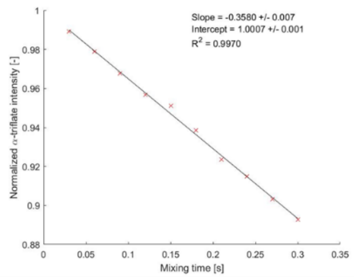

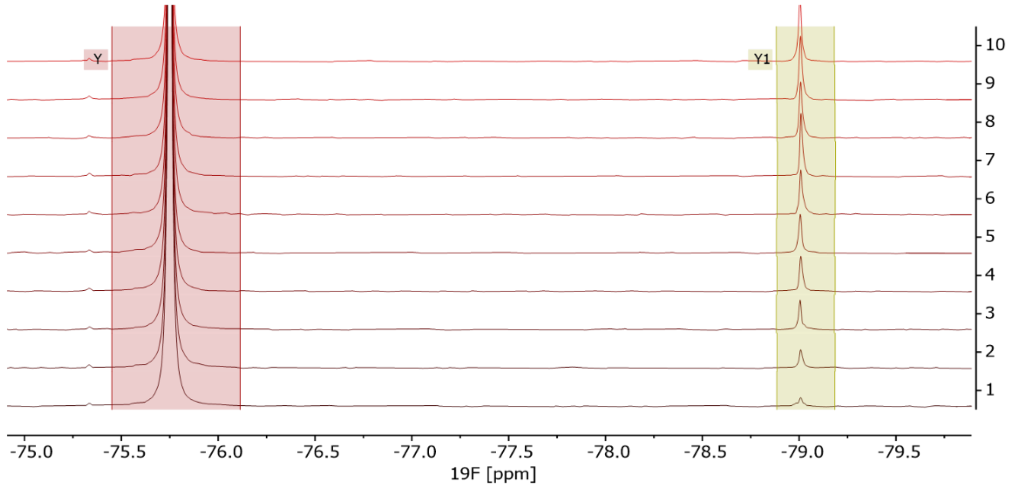

Figure S64: ^{19}F EXSY data.

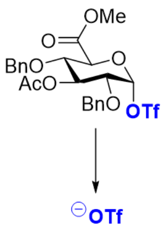

Spectrum number	Mixing Time [s]	α-triflate (-75.72 ppm) resonance intensity	⁻OTf (-78.93 ppm) resonance intensity
1	0.02	11870.6	218.416
2	0.04	11139.9	250.708
3	0.06	10370	325.178
4	0.08	9895.37	469.729
5	0.1	9255.59	619.627
6	0.12	8760.64	728.083
7	0.14	8183.48	750.784
8	0.16	7755.12	822.144
9	0.18	7393.65	864.542
10	0.2	6993.42	913.192

Temperature = -40 °C [OTf] = 217 mM

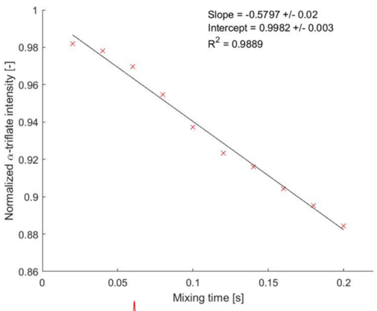

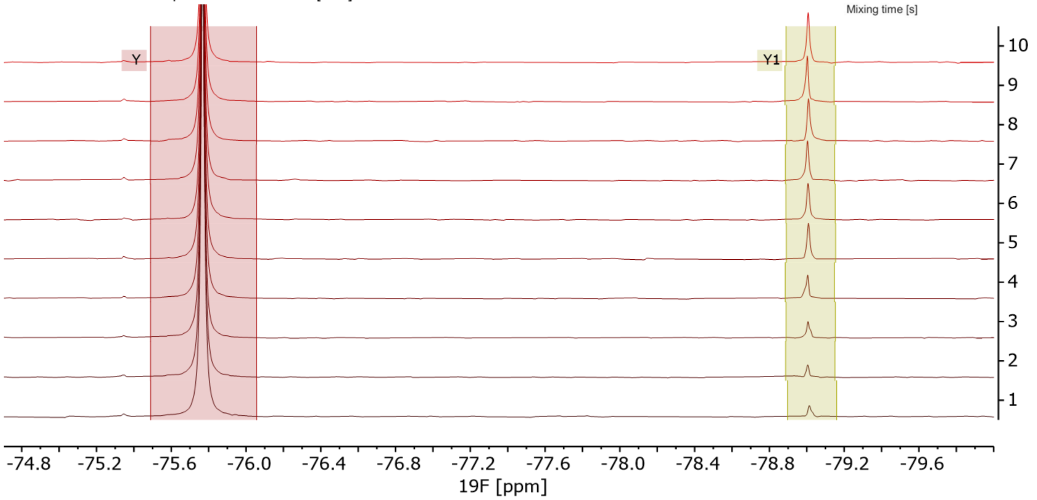

Figure S65: ^{19}F EXSY data.

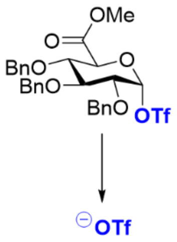

Spectrum number	Mixing Time [s]	α-triflate (-75.86 ppm) resonance intensity	-OTf (-78.99 ppm) resonance intensity
1	0.1	20585.2	140.635
2	0.2	16701.3	192.517
3	0.3	13659.9	255.037
4	0.4	11154.5	254.943
5	0.5	9205.99	340.774
6	0.6	7607.39	309.429
7	0.7	6239.11	323.977
8	0.8	5143.87	350.66
9	0.9	4316.66	352.504
10	1	3556	325.432

Temperature = -50 °C [OTf] = 26 mM

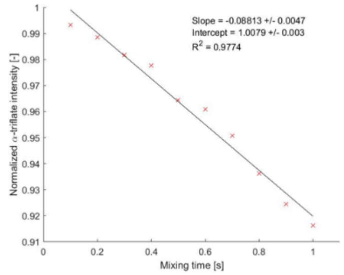

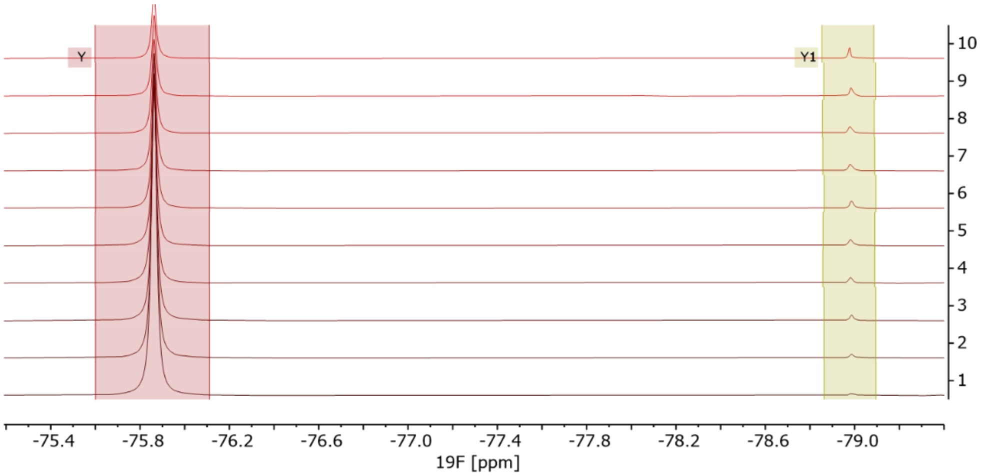

Figure S66: ^{19}F EXSY data.

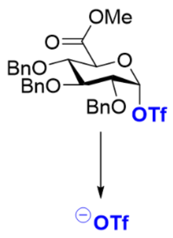

Spectrum number	Mixing Time [s]	α-triflate (-75.86 ppm) resonance intensity	-OTf (-78.99 ppm) resonance intensity
1	0.05	19463.1	121.855
2	0.1	17272	292.91
3	0.15	15422.8	394.067
4	0.2	13705	523.05
5	0.25	12212.6	555.769
6	0.3	10890.9	639.125
7	0.35	9727.5	695.214
8	0.4	8693.6	740.069
9	0.45	7795.6	776.154
10	0.5	7046.17	796.821

Temperature = -50 °C [OTf] = 63 mM

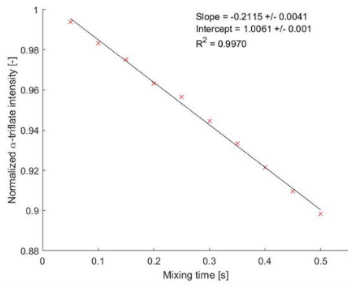

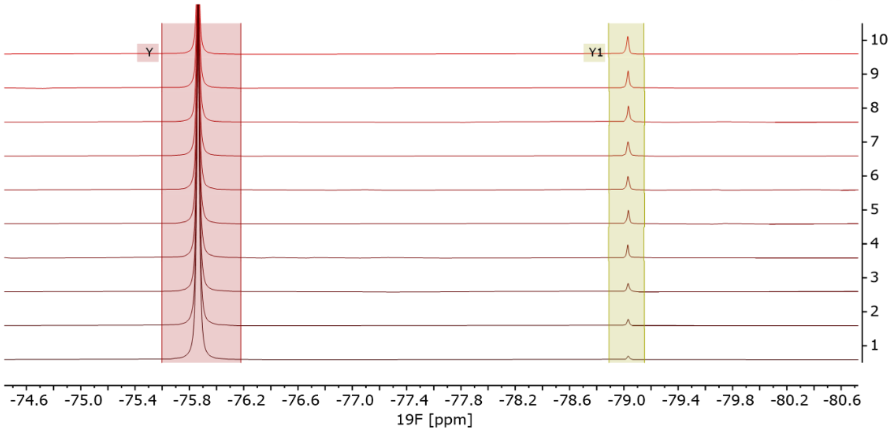

Figure S67: ^{19}F EXSY data.

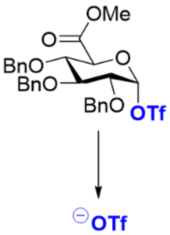

Spectrum number	Mixing Time [s]	α-triflate (-75.86 ppm) resonance intensity	⁻OTf (-78.99 ppm) resonance intensity
1	0.03	15400.6	130.893
2	0.06	14085.4	247.343
3	0.09	12885.8	471.78
4	0.12	11943.3	550.556
5	0.15	11006.7	595.626
6	0.18	10167.3	784.838
7	0.21	9452.8	807.013
8	0.24	8766.79	918.182
9	0.27	8167	945.492
10	0.3	7519.5	1019.21

Temperature = -50 °C [OTf] = 112 mM

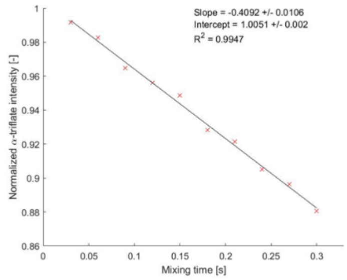

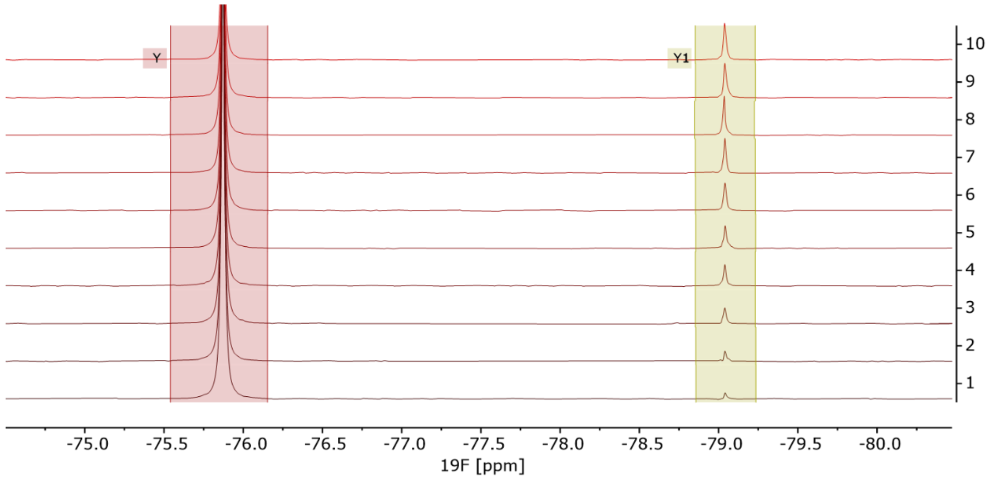

Figure S68: [19]F EXSY data.

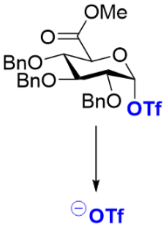

Spectrum number	Mixing Time [s]	α-triflate (-75.86 ppm) resonance intensity	⁻OTf (-78.99 ppm) resonance intensity
1	0.02	10617.6	163.297
2	0.04	10031.1	199.75
3	0.06	9501.96	360.511
4	0.08	8944	430.029
5	0.1	8422.9	586.584
6	0.12	7916.87	659.99
7	0.14	7509.69	753.067
8	0.16	6997.1	770.615
9	0.18	6560.91	885.5
10	0.2	6235.84	899.848

Temperature = -50 °C [OTf] = 190 mM

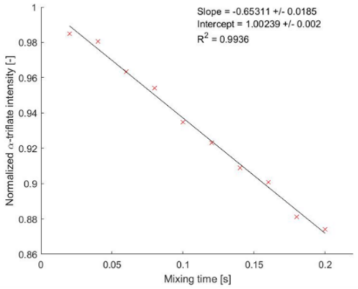

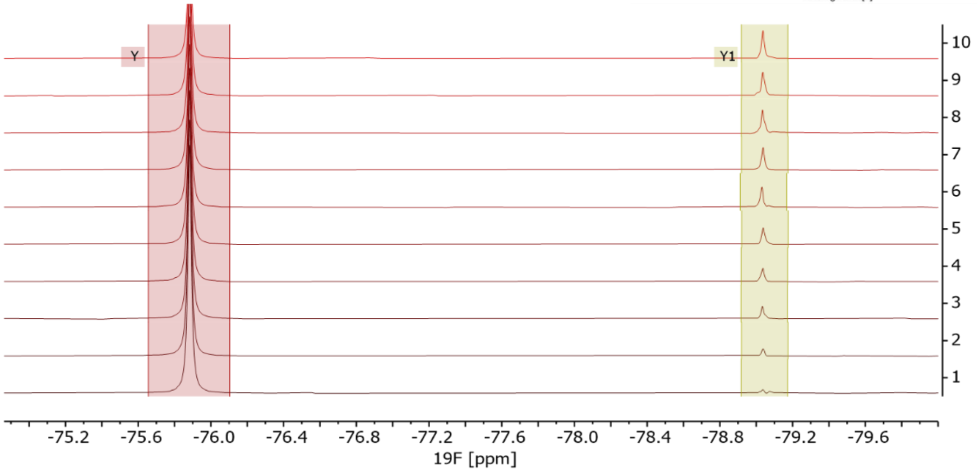

Figure S69: [19]F EXSY data.

Kinetic data – Ring flip – Tetrabutyl ammonium triflate

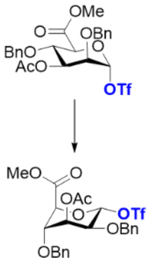

Spectrum number	Mixing Time [s]	Equatorial α-triflate resonance intensity	Axial α-triflate resonance intensity
1	0.002	480.247	11852.6
2	0.003	605.927	11531.5
3	0.004	747.921	11009.2
4	0.005	948.449	10619.7
5	0.006	1024.53	10582.6
6	0.007	1068	10514.1
7	0.008	1214.4	10446.7
8	0.009	1185.91	10008.7
9	0.01	1514.93	9771.75

Temperature = -80 °C [OTf] = 46 mM

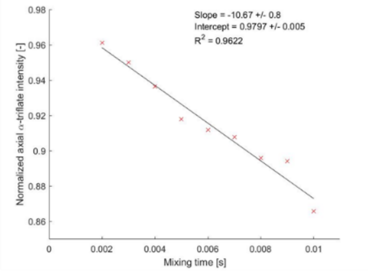

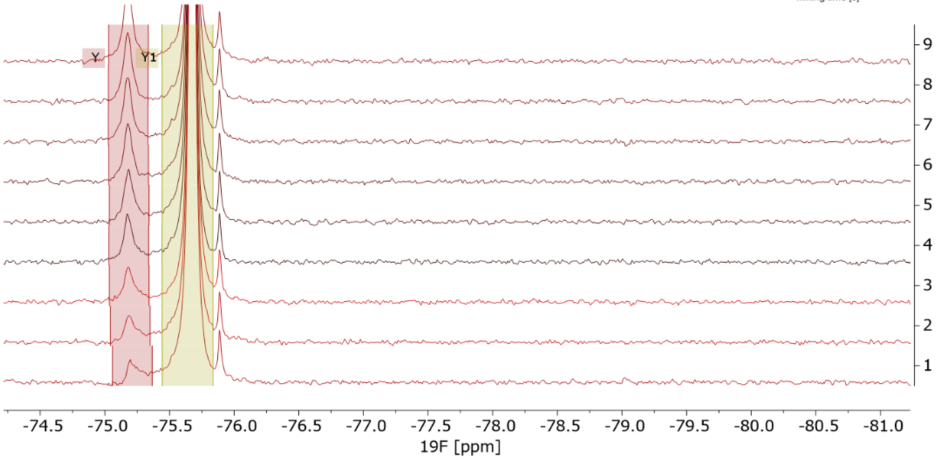

Figure S70: ^{19}F EXSY data.

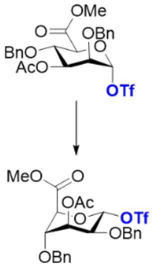

Spectrum number	Mixing Time [s]	Equatorial α-triflate resonance intensity	Axial α-triflate resonance intensity
1	0.002	453.615	9544.62
2	0.003	468.001	9252.76
3	0.004	573.377	8862.8
4	0.005	495.962	8401.41
5	0.006	844.528	8607.1
6	0.007	895.754	8552.98
7	0.008	905.393	8399.67
8	0.009	1073.32	8127.87
9	0.01	1148.9	7847.55

Temperature = -80 °C [OTf] = 84 mM

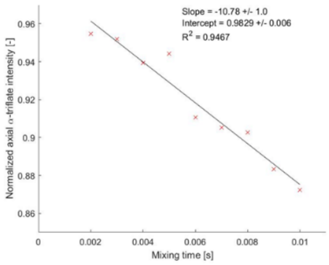

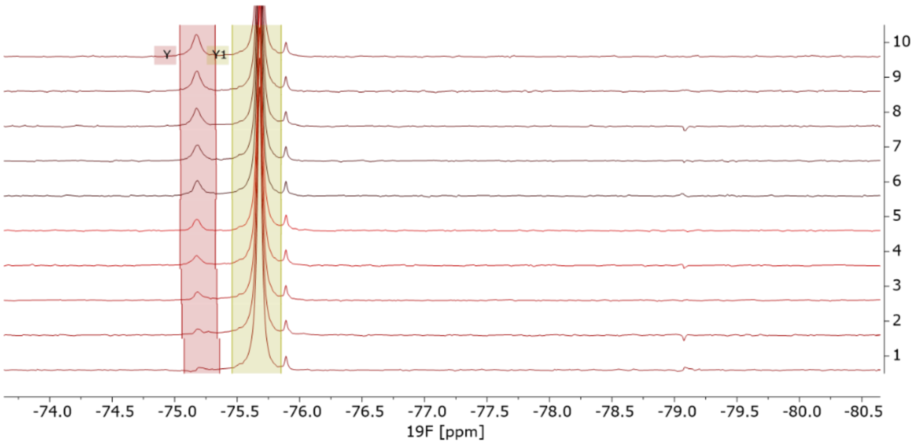

Figure S71: ^{19}F EXSY data.

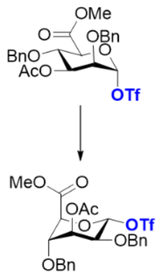

Spectrum number	Mixing Time [s]	Equatorial α-triflate resonance intensity	Axial α-triflate resonance intensity
1	0.002	7928.99	299.125
2	0.003	7607	332.833
3	0.004	7282.96	316.213
4	0.005	7016.41	468.435
5	0.006	7070.92	643.095
6	0.007	7158.74	713.872
7	0.008	7064.55	770.378
8	0.009	6592.44	729.362
9	0.01	6533.96	937.556

Temperature = -80 °C [OTf] = 145 mM

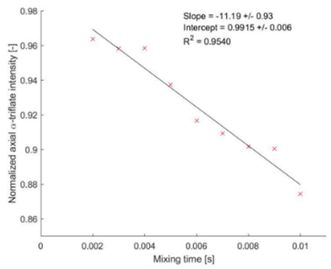

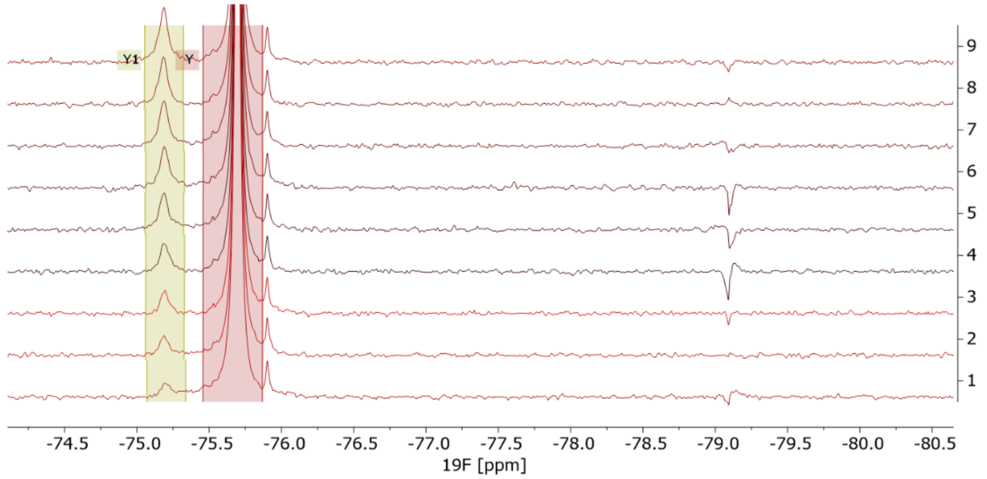

Figure S72: ^{19}F EXSY data.

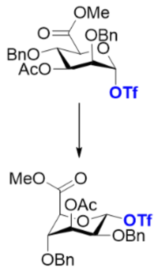

Spectrum number	Mixing Time [s]	Equatorial α-triflate resonance intensity	Axial α-triflate resonance intensity
1	0.002	120.385	5471.02
2	0.003	139.028	5405.31
3	0.004	235.016	5190.03
4	0.005	261.384	4958.51
5	0.006	412.414	5086.91
6	0.007	350.302	5063.02
7	0.008	435.132	4994.72
8	0.009	553.582	4812.71
9	0.01	645.737	4791.82

Temperature = -80 °C [OTf] = 223 mM

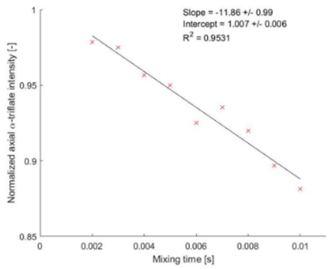

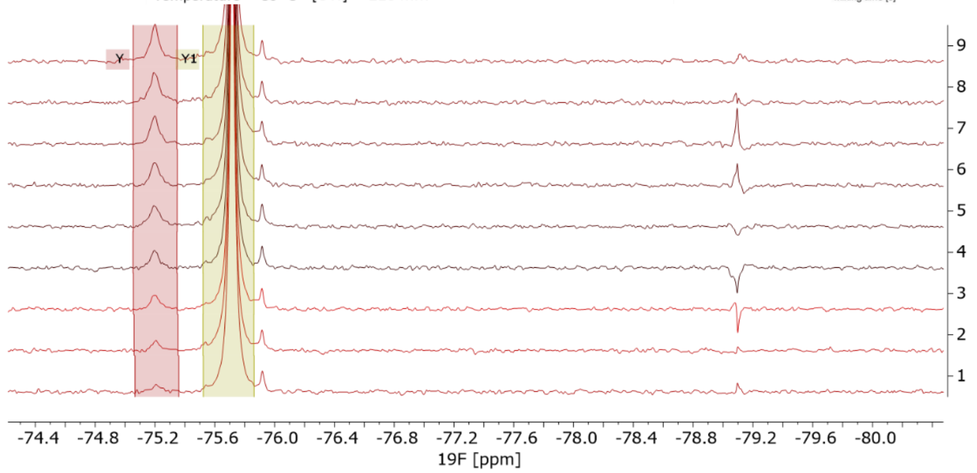

Figure S73: ^{19}F EXSY data.

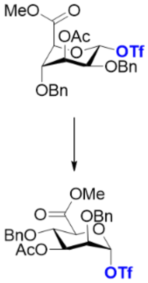

Spectrum number	Mixing Time [s]	Equatorial α-triflate resonance intensity	Axial α-triflate resonance intensity
1	0.001	3016.97	545.727
2	0.002	2423.23	173.919
3	0.003	2765.11	647.592
4	0.004	2187.63	453.158
5	0.005	2298.05	596.844
6	0.006	2221.16	723.882
7	0.007	2018.93	726.137
8	0.008	1895.21	724.529
9	0.009	1798.27	768.5
10	0.01	1888.69	931.385

Temperature = -80 °C [OTf] = 46 mM

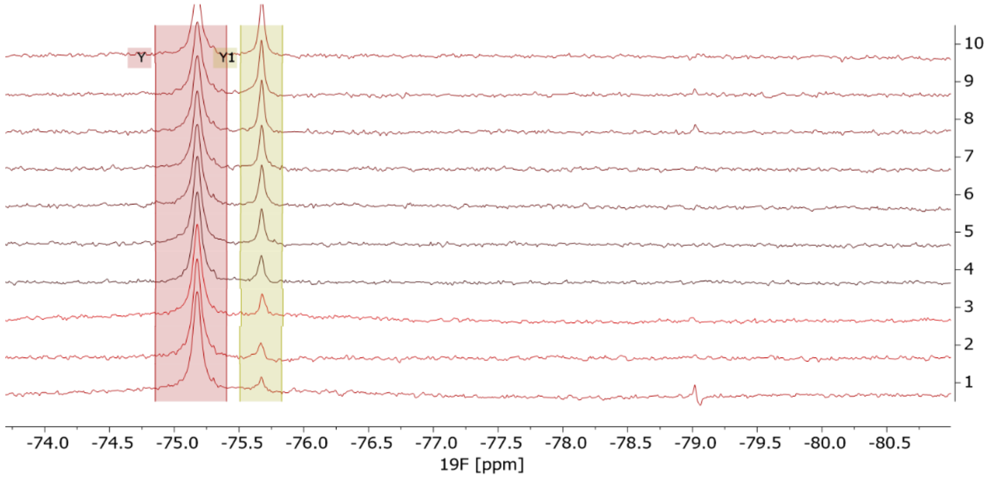

Figure S74: ^{19}F EXSY data.

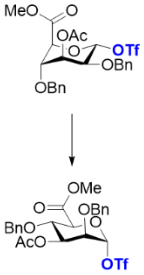

Spectrum number	Mixing Time [s]	Equatorial α-triflate resonance intensity	Axial α-triflate resonance intensity
1	0.001	2161.57	178.507
2	0.002	2083.77	309.906
3	0.003	1994.45	379.774
4	0.004	1887.43	415.013
5	0.005	1725.4	403.979
6	0.006	1605.26	450.831
7	0.007	1572.3	533.849
8	0.008	1499.48	552.07
9	0.009	1428.17	634.507
10	0.01	1349.59	678.969

Temperature = -80 °C [OTf] = 84 mM

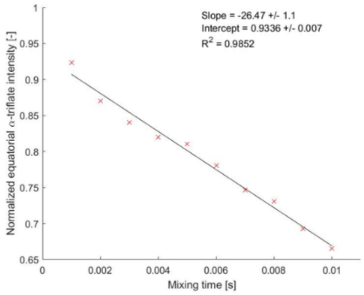

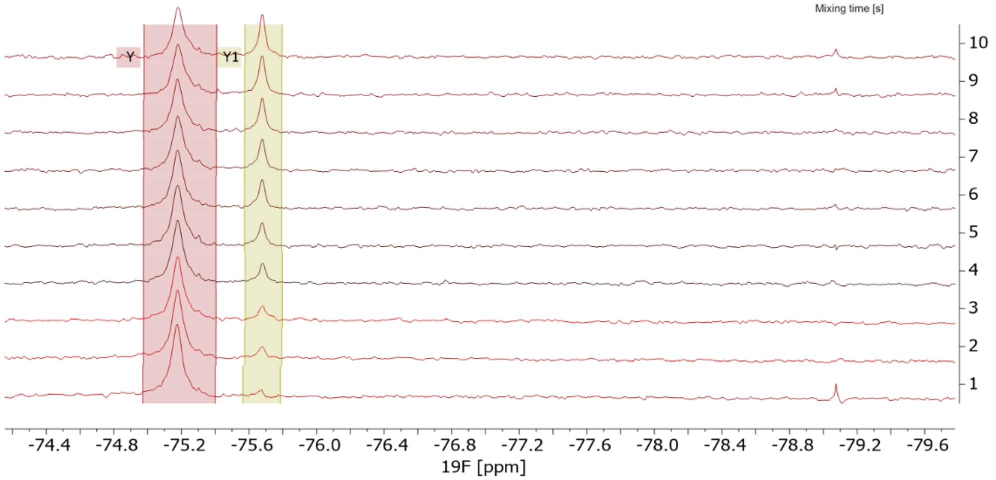

Figure S75: ^{19}F EXSY data.

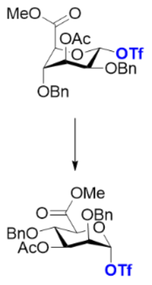

Spectrum number	Mixing Time [s]	Equatorial α-triflate resonance intensity	Axial α-triflate resonance intensity
1	0.001	1442.44	78.414
2	0.002	1420.69	129.341
3	0.003	1350.6	151.942
4	0.004	1480.55	260.902
5	0.005	1294.78	271.689
6	0.006	1409.2	407.283
7	0.007	1212.85	366.79
8	0.008	1079.78	411.734
9	0.009	1229.2	549.319
10	0.01	1196.32	533.98

Temperature = -80 °C [OTf] = 145 mM

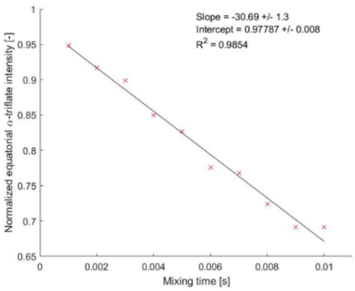

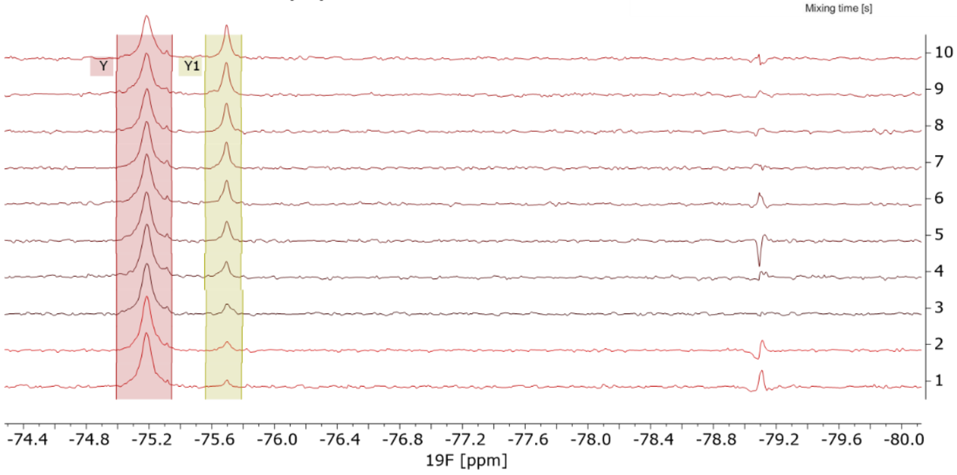

Figure S76: ^{19}F EXSY data.

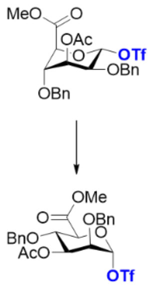

Spectrum number	Mixing Time [s]	Equatorial α-triflate resonance intensity	Axial α-triflate resonance intensity
1	0.001	1203.54	35.6757
2	0.002	1093.68	97.7432
3	0.003	1093.19	158.948
4	0.004	1036.25	180.446
5	0.005	1016.2	197.777
6	0.006	947.776	203.14
7	0.007	963.561	242.816
8	0.008	891	266.567
9	0.009	783.099	323.979
10	0.01	852.72	342.045

Temperature = -80 °C [OTf] = 223 mM

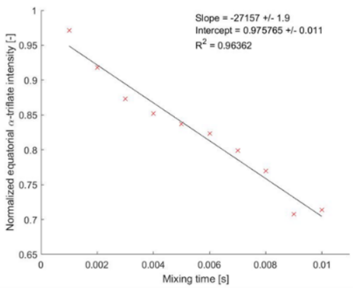

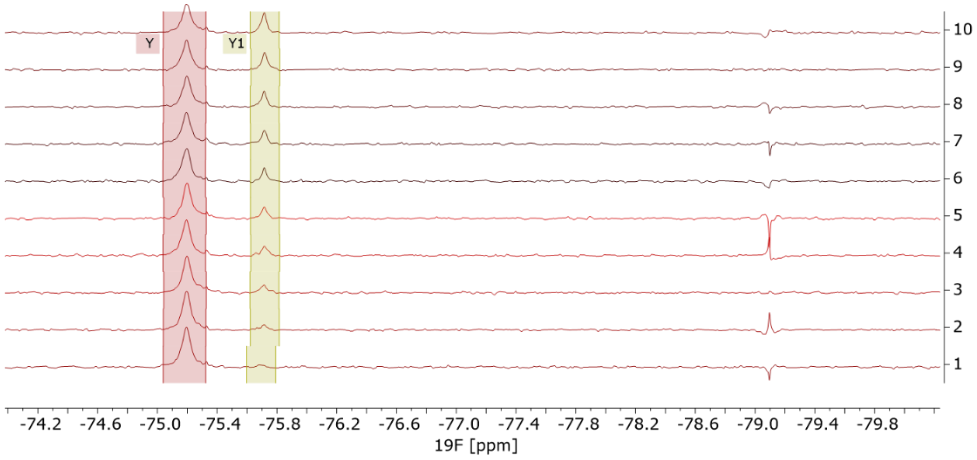

Figure S77: ^{19}F EXSY data.

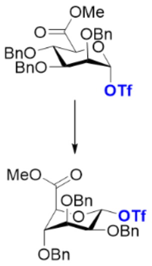

Spectrum number	Mixing Time [s]	Equatorial α-triflate resonance intensity	Axial α-triflate resonance intensity
1	0.0005	291.684	8695.52
2	0.001	350.142	8826.01
3	0.0015	446.289	8966.21
4	0.002	496.449	8746.4
5	0.0025	612.732	8962.86
6	0.003	897.984	8902.65
7	0.0035	882.479	8503.19
8	0.004	957.358	8356.77
9	0.0045	1043.49	8342.79
10	0.005	1255.43	8583.22

Temperature = -80 °C [OTf] = 67 mM

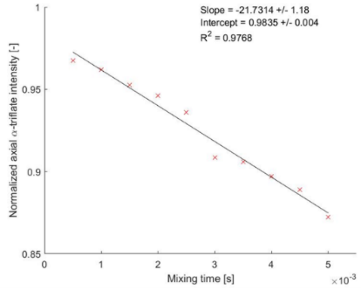

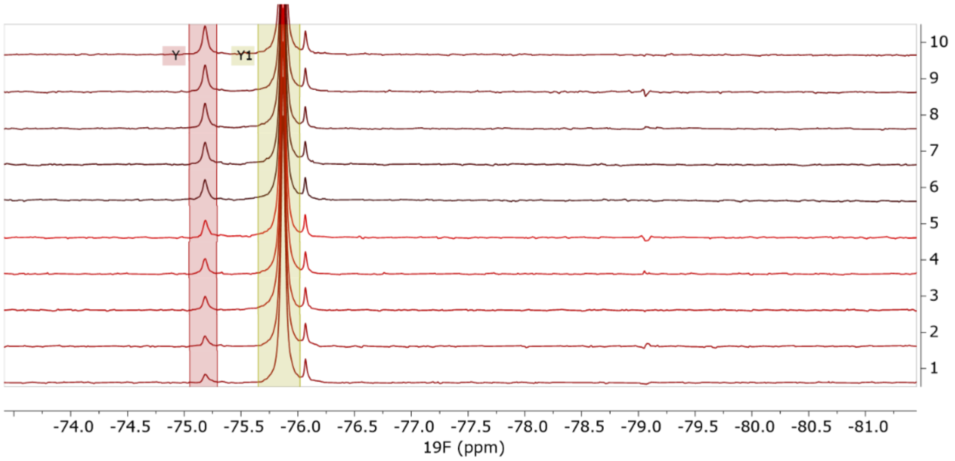

Figure S78: ^{19}F EXSY data.

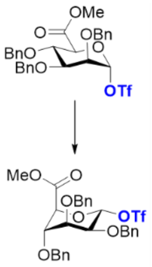

Spectrum number	Mixing Time [s]	Equatorial α-triflate resonance intensity	Axial α-triflate resonance intensity
1	0.0005	235.453	5743.13
2	0.001	250.221	5685.73
3	0.0015	316.822	5715.76
4	0.002	376.587	5667.98
5	0.0025	422.031	5572.16
6	0.003	568.991	5574.43
7	0.0035	571.397	5457.13
8	0.004	720.912	5305.57
9	0.0045	816.096	5307.58
10	0.0005	235.453	5743.13

Temperature = -80 °C [OTf] = 123 mM

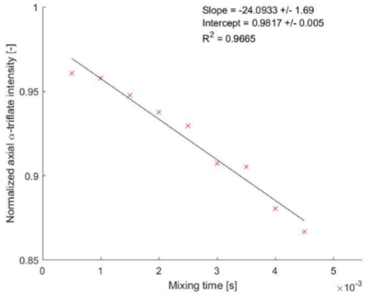

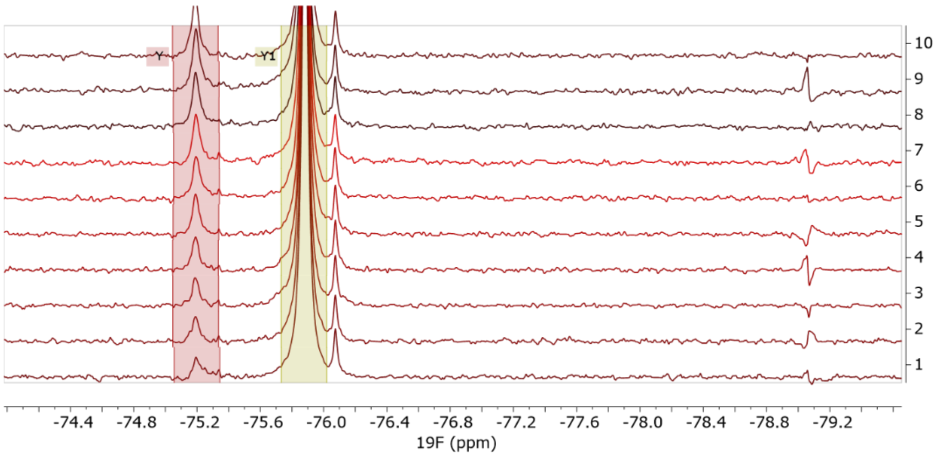

Figure S79: ^{19}F EXSY data.

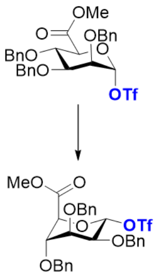

Spectrum number	Mixing Time [s]	Equatorial α-triflate resonance intensity	Axial α-triflate resonance intensity
1	0.0015	239.583	4376.86
2	0.002	246.756	4427.64
3	0.0025	287.175	4288.57
4	0.004	410.832	3997.67
5	0.0045	477.338	3971.4
6	0.005	514.869	3906.09

Temperature = -80 °C [OTf] = 164 mM

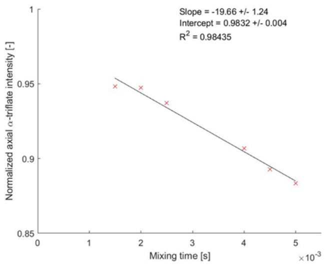

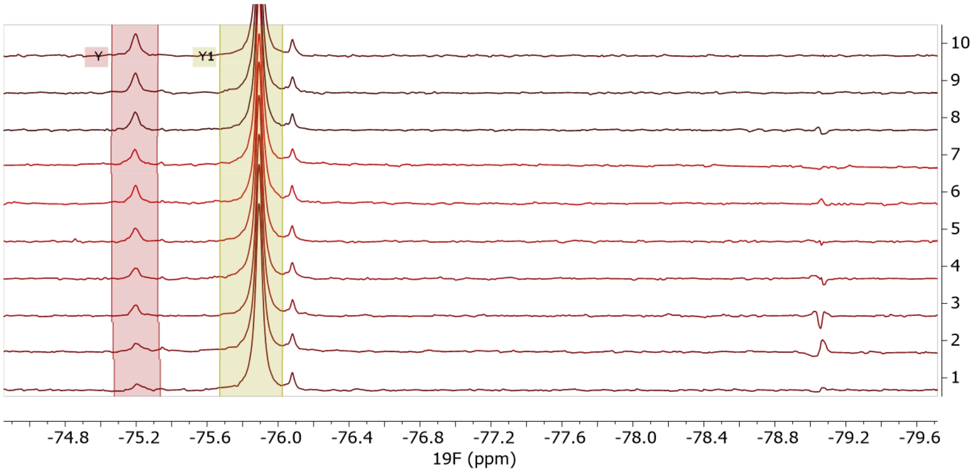

Figure S80: ^{19}F EXSY data.

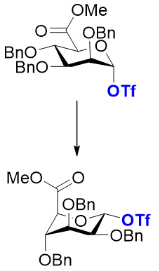

Spectrum number	Mixing Time [s]	Equatorial α-triflate resonance intensity	Axial α-triflate resonance intensity
1	0.0005	85.6667	3344.44
2	0.001	137.988	3369.56
3	0.0015	215.698	3350.52
4	0.002	212.699	3242.14
5	0.0025	206.844	3147.57
6	0.003	228.326	3071.6
7	0.0035	270.309	2987.96
8	0.004	320.089	3058.37
9	0.0045	354.916	2993.91
10	0.005	371.707	3045.25

Temperature = -80 °C [OTf] = 205 mM

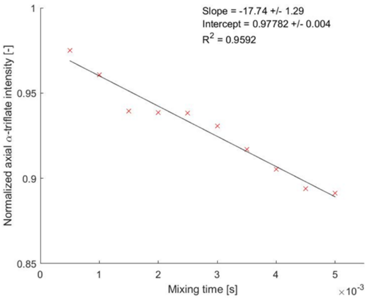

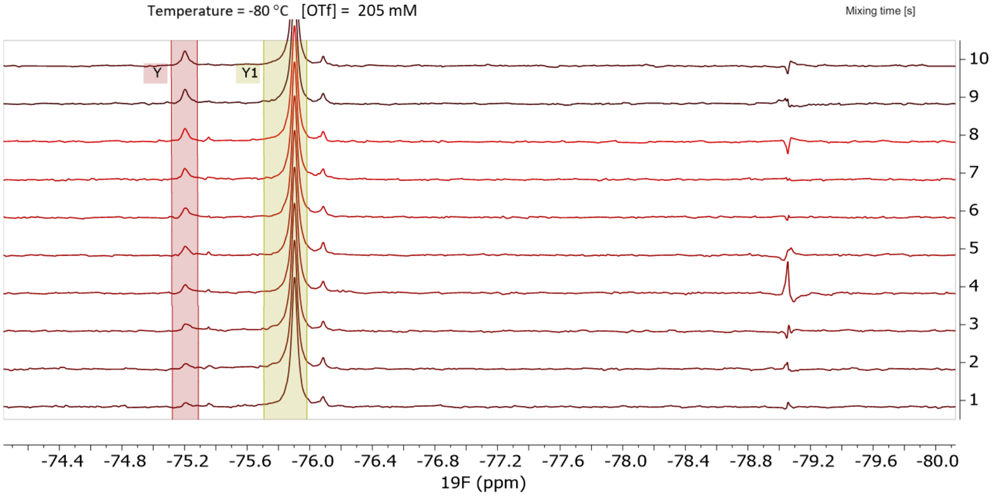

Figure S81: ^{19}F EXSY data.

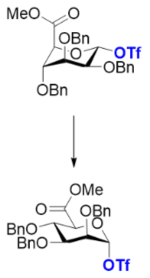

Spectrum number	Mixing Time [s]	Equatorial α-triflate resonance intensity	Axial α-triflate resonance intensity
1	0.0015	6630.71	404.9
2	0.002	6621.63	429.424
3	0.0025	6489.16	555.755
4	0.003	6516.57	583.496
5	0.0035	6392.08	698.854
6	0.004	6406.48	842.509
7	0.0045	6241.28	839.53

Temperature = -80 °C [OTf] = 67 mM

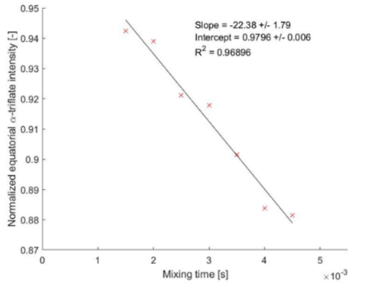

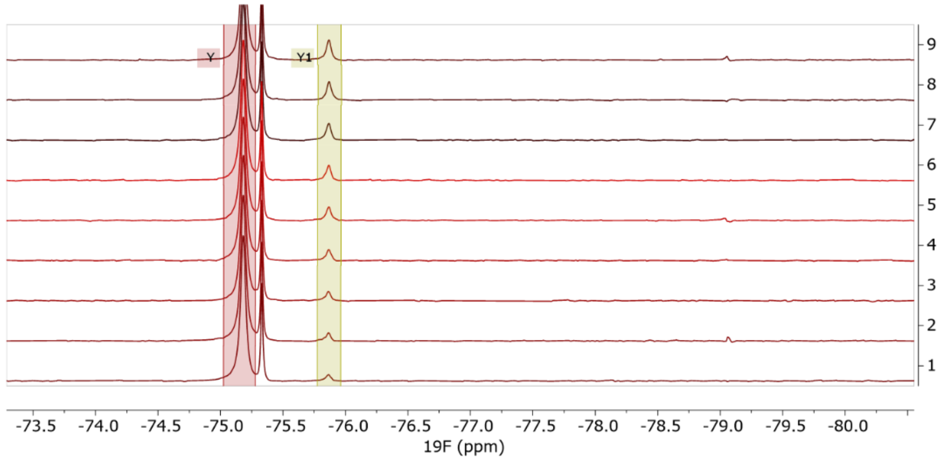

Figure S82: ^{19}F EXSY data.

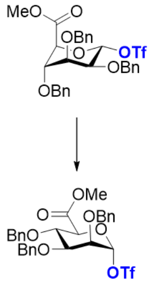

Spectrum number	Mixing Time [s]	Equatorial α-triflate resonance intensity	Axial α-triflate resonance intensity
1	0.0015	5025.51	225.949
2	0.002	5319.05	370.532
3	0.0025	4794.24	442.984
4	0.003	4821.54	449.527
5	0.0035	4754.91	518.791
6	0.004	4684.95	568.126
7	0.0045	4657.3	647.263
8	0.005	4553.61	691.391

Temperature = -80 °C [OTf] = 123 mM

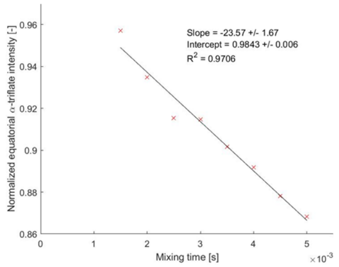

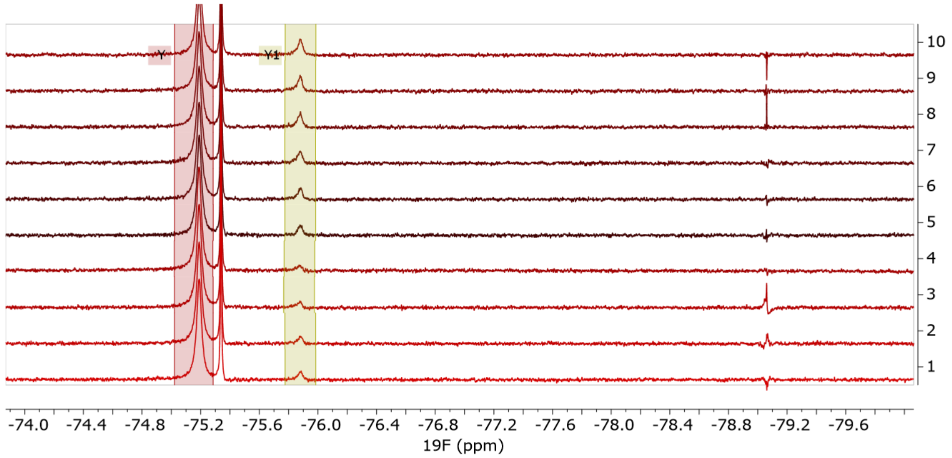

Figure S83: ^{19}F EXSY data.

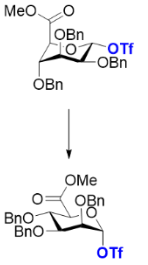

Spectrum number	Mixing Time [s]	Equatorial α-triflate resonance intensity	Axial α-triflate resonance intensity
1	0.0005	4066.21	116.844
2	0.001	4024.03	158.151
3	0.0015	3896.83	225.62
4	0.002	3939.3	263.187
5	0.0025	3795.1	303.39
6	0.003	3764.88	347.33
7	0.0035	3747.17	382.744
8	0.004	3704.2	408.016
9	0.0045	3656.96	463.019
10	0.005	3594.44	515.129

Temperature = -80 °C [OTf] = 164 mM

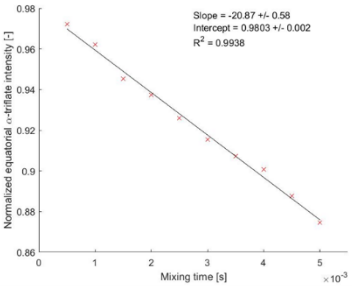

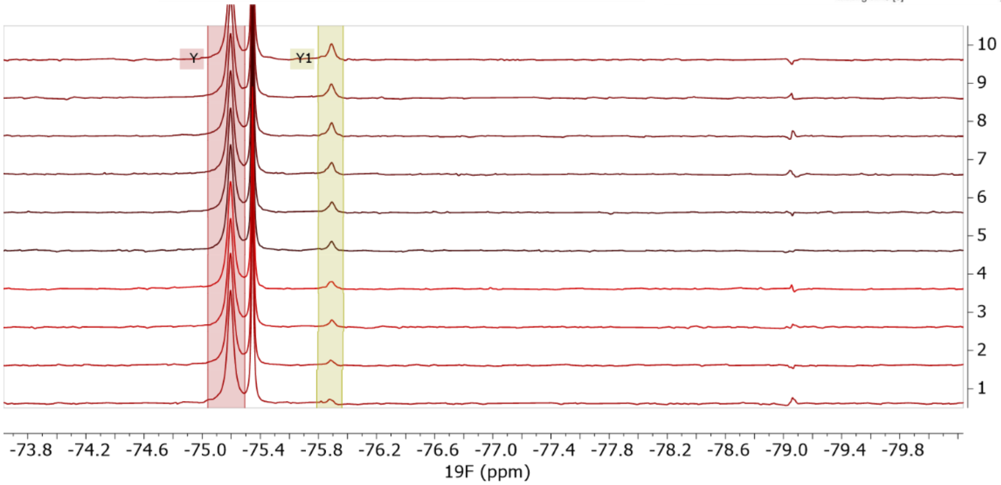

Figure S84: ^{19}F EXSY data.

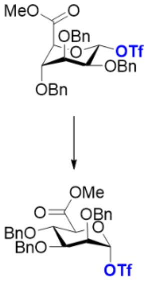

Spectrum number	Mixing Time [s]	Equatorial α-triflate resonance intensity	Axial α-triflate resonance intensity
1	0.001	3153.48	115.69
2	0.0015	3131.78	167.483
3	0.002	3102.23	130.73
4	0.0025	3031.18	218.007
5	0.003	2950.33	277.089
6	0.0035	2942.63	302.098
7	0.004	2955.05	297.776
8	0.0045	2858.63	340.371
9	0.005	2780.13	388.578

Temperature = -80 °C [OTf] = 205 mM

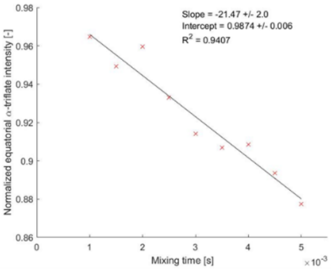

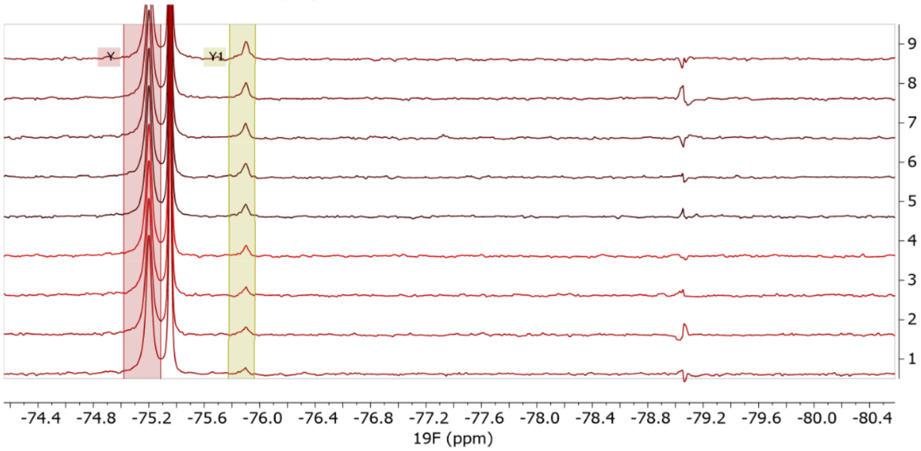

Figure S85: ^{19}F EXSY data.

^{19}F EXSY profiles raw data

Table S5: ^{19}F EXSY data selecting the free triflate resonance at varying mix times.

No.	Mix time [s]	Integral equatorial triflate [-]	Integral axial triflate [-]	Integral OTf
1	0.02	0.03088	0	17.404
2	0.03	0.04425	0.020802	16.8054
3	0.04	0.053721	0.027817	16.3091
4	0.05	0.062447	0.034034	15.988
5	0.06	0.068268	0.040476	15.7117
6	0.07	0.071457	0.05305	15.4035
7	0.08	0.083379	0.05635	14.9497
8	0.09	0.080908	0.069713	14.6417
9	0.1	0.073985	0.073746	11.3894
10	0.125	0.084091	0.088836	10.6416
11	0.15	0.084579	0.101907	9.9585
12	0.175	0.090042	0.11741	9.46573
13	0.2	0.093094	0.124483	8.76271
14	0.225	0.095665	0.130835	8.35573
15	0.25	0.091108	0.128809	7.69028
16	0.275	0.092405	0.139817	7.27146
17	0.3	0.09345	0.138792	6.79495
18	0.325	0.087371	0.142719	6.36592
19	0.35	0.089673	0.147353	6.02218
20	0.375	0.087134	0.142414	5.64814
21	0.4	0.081677	0.153585	5.28487

Table S6: ^{19}F EXSY data selecting the axial α-triflate resonance at varying mix times.

No.	Mix time [s]	Integral equatorial triflate [-]	Integral axial triflate [-]	Integral OTf
1	0.001	0.058929	6.72877	0
2	0.002	0.127314	6.61713	0
3	0.003	0.226821	6.35161	0
4	0.004	0.228766	6.60351	0
5	0.005	0.277397	6.25647	0
6	0.006	0.383486	6.11337	0
7	0.007	0.388755	6.38102	0
8	0.008	0.442725	6.03232	0
9	0.009	0.484277	5.8482	0
10	0.01	0.538547	6.07934	0.003853
11	0.02	0.908892	5.22681	0.003616
12	0.03	1.16533	4.88461	0.016658
13	0.04	1.28802	4.60447	0.0176
14	0.05	1.39858	4.17456	0.02316
15	0.06	1.43424	4.02328	0.03758
16	0.07	1.46403	3.76516	0.045717
17	0.08	1.49895	3.63362	0.049482
18	0.09	1.46101	3.488	0.059763
19	0.1	1.14042	2.56709	0.062493
20	0.125	1.0762	2.33563	0.072534
21	0.15	0.998632	2.12597	0.086257
22	0.175	0.920956	1.95475	0.097813
23	0.2	0.855822	1.80683	0.10386
24	0.225	0.793035	1.67783	0.107742
25	0.25	0.719188	1.52949	0.116471
26	0.275	0.65319	1.41174	0.117769
27	0.3	0.619307	1.29901	0.118599
28	0.325	0.553757	1.19664	0.122386
29	0.35	0.522895	1.09605	0.116705
30	0.375	0.472898	1.00787	0.121852
31	0.4	0.430122	0.935788	0.119223

Table S7: ^{19}F EXSY data selecting the equatorial α-triflate resonance at varying mix times.

No.	Mix time [s]	Integral equatorial triflate [-]	Integral axial triflate [-]	Integral OTf
1	0.001	2.60534	0.054105	0
2	0.002	2.52609	0.095724	0
3	0.003	2.39825	0.155516	0
4	0.004	2.39532	0.181514	0
5	0.005	2.32983	0.231743	0
6	0.006	2.24023	0.287073	0
7	0.007	2.23532	0.315507	0
8	0.008	2.17831	0.351528	0
9	0.009	2.09084	0.388211	0
10	0.01	2.09685	0.434179	0.01664
11	0.02	1.69833	0.730536	0.030255
12	0.03	1.37923	0.828975	0.034695
13	0.04	1.18517	1.04904	0.042614
14	0.05	1.02107	1.13754	0.042893
15	0.06	0.880098	1.1749	0.043981
16	0.07	0.788015	1.20107	0.054668
17	0.08	0.73658	1.1965	0.054611
18	0.09	0.680833	1.1897	0.06228
19	0.1	0.458325	0.905973	0.053458
20	0.125	0.413221	0.849252	0.052366
21	0.15	0.37168	0.783932	0.057827
22	0.175	0.336309	0.730374	0.057245
23	0.2	0.31287	0.665395	0.060575
24	0.225	0.290299	0.618028	0.065397
25	0.25	0.263523	0.570614	0.06579
26	0.275	0.250136	0.516915	0.059137
27	0.3	0.219733	0.476823	0.057389
28	0.325	0.206961	0.44525	0.067812
29	0.35	0.19302	0.403122	0.061031
30	0.375	0.174497	0.371522	0.057918
31	0.4	0.159681	0.352578	0.059815

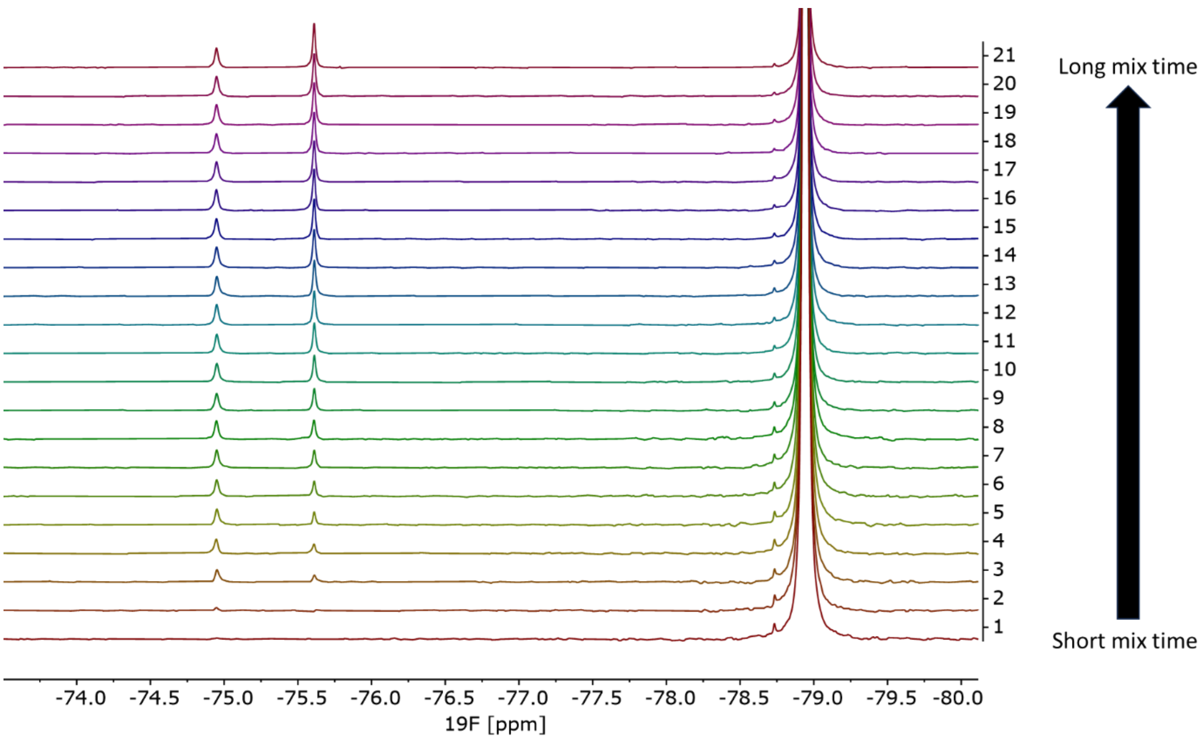

Figure S86: ^{19}F EXSY data on selecting the free triflate resonance and increment the mix time to 400 ms.

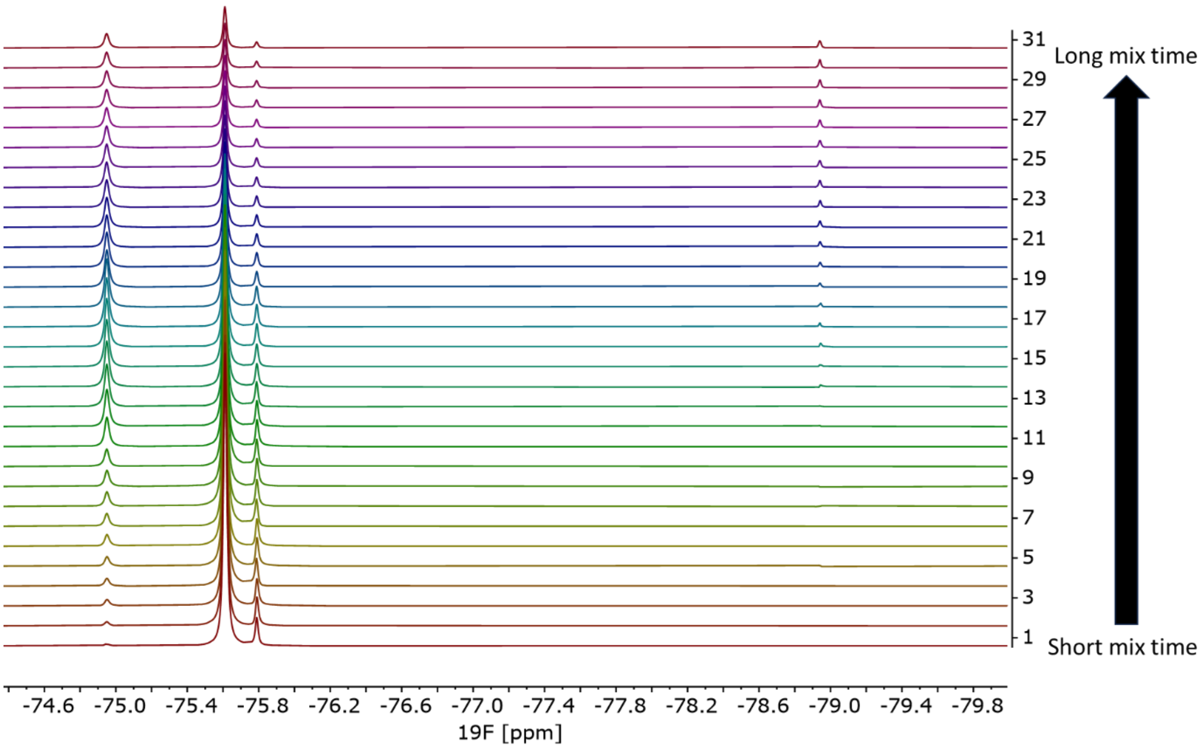

Figure S87: ^{19}F EXSY data on selecting the axial α-triflate resonance and increment the mix time to 400 ms.

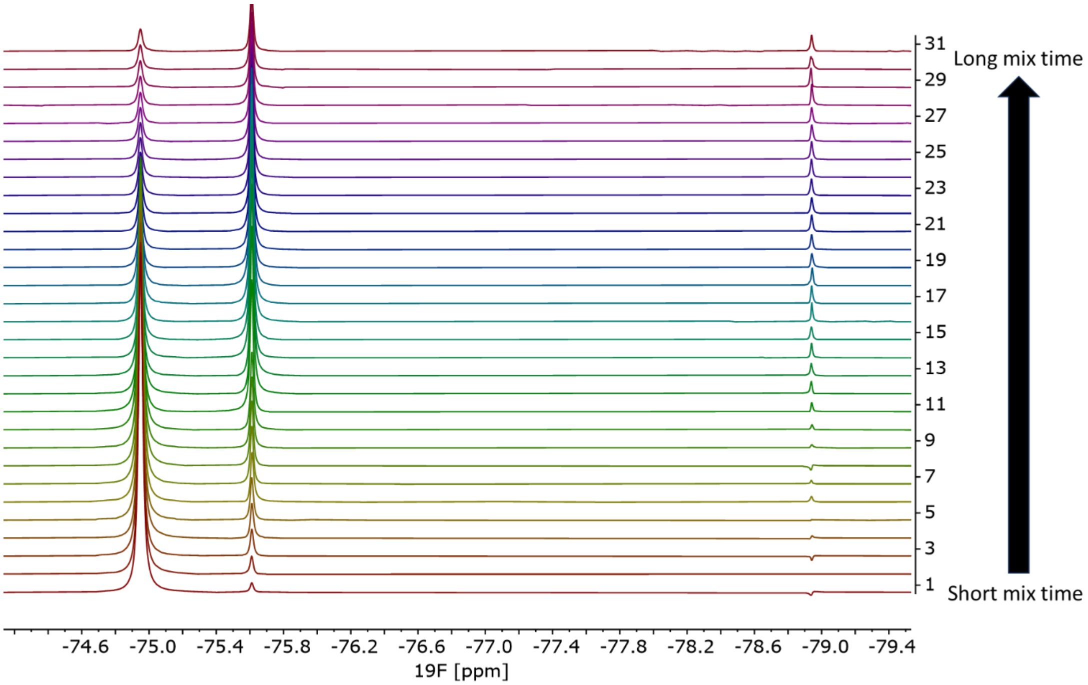

Figure S88: ^{19}F EXSY data on selecting the equatorial α-triflate resonance and increment the mix time to 400 ms.

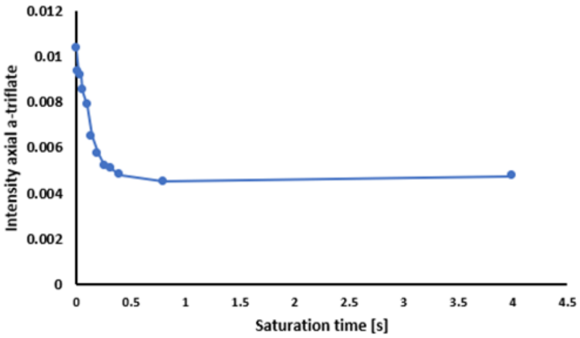

Saturation time [s]	Axial α-triflate resonance intensity [-]
0	0.01035
0.02	0.009335
0.04	0.009199
0.06	0.008542
0.1	0.007874
0.14	0.006518
0.2	0.005749
0.26	0.005231
0.32	0.005083
0.4	0.004812
0.8	0.004522
4	0.004759

The lowest value was used for $M_z(\infty)$

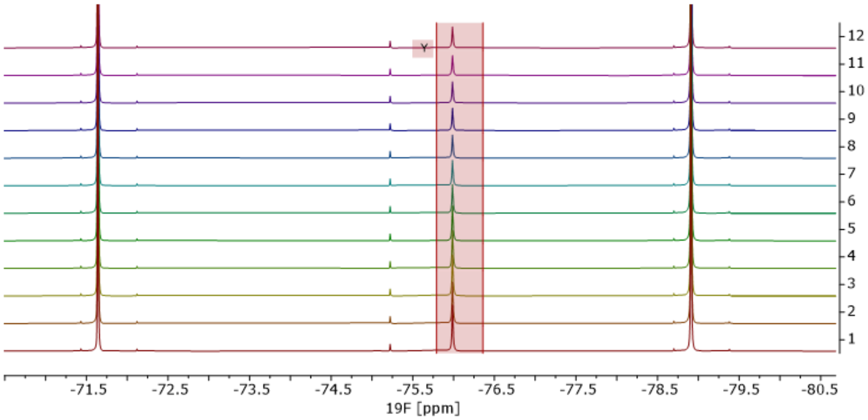

Figure S89: Studying axial α-triflate kinetics in rhamnose.

Synthesis spectra

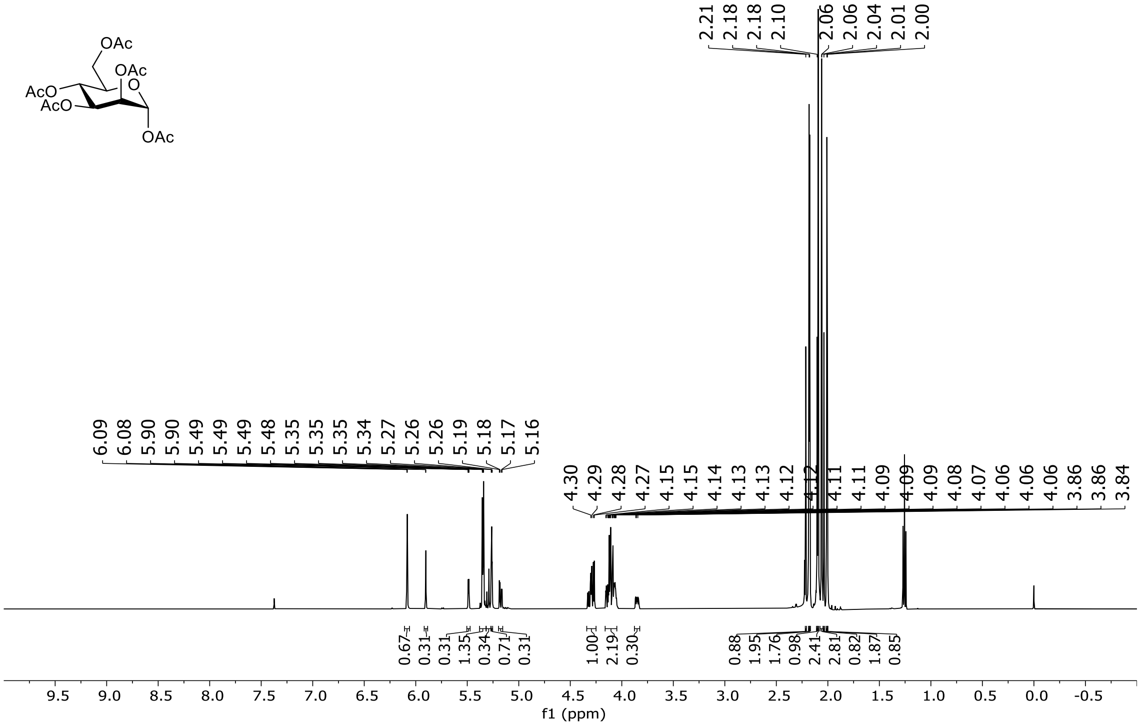

Figure S90: ^{1}H NMR spectrum.

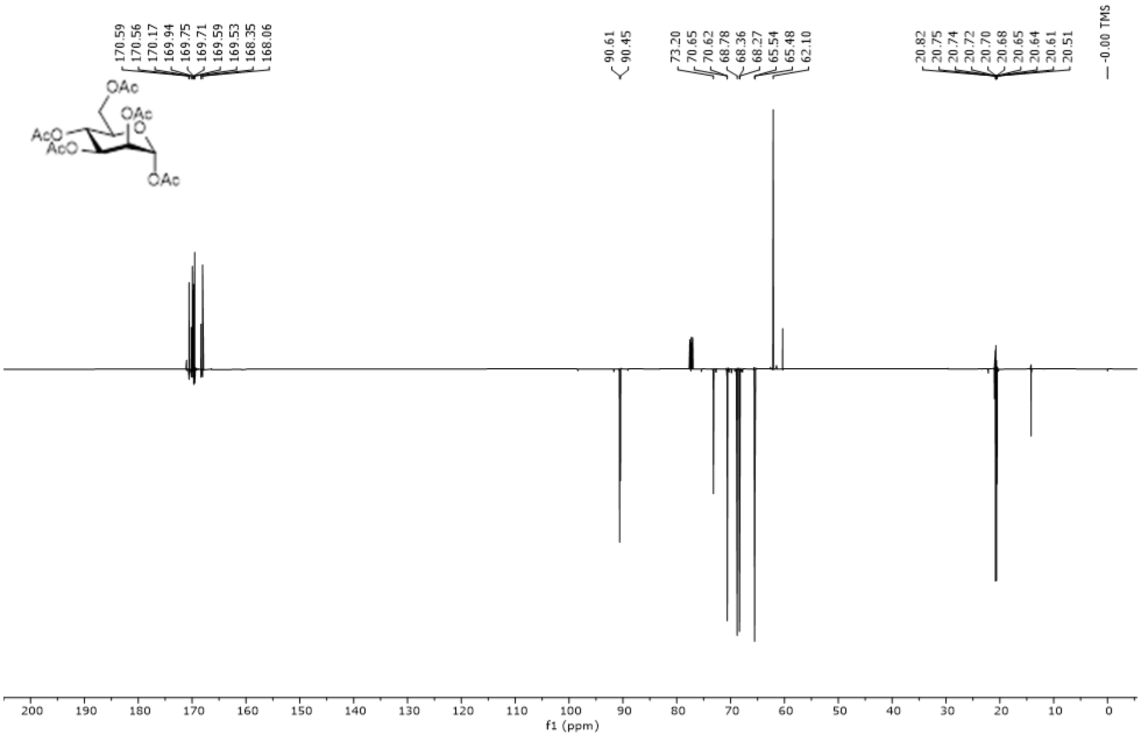

Figure S91: ^{13}C NMR spectrum.

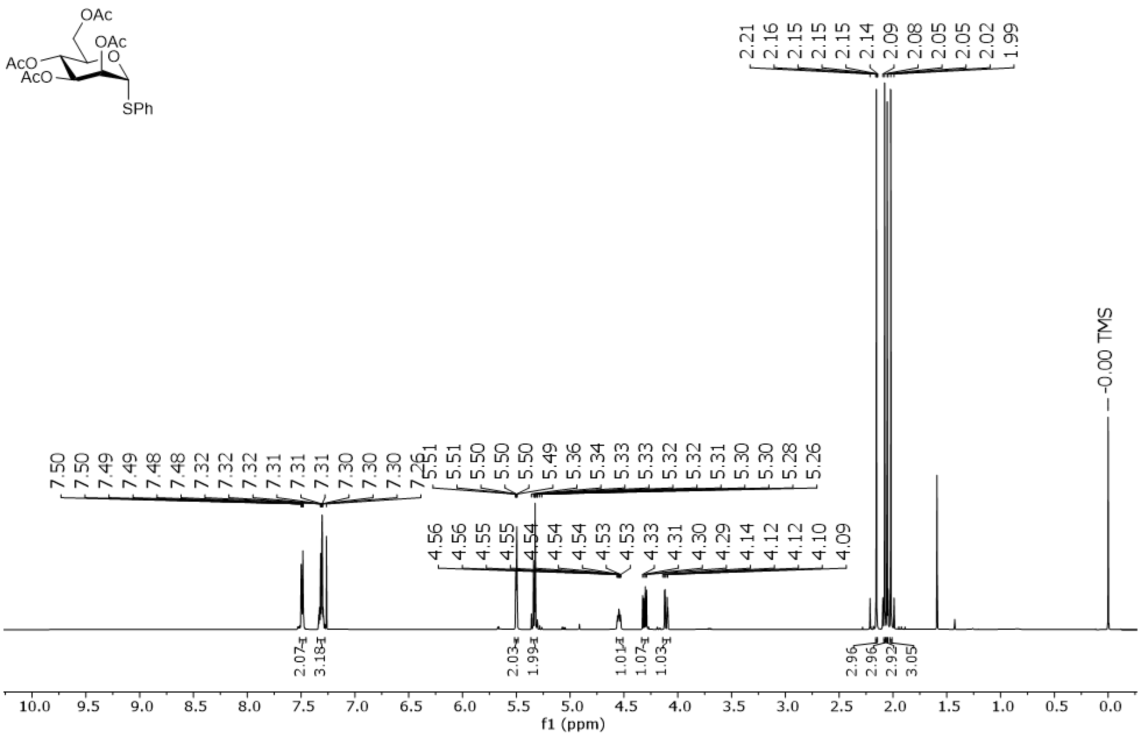

Figure S92: ^{1}H NMR spectrum.

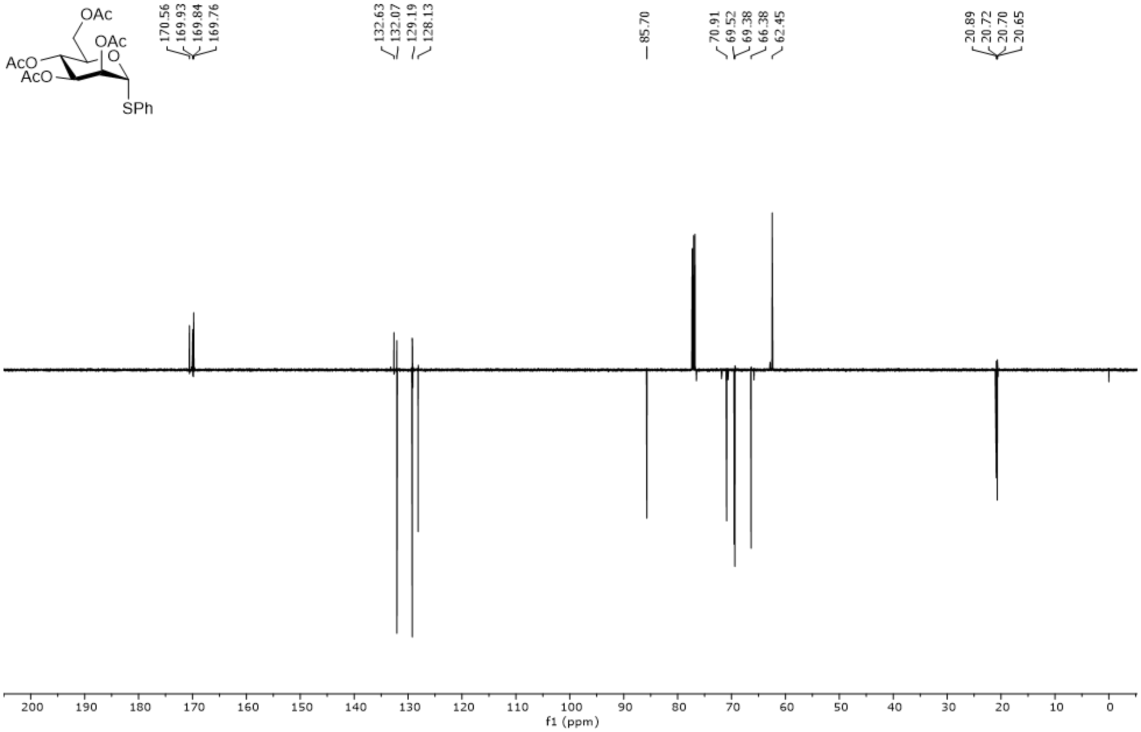

Figure S93: ^{13}C NMR spectrum.

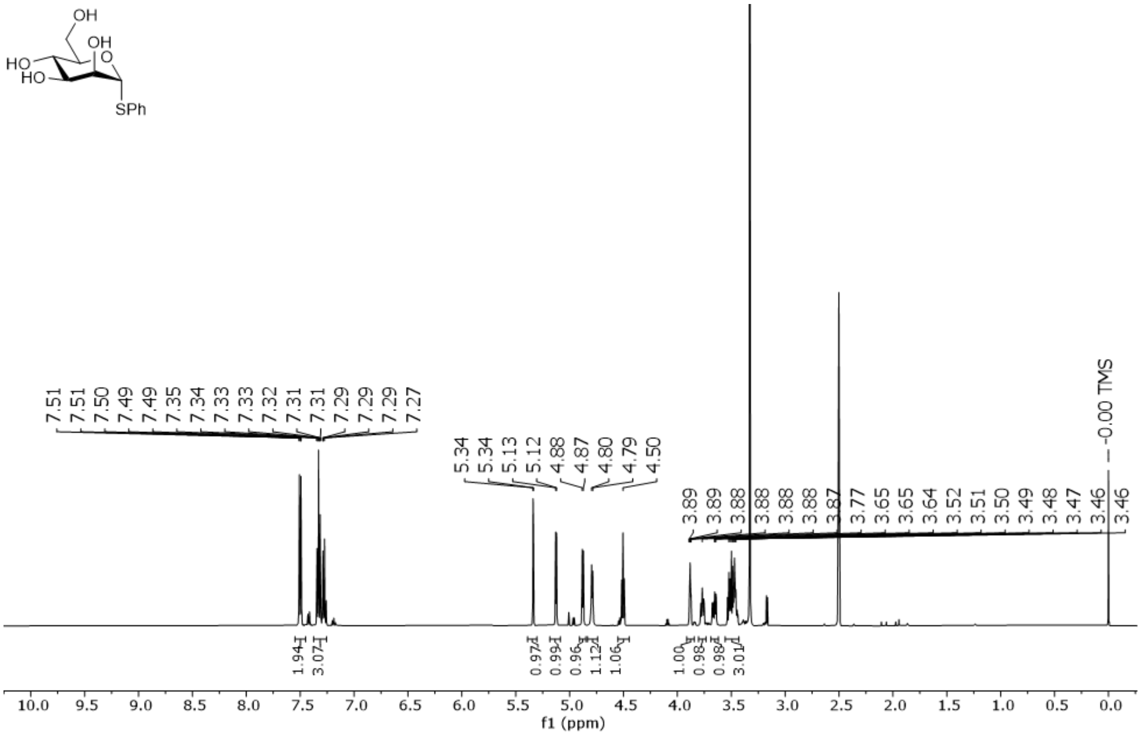

Figure S94: ^{1}H NMR spectrum.

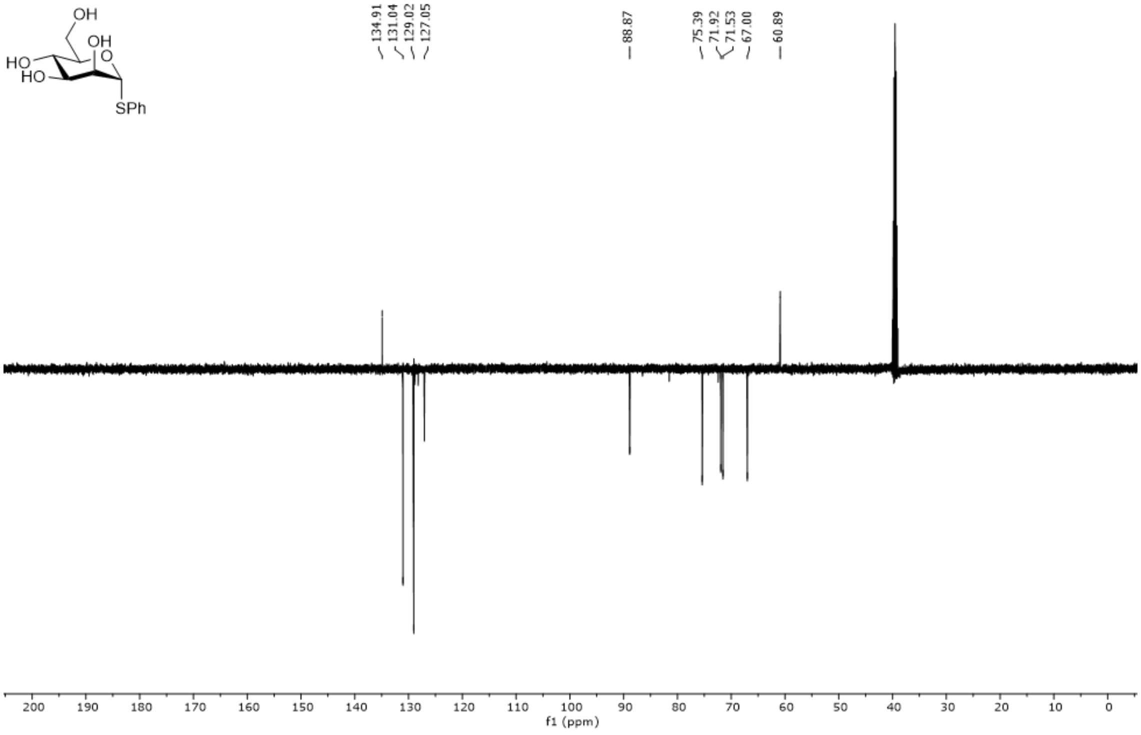

Figure S95: ^{13}C NMR spectrum.

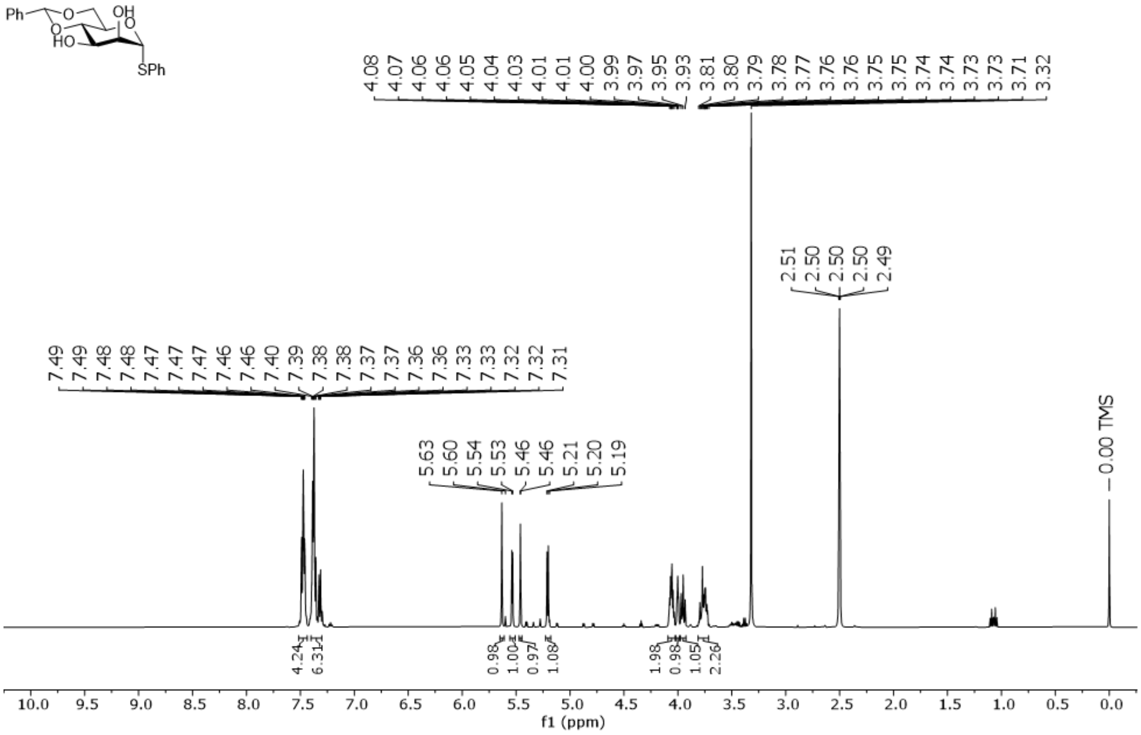

Figure S96: ^{1}H NMR spectrum.

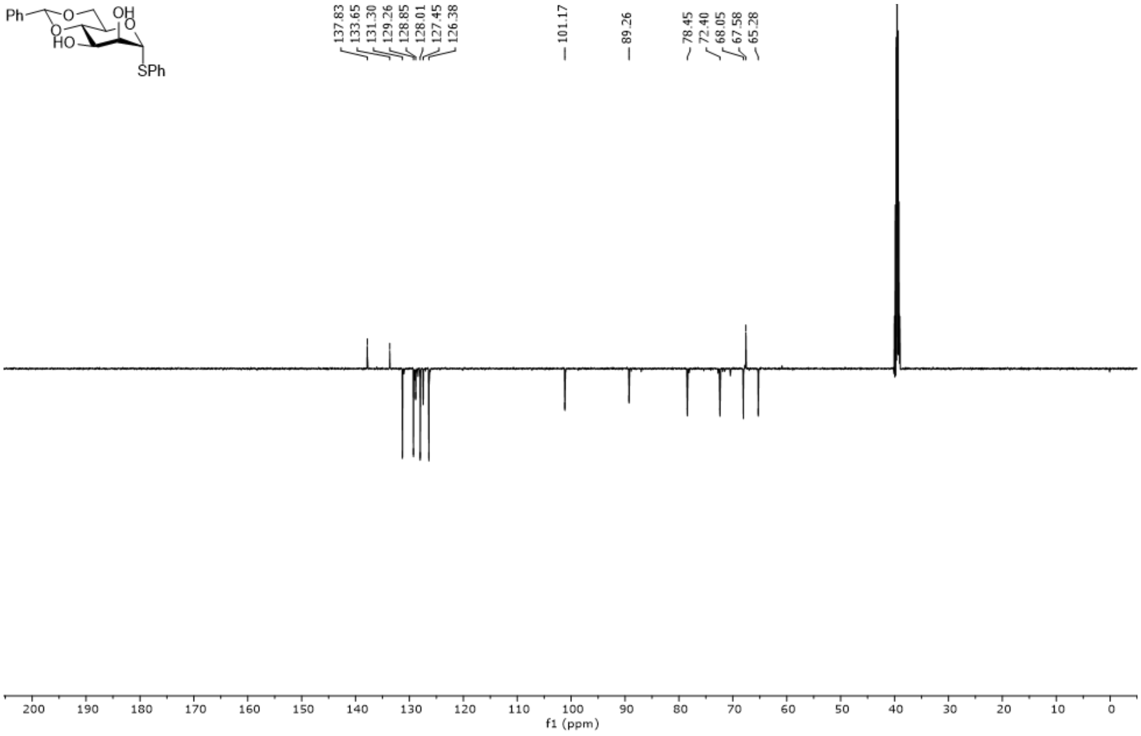

Figure S97: ^{13}C NMR spectrum.

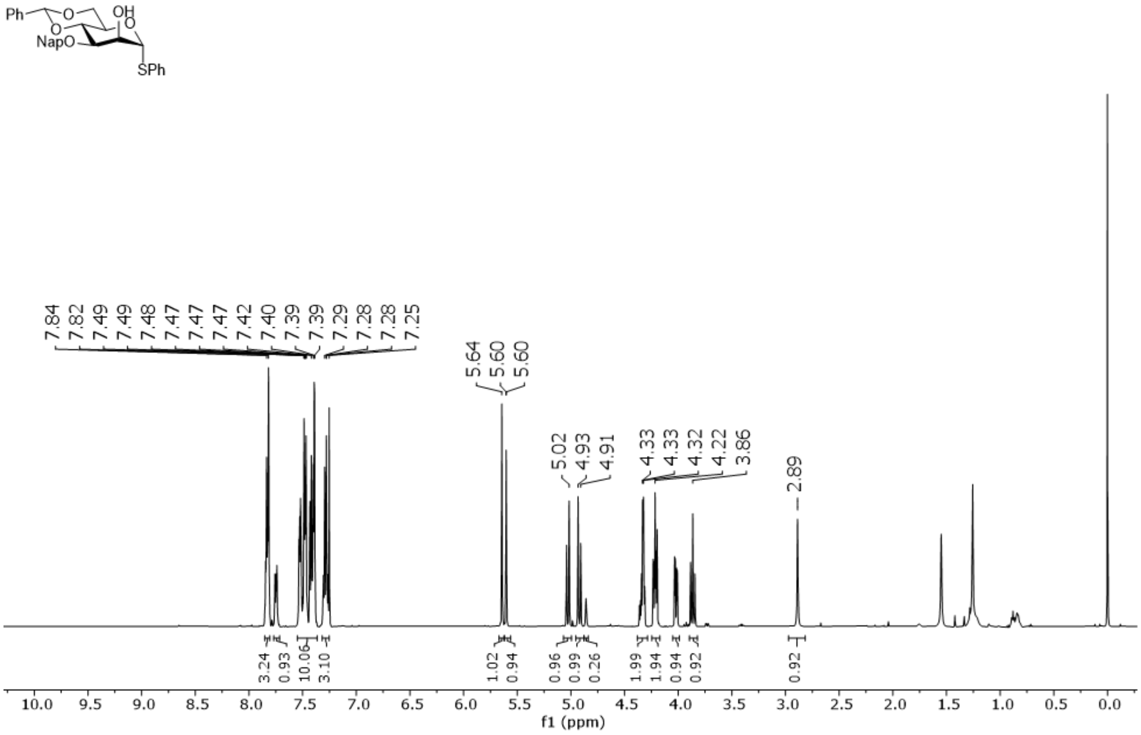

Figure S98: ^{1}H NMR spectrum.

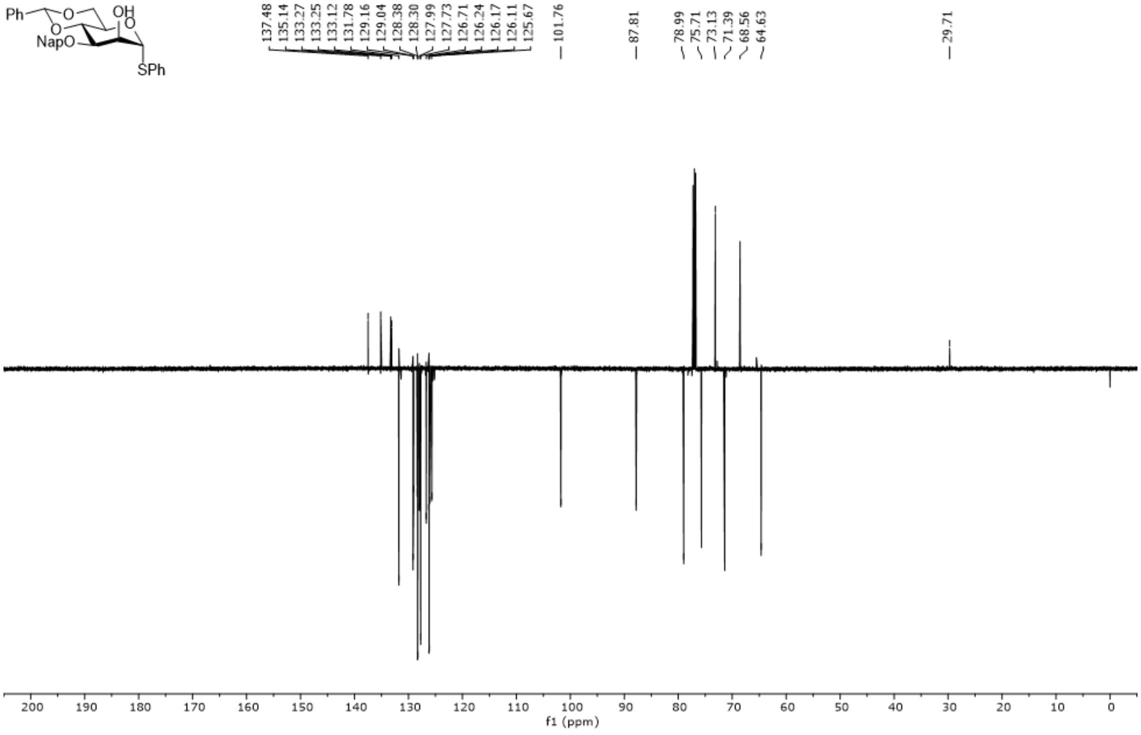

Figure S99: ^{13}C NMR spectrum.

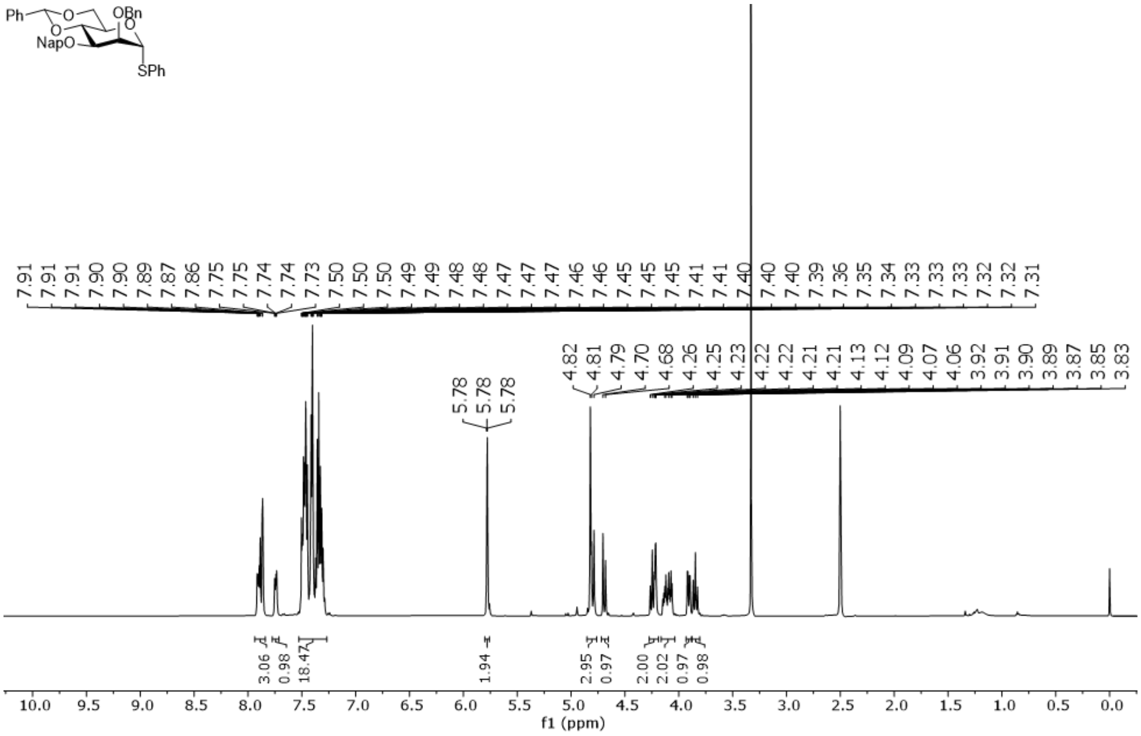

Figure S100: ^{1}H NMR spectrum.

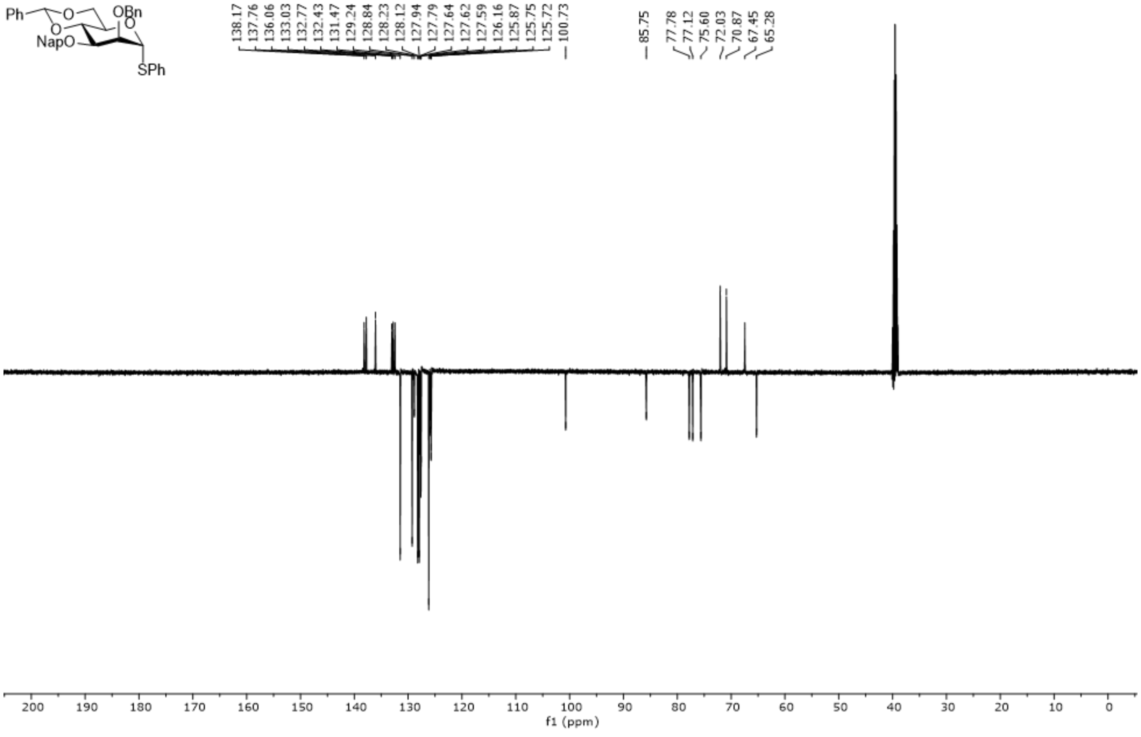

Figure S101: ^{13}C NMR spectrum.

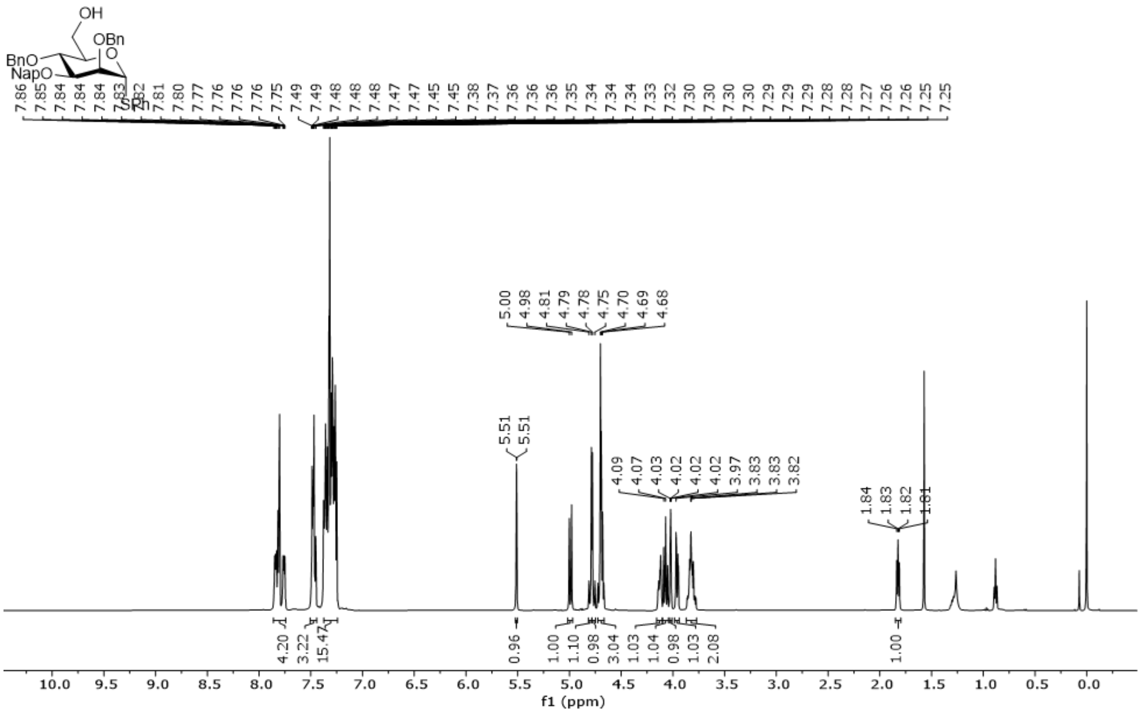

Figure S102: ^{1}H NMR spectrum.

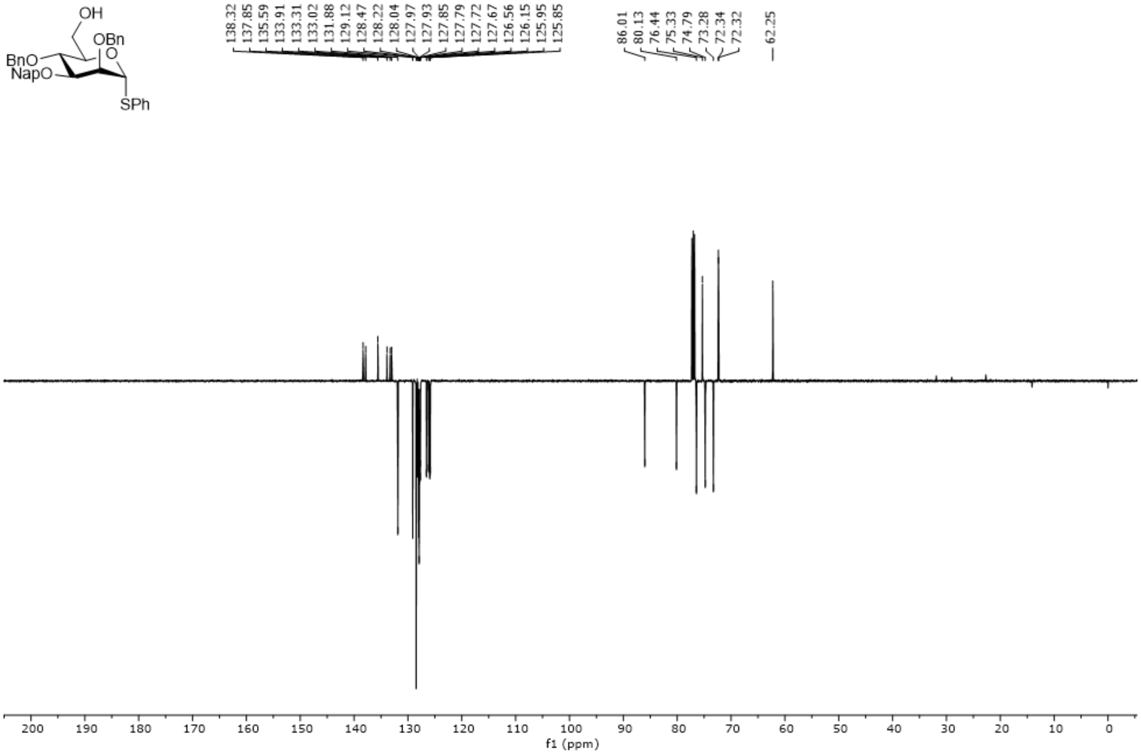

Figure S103: ^{13}C NMR spectrum.

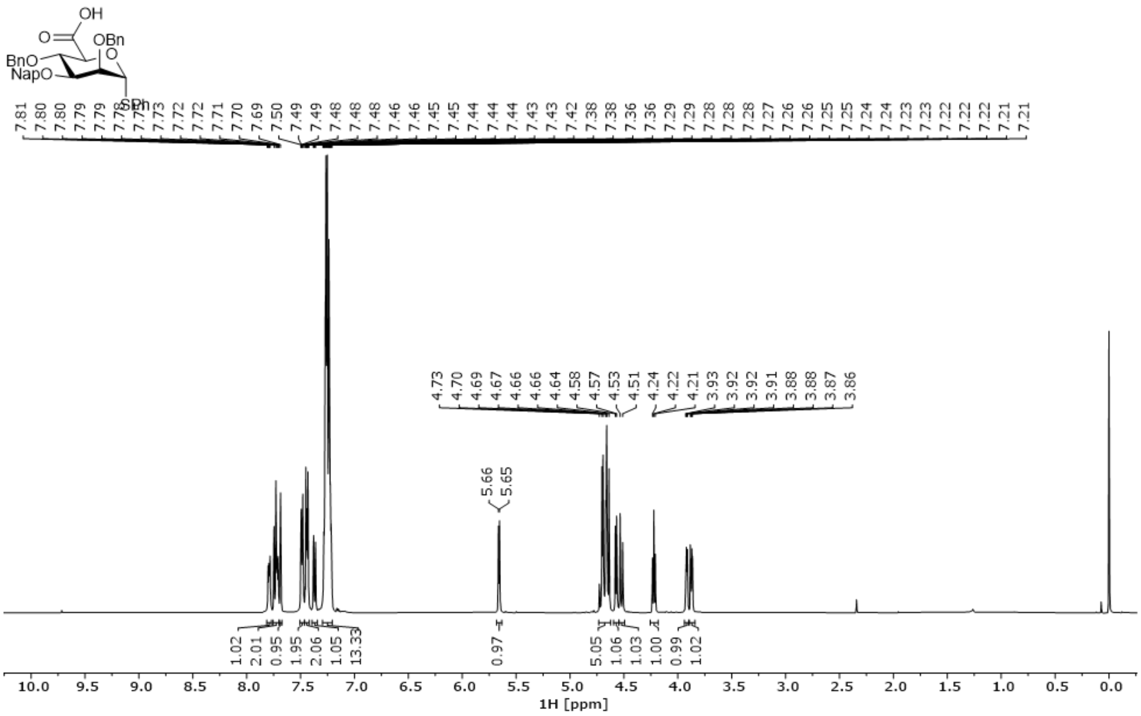

Figure S104: ^{1}H NMR spectrum.

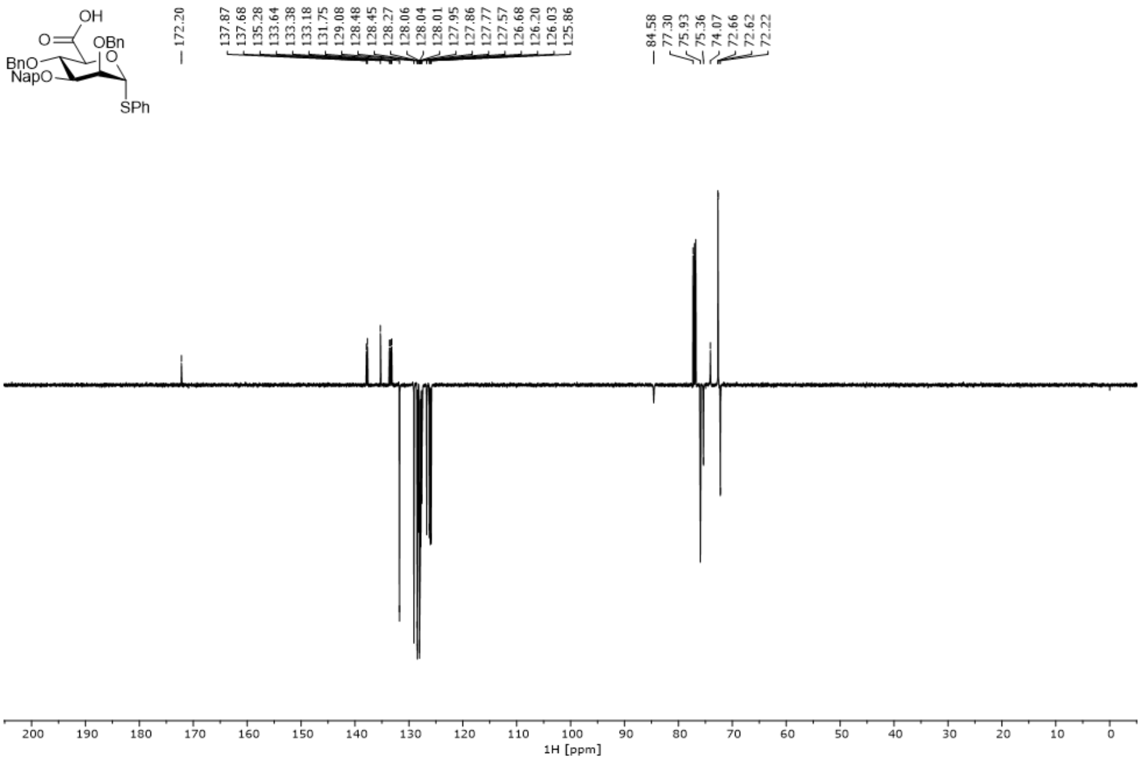

Figure S105: ^{13}C NMR spectrum.

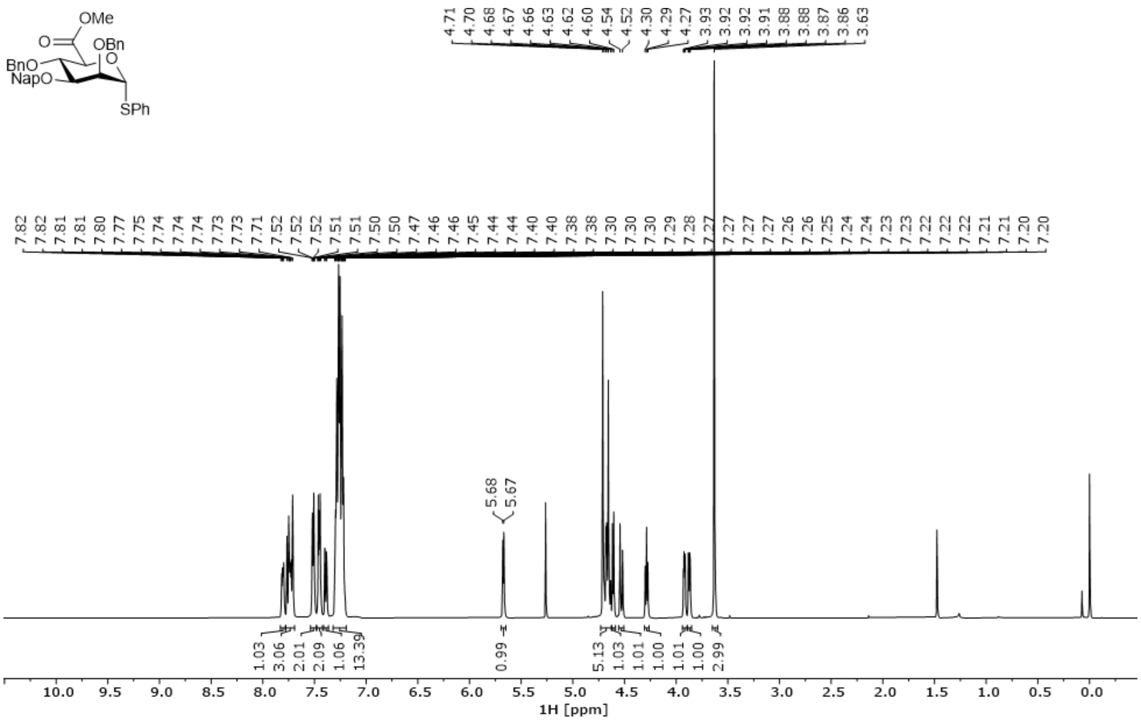

Figure S106: ^{1}H NMR spectrum.

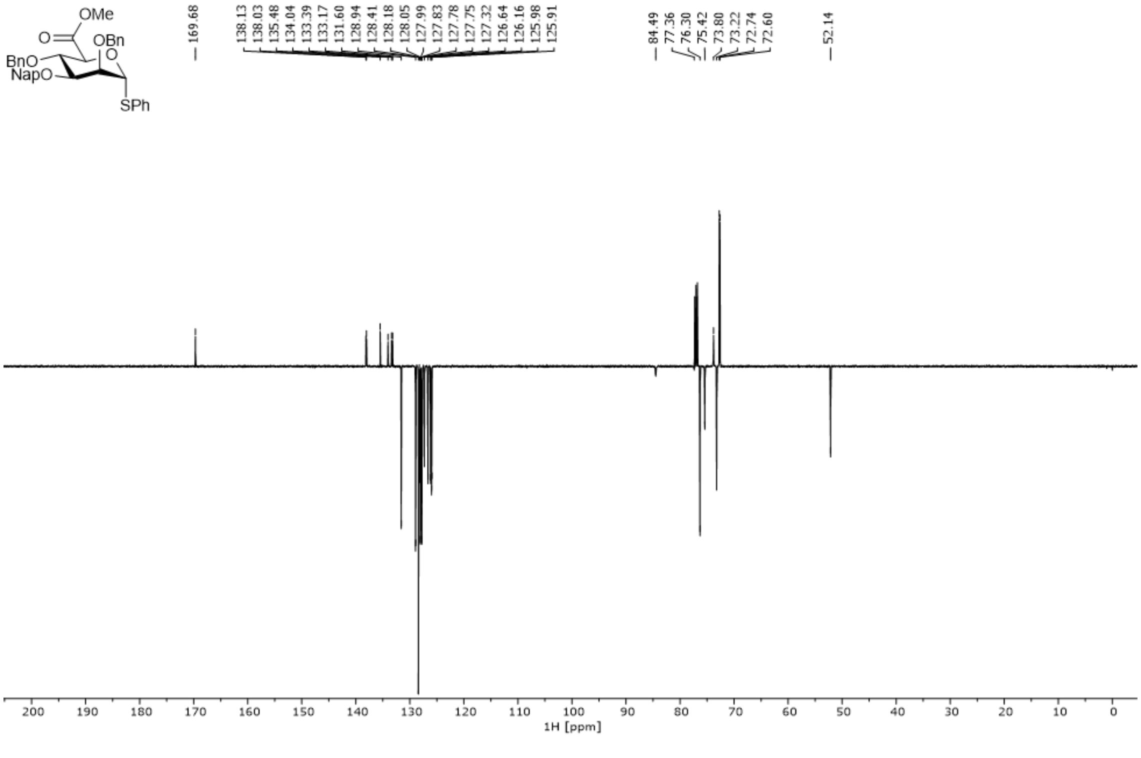

Figure S107: ^{13}C NMR spectrum.

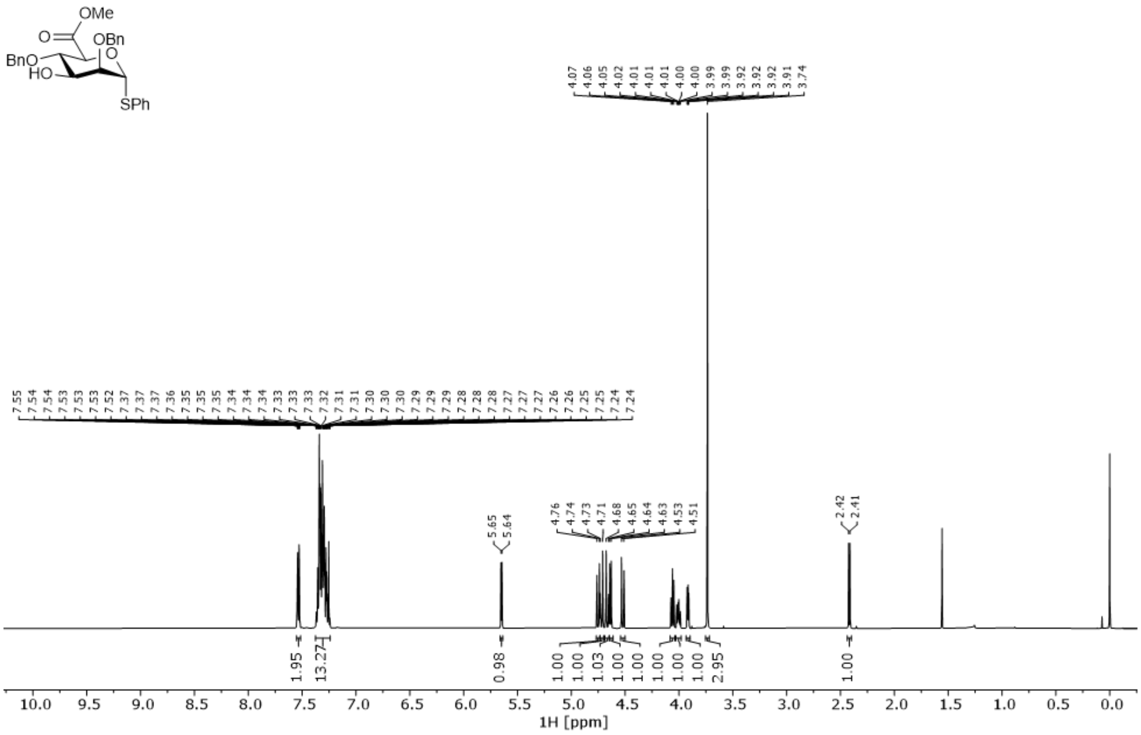

Figure S108: ^{1}H NMR spectrum.

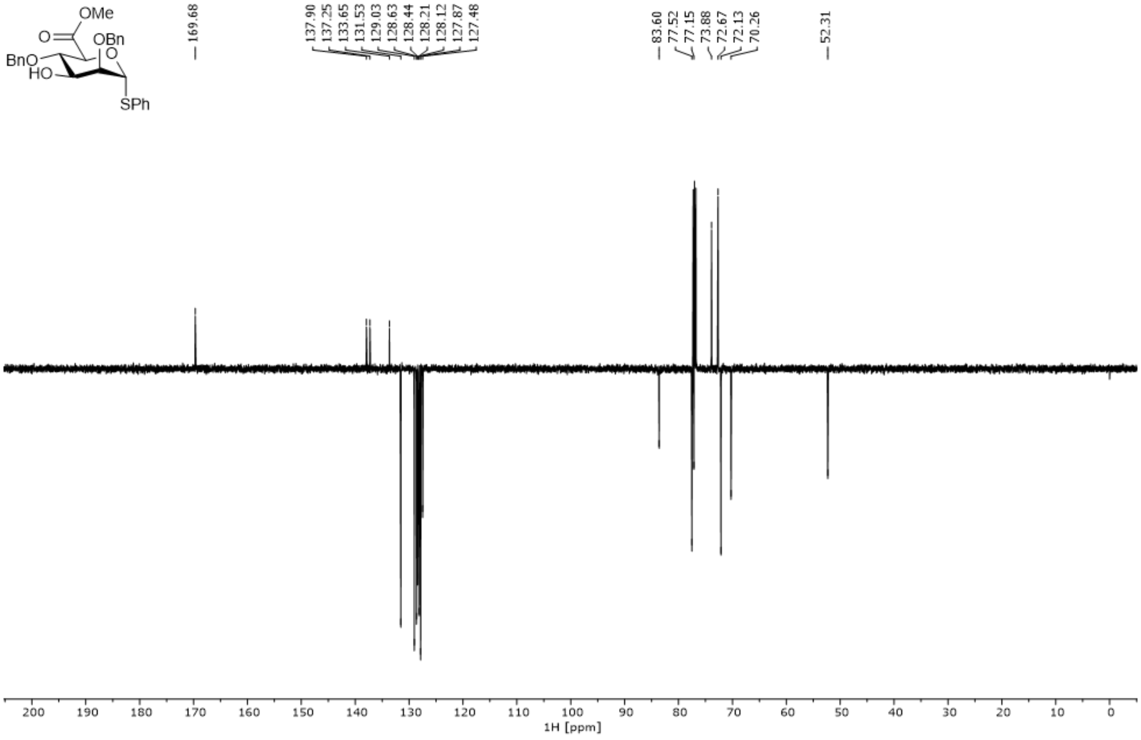

Figure S109: ^{13}C NMR spectrum.

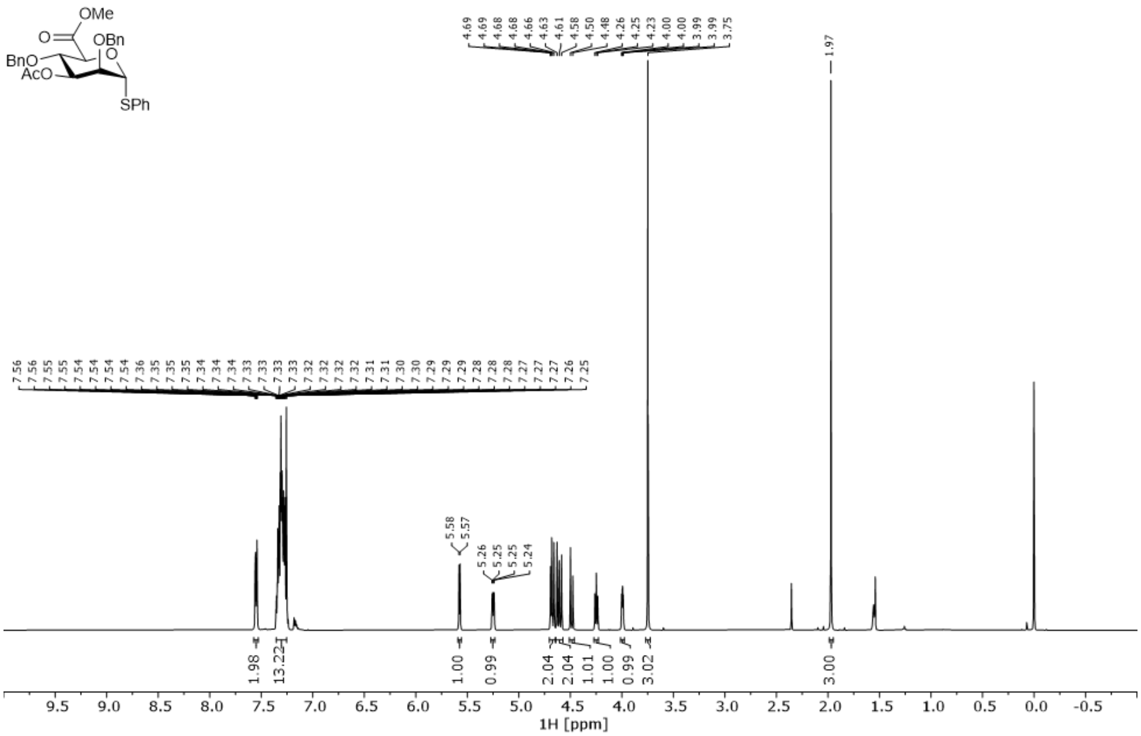

Figure S110: ^{1}H NMR spectrum.

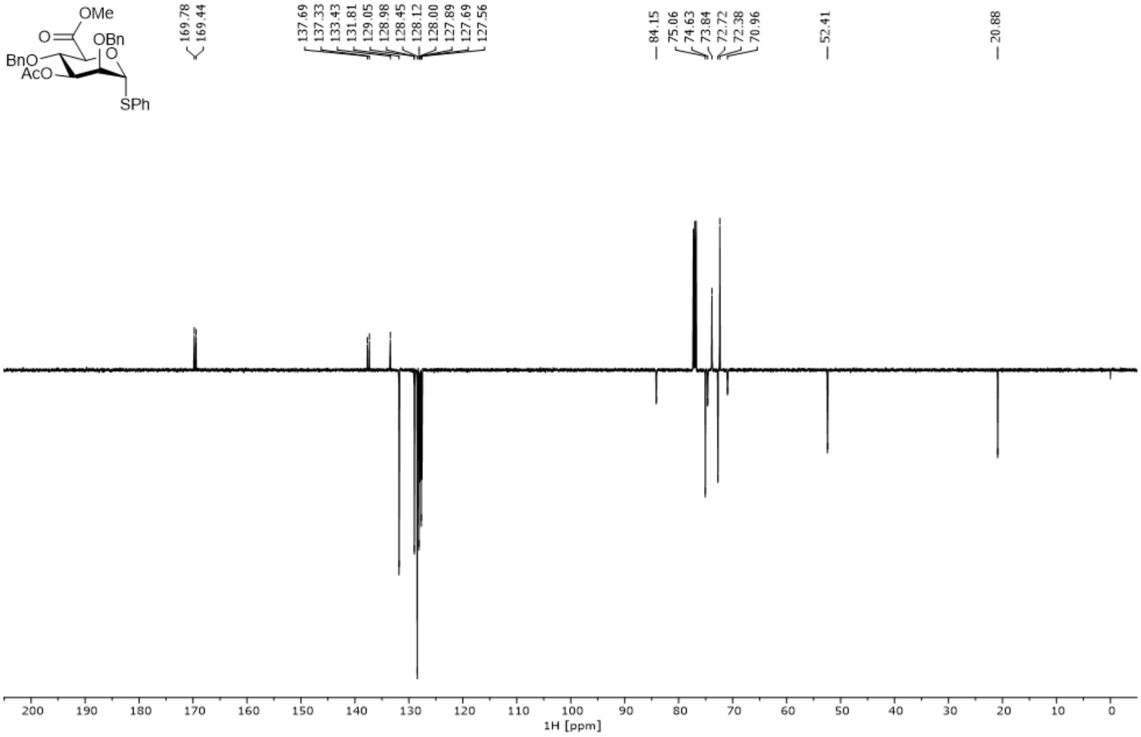

Figure S111: ^{13}C NMR spectrum.

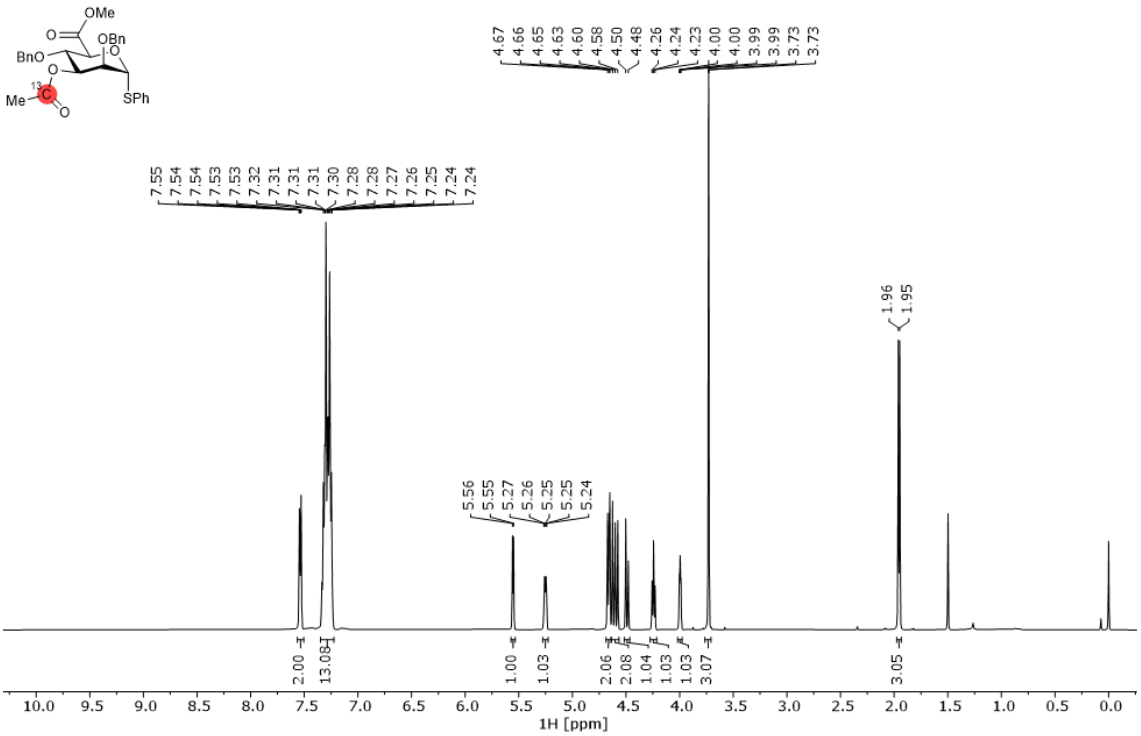

Figure S112: ^{1}H NMR spectrum.

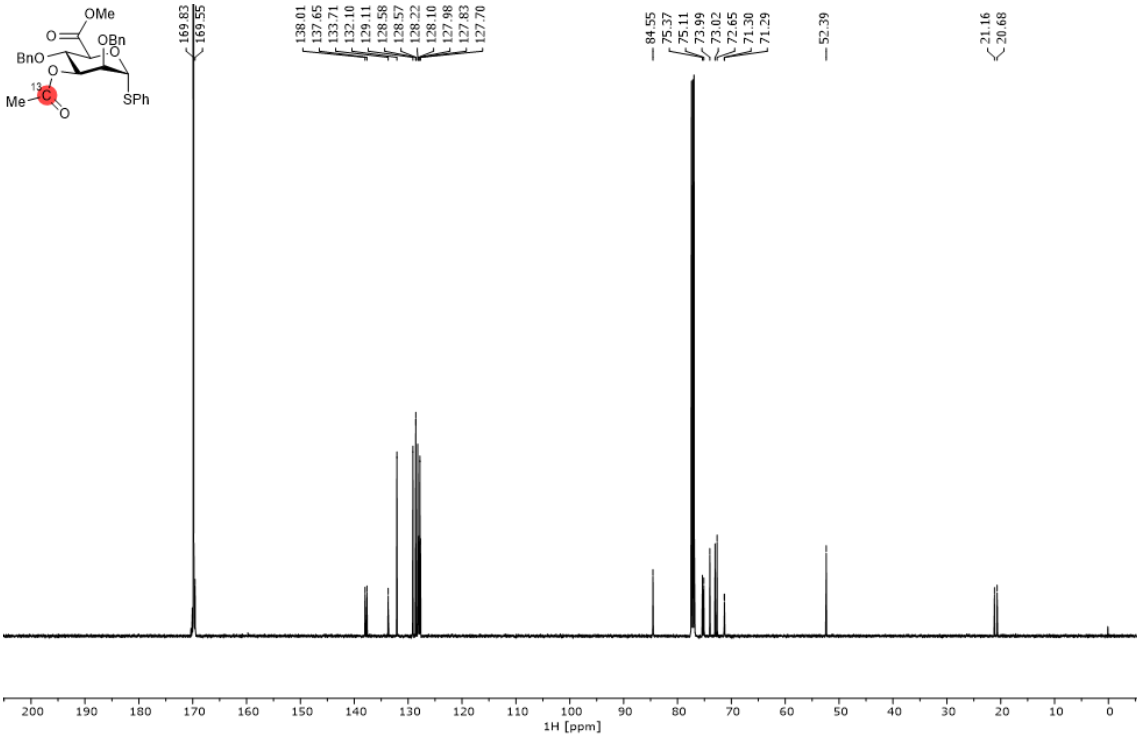

Figure S113: ^{13}C NMR spectrum.

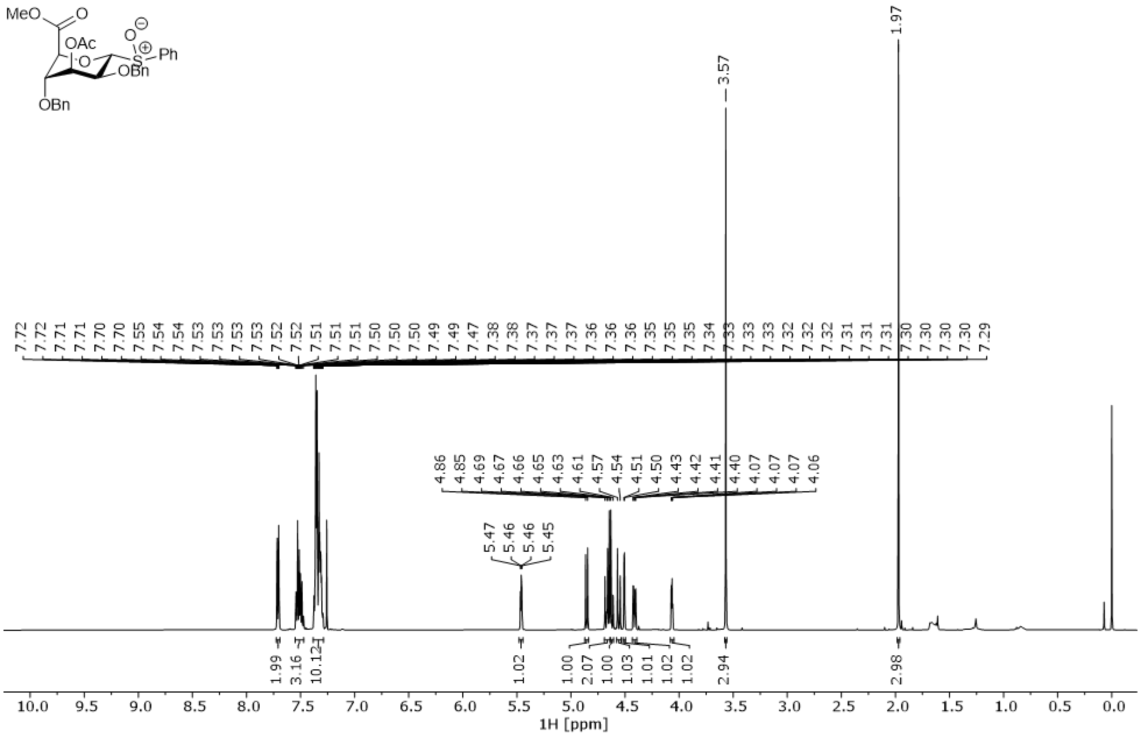

Figure S114: ^{1}H NMR spectrum.

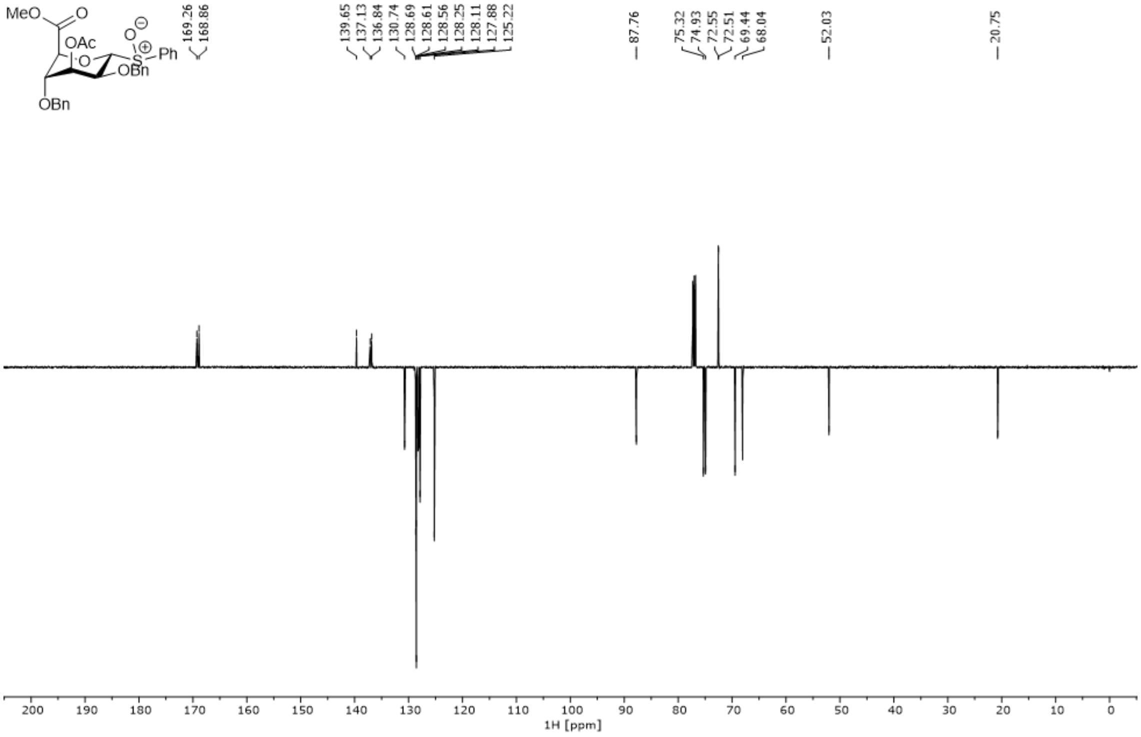

Figure S115: ^{13}C NMR spectrum.

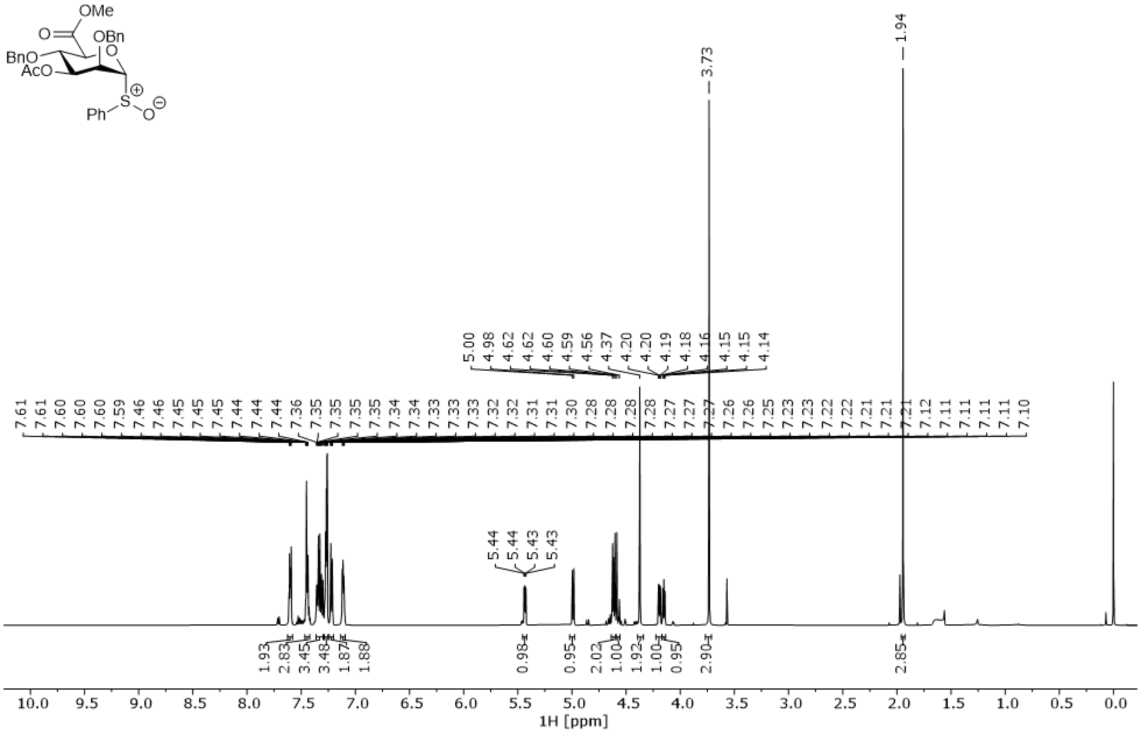

Figure S116: ^{1}H NMR spectrum.

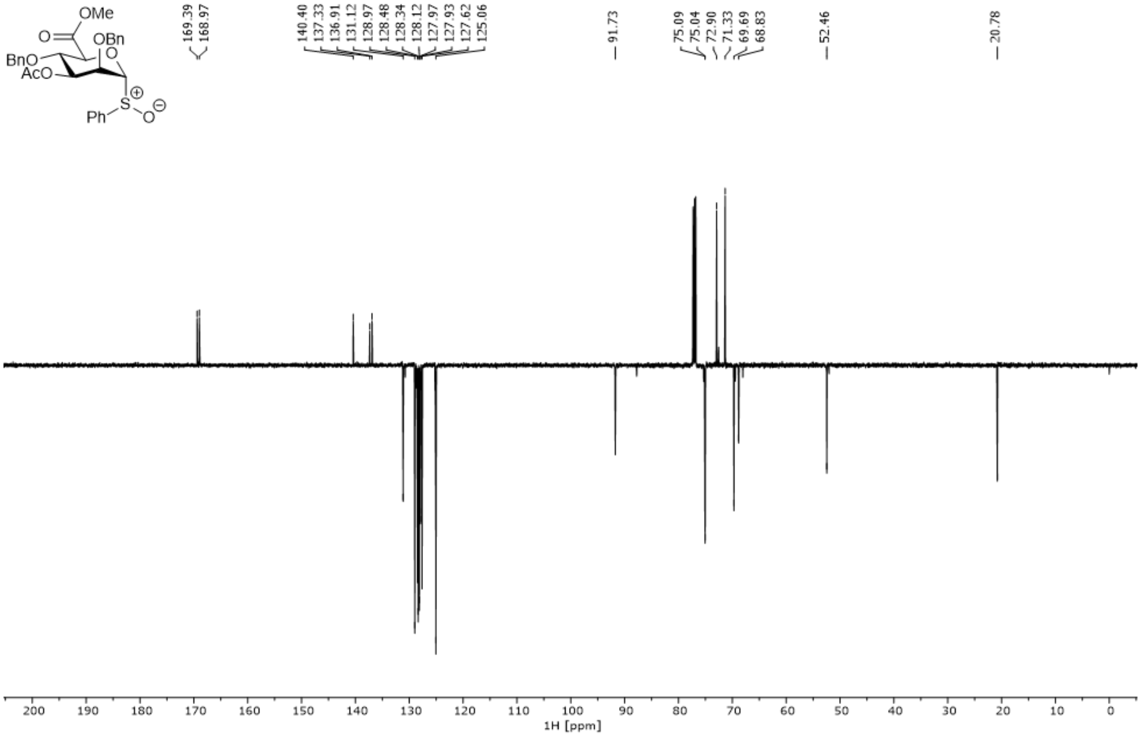

Figure S117: ^{13}C NMR spectrum.

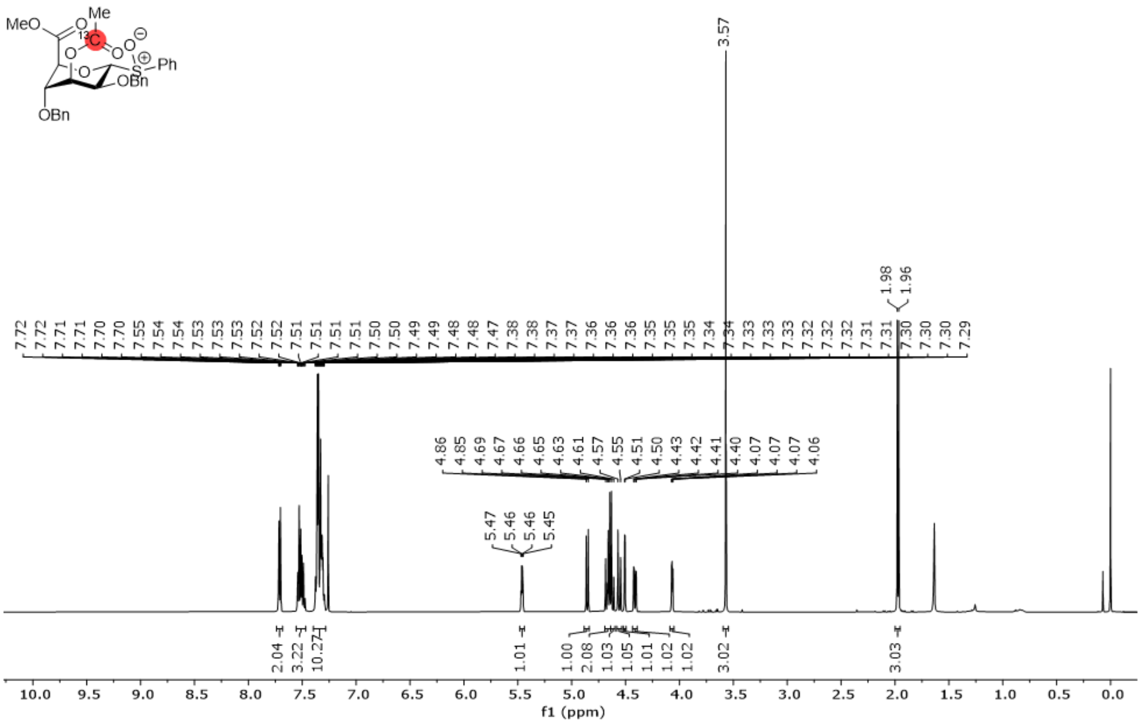

Figure S118: ^{1}H NMR spectrum.

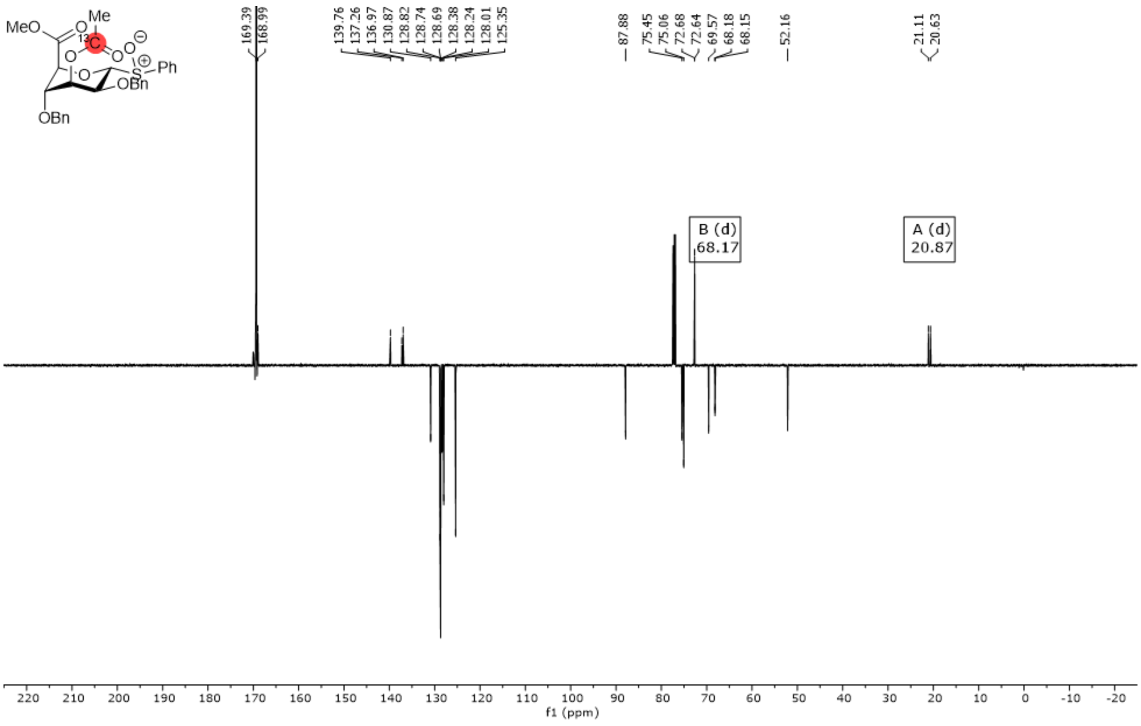

Figure S119: ^{13}C NMR spectrum.

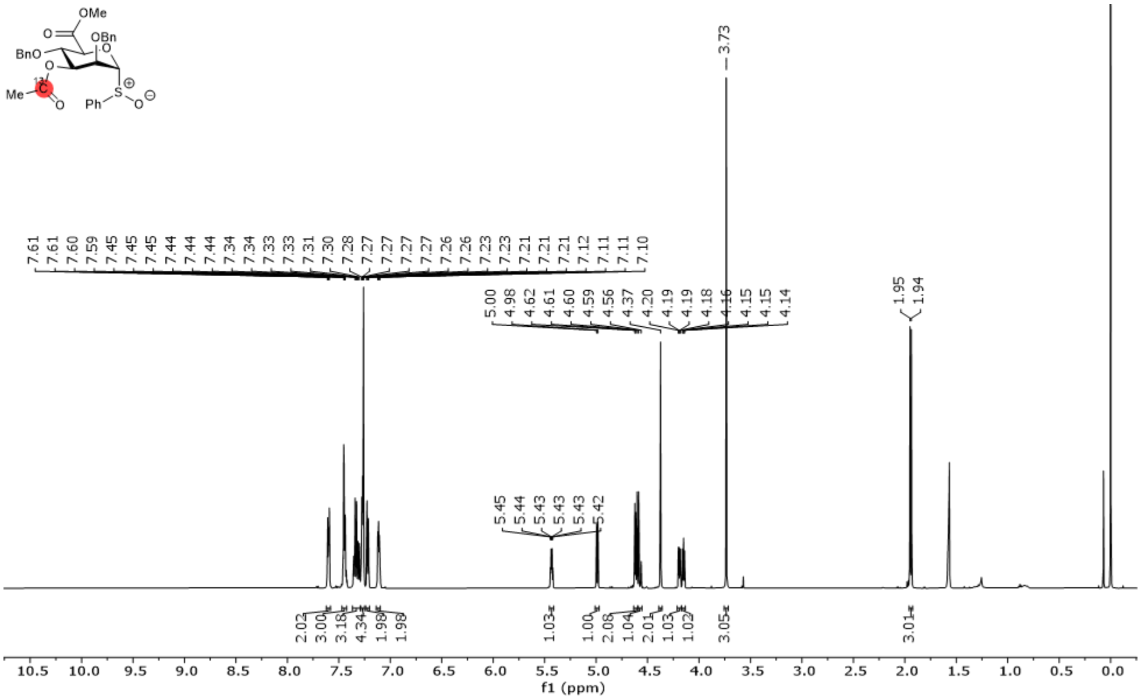

Figure S120: ^{1}H NMR spectrum.

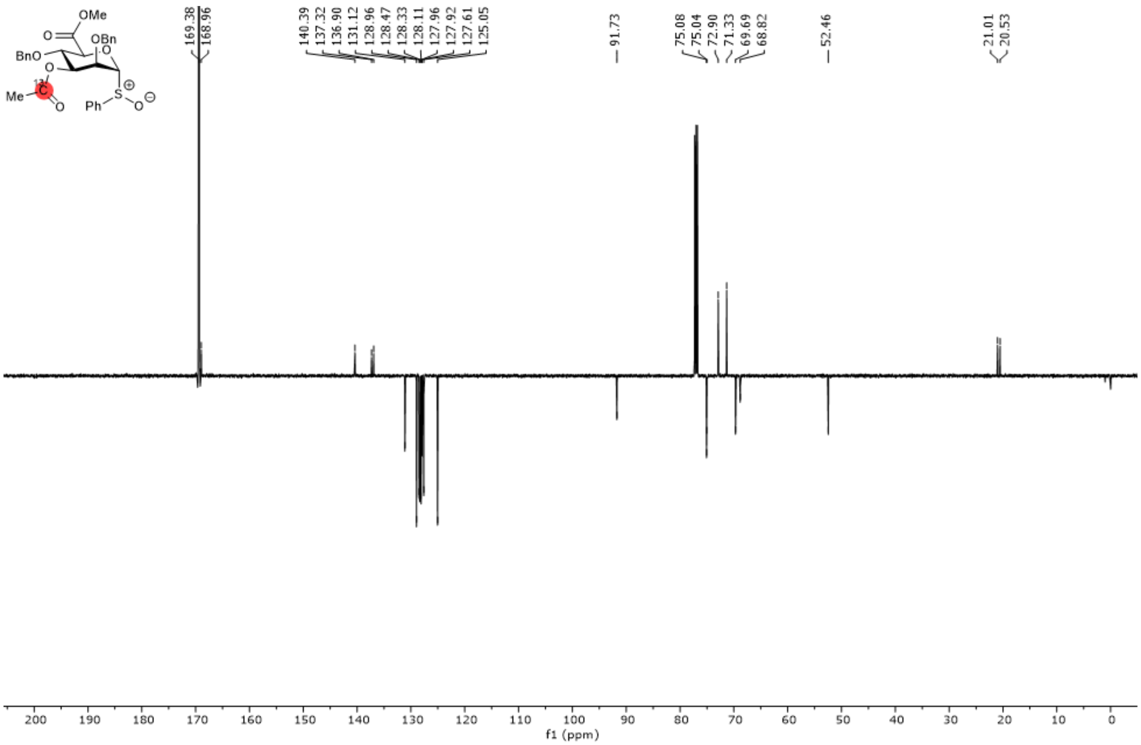

Figure S121: ^{13}C NMR spectrum.

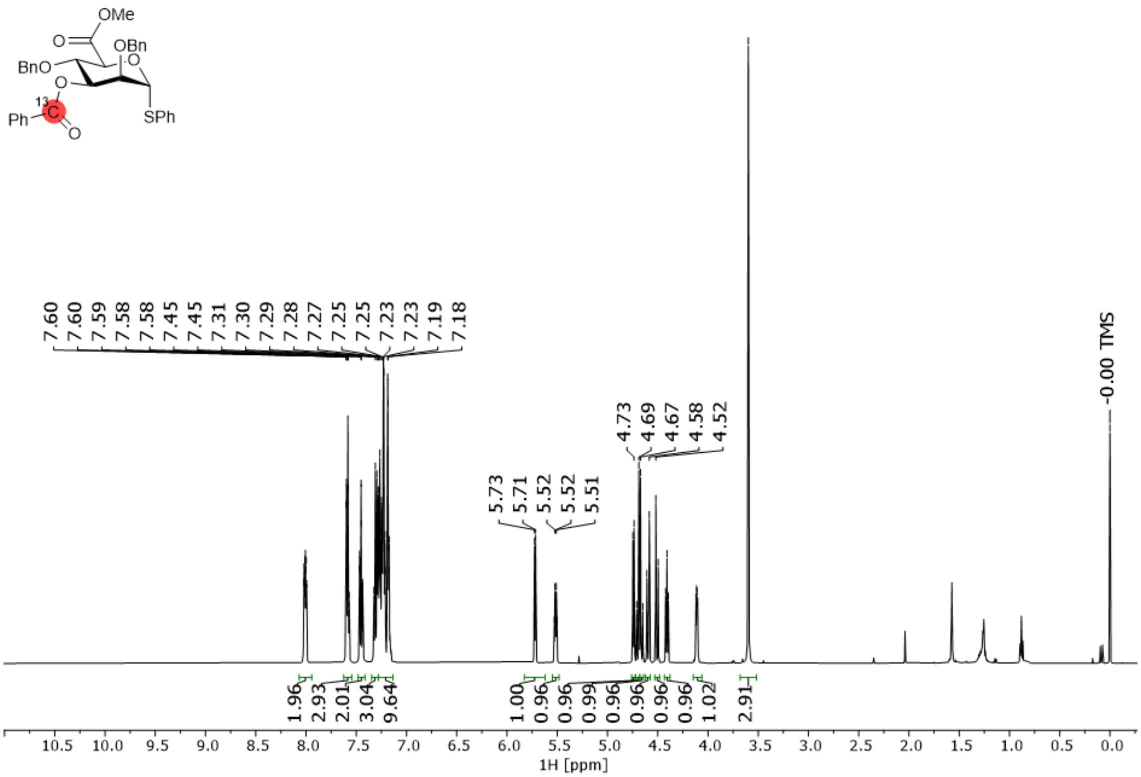

Figure S122: ^{1}H NMR spectrum.

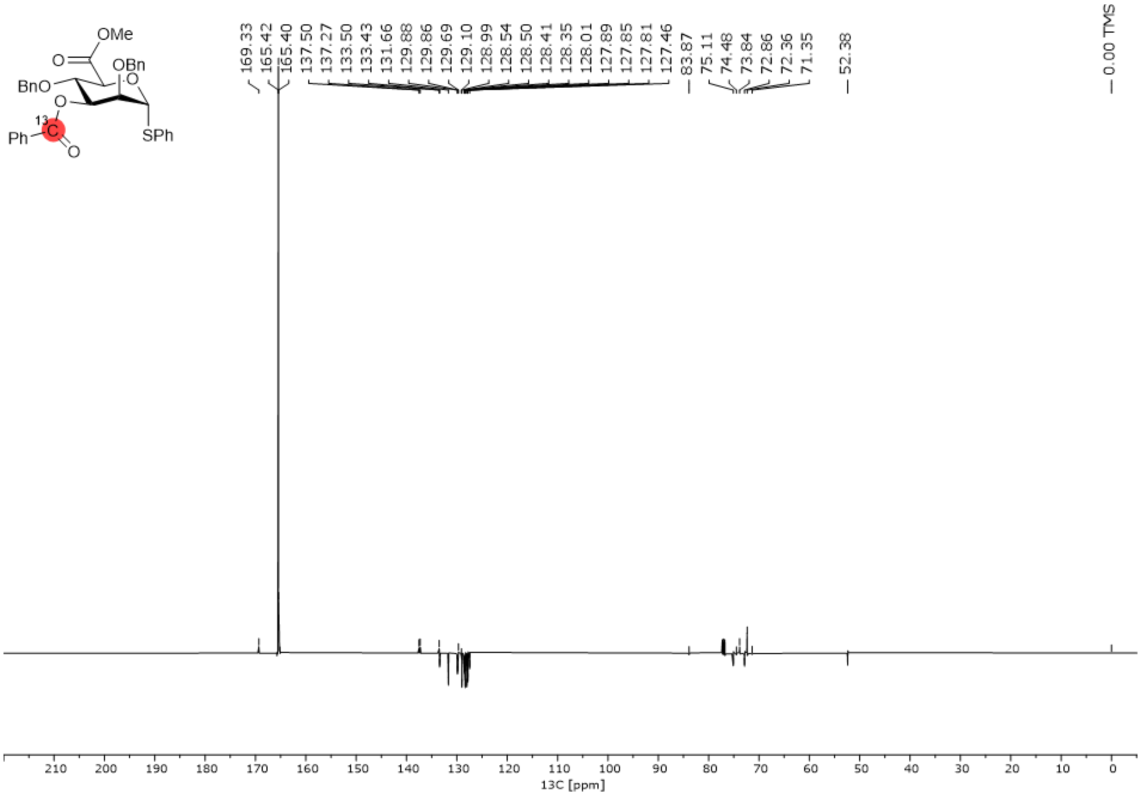

Figure S123: ^{13}C NMR spectrum.

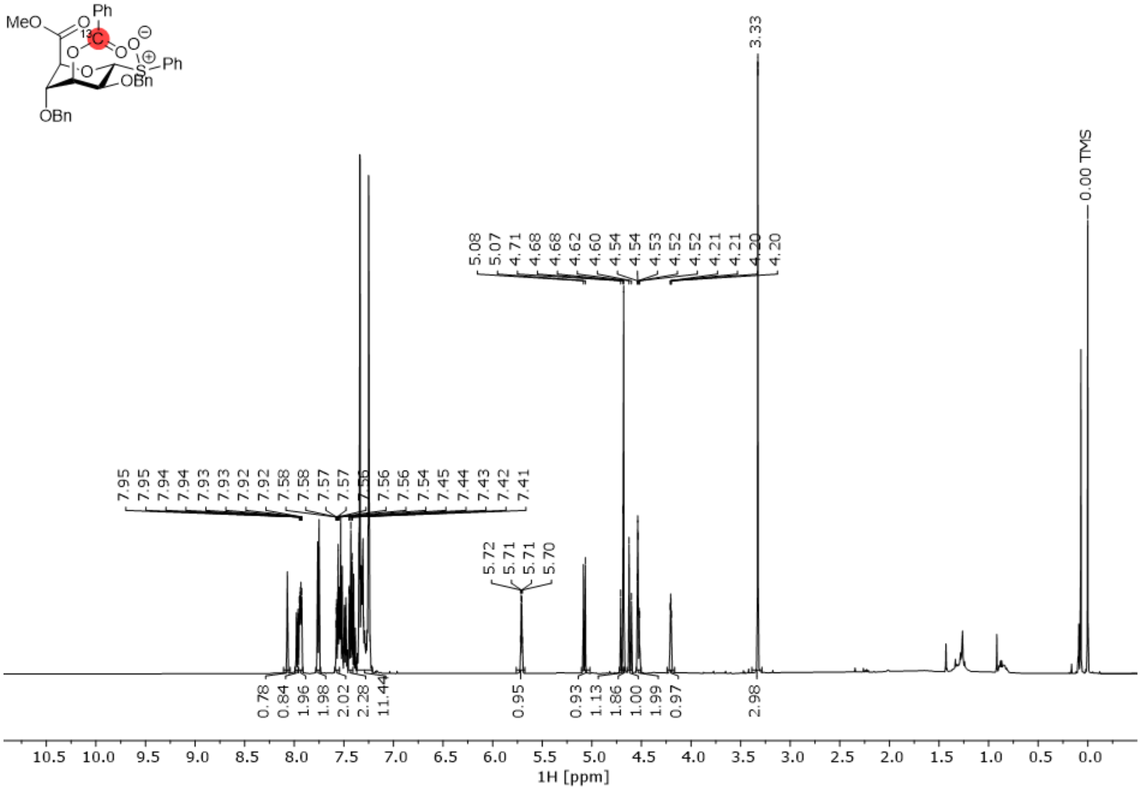

Figure S124: ^{1}H NMR spectrum.

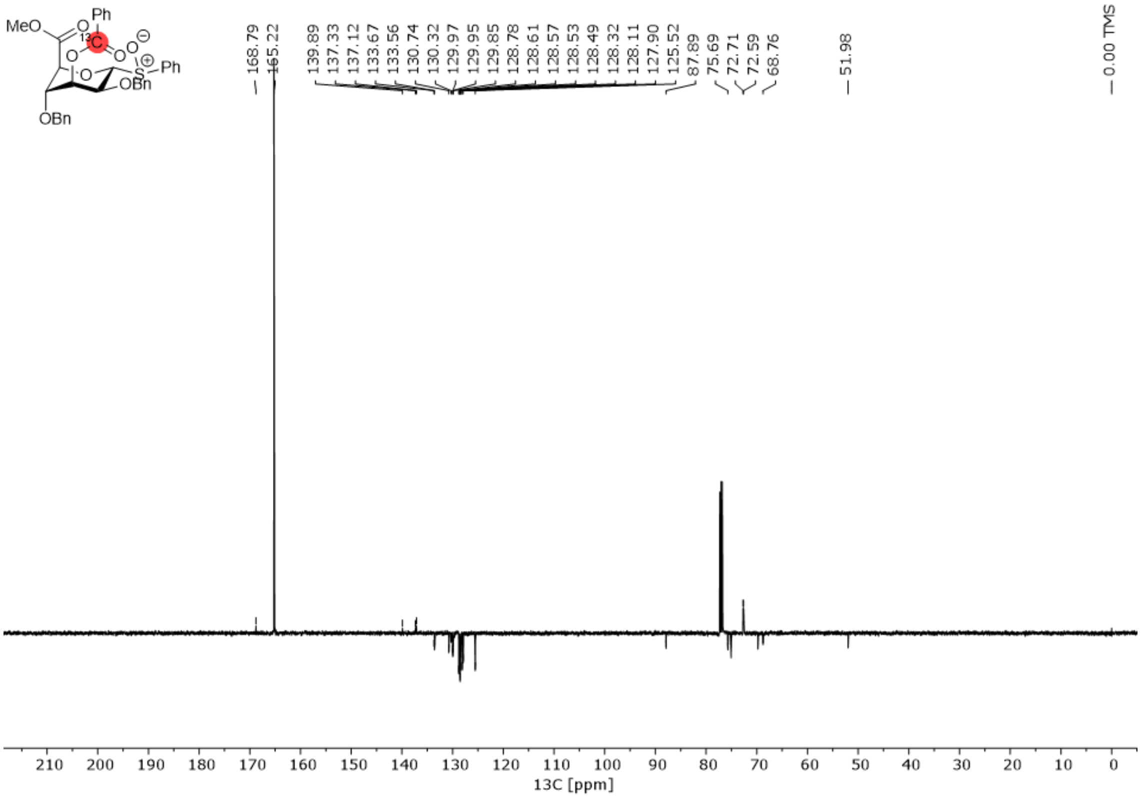

Figure S125: ^{13}C NMR spectrum.

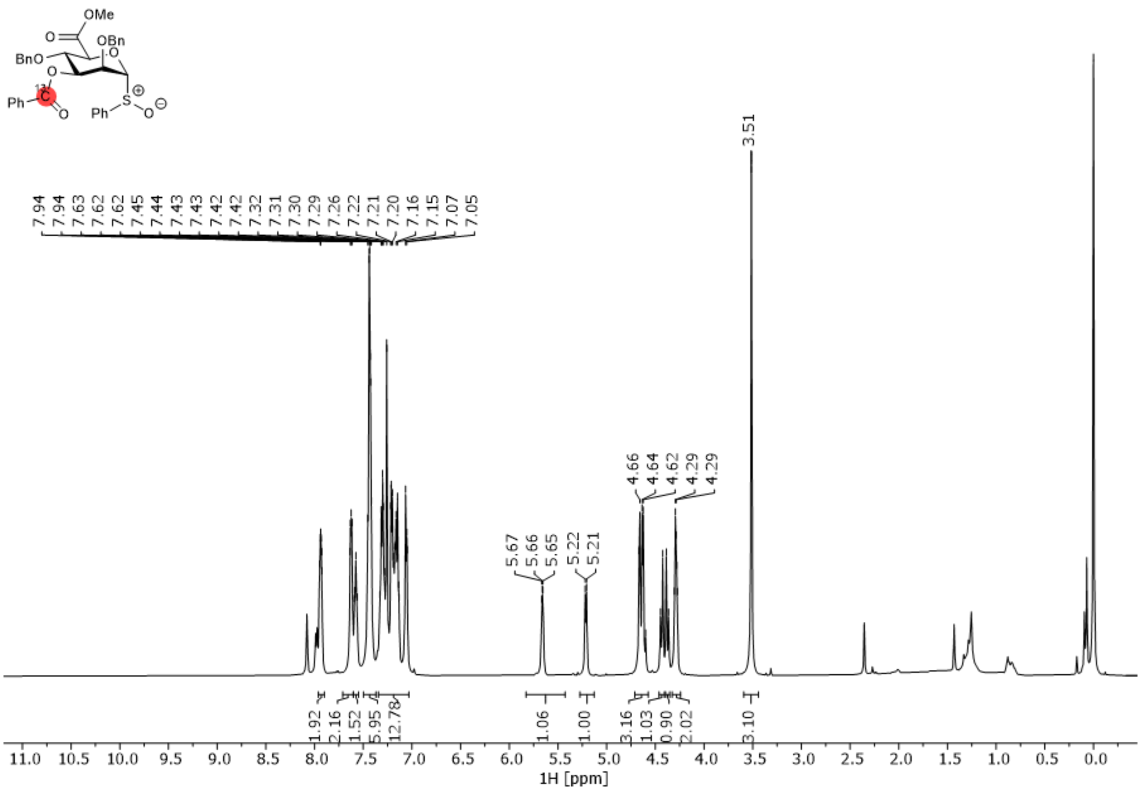

Figure S126: ^{1}H NMR spectrum.

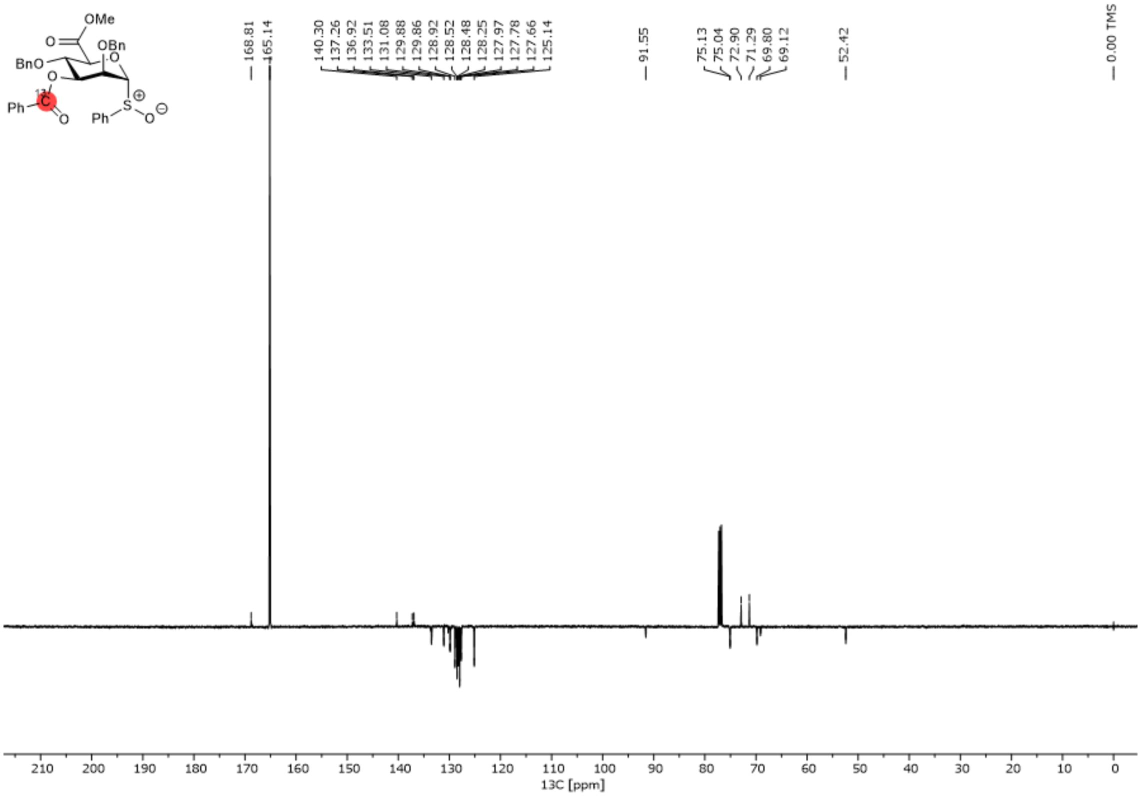

Figure S127: ^{13}C NMR spectrum.

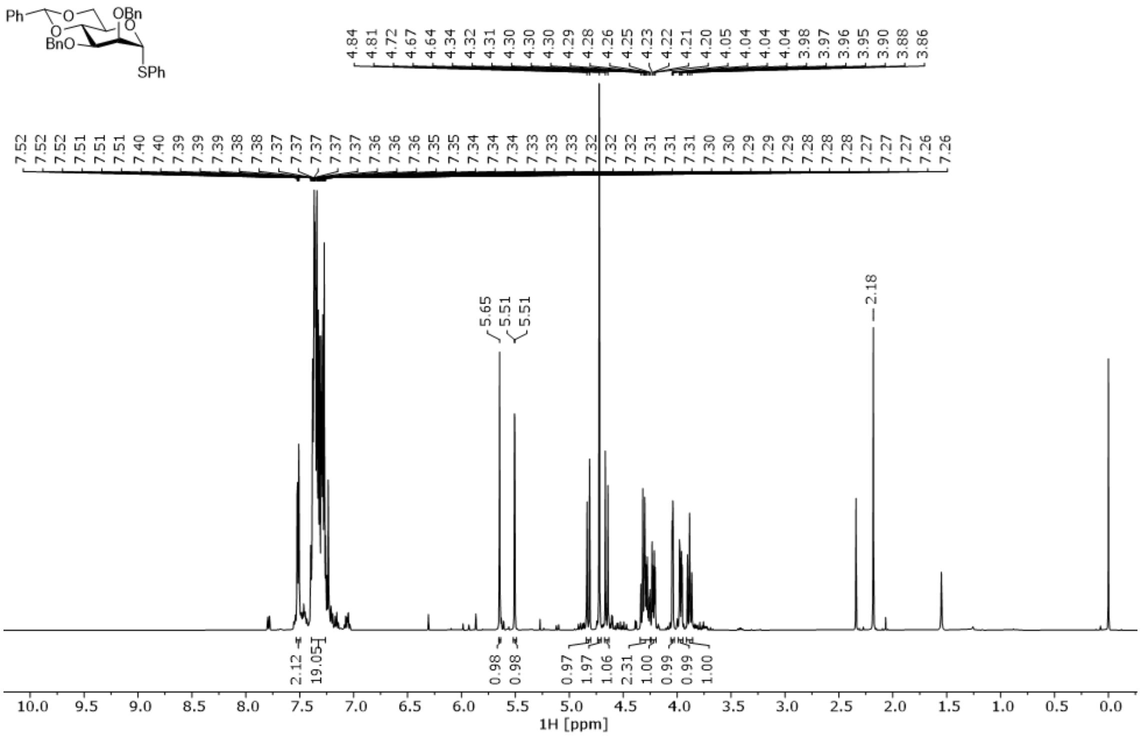

Figure S128: ^{1}H NMR spectrum.

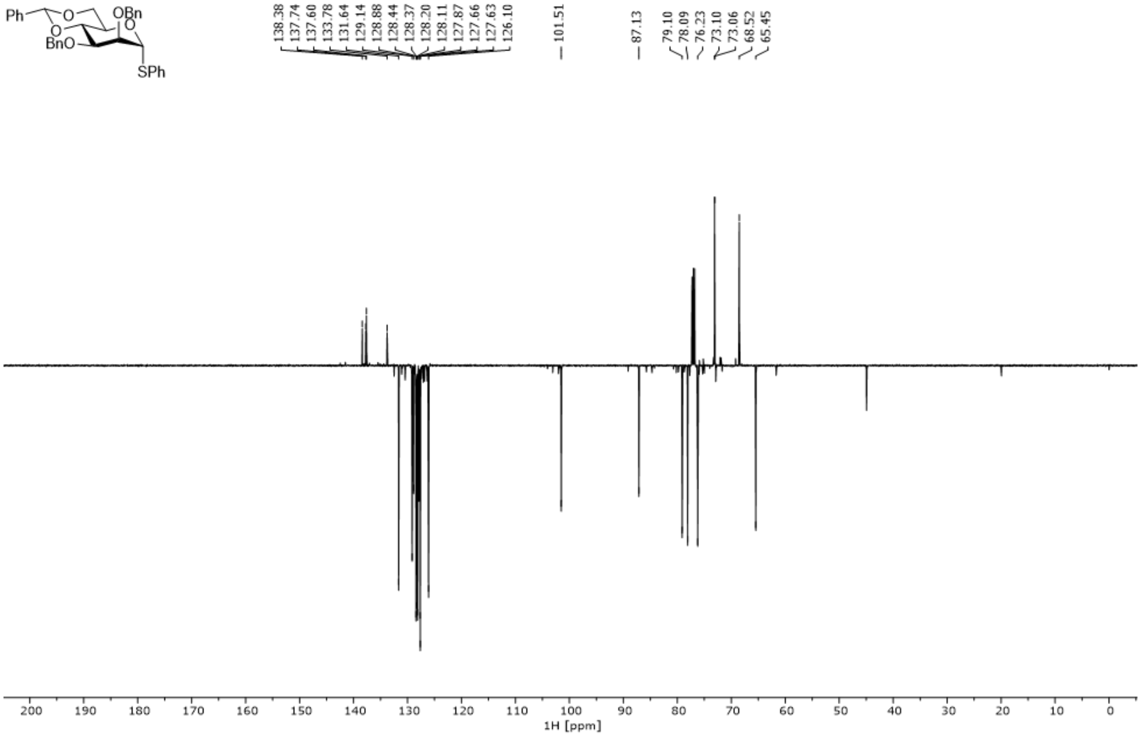

Figure S129: ^{13}C NMR spectrum.

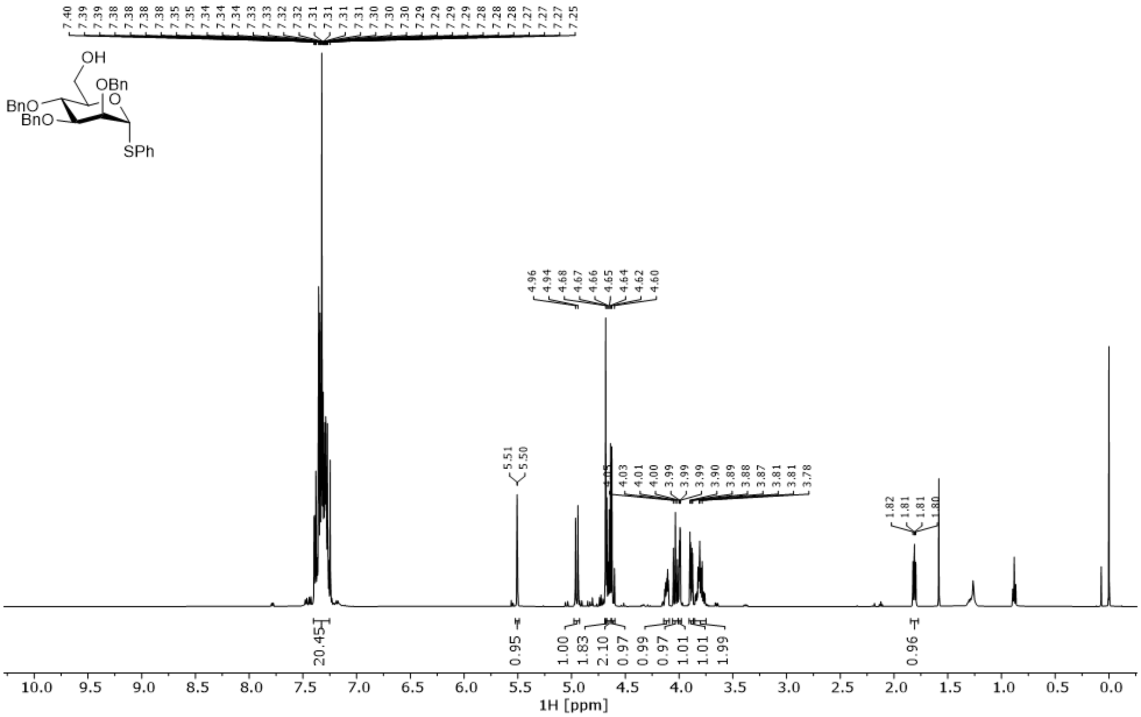

Figure S130: ^{1}H NMR spectrum.

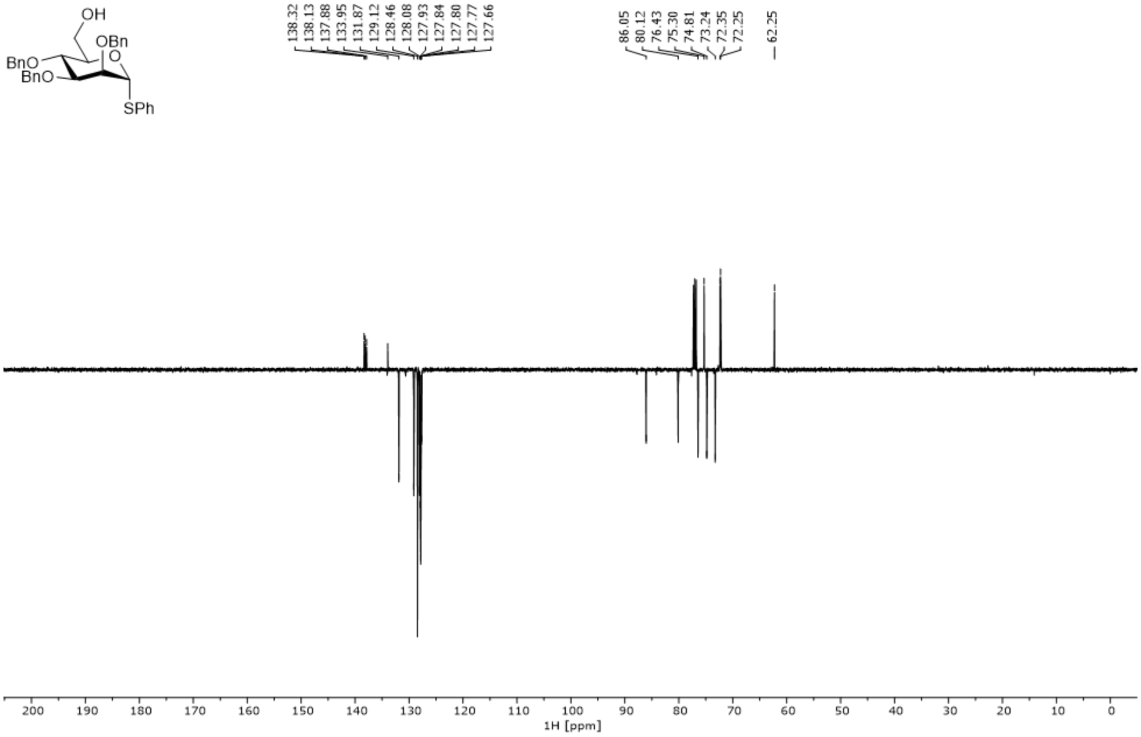

Figure S131: ^{13}C NMR spectrum.

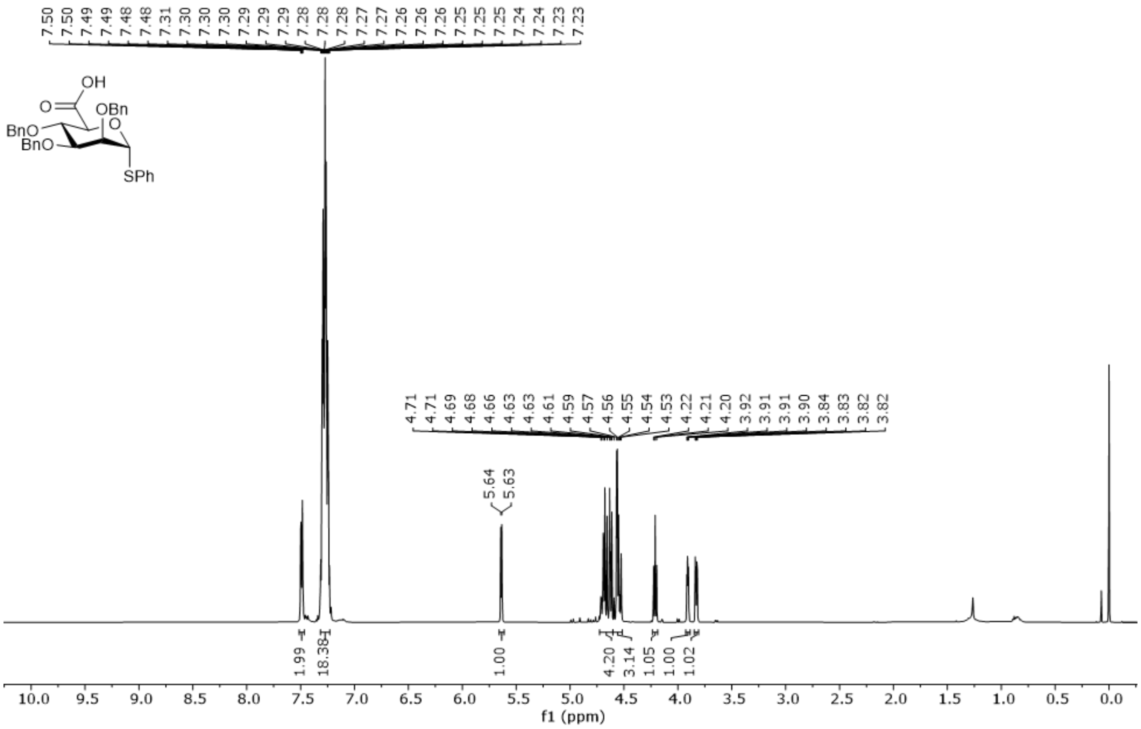

Figure S132: ^{1}H NMR spectrum.

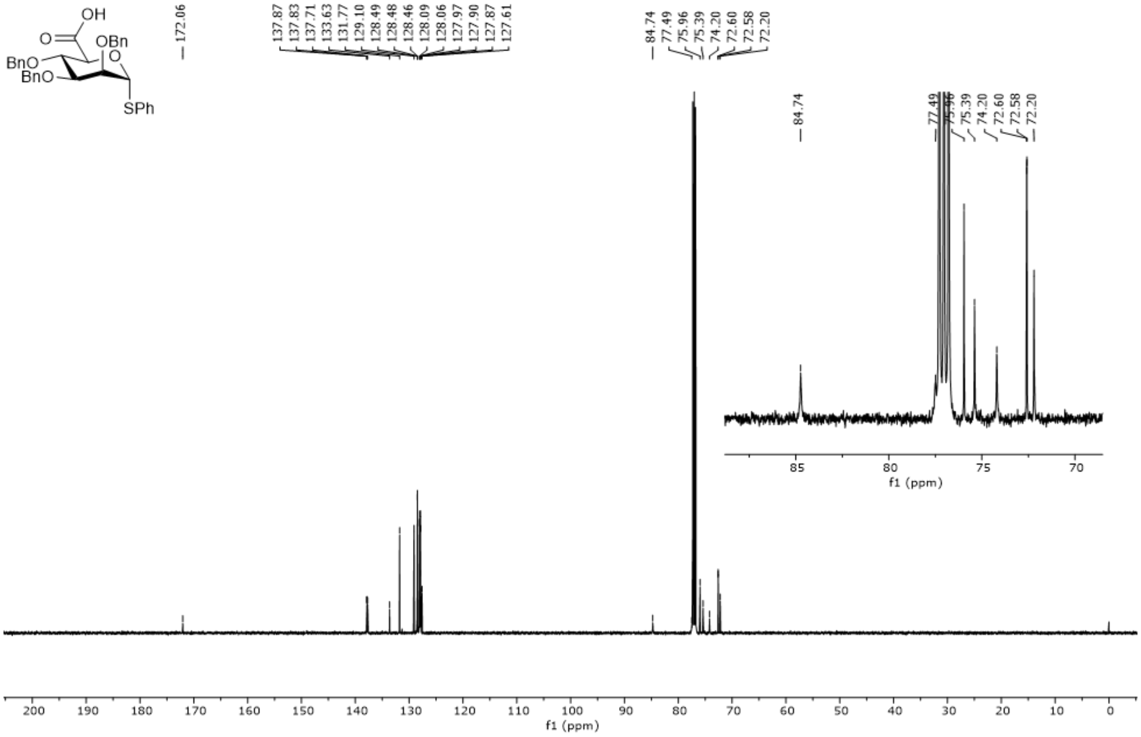

Figure S133: ^{13}C NMR spectrum.

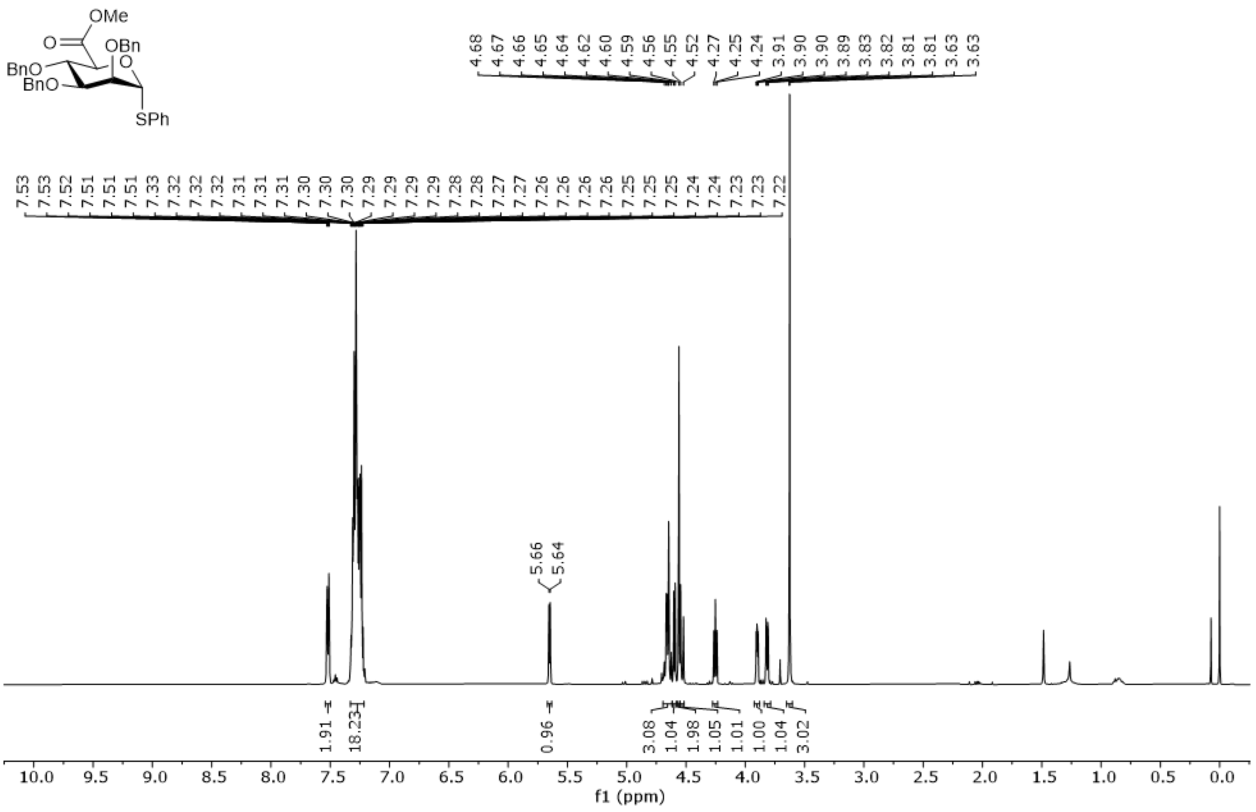

Figure S134: ^{1}H NMR spectrum.

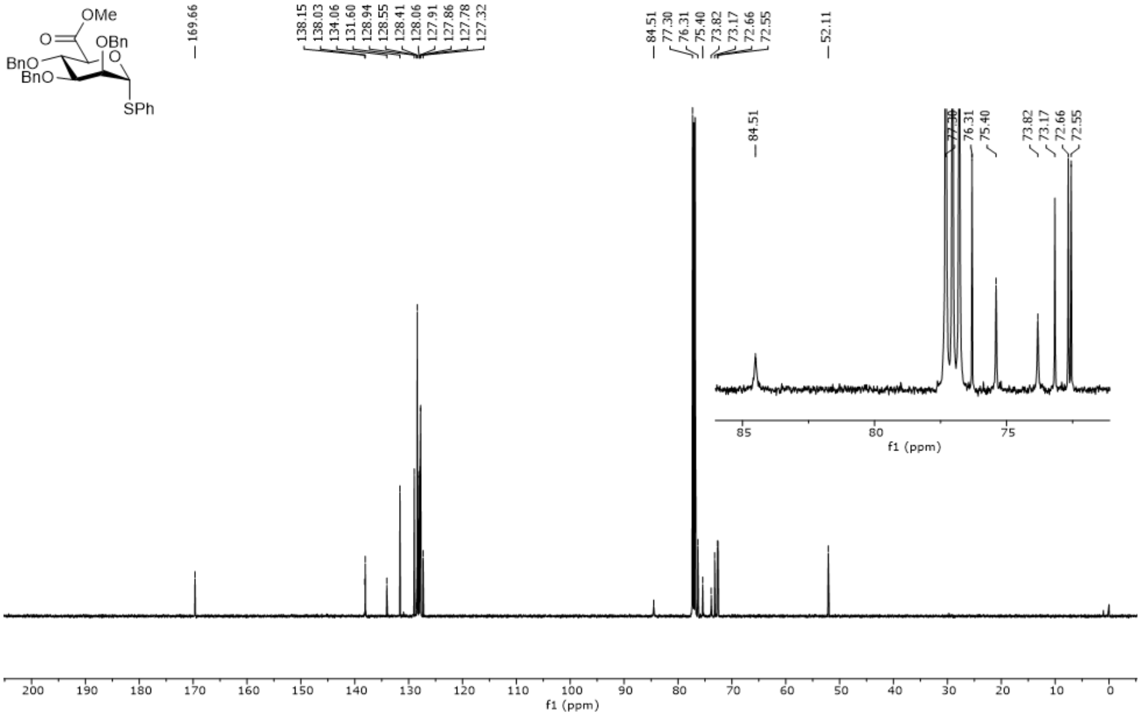

Figure S135: ^{13}C NMR spectrum.

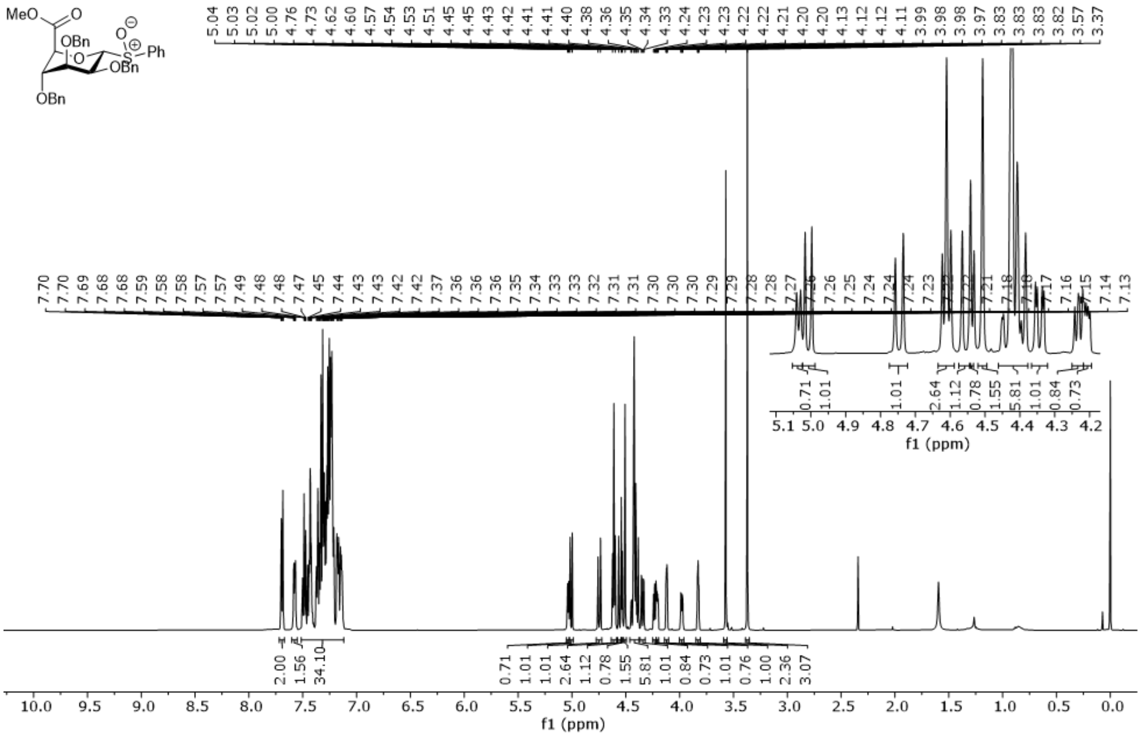

Figure S136: ^{1}H NMR spectrum.

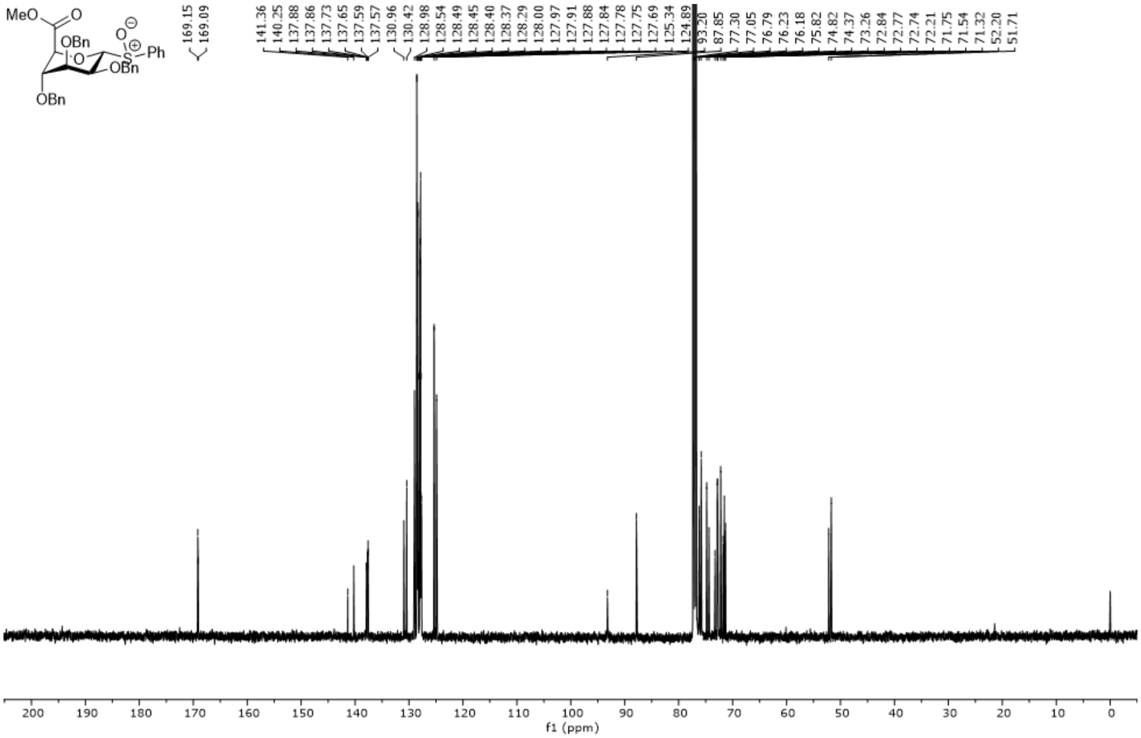

Figure S137: ^{13}C NMR spectrum.

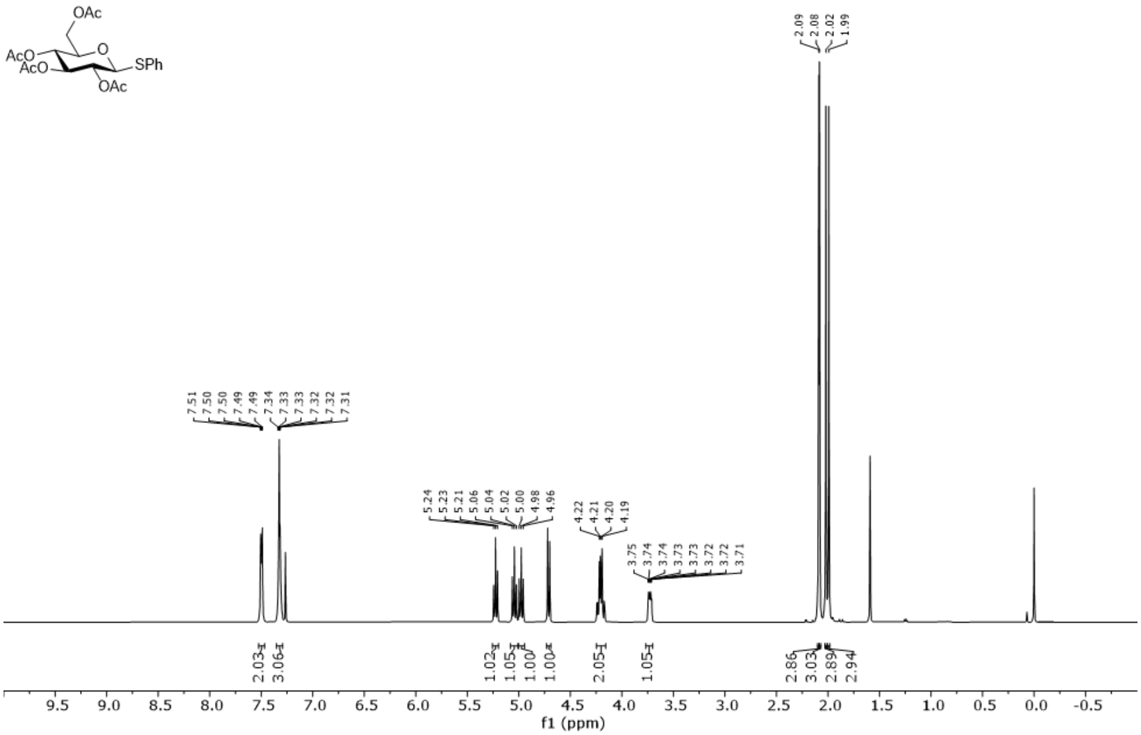

Figure S138: ^{1}H NMR spectrum.

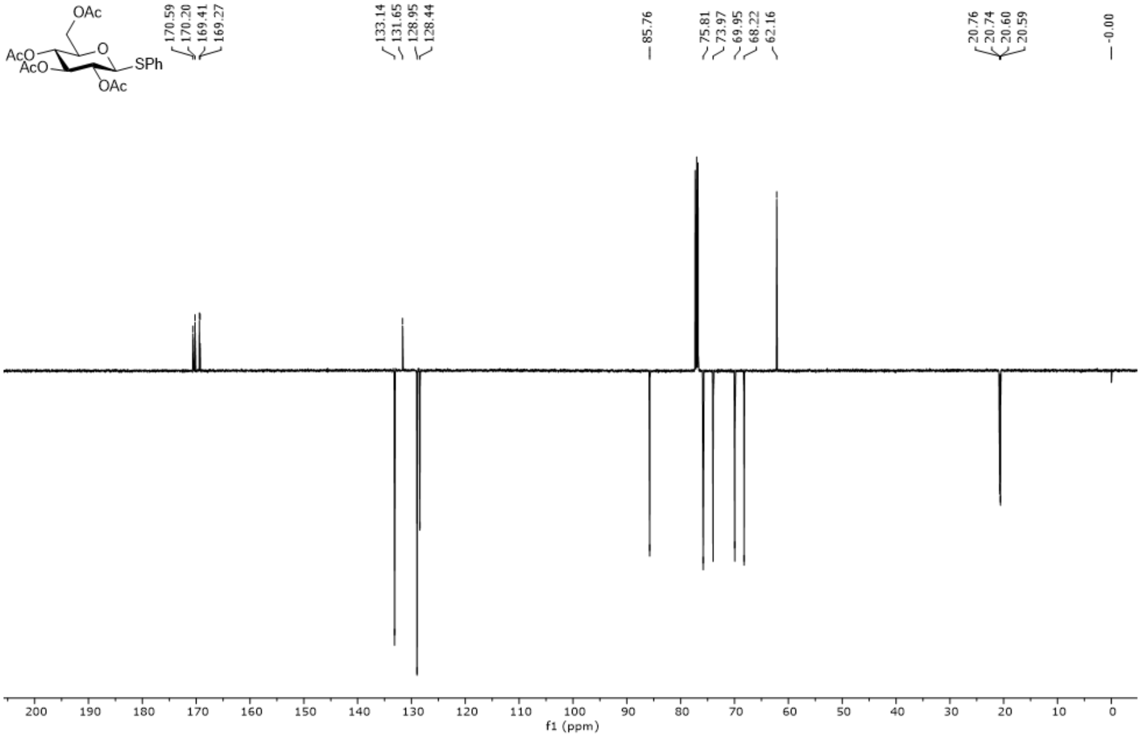

Figure S139: ^{13}C NMR spectrum.

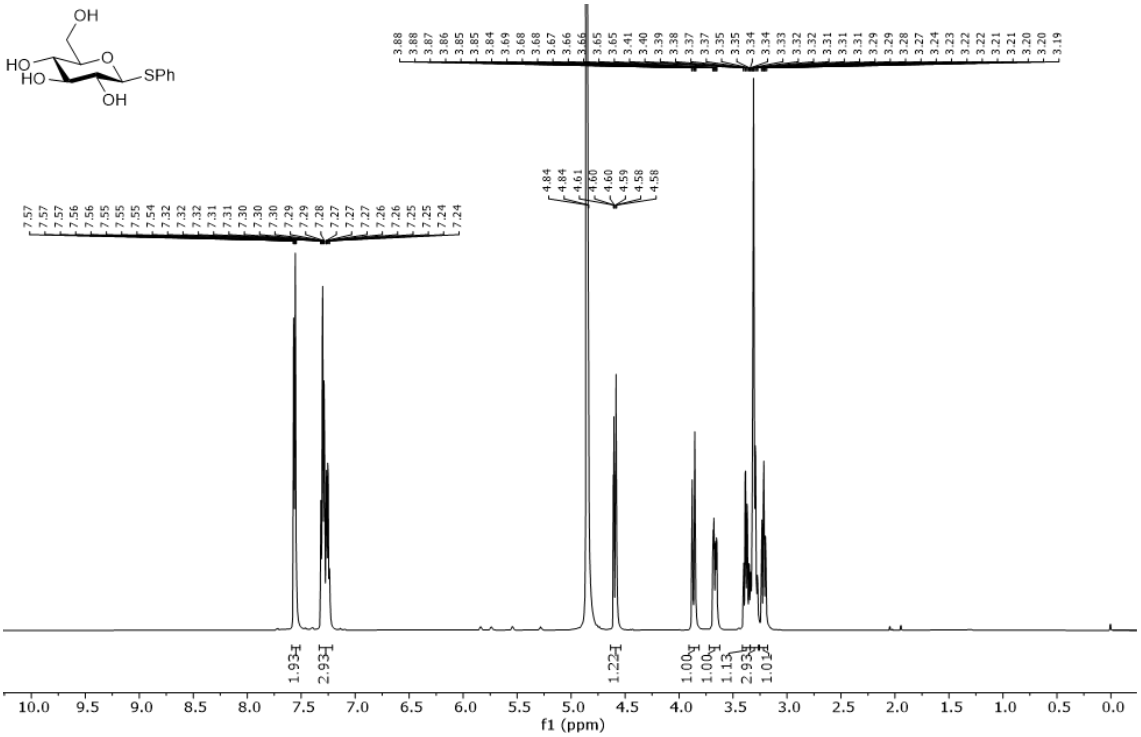

Figure S140: ^{1}H NMR spectrum.

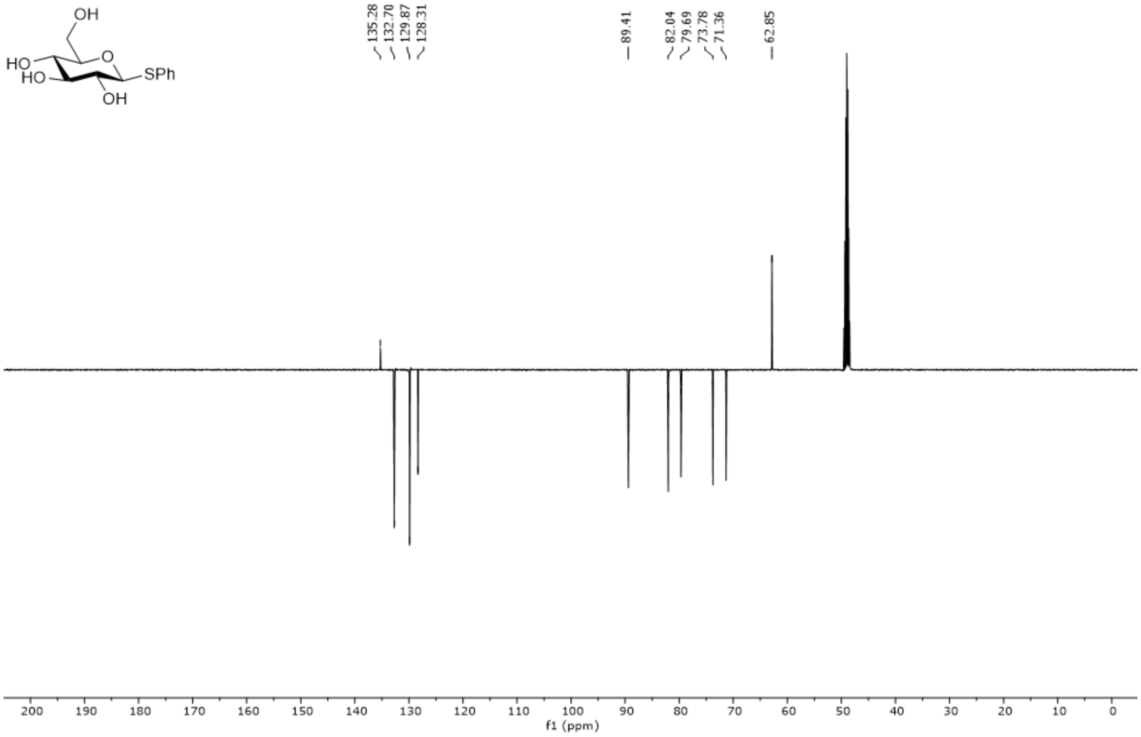

Figure S141: ^{13}C NMR spectrum.

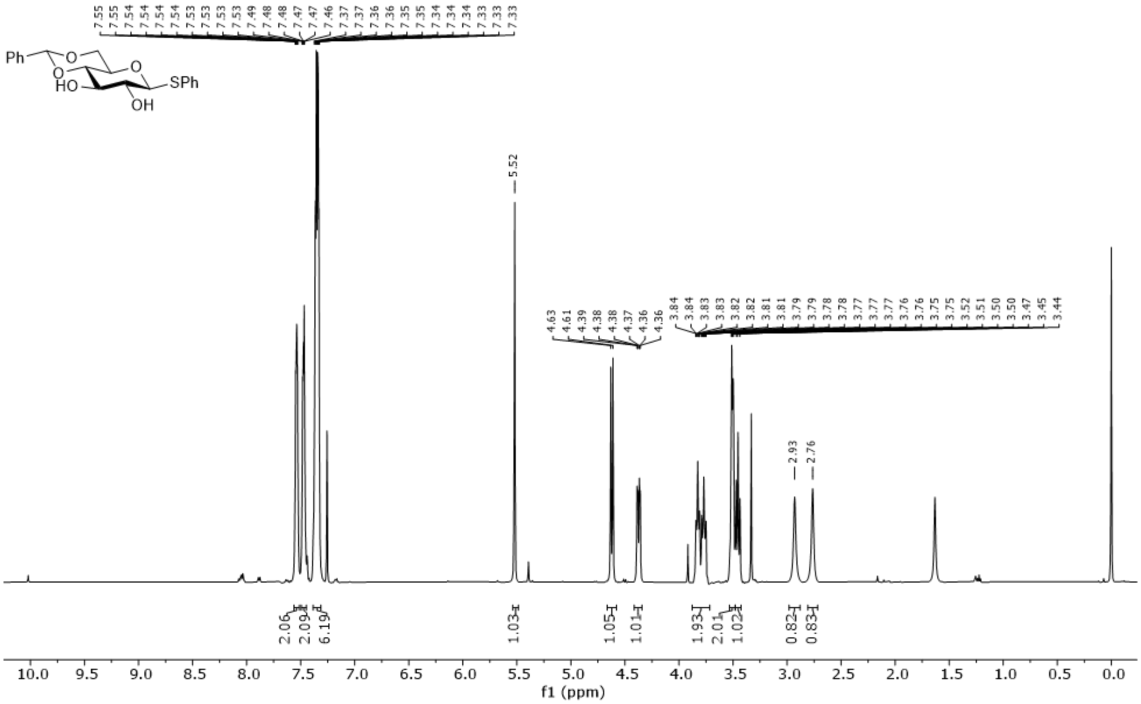

Figure S142: ^{1}H NMR spectrum.

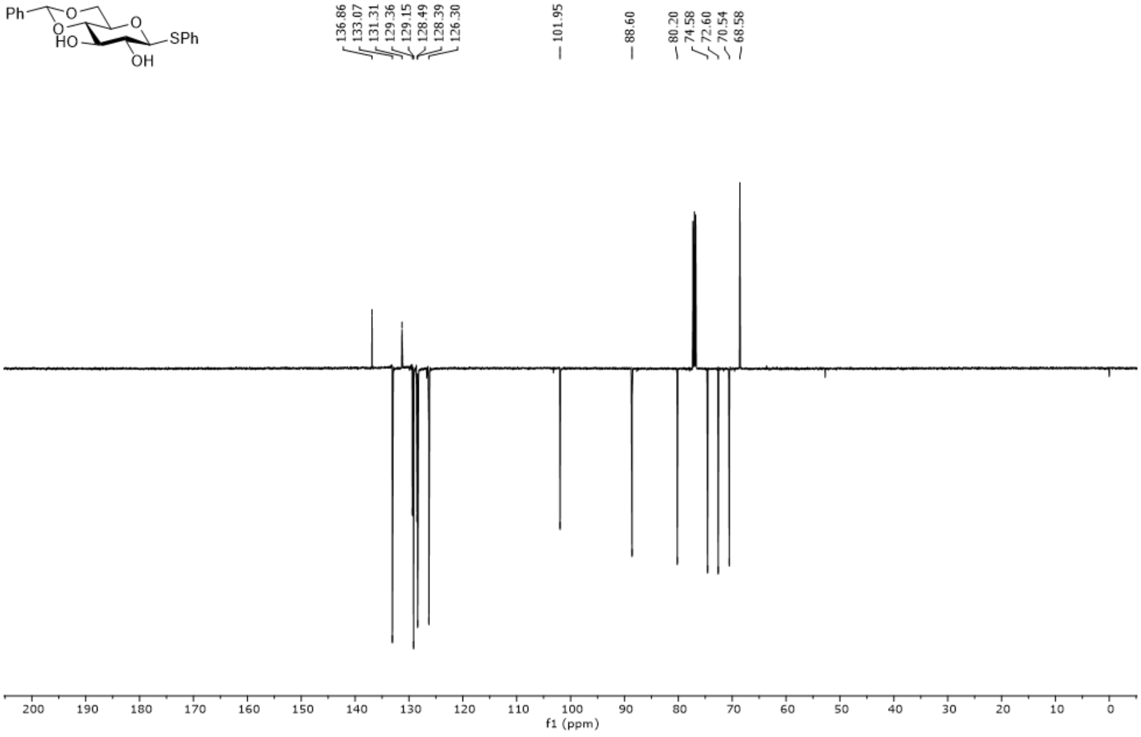

Figure S143: ^{13}C NMR spectrum.

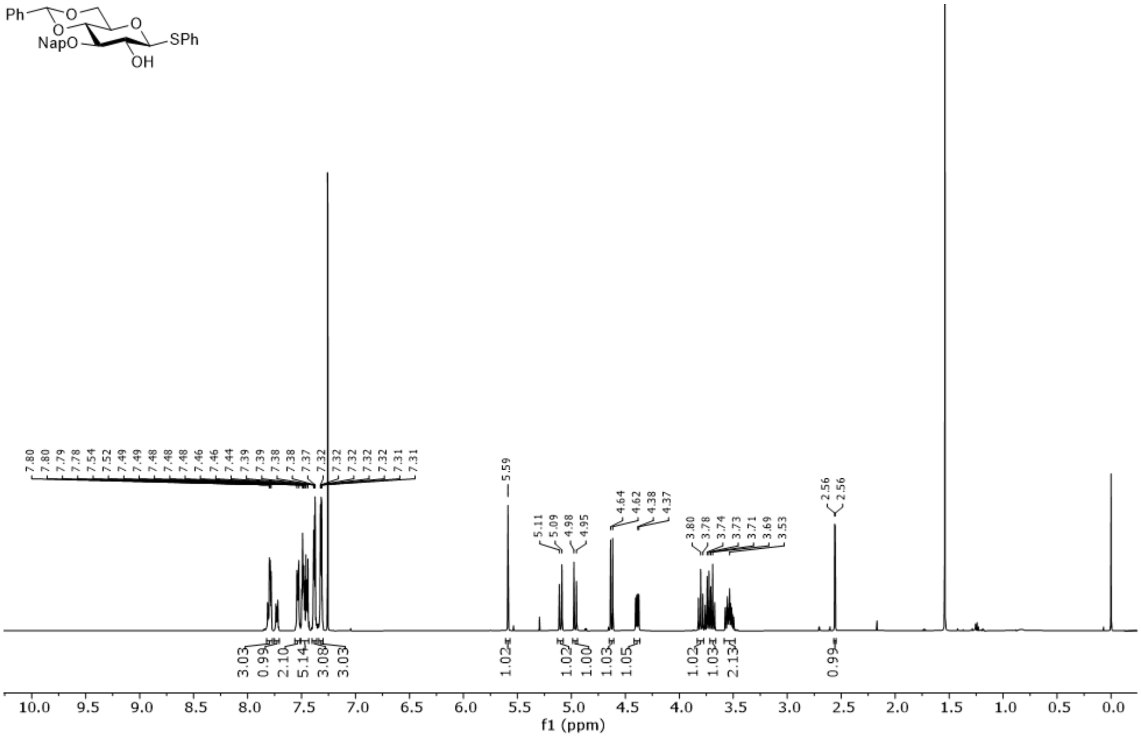

Figure S144: ^{1}H NMR spectrum.

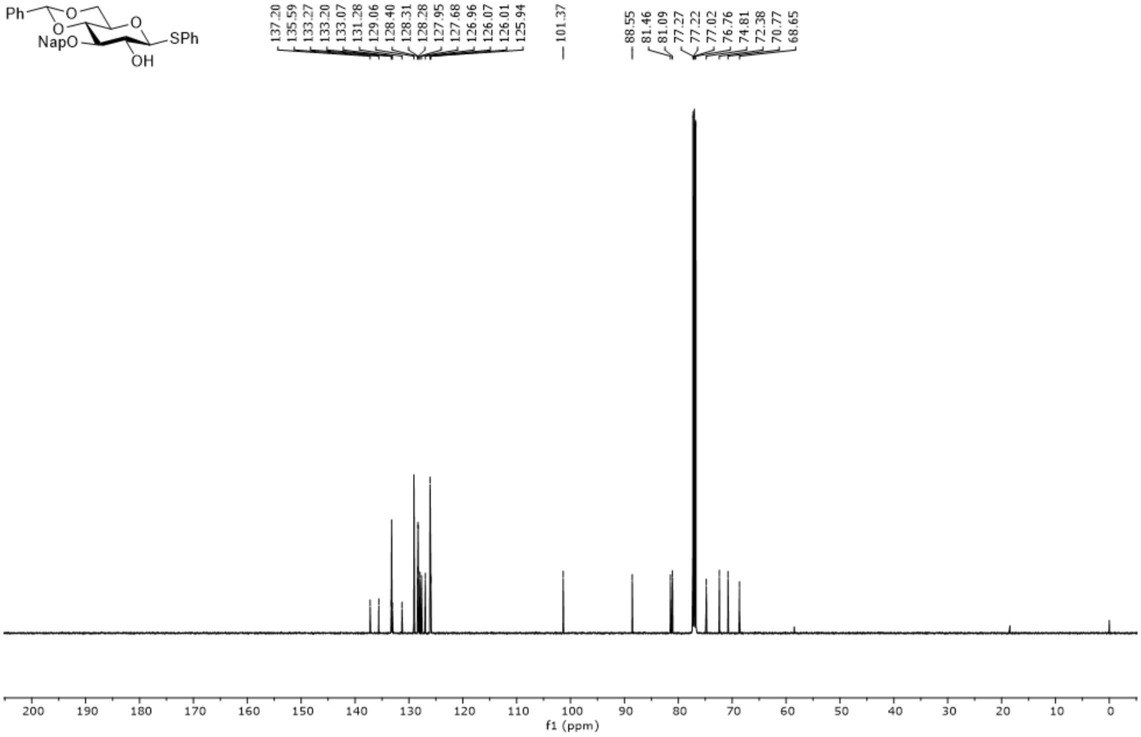

Figure S145: ^{13}C NMR spectrum.

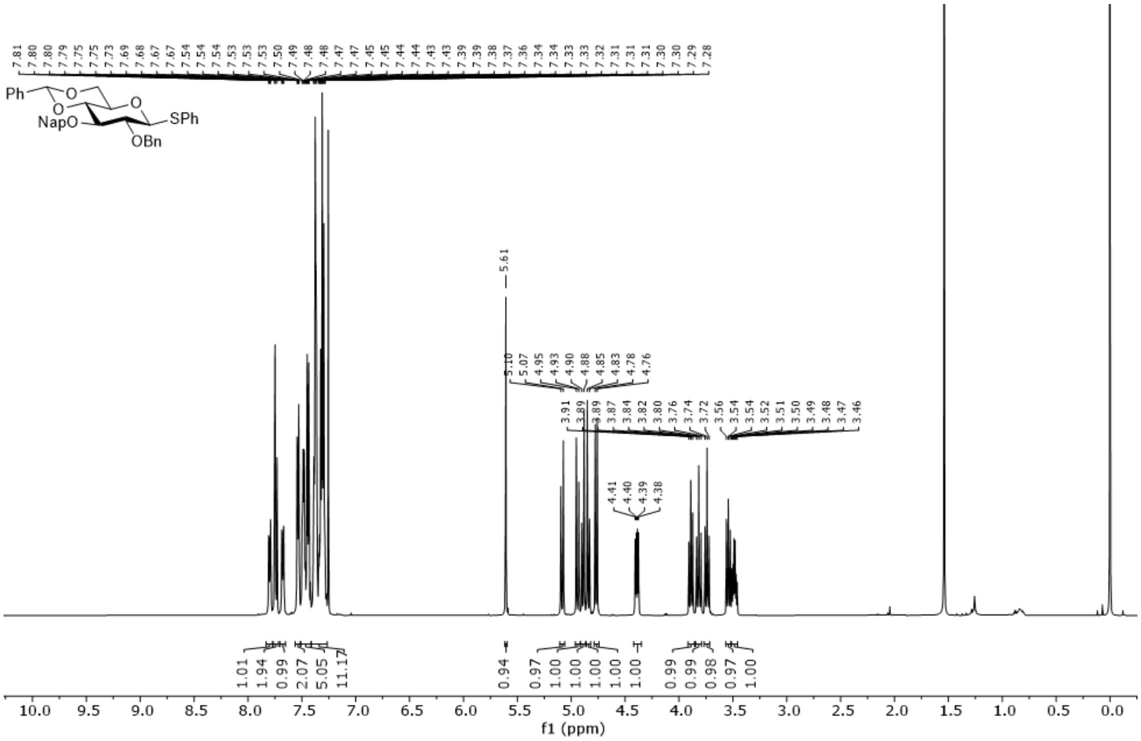

Figure S146: ^{1}H NMR spectrum.

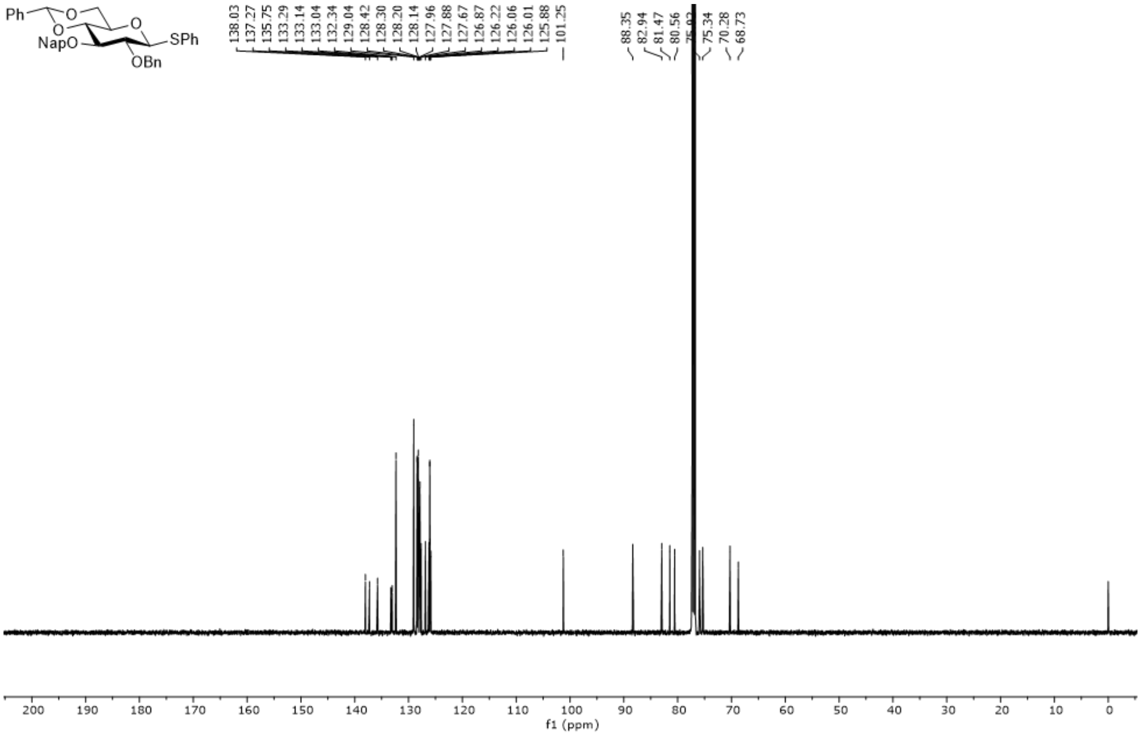

Figure S147: ^{13}C NMR spectrum.

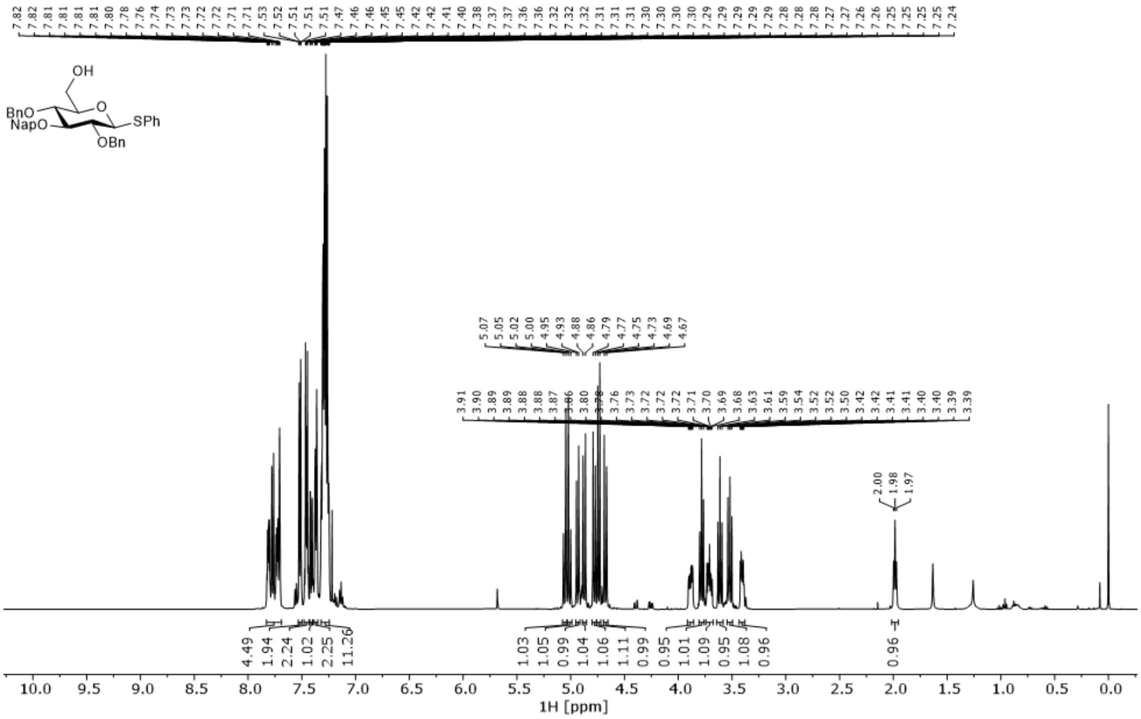

Figure S148: ^{1}H NMR spectrum.

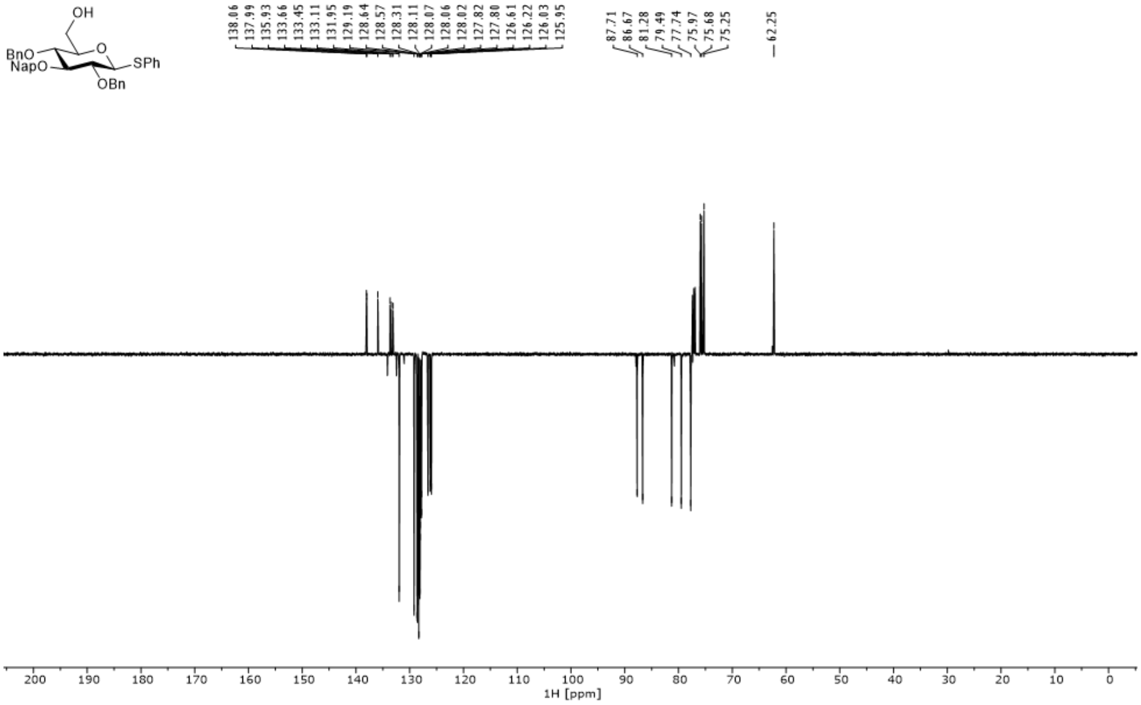

Figure S149: ^{13}C NMR spectrum.

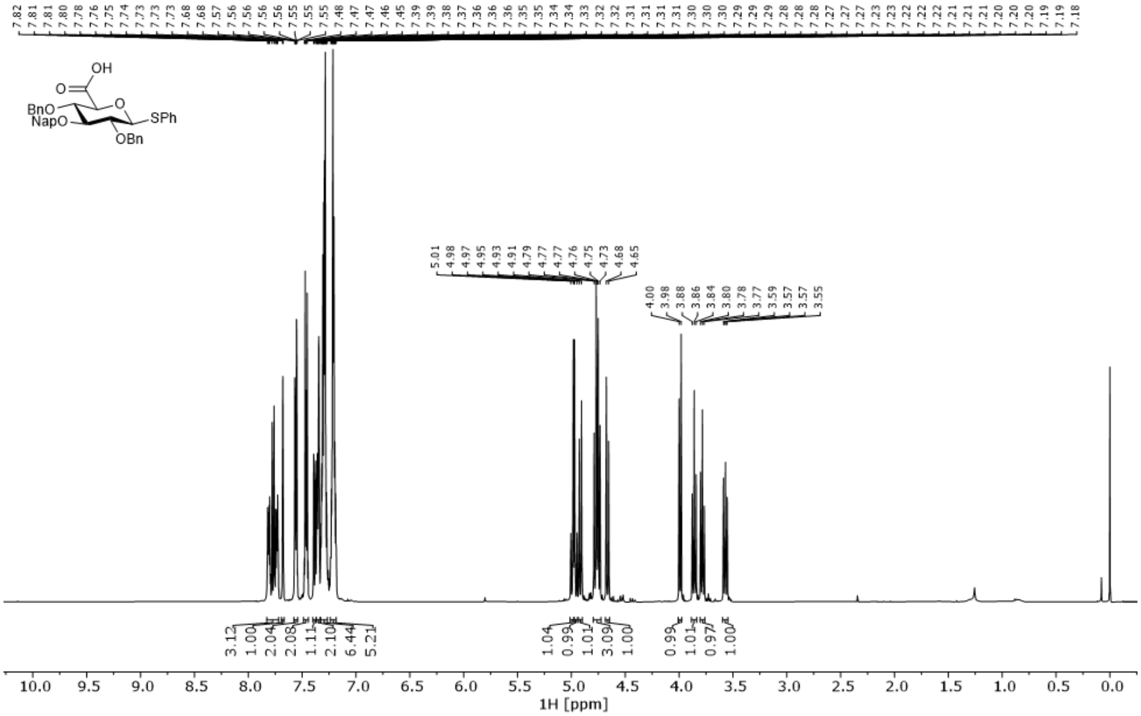

Figure S150: ^{1}H NMR spectrum.

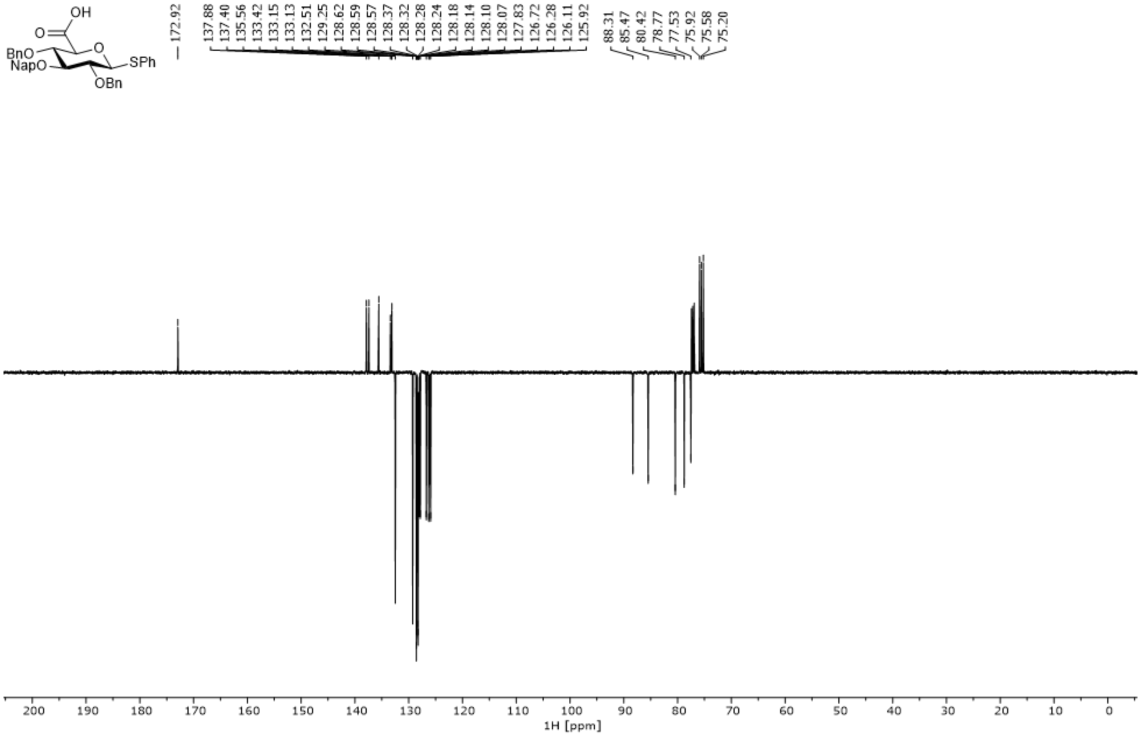

Figure S151: ^{13}C NMR spectrum.

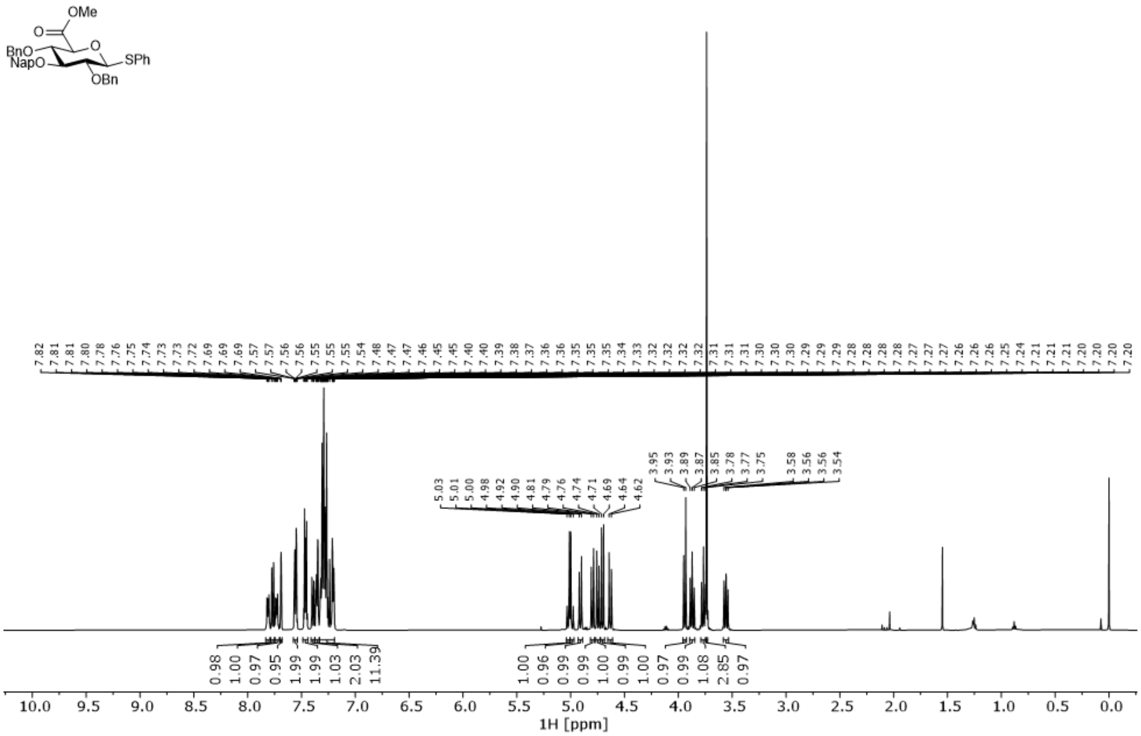

Figure S152: ^{1}H NMR spectrum.

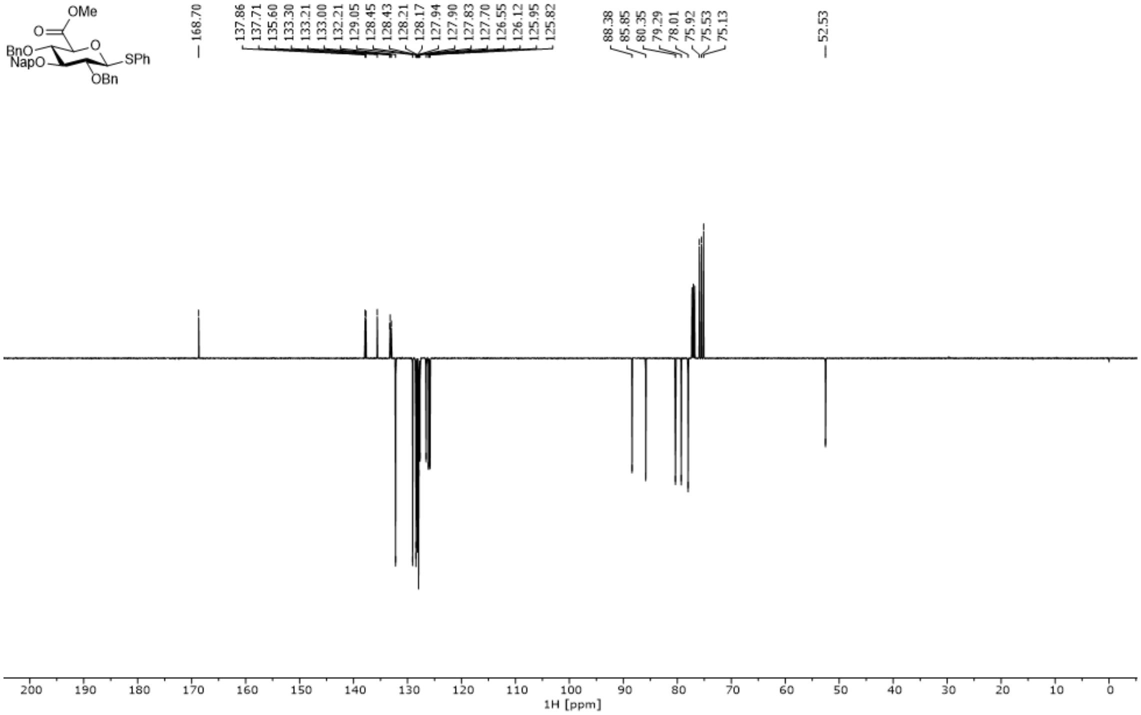

Figure S153: ^{13}C NMR spectrum.

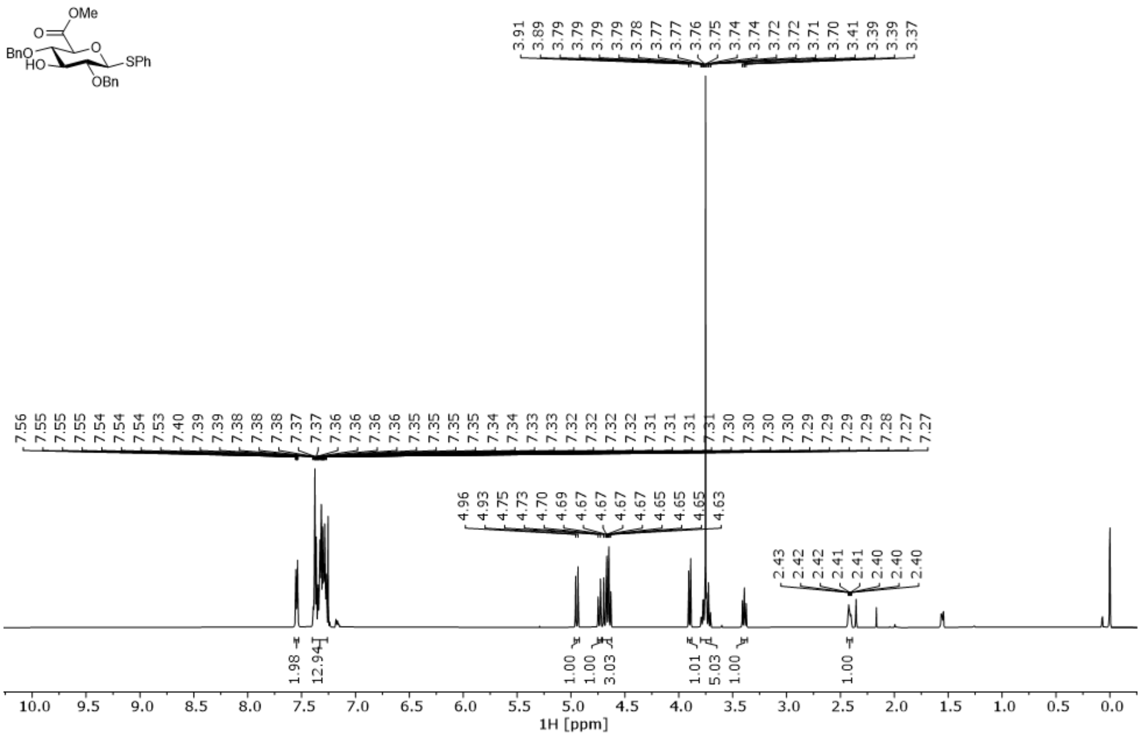

Figure S154: ^{1}H NMR spectrum.

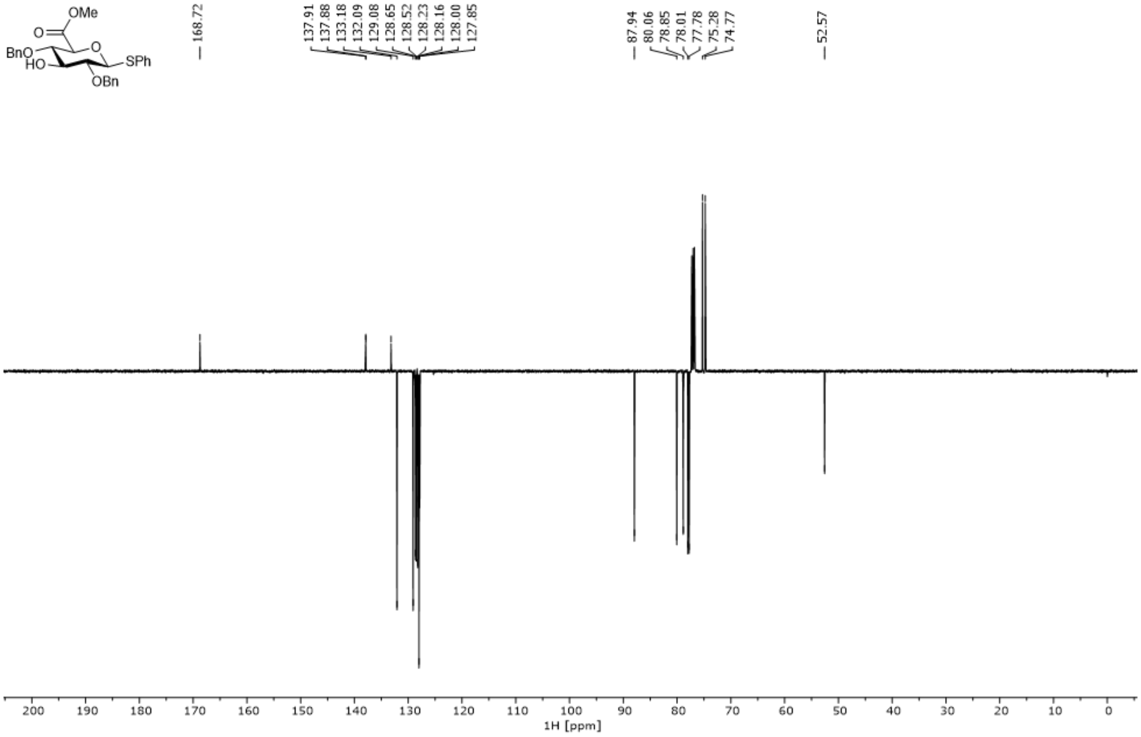

Figure S155: ^{13}C NMR spectrum.

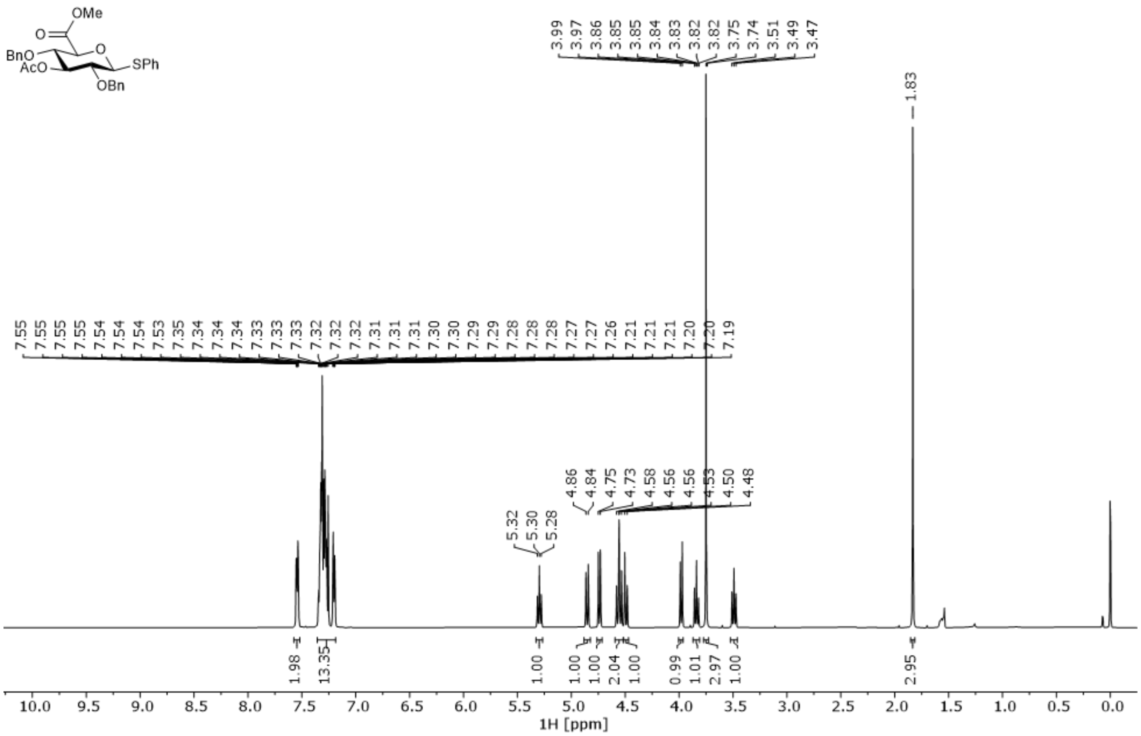

Figure S156: ^{1}H NMR spectrum.

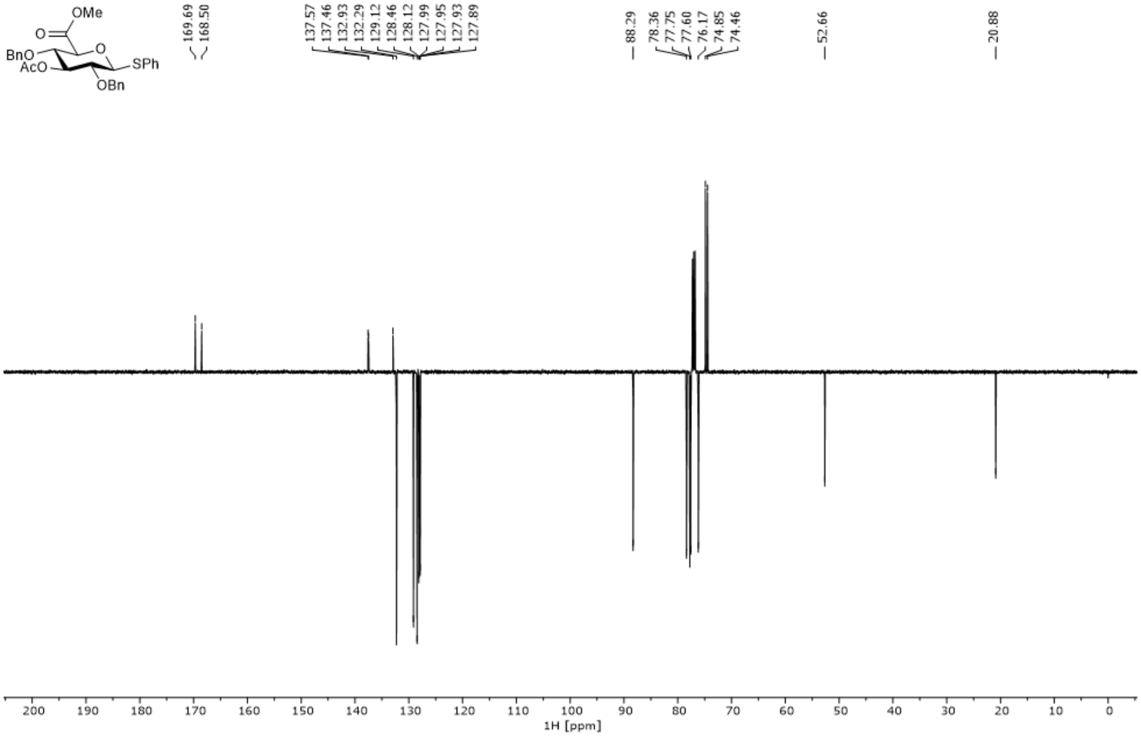

Figure S157: ^{13}C NMR spectrum.

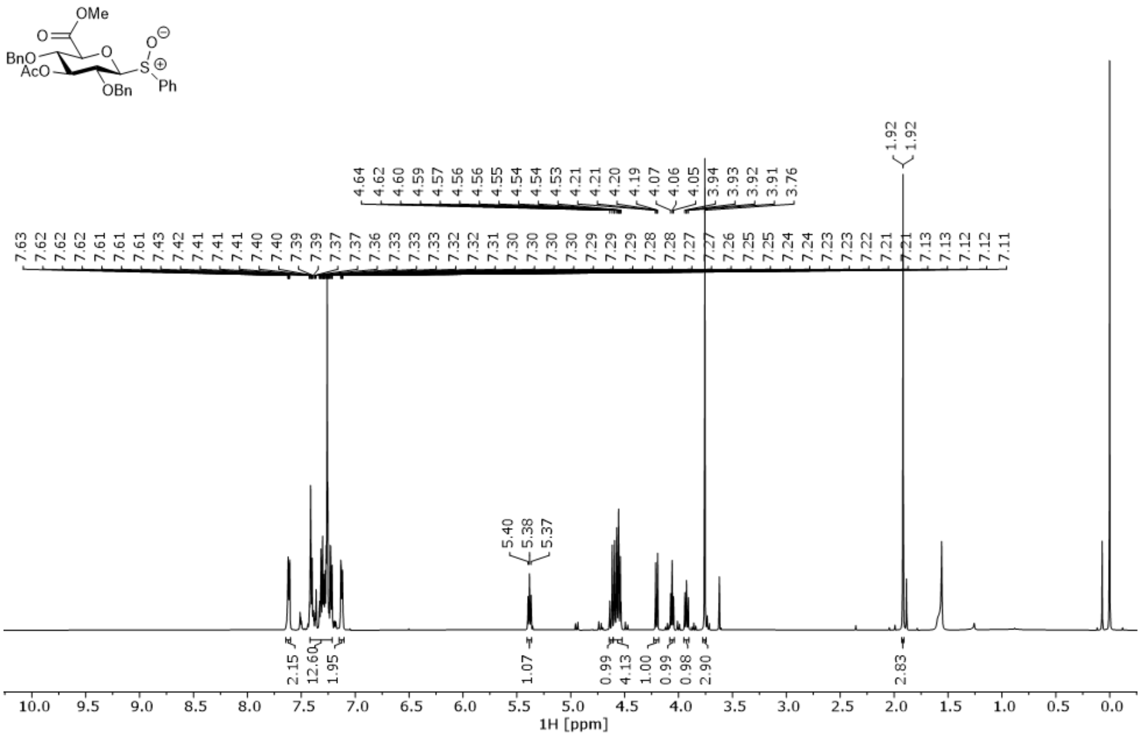

Figure S158: ^{1}H NMR spectrum.

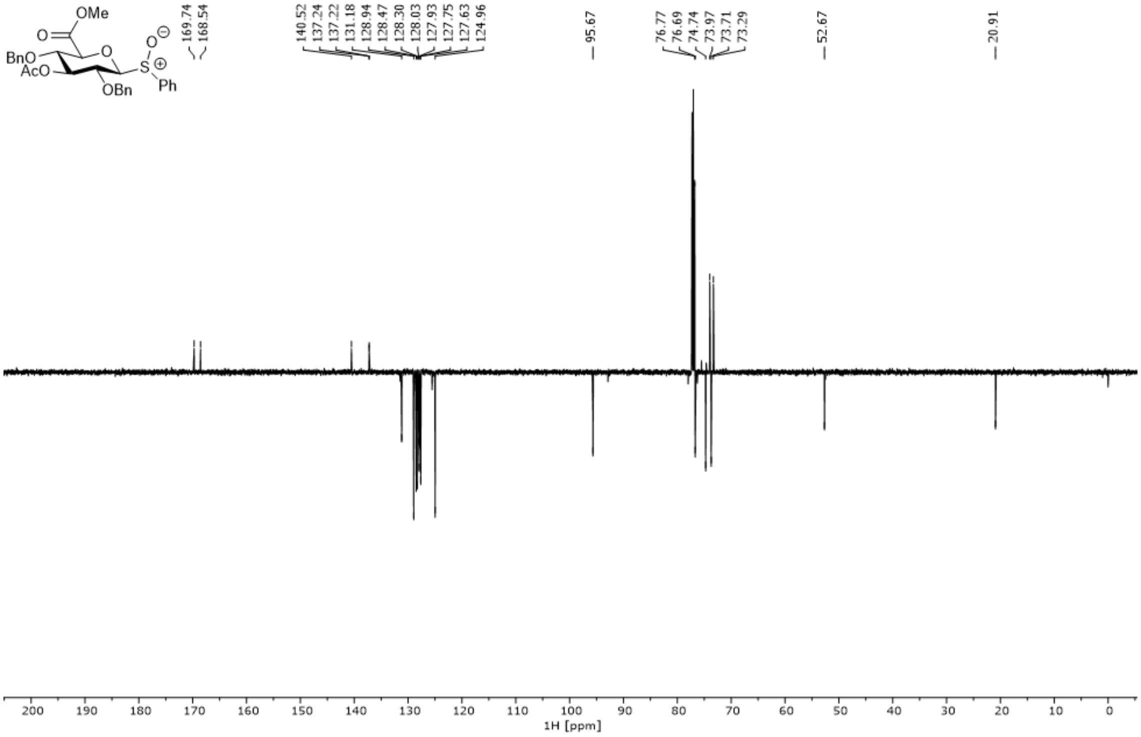

Figure S159: ^{13}C NMR spectrum.

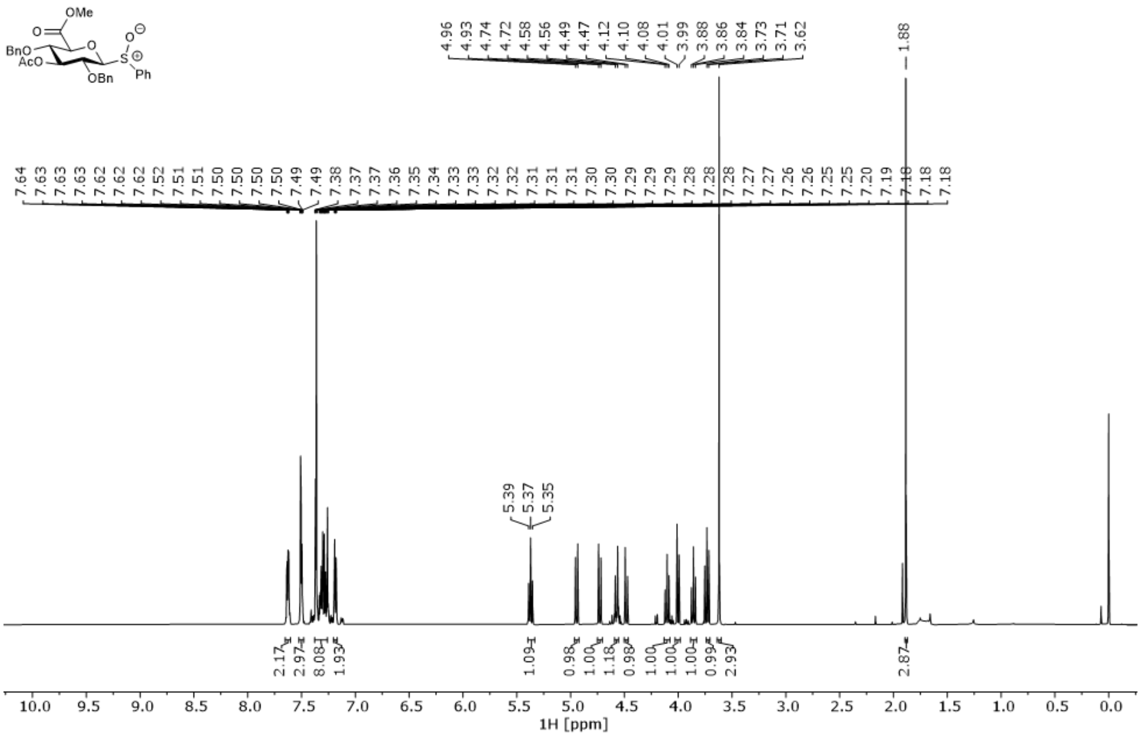

Figure S160: ^{1}H NMR spectrum.

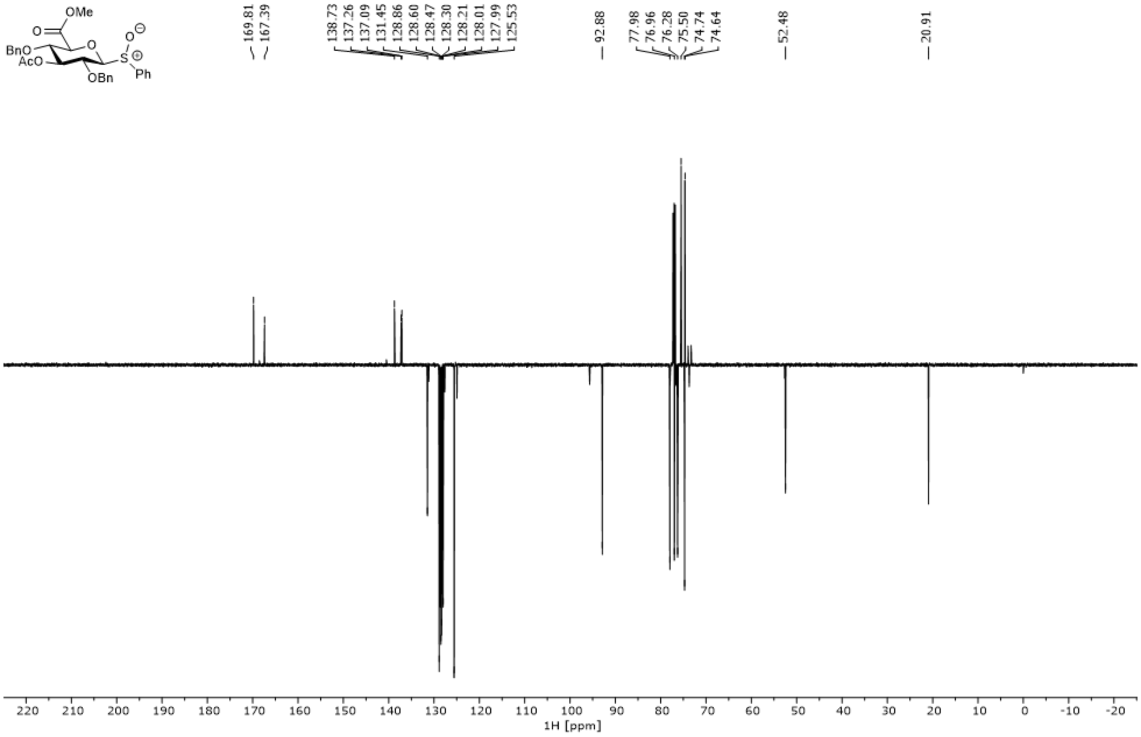

Figure S161: ^{13}C NMR spectrum.

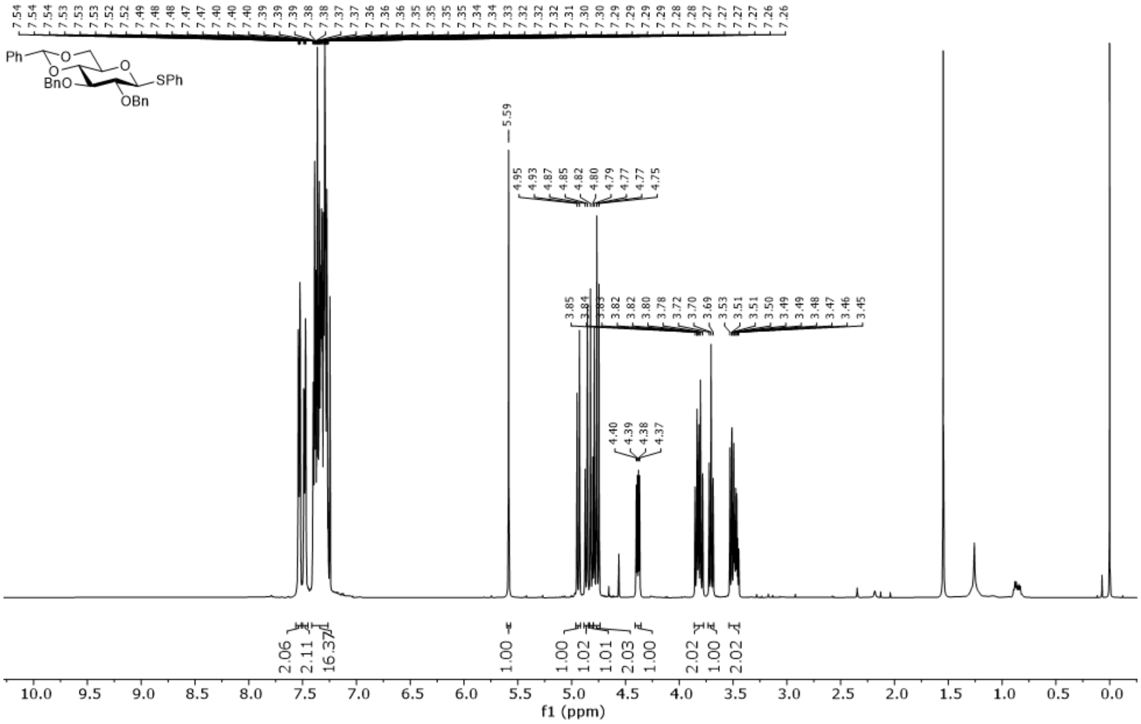

Figure S162: ^{1}H NMR spectrum.

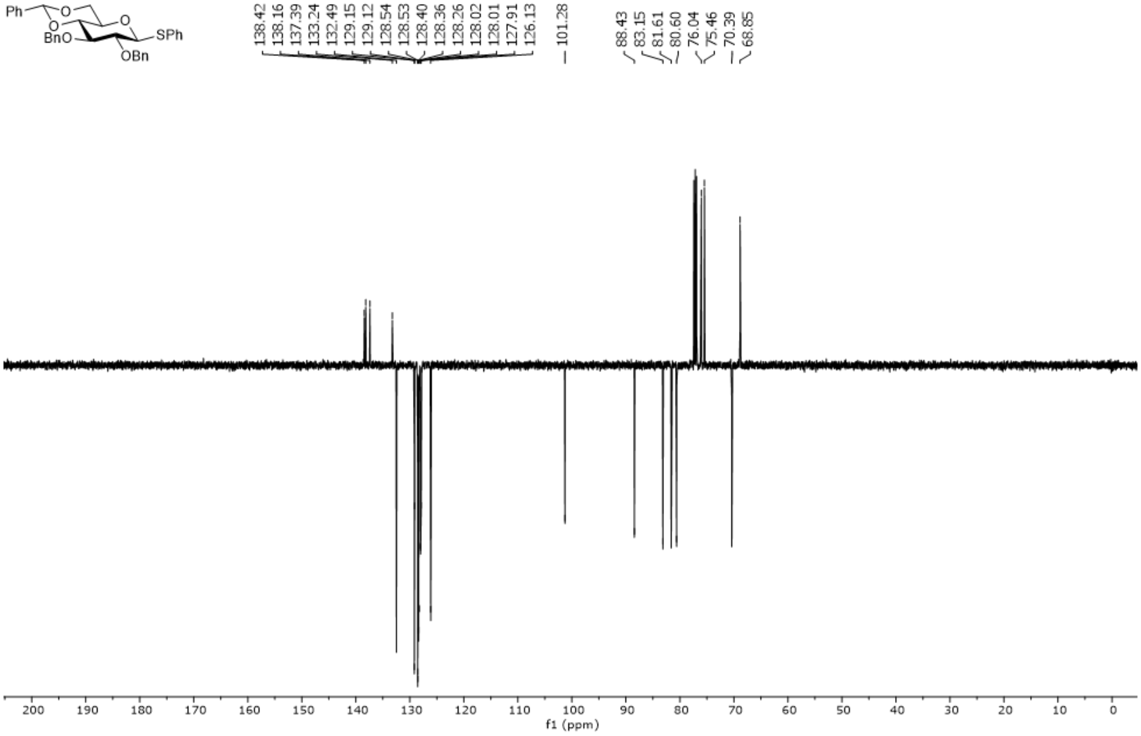

Figure S163: ^{13}C NMR spectrum.

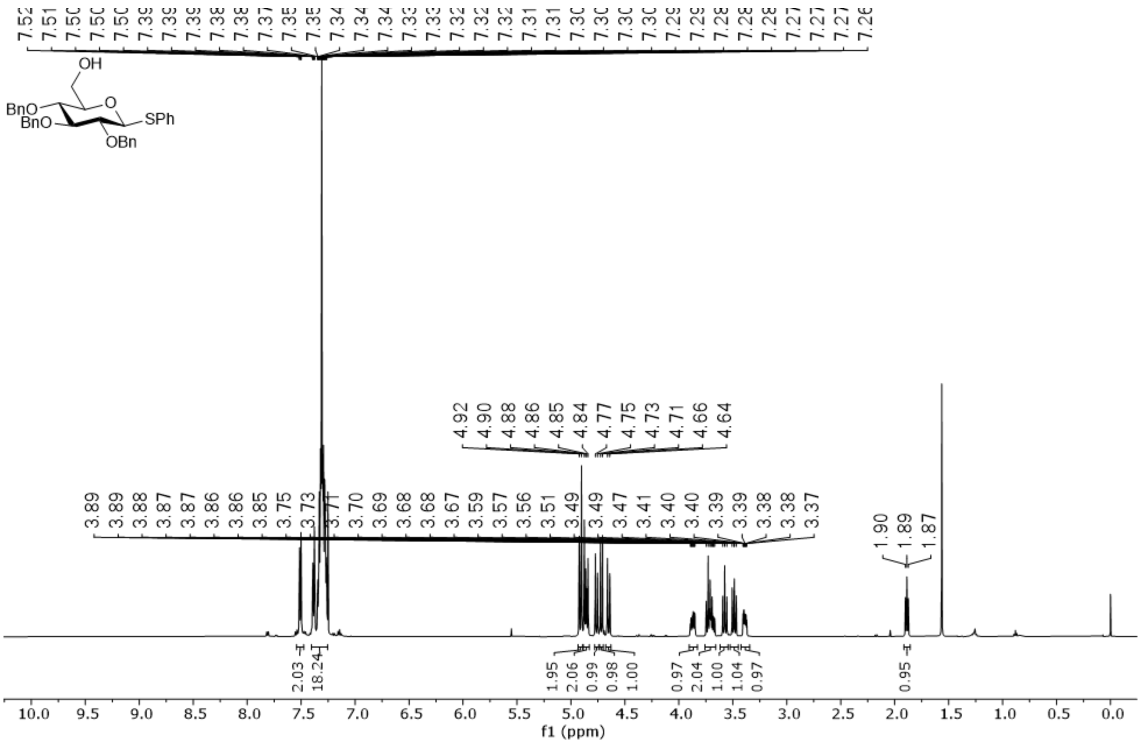

Figure S164: ^{1}H NMR spectrum.

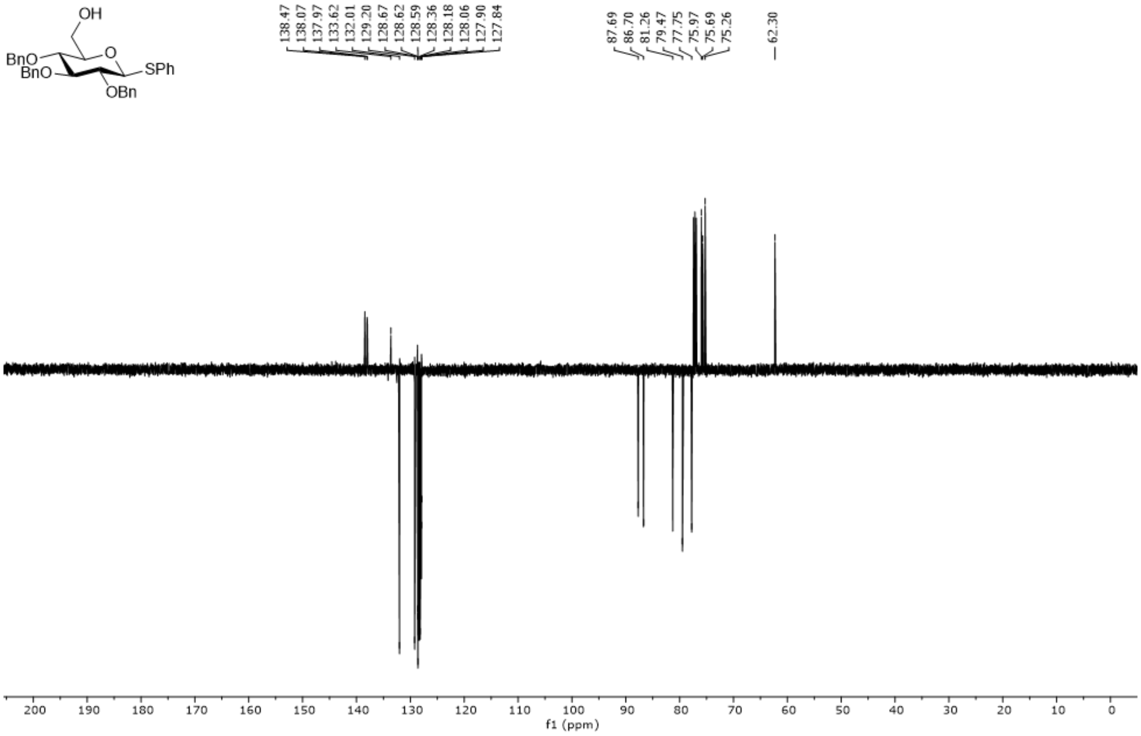

Figure S165: ^{13}C NMR spectrum.

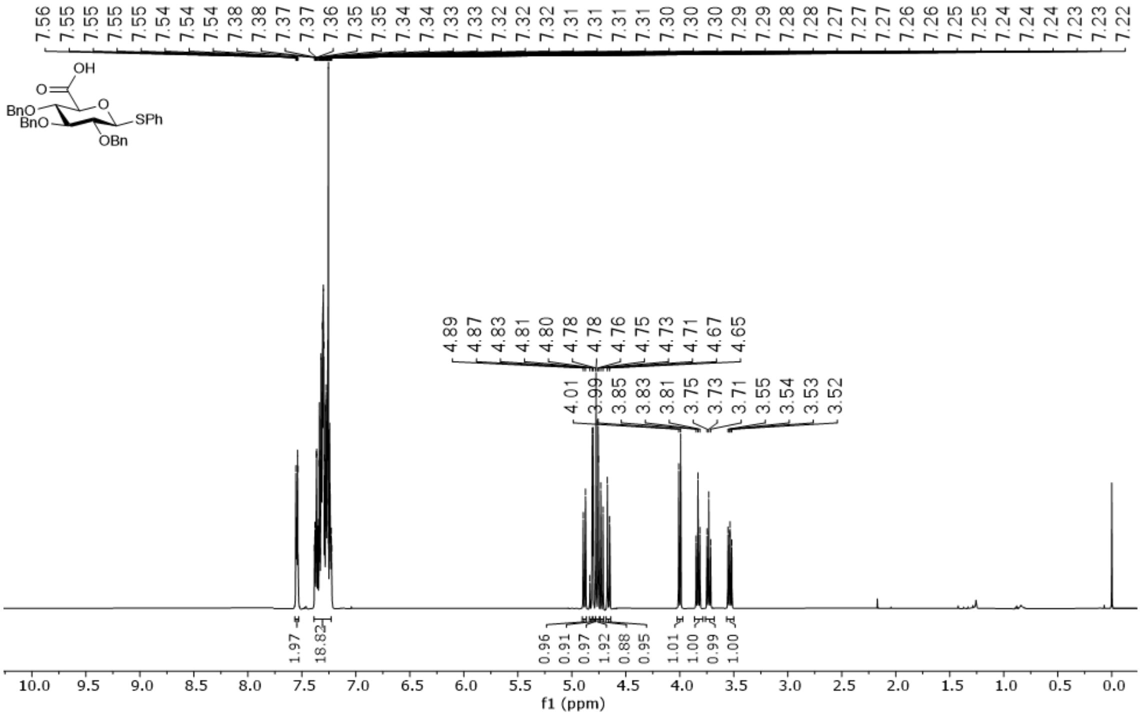

Figure S166: ^{1}H NMR spectrum.

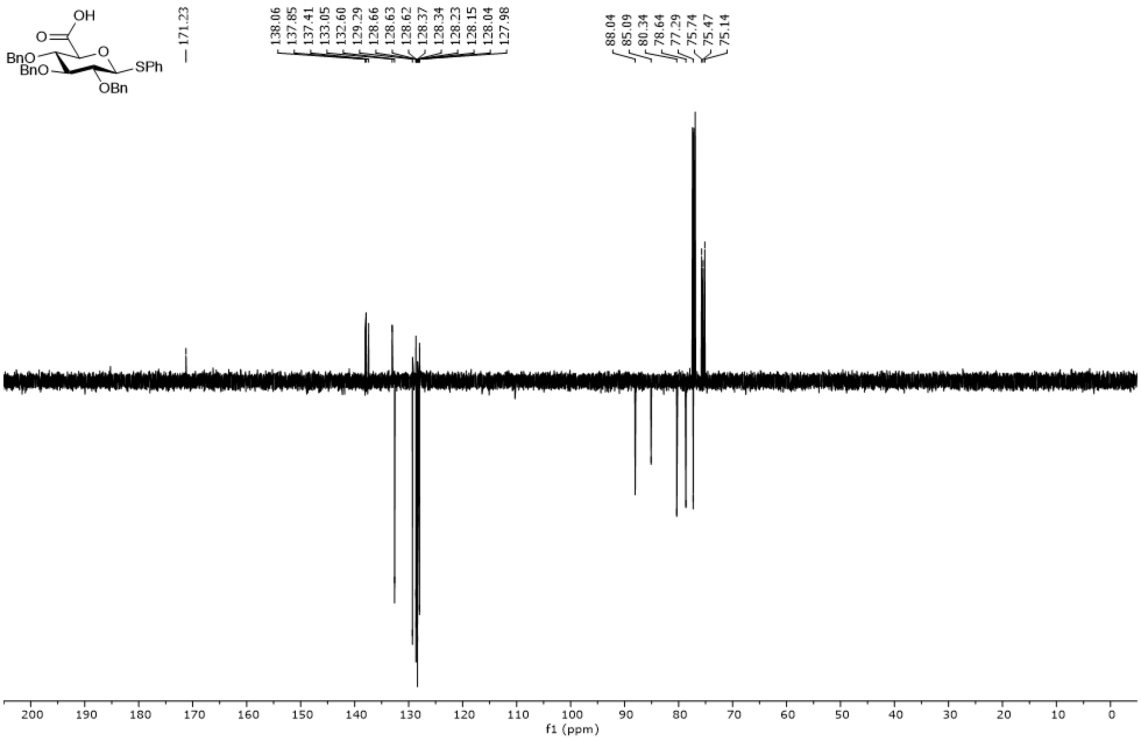

Figure S167: ^{13}C NMR spectrum.

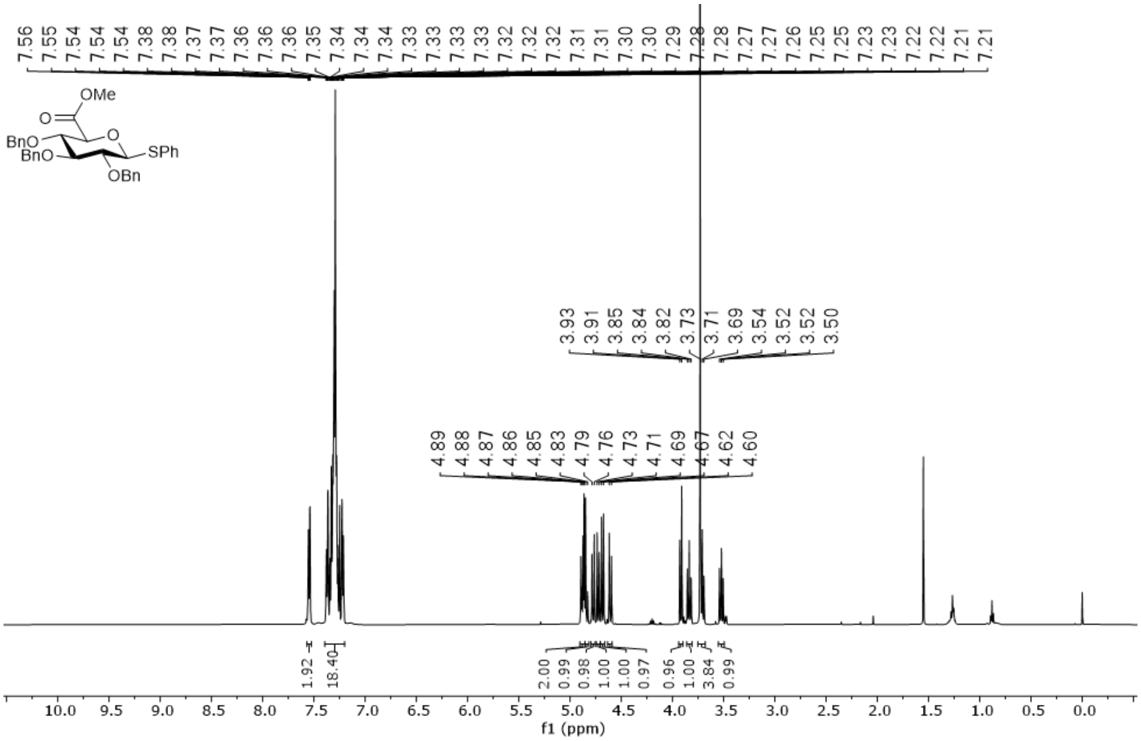

Figure S168: ^{1}H NMR spectrum.

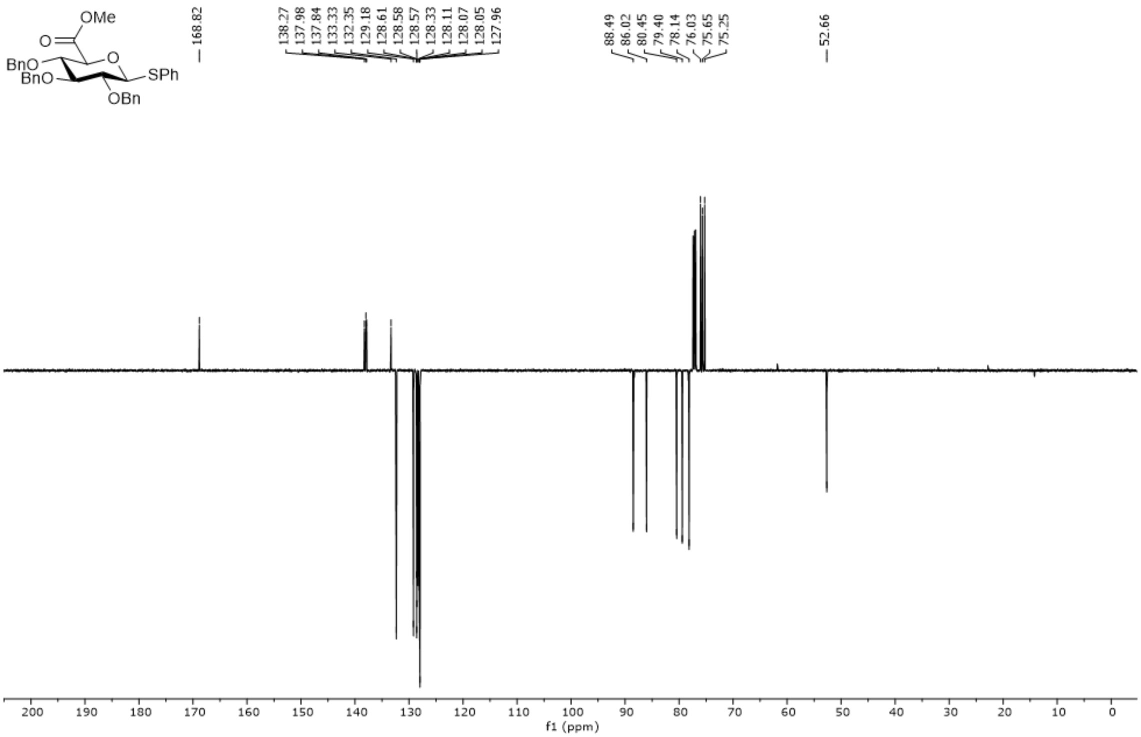

Figure S169: ^{13}C NMR spectrum.

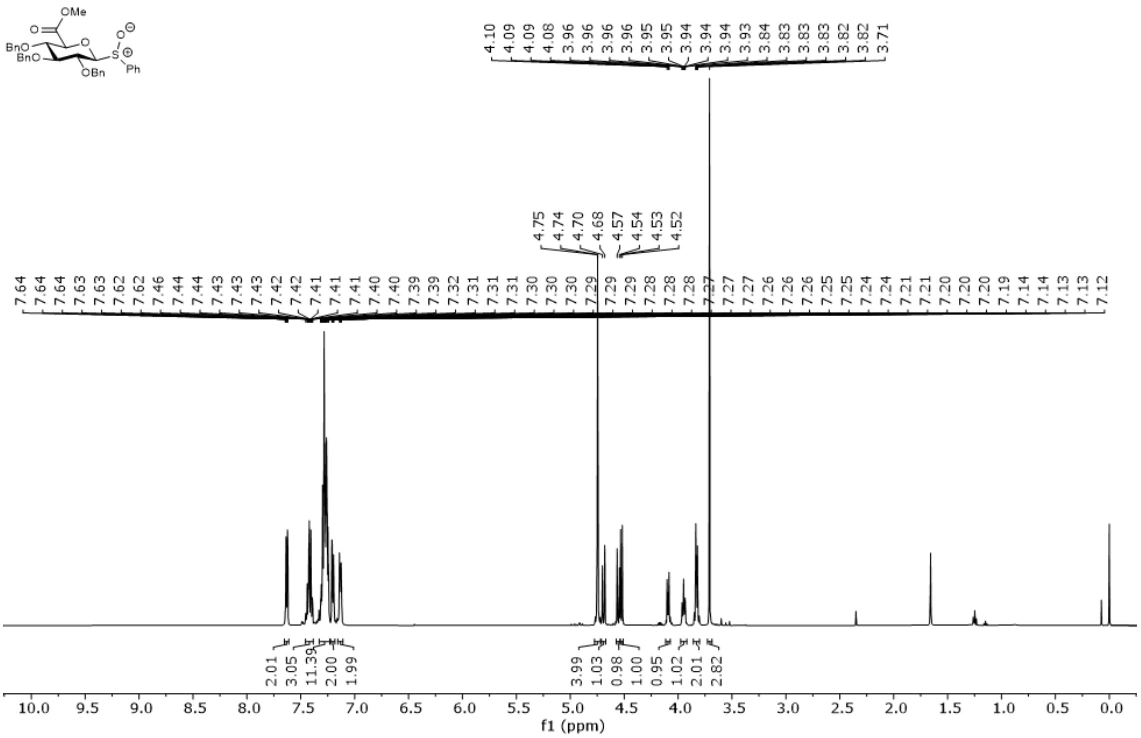

Figure S170: ^{1}H NMR spectrum.

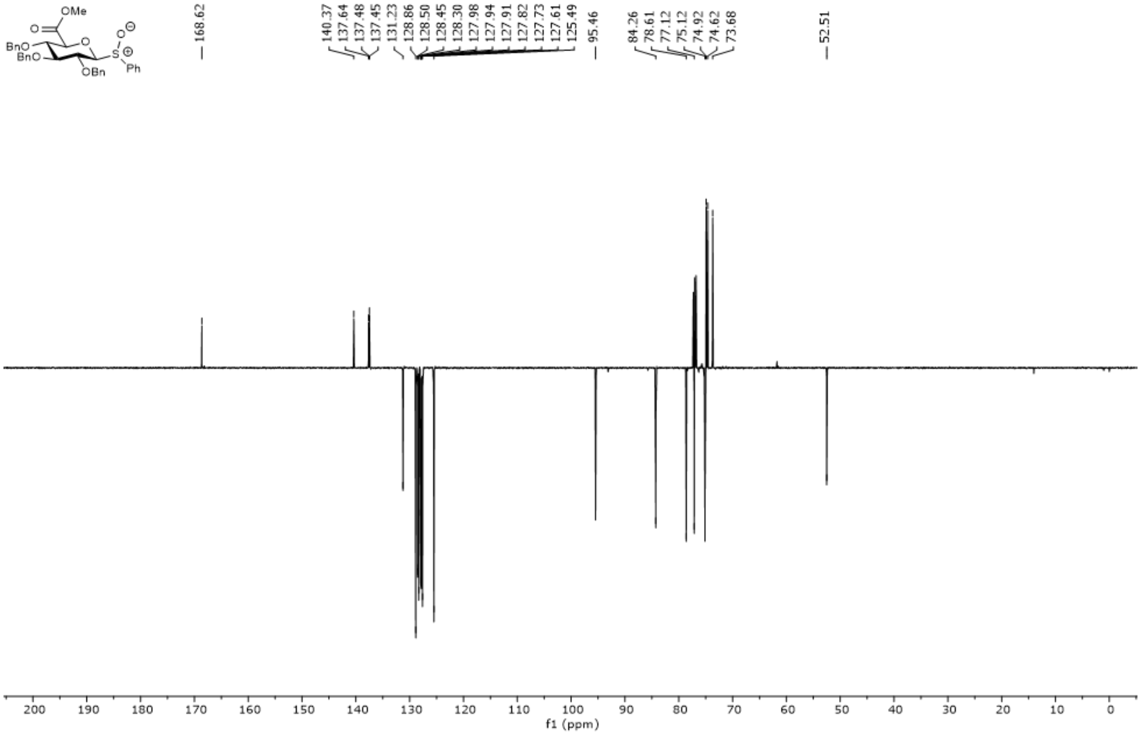

Figure S171: ^{13}C NMR spectrum.

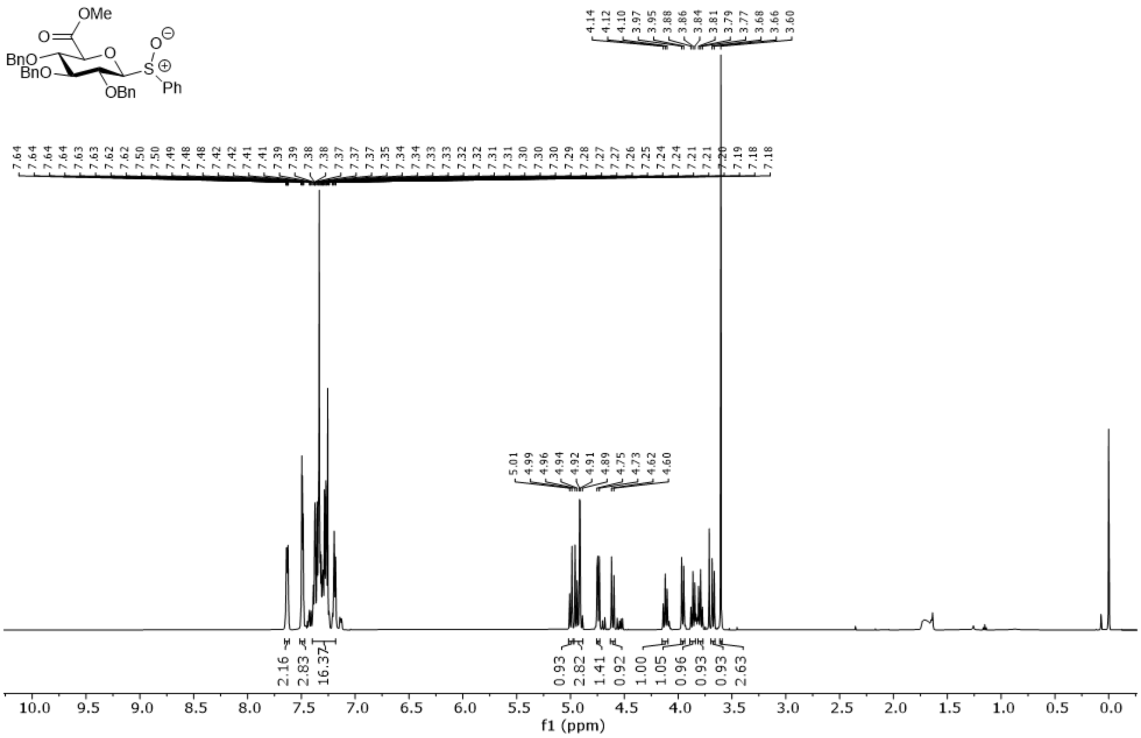

Figure S172: ^{1}H NMR spectrum.

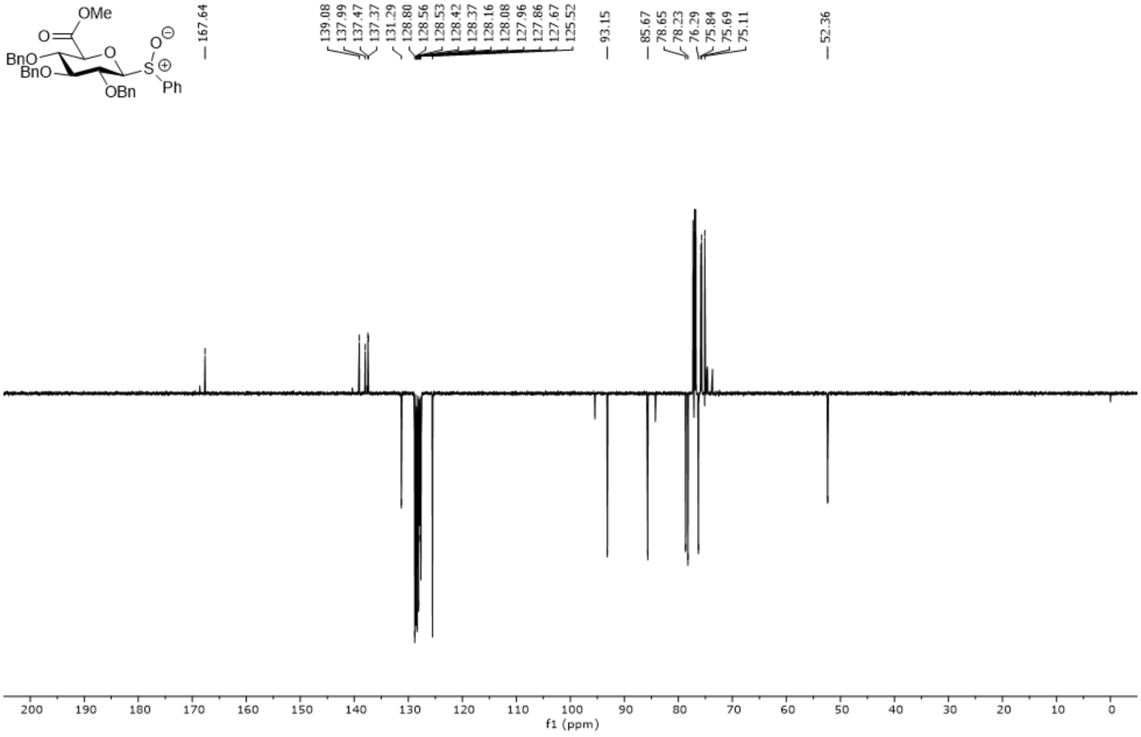

Figure S173: ^{13}C NMR spectrum.

Coordinates

*Mannuronic acid donor **1**; C-1,C-3-dioxanium ion (0.0 kJ/mol)*

O	3.159460000	-1.694538000	0.809401000
C	3.173062000	-0.941704000	-0.146400000
O	4.177182000	-0.131877000	-0.467851000
C	5.346711000	-0.157459000	0.397066000
C	2.002224000	-0.761881000	-1.122712000
O	1.576035000	-2.059959000	-1.599980000
C	0.471779000	-2.686216000	-1.089123000
C	-0.740506000	-1.795072000	-0.846510000
O	-1.761765000	-2.599131000	-0.310764000
C	-3.112812000	-2.326776000	-0.777470000
C	-3.679368000	-1.016691000	-0.285608000
C	-3.946139000	0.028239000	-1.179969000
C	-4.485925000	1.235431000	-0.723365000
C	-4.758191000	1.408028000	0.635245000
C	-4.496115000	0.369304000	1.536803000
C	-3.965297000	-0.836521000	1.077967000
C	-0.249279000	-0.729465000	0.125378000
O	0.246545000	-1.399736000	1.348821000
C	0.690506000	-2.613429000	1.326066000
C	1.075164000	-3.232976000	2.615598000
O	0.798326000	-3.313339000	0.257519000
C	0.868738000	0.112864000	-0.518113000
O	0.195061000	0.833130000	-1.528931000
C	0.749789000	2.135594000	-1.858978000
C	0.717797000	3.108898000	-0.705265000
C	1.902062000	3.679254000	-0.219791000
C	1.869950000	4.594944000	0.837215000
C	0.650826000	4.939699000	1.423418000
C	-0.536954000	4.372475000	0.947005000
C	-0.504654000	3.466423000	-0.113223000
H	6.036589000	0.561001000	-0.040294000
H	5.064666000	0.134184000	1.410149000
H	5.776581000	-1.160088000	0.402095000
H	2.374801000	-0.255776000	-2.013711000
H	0.267145000	-3.565289000	-1.695687000
H	-1.020526000	-1.308996000	-1.787539000
H	-3.682987000	-3.175960000	-0.394653000
H	-3.123373000	-2.369598000	-1.873591000
H	-3.748764000	-0.105078000	-2.241002000
H	-4.703242000	2.031627000	-1.429031000
H	-5.187078000	2.340129000	0.990494000
H	-4.723563000	0.494435000	2.591160000
H	-3.783314000	-1.648815000	1.777367000
H	-1.044391000	-0.082981000	0.489826000
H	0.616322000	-2.698949000	3.447340000
H	0.792199000	-4.287474000	2.615393000
H	2.165866000	-3.167186000	2.696297000
H	1.289864000	0.790149000	0.236187000
H	1.773743000	2.016952000	-2.236777000
H	0.121055000	2.468694000	-2.687842000
H	2.853069000	3.419551000	-0.679181000
H	2.793235000	5.038080000	1.197739000
H	0.622725000	5.652049000	2.242224000
H	-1.487308000	4.647610000	1.394412000
H	-1.430262000	3.037542000	-0.488490000

*Mannuronic acid donor **1**; C-1,C-5-dioxolanium ion (41.6 kJ/mol)*

C	-0.817419000	4.382225000	-1.306575000
C	-0.948277000	2.919809000	-1.626205000
O	-0.171970000	2.150953000	-0.779219000
C	-0.282149000	0.721238000	-0.924044000
C	1.148616000	0.159884000	-0.745180000
O	1.191462000	-1.126465000	-1.294381000
C	2.415764000	-1.500945000	-1.982614000
C	3.610772000	-1.645952000	-1.070230000
C	3.661882000	-2.689094000	-0.130471000
C	4.755641000	-2.815605000	0.726900000
C	5.816804000	-1.905066000	0.650894000
C	5.780248000	-0.870784000	-0.286784000
C	4.679869000	-0.742072000	-1.141766000
C	1.485556000	0.073160000	0.782371000
C	1.149404000	1.359576000	1.505626000
O	1.953041000	2.336727000	1.646891000
C	1.499438000	3.558119000	2.342908000
O	-0.044196000	1.346535000	1.966877000
O	0.535723000	-0.788519000	1.430157000
C	-0.615844000	-0.027492000	1.495404000
C	-1.280557000	0.162865000	0.134530000
O	-2.407798000	0.976879000	0.309112000
C	-3.584144000	0.620256000	-0.480969000
C	-4.137319000	-0.738197000	-0.130392000
C	-4.101143000	-1.787713000	-1.058747000
C	-4.612352000	-3.047762000	-0.729983000
C	-5.160876000	-3.268869000	0.534453000
C	-5.204492000	-2.226565000	1.468242000
C	-4.698716000	-0.969598000	1.136529000
O	-1.629614000	2.436449000	-2.496086000
H	0.228905000	4.660081000	-1.155108000
H	-1.369364000	4.598477000	-0.385269000
H	-1.246465000	4.966558000	-2.120064000
H	-0.634337000	0.489564000	-1.929611000
H	1.854329000	0.846323000	-1.231594000
H	2.155663000	-2.451437000	-2.453044000
H	2.615633000	-0.765329000	-2.772001000
H	2.844372000	-3.403376000	-0.075788000
H	4.789312000	-3.630645000	1.443540000
H	6.672613000	-2.011936000	1.310388000
H	6.608245000	-0.172368000	-0.361407000
H	4.664710000	0.054116000	-1.882737000
H	2.495192000	-0.291145000	0.965511000
H	1.247366000	3.298799000	3.371158000
H	0.638818000	3.959947000	1.809682000
H	2.355798000	4.225117000	2.296283000
H	-1.285261000	-0.351585000	2.289086000
H	-1.552294000	-0.859707000	-0.167603000
H	-3.337867000	0.692203000	-1.545516000
H	-4.290840000	1.416875000	-0.239268000
H	-3.688015000	-1.615904000	-2.050055000
H	-4.585749000	-3.849892000	-1.461200000

H	-5.562545000	-4.244504000	0.790477000
H	-5.645419000	-2.392160000	2.446691000
H	-4.750227000	-0.157062000	1.857270000

*Mannuronic acid donor **1**; oxocarbenium ion (56.1 kJ/mol)*

O	1.778356000	2.426852000	2.005885000
C	2.126916000	1.720867000	1.092097000
O	3.118192000	1.948566000	0.232790000
C	3.875316000	3.176482000	0.432957000
C	1.501494000	0.351060000	0.769377000
O	0.543788000	0.094812000	1.880314000
C	-0.703547000	0.207110000	1.741406000
C	-1.427077000	0.423903000	0.468336000
O	-2.552177000	1.192319000	0.771751000
C	-3.753427000	0.917208000	-0.031514000
C	-4.172265000	-0.526015000	0.028099000
C	-4.693500000	-1.067267000	1.216514000
C	-5.069149000	-2.408571000	1.277127000
C	-4.934321000	-3.227101000	0.147899000
C	-4.424570000	-2.699345000	-1.039409000
C	-4.042953000	-1.354630000	-1.097578000
C	-0.510901000	0.962289000	-0.654157000
O	-0.237728000	2.343884000	-0.442394000
C	-1.023770000	3.250737000	-1.136412000
C	-0.662643000	4.660259000	-0.772302000
O	-1.862979000	2.894617000	-1.927174000
C	0.820353000	0.191065000	-0.602131000
O	0.488997000	-1.163169000	-0.829823000
C	1.415554000	-1.942546000	-1.640373000
C	2.711995000	-2.271476000	-0.940570000
C	2.724927000	-3.178175000	0.132970000
C	3.917415000	-3.476613000	0.793123000
C	5.115001000	-2.879219000	0.381546000
C	5.114551000	-1.986494000	-0.692070000
C	3.916940000	-1.683337000	-1.348462000
H	4.624724000	3.177234000	-0.355579000
H	3.211330000	4.037726000	0.347338000
H	4.342367000	3.162247000	1.418768000
H	2.250004000	-0.430335000	0.906625000
H	-1.272479000	0.085435000	2.665574000
H	-1.706407000	-0.618778000	0.180838000
H	-3.566955000	1.247873000	-1.057466000
H	-4.493179000	1.579321000	0.421253000
H	-4.819580000	-0.428788000	2.087519000
H	-5.482358000	-2.814665000	2.195390000
H	-5.238238000	-4.268341000	0.193953000
H	-4.332417000	-3.326780000	-1.920549000
H	-3.666284000	-0.940482000	-2.029923000
H	-0.987118000	0.814384000	-1.624147000
H	-0.720517000	4.797024000	0.311158000
H	-1.340795000	5.346535000	-1.277897000
H	0.367001000	4.870749000	-1.078436000
H	1.507444000	0.588148000	-1.358667000
H	0.846425000	-2.846111000	-1.868886000
H	1.600515000	-1.401759000	-2.576559000
H	1.800047000	-3.658870000	0.442623000
H	3.918317000	-4.185221000	1.615783000
H	6.044227000	-3.120390000	0.888436000
H	6.043194000	-1.532843000	-1.024434000
H	3.925098000	-0.997498000	-2.192355000

*Mannuronic acid donor **1**; C-3 ring-opened (S) ion (22.6 kJ/mol)*

O	0.051325000	3.540215000	-0.525365000
C	0.985069000	3.078279000	-1.139261000
C	1.604502000	1.685645000	-0.917801000
O	2.960403000	1.940578000	-0.611285000
C	3.946430000	1.176098000	-1.352253000
C	3.938162000	-0.298141000	-1.020498000
C	4.363580000	-0.740424000	0.243673000
C	4.353282000	-2.099785000	0.559109000
C	3.922509000	-3.037422000	-0.387913000
C	3.503916000	-2.609421000	-1.649560000
C	3.510935000	-1.245091000	-1.961941000
C	0.938822000	0.830135000	0.173337000
O	0.775823000	1.721530000	1.356745000
C	-0.350093000	1.938434000	1.918456000
C	-0.460164000	3.048649000	2.889915000
O	-1.403612000	1.222058000	1.700285000
C	-0.371714000	0.137171000	-0.241184000
O	-1.024171000	0.897324000	-1.225709000
C	-1.826223000	0.167518000	-2.193716000
C	-3.160559000	-0.307877000	-1.668146000
C	-3.450047000	-1.676176000	-1.580371000
C	-4.690761000	-2.111148000	-1.102063000
C	-5.650158000	-1.178162000	-0.703646000
C	-5.370795000	0.191049000	-0.791339000
C	-4.135946000	0.623849000	-1.275544000
C	-1.249902000	-0.055258000	1.013517000
C	-0.669527000	-1.046761000	2.033455000
O	-0.873043000	-2.293092000	1.622790000
C	-0.371581000	-3.355206000	2.486165000
O	-0.096136000	-0.700135000	3.043145000
H	1.524019000	3.663239000	-1.908702000
H	1.501021000	1.133207000	-1.863495000
H	4.893231000	1.642135000	-1.071050000
H	3.790513000	1.333059000	-2.427147000
H	4.714988000	-0.016729000	0.974855000
H	4.700874000	-2.430918000	1.533221000
H	3.933757000	-4.096548000	-0.148590000
H	3.189169000	-3.333733000	-2.394916000
H	3.209143000	-0.919664000	-2.955053000
H	1.659855000	0.092391000	0.525402000
H	-1.469766000	3.462601000	2.860296000
H	0.287010000	3.812346000	2.677319000
H	-0.287673000	2.635662000	3.891835000
H	-0.107308000	-0.858616000	-0.625113000
H	-1.960257000	0.892576000	-3.000102000
H	-1.238434000	-0.676264000	-2.579425000
H	-2.710003000	-2.407003000	-1.898402000
H	-4.907273000	-3.173635000	-1.047579000

H	-6.615331000	-1.513147000	-0.336444000
H	-6.120802000	0.918545000	-0.496157000
H	-3.926167000	1.687511000	-1.354170000
H	-2.260924000	-0.339422000	0.728881000
H	-0.845260000	-3.282946000	3.466072000
H	0.711441000	-3.268921000	2.585331000
H	-0.648947000	-4.279296000	1.983943000

*Mannuronic acid donor **1**; C-3 ring-opened (R) ion (56.1 kJ/mol)*

O	3.327257000	1.515138000	0.218009000
C	2.301692000	1.899226000	-0.307783000
C	1.236353000	1.000589000	-0.943108000
O	0.647562000	1.745280000	-1.984418000
C	-0.809429000	1.812657000	-1.985557000
C	-1.365018000	2.551609000	-0.792775000
C	-2.157515000	1.888471000	0.154458000
C	-2.673446000	2.580087000	1.256536000
C	-2.394897000	3.937929000	1.422945000
C	-1.604538000	4.608061000	0.481072000
C	-1.096514000	3.919922000	-0.620819000
C	1.717049000	-0.375644000	-1.495529000
O	3.191892000	-0.437077000	-1.577392000
C	3.872901000	-0.758535000	-0.529742000
C	5.336942000	-0.527090000	-0.542119000
O	3.386957000	-1.413633000	0.469078000
C	1.228196000	-1.651475000	-0.792986000
O	-0.146997000	-1.501320000	-0.588605000
C	-0.924843000	-2.745891000	-0.558873000
C	-2.378725000	-2.394682000	-0.441273000
C	-3.153260000	-2.176454000	-1.589793000
C	-4.503158000	-1.837424000	-1.478530000
C	-5.090327000	-1.717764000	-0.215248000
C	-4.326416000	-1.939927000	0.934574000
C	-2.975890000	-2.277482000	0.822351000
C	2.004507000	-1.926592000	0.529325000
C	1.300329000	-1.416478000	1.794125000
O	1.622239000	-0.166904000	2.107741000
C	1.011783000	0.373884000	3.316308000
O	0.528843000	-2.135274000	2.386332000
H	2.034390000	2.969753000	-0.347634000
H	0.512621000	0.812575000	-0.135875000
H	-1.221363000	0.798550000	-2.040199000
H	-1.029946000	2.332758000	-2.920208000
H	-2.387818000	0.834180000	0.022965000
H	-3.299187000	2.059511000	1.975465000
H	-2.800046000	4.477230000	2.273845000
H	-1.399070000	5.667708000	0.599425000
H	-0.498337000	4.447164000	-1.359872000
H	1.414002000	-0.407812000	-2.540934000
H	5.580941000	0.294957000	-1.213328000
H	5.829940000	-1.442909000	-0.891460000
H	5.679453000	-0.316823000	0.472172000
H	1.436642000	-2.490218000	-1.473303000
H	-0.596093000	-3.342655000	0.297608000
H	-0.718875000	-3.290923000	-1.488665000
H	-2.703060000	-2.284219000	-2.573785000
H	-5.097577000	-1.678292000	-2.372946000
H	-6.142221000	-1.463398000	-0.127865000
H	-4.784763000	-1.862096000	1.915813000
H	-2.383329000	-2.458725000	1.715253000
H	2.144106000	-2.997733000	0.678239000
H	1.482718000	1.344141000	3.459075000
H	-0.064220000	0.475416000	3.167246000
H	1.214412000	-0.289802000	4.157630000

*Glucuronic acid donor **5**; C-1,C-3-dioxanium ion (21.6 kJ/mol)*

O	2.371880000	2.206255000	-0.702711000
C	2.425680000	1.700211000	0.406609000
O	3.523178000	1.552836000	1.131228000
C	4.766814000	2.037799000	0.553983000
C	1.185001000	1.150403000	1.121623000
O	0.392826000	2.318322000	1.484066000
C	-0.739490000	2.647094000	0.820651000
C	-1.660514000	1.489177000	0.461982000
O	-2.065338000	0.874938000	1.651022000
C	-3.477908000	0.534965000	1.743656000
C	-3.919712000	-0.504001000	0.742791000
C	-4.739099000	-0.153483000	-0.339694000
C	-5.142663000	-1.116996000	-1.270455000
C	-4.729813000	-2.442768000	-1.123334000
C	-3.918406000	-2.804638000	-0.041025000
C	-3.517465000	-1.842253000	0.886334000
C	-0.814577000	0.563350000	-0.415667000
O	-0.432206000	1.331221000	-1.636168000
C	-0.206608000	2.603059000	-1.585953000
C	0.246049000	3.270008000	-2.830284000
O	-0.383979000	3.316199000	-0.535224000
C	0.486467000	0.063465000	0.256191000
O	0.206109000	-1.044318000	1.079099000
C	0.337056000	-2.341557000	0.442477000
C	1.751990000	-2.665688000	0.024472000
C	2.070150000	-2.886282000	-1.322334000
C	3.378417000	-3.203357000	-1.703213000
C	4.381344000	-3.296695000	-0.736622000
C	4.073623000	-3.076888000	0.611426000
C	2.767485000	-2.765691000	0.989550000
H	5.528002000	1.814526000	1.298268000
H	4.694788000	3.111979000	0.376630000
H	4.966733000	1.514649000	-0.382460000
H	1.457551000	0.695210000	2.072209000
H	-1.216928000	3.470148000	1.347831000
H	-2.511389000	1.872311000	-0.117437000
H	-4.070483000	1.452878000	1.639118000
H	-3.577054000	0.174408000	2.769092000
H	-5.087444000	0.871901000	-0.443752000
H	-5.787315000	-0.834760000	-2.097249000
H	-5.050440000	-3.195053000	-1.837498000
H	-3.615630000	-3.839638000	0.087453000
H	-2.897941000	-2.125984000	1.732872000
H	-1.386732000	-0.276257000	-0.805821000
H	-0.084738000	2.711223000	-3.706208000
H	1.342137000	3.278384000	-2.803866000
H	-0.109551000	4.300998000	-2.854706000
H	1.174344000	-0.222185000	-0.549763000

H	-0.019434000	-3.031312000	1.211000000
H	-0.345084000	-2.412840000	-0.415215000
H	1.289521000	-2.828386000	-2.077833000
H	3.609740000	-3.381352000	-2.749012000
H	5.396264000	-3.549332000	-1.028163000
H	4.849091000	-3.162391000	1.366734000
H	2.529294000	-2.608149000	2.038310000

Glucuronic acid donor ***5****; C-1,C-5-dioxolanium ion (40.8 kJ/mol)*

O	1.017823000	0.201789000	2.752768000
C	1.154247000	1.257235000	2.183537000
O	0.938241000	1.389748000	0.824244000
C	0.713081000	0.152251000	0.098989000
C	-0.790408000	-0.216047000	0.206010000
O	-0.955346000	-1.584529000	-0.015781000
C	-1.895526000	-2.266043000	0.857561000
C	-3.331492000	-1.846578000	0.644472000
C	-3.990550000	-2.161079000	-0.556122000
C	-5.310270000	-1.758415000	-0.765680000
C	-5.991848000	-1.041595000	0.225706000
C	-5.349019000	-0.732590000	1.426209000
C	-4.023537000	-1.132148000	1.632095000
C	-1.595583000	0.568585000	-0.884833000
C	-1.285369000	2.047642000	-0.853270000
O	-1.896345000	2.896298000	-0.124921000
C	-1.487994000	4.314967000	-0.157841000
O	-0.324978000	2.350900000	-1.644241000
O	-1.073440000	0.265820000	-2.188015000
C	0.108688000	0.963073000	-2.250965000
C	1.216176000	0.406237000	-1.347824000
O	1.594617000	-0.790839000	-1.986112000
C	3.016124000	-0.957871000	-2.240245000
C	3.846151000	-1.077685000	-0.985368000
C	3.694733000	-2.189312000	-0.139960000
C	4.458843000	-2.303405000	1.021236000
C	5.391560000	-1.311910000	1.348597000
C	5.556195000	-0.207739000	0.510354000
C	4.783292000	-0.091192000	-0.650200000
C	1.587501000	2.556153000	2.803017000
H	1.298744000	-0.650309000	0.546423000
H	-1.148097000	0.085268000	1.197868000
H	-1.747392000	-3.319184000	0.609811000
H	-1.590523000	-2.105645000	1.898842000
H	-3.467382000	-2.726851000	-1.322913000
H	-5.813161000	-2.015503000	-1.693140000
H	-7.022585000	-0.739824000	0.066407000
H	-5.878386000	-0.192073000	2.205124000
H	-3.533224000	-0.905363000	2.576124000
H	-2.658601000	0.333720000	-0.853508000
H	-0.435994000	4.381647000	0.117576000
H	-1.662847000	4.702868000	-1.161430000
H	-2.132022000	4.795096000	0.573768000
H	0.406780000	1.159541000	-3.278322000
H	2.039033000	1.129632000	-1.296901000
H	3.054310000	-1.872389000	-2.836082000
H	3.372358000	-0.125747000	-2.861827000
H	2.979518000	-2.966177000	-0.397937000
H	4.337217000	-3.168600000	1.665809000
H	5.993130000	-1.407886000	2.247315000
H	6.289648000	0.555931000	0.751339000
H	4.928445000	0.761804000	-1.309578000
H	1.030089000	3.402288000	2.394526000
H	1.458455000	2.501182000	3.883750000
H	2.647936000	2.717864000	2.580564000

Glucuronic acid donor ***5****; oxocarbenium ion (39.6 kJ/mol)*

O	-0.111152000	1.387615000	2.128009000
C	0.990385000	1.216269000	1.651830000
O	2.065762000	1.967934000	1.825336000
C	1.915277000	3.144641000	2.671368000
C	1.318434000	0.038363000	0.719967000
O	0.324232000	-1.002518000	1.050024000
C	-0.841530000	-0.921589000	0.570872000
C	-1.293661000	-0.016419000	-0.532663000
O	-1.827966000	-0.943602000	-1.453364000
C	-3.271900000	-0.849107000	-1.703969000
C	-4.100005000	-1.193441000	-0.493414000
C	-4.156033000	-2.517667000	-0.024541000
C	-4.899147000	-2.833856000	1.113437000
C	-5.601848000	-1.831070000	1.793037000
C	-5.562638000	-0.515265000	1.328067000
C	-4.813322000	-0.198108000	0.189856000
C	-0.164538000	0.835200000	-1.184638000
O	-0.224259000	2.214435000	-0.790805000
C	-1.277049000	2.943868000	-1.291737000
C	-1.188861000	4.388127000	-0.894137000
O	-2.146221000	2.424946000	-1.955972000
C	1.238252000	0.338657000	-0.793448000
O	1.469302000	-0.850278000	-1.508634000
C	2.800756000	-1.030369000	-2.069046000
C	3.874203000	-1.258642000	-1.032387000
C	4.847522000	-0.282076000	-0.780358000
C	5.834008000	-0.493215000	0.189022000
C	5.852312000	-1.684522000	0.917078000
C	4.887994000	-2.668958000	0.668825000
C	3.908147000	-2.458623000	-0.302095000
H	1.181874000	3.821244000	2.230692000
H	2.903175000	3.598859000	2.697662000
H	1.595386000	2.839350000	3.668512000
H	2.272581000	-0.418459000	0.974804000
H	-1.535115000	-1.681455000	0.932976000
H	-2.058254000	0.649065000	-0.109138000
H	-3.482427000	0.162469000	-2.064194000
H	-3.417849000	-1.563487000	-2.516085000
H	-3.630663000	-3.302275000	-0.564209000
H	-4.945838000	-3.861310000	1.461571000
H	-6.187840000	-2.080055000	2.672352000
H	-6.118745000	0.261899000	1.843185000
H	-4.801825000	0.823974000	-0.181425000
H	-0.289225000	0.743446000	-2.264648000

H	-1.995350000	4.942046000	-1.373157000
H	-1.277522000	4.477712000	0.193498000
H	-0.221019000	4.805777000	-1.185562000
H	1.970404000	1.123044000	-1.018808000
H	3.039119000	-0.160951000	-2.694222000
H	2.679864000	-1.900157000	-2.717922000
H	4.848815000	0.640640000	-1.356199000
H	6.588904000	0.266499000	0.367003000
H	6.620844000	-1.853575000	1.665020000
H	4.911491000	-3.603898000	1.220496000
H	3.171063000	-3.231856000	-0.504520000

*Glucuronic acid donor **5**; C-3 ring-opened (S) ion (0.0 kJ/mol)*

O	-0.677587000	-2.568219000	-3.033261000
C	-0.196828000	-1.461575000	-2.941620000
C	-0.825454000	-0.339857000	-2.093120000
O	-0.042843000	0.823111000	-2.216107000
C	-0.754607000	2.057763000	-2.493218000
C	-1.534205000	2.583104000	-1.310910000
C	-2.934385000	2.515810000	-1.283357000
C	-3.650073000	2.994840000	-0.180851000
C	-2.969171000	3.550695000	0.904417000
C	-1.571407000	3.629754000	0.883687000
C	-0.859113000	3.149437000	-0.216640000
C	-0.949555000	-0.693735000	-0.592765000
O	-1.813599000	-1.902854000	-0.540305000
C	-1.558909000	-2.921341000	0.187700000
C	-2.348522000	-4.152099000	-0.041469000
O	-0.633502000	-2.947126000	1.089124000
C	0.374123000	-0.930415000	0.134452000
O	1.220784000	-1.666842000	-0.712643000
C	2.632650000	-1.740557000	-0.353868000
C	3.292578000	-0.401293000	-0.136920000
C	3.416618000	0.513793000	-1.194674000
C	4.053799000	1.738782000	-0.996837000
C	4.580495000	2.062974000	0.258836000
C	4.465376000	1.158453000	1.316159000
C	3.821339000	-0.066721000	1.117551000
C	0.077868000	-1.714526000	1.429388000
C	-0.793724000	-0.952339000	2.435983000
O	-0.036208000	-0.180099000	3.203201000
C	-0.728547000	0.647943000	4.183820000
O	-2.000998000	-1.052333000	2.465365000
H	0.700633000	-1.147586000	-3.507645000
H	-1.845710000	-0.190669000	-2.475698000
H	-1.411956000	1.905051000	-3.358194000
H	0.038559000	2.750713000	-2.782060000
H	-3.472297000	2.107789000	-2.136037000
H	-4.734638000	2.946006000	-0.177140000
H	-3.524263000	3.938077000	1.753424000
H	-1.040240000	4.088074000	1.713001000
H	0.224998000	3.230197000	-0.238384000
H	-1.533810000	0.076768000	-0.087534000
H	-2.137752000	-4.505353000	-1.057609000
H	-3.413774000	-3.907163000	0.009275000
H	-2.094701000	-4.914668000	0.693086000
H	0.805115000	0.043647000	0.396113000
H	2.749675000	-2.376264000	0.532846000
H	3.066301000	-2.274165000	-1.202358000
H	3.014836000	0.265377000	-2.172594000
H	4.154402000	2.434994000	-1.824012000
H	5.086974000	3.011808000	0.407132000
H	4.881606000	1.399791000	2.289372000
H	3.749956000	-0.773998000	1.940964000
H	0.996217000	-2.056669000	1.902958000
H	0.061365000	1.185416000	4.703559000
H	-1.288175000	0.010088000	4.869187000
H	-1.403776000	1.334309000	3.670902000

*Glucuronic acid donor **5**; C-3 ring-opened (R) ion (15.9 kJ/mol)*

O	-1.231427000	1.660357000	-2.472414000
C	-0.190240000	1.983930000	-1.943671000
C	0.743585000	1.002159000	-1.220098000
O	1.971164000	1.050762000	-1.926782000
C	3.067771000	1.762468000	-1.289269000
C	3.776657000	0.942297000	-0.239783000
C	4.647595000	-0.089383000	-0.626078000
C	5.314607000	-0.851684000	0.334016000
C	5.118406000	-0.592257000	1.696454000
C	4.257940000	0.434498000	2.091407000
C	3.594141000	1.198886000	1.125846000
C	0.188394000	-0.418963000	-1.306421000
O	1.240401000	-1.297794000	-0.720905000
C	0.947758000	-2.444795000	-0.232950000
C	2.024432000	-3.234373000	0.410312000
O	-0.229043000	-2.967723000	-0.269498000
C	-1.112464000	-0.698577000	-0.551197000
O	-0.906229000	-0.381559000	0.807089000
C	-2.022355000	0.222926000	1.531297000
C	-2.237521000	1.671728000	1.177296000
C	-1.326903000	2.647195000	1.616907000
C	-1.513630000	3.990215000	1.287207000
C	-2.618867000	4.375821000	0.518596000
C	-3.534843000	3.415580000	0.085962000
C	-3.343390000	2.070042000	0.414748000
C	-1.399577000	-2.202214000	-0.722561000
C	-2.577564000	-2.743646000	0.097386000
O	-3.713769000	-2.343781000	-0.479200000
C	-4.950401000	-2.788796000	0.148903000
O	-2.466550000	-3.415159000	1.091891000
H	0.202421000	3.015762000	-2.009484000
H	0.855795000	1.288541000	-0.167600000
H	3.734921000	1.999622000	-2.120695000
H	2.698764000	2.705102000	-0.867958000
H	4.814845000	-0.281803000	-1.682869000
H	6.006978000	-1.628879000	0.023059000
H	5.651716000	-1.172947000	2.443238000
H	4.118117000	0.653439000	3.145730000

H	2.950532000	2.017357000	1.440179000
H	0.112196000	-0.712593000	-2.356246000
H	1.996900000	-4.262898000	0.040477000
H	2.993100000	-2.769524000	0.230299000
H	1.822236000	-3.267002000	1.487519000
H	-1.915428000	-0.122933000	-1.021872000
H	-1.732728000	0.101532000	2.576824000
H	-2.932635000	-0.359899000	1.358319000
H	-0.482793000	2.353464000	2.236623000
H	-0.813283000	4.739126000	1.645224000
H	-2.769555000	5.422013000	0.270788000
H	-4.396949000	3.710758000	-0.503748000
H	-4.066979000	1.329206000	0.083125000
H	-1.549814000	-2.453007000	-1.776157000
H	-5.744867000	-2.376975000	-0.469636000
H	-4.984308000	-3.879121000	0.157665000
H	-5.006715000	-2.407495000	1.169893000

*Mannuronic acid donor **2**; C-1,C-5-dioxolanium ion (0.0 kJ/mol)*

C	1.275379000	-1.309763000	4.203625000
O	1.791784000	-1.015888000	2.853175000
C	1.256826000	-1.588422000	1.849603000
O	0.356632000	-2.497451000	1.950607000
C	0.022835000	-2.860636000	0.487685000
C	-1.091147000	-1.935147000	-0.013900000
O	-2.288846000	-2.264111000	0.642032000
C	-3.433352000	-2.534647000	-0.208035000
C	-4.017121000	-1.297738000	-0.848192000
C	-3.803157000	-1.018178000	-2.204971000
C	-4.336749000	0.135782000	-2.788295000
C	-5.095274000	1.019890000	-2.017749000
C	-5.323406000	0.745239000	-0.664093000
C	-4.788748000	-0.406515000	-0.084885000
C	-0.694113000	-0.440498000	0.153700000
O	-0.817433000	-0.073493000	1.524029000
C	-1.589771000	1.128403000	1.790377000
C	-0.921646000	2.402483000	1.328967000
C	-1.365726000	3.068011000	0.177615000
C	-0.737542000	4.240640000	-0.254766000
C	0.341565000	4.761094000	0.463092000
C	0.787754000	4.109917000	1.619003000
C	0.158010000	2.940721000	2.047893000
C	0.785003000	-0.209529000	-0.250525000
O	0.910225000	-0.277897000	-1.643815000
C	1.863971000	0.631715000	-2.252026000
C	3.304921000	0.311031000	-1.929438000
C	3.900913000	-0.857026000	-2.432995000
C	5.225503000	-1.166805000	-2.121766000
C	5.974882000	-0.308435000	-1.308061000
C	5.394554000	0.860173000	-0.810024000
C	4.064385000	1.166042000	-1.118747000
C	1.643468000	-1.346245000	0.405156000
O	1.233699000	-2.614482000	-0.138158000
H	1.859754000	-0.670240000	4.859398000
H	1.442438000	-2.364920000	4.420313000
H	0.214746000	-1.061125000	4.225529000
H	-0.227592000	-3.919485000	0.499258000
H	-1.142452000	-2.141515000	-1.092714000
H	-4.150996000	-3.001929000	0.469720000
H	-3.151189000	-3.270474000	-0.972535000
H	-3.233668000	-1.713849000	-2.817109000
H	-4.171560000	0.334739000	-3.842879000
H	-5.522361000	1.909151000	-2.471501000
H	-5.931564000	1.419202000	-0.067900000
H	-4.980936000	-0.626037000	0.962484000
H	-1.338382000	0.170960000	-0.482898000
H	-2.575270000	1.012381000	1.327353000
H	-1.715584000	1.120967000	2.875906000
H	-2.217613000	2.679176000	-0.374953000
H	-1.097762000	4.750714000	-1.143093000
H	0.823525000	5.676883000	0.134536000
H	1.612963000	4.523115000	2.191449000
H	0.496501000	2.449616000	2.957344000
H	1.102325000	0.760414000	0.150377000
H	1.665305000	0.527915000	-3.321061000
H	1.617210000	1.656945000	-1.949159000
H	3.324177000	-1.521629000	-3.071074000
H	5.678560000	-2.068546000	-2.522631000
H	7.008716000	-0.544989000	-1.075526000
H	5.975780000	1.536677000	-0.190645000
H	3.620665000	2.083925000	-0.739993000
H	2.712470000	-1.210798000	0.249552000

*Mannuronic acid donor **2**; oxocarbenium ion (18.1 kJ/mol)*

O	0.011156000	3.615425000	-1.371369000
C	-0.933306000	2.903964000	-1.609677000
O	-2.198462000	3.289584000	-1.775859000
C	-2.459069000	4.717322000	-1.652925000
C	-0.849392000	1.383164000	-1.835045000
O	0.600181000	1.075537000	-1.756039000
C	1.100395000	0.248245000	-0.935325000
C	0.272883000	-0.637523000	-0.076296000
O	0.989274000	-1.210270000	0.973774000
C	1.553164000	-2.525415000	0.701212000
C	2.617766000	-2.503533000	-0.369466000
C	3.866106000	-1.911298000	-0.111909000
C	4.844972000	-1.865680000	-1.105306000
C	4.590946000	-2.414198000	-2.368645000
C	3.356135000	-3.009518000	-2.633273000
C	2.373909000	-3.051636000	-1.637178000
C	-0.981227000	0.162319000	0.379004000
O	-0.719842000	1.398861000	0.991924000

C	-0.072874000	1.407404000	2.285656000
C	1.432008000	1.549230000	2.189894000
C	2.256872000	0.923446000	3.133415000
C	3.640853000	1.102710000	3.095487000
C	4.221280000	1.896858000	2.099344000
C	3.407213000	2.520930000	1.152391000
C	2.016293000	2.357685000	1.200069000
C	-1.736415000	0.509005000	-0.922377000
O	-2.074364000	-0.663964000	-1.638443000
C	-3.477176000	-1.061807000	-1.600977000
C	-3.932728000	-1.552827000	-0.249488000
C	-4.749098000	-0.756935000	0.566005000
C	-5.154704000	-1.209182000	1.825803000
C	-4.747342000	-2.465298000	2.280137000
C	-3.940683000	-3.272436000	1.469462000
C	-3.539424000	-2.819597000	0.212175000
H	-2.205035000	5.050829000	-0.645768000
H	-1.867529000	5.263593000	-2.388897000
H	-3.524014000	4.826861000	-1.846307000
H	-1.099168000	1.166463000	-2.876532000
H	2.189745000	0.213054000	-0.935844000
H	-0.143126000	-1.403125000	-0.760774000
H	1.958499000	-2.831743000	1.667199000
H	0.738527000	-3.209317000	0.430095000
H	4.068386000	-1.489091000	0.869048000
H	5.809562000	-1.414582000	-0.893245000
H	5.357062000	-2.385489000	-3.137284000
H	3.158694000	-3.447568000	-3.606836000
H	1.420922000	-3.534001000	-1.843200000
H	-1.588034000	-0.497512000	1.009918000
H	-0.506152000	2.273064000	2.796733000
H	-0.346439000	0.506566000	2.845671000
H	1.816326000	0.293184000	3.901690000
H	4.267284000	0.622487000	3.841492000
H	5.297611000	2.037287000	2.072708000
H	3.845984000	3.157324000	0.389299000
H	1.389129000	2.889803000	0.491511000
H	-2.622684000	1.098741000	-0.669658000
H	-3.533014000	-1.851904000	-2.352529000
H	-4.089277000	-0.217069000	-1.939007000
H	-5.086826000	0.213600000	0.209748000
H	-5.794509000	-0.586800000	2.443885000
H	-5.068203000	-2.822002000	3.254046000
H	-3.640986000	-4.258471000	1.811584000
H	-2.930415000	-3.457948000	-0.423634000

*Glucuronic acid donor **6**; C-1,C-5-dioxolanium ion (0.0 kJ/mol)*

C	1.515790000	-0.688409000	4.365477000
O	1.914498000	-0.598836000	2.948806000
C	1.318234000	-1.322670000	2.086602000
O	0.435942000	-2.204018000	2.388462000
C	-0.011063000	-2.772961000	1.006679000
C	-1.193952000	-1.918446000	0.530754000
O	-1.575250000	-2.516326000	-0.687707000
C	-2.994163000	-2.749411000	-0.863897000
C	-3.805965000	-1.484252000	-1.019583000
C	-4.730156000	-1.098969000	-0.039313000
C	-5.484763000	0.069987000	-0.189721000
C	-5.318028000	0.865366000	-1.325324000
C	-4.398809000	0.487136000	-2.311930000
C	-3.650139000	-0.680679000	-2.161046000
C	-0.786066000	-0.423004000	0.375433000
O	-0.887832000	0.173577000	1.672743000
C	-1.622071000	1.429173000	1.734132000
C	-0.918213000	2.591398000	1.074943000
C	-1.323546000	3.052951000	-0.185340000
C	-0.662247000	4.121844000	-0.798794000
C	0.410883000	4.742264000	-0.154923000
C	0.817834000	4.295875000	1.107682000
C	0.155150000	3.229602000	1.717268000
C	0.672501000	-0.222142000	-0.117210000
O	0.746400000	-0.401439000	-1.501279000
C	1.687568000	0.442671000	-2.210131000
C	3.137234000	0.106360000	-1.943369000
C	3.966706000	1.004905000	-1.258251000
C	5.305557000	0.686787000	-1.003496000
C	5.824188000	-0.538048000	-1.429174000
C	5.003988000	-1.440965000	-2.116725000
C	3.670840000	-1.119308000	-2.375054000
C	1.580421000	-1.281881000	0.598633000
O	1.134494000	-2.605074000	0.261094000
H	1.688436000	-1.705959000	4.716171000
H	2.162339000	0.023129000	4.871719000
H	0.464718000	-0.411467000	4.442769000
H	-0.235841000	-3.825689000	1.162990000
H	-1.992085000	-1.965849000	1.282150000
H	-3.039178000	-3.361372000	-1.767628000
H	-3.371531000	-3.347528000	-0.023675000
H	-4.878428000	-1.724690000	0.837929000
H	-6.207681000	0.349431000	0.570863000
H	-5.910495000	1.766726000	-1.450466000
H	-4.280923000	1.093101000	-3.205530000
H	-2.944490000	-0.977373000	-2.932637000
H	-1.465946000	0.042540000	-0.341444000
H	-2.612086000	1.271237000	1.291090000
H	-1.746884000	1.603831000	2.805597000
H	-2.169668000	2.586211000	-0.683648000
H	-0.992469000	4.475583000	-1.770852000
H	0.918204000	5.579141000	-0.625208000
H	1.637804000	4.789782000	1.620546000
H	0.462298000	2.899863000	2.707107000
H	0.994234000	0.781246000	0.187107000
H	1.433068000	0.277206000	-3.259569000
H	1.481852000	1.491884000	-1.962627000
H	3.570014000	1.965035000	-0.936246000
H	5.940971000	1.396655000	-0.482386000
H	6.864156000	-0.785036000	-1.238496000
H	5.408007000	-2.388216000	-2.461128000
H	3.037837000	-1.819710000	-2.913672000
H	2.631479000	-1.173844000	0.336290000

*Glucuronic acid donor **6**; oxocarbenium ion (4.5 kJ/mol)*

O	-0.956408000	-2.426939000	1.936616000
C	0.165848000	-2.787646000	1.660349000
O	1.074413000	-3.291832000	2.483240000
C	0.707957000	-3.379867000	3.889774000
C	0.741772000	-2.732806000	0.238905000
O	-0.398810000	-2.649021000	-0.701110000
C	-0.892923000	-1.518377000	-0.980278000
C	-0.508876000	-0.226169000	-0.347630000
O	-0.728796000	0.820029000	-1.256963000
C	-2.034079000	1.472437000	-1.190551000
C	-3.160706000	0.591326000	-1.674491000
C	-4.046994000	-0.004231000	-0.764950000
C	-5.062561000	-0.856298000	-1.214081000
C	-5.198857000	-1.120271000	-2.578534000
C	-4.323431000	-0.525086000	-3.495342000
C	-3.312106000	0.325716000	-3.045896000
C	0.975658000	-0.205652000	0.197850000
O	1.045467000	0.004480000	1.586653000
C	1.091511000	1.372606000	2.049775000
C	-0.262579000	2.040357000	2.164766000
C	-1.320485000	1.403536000	2.834731000
C	-2.548363000	2.049992000	2.993678000
C	-2.730586000	3.345612000	2.496362000
C	-1.681585000	3.987188000	1.833451000
C	-0.456225000	3.333779000	1.663722000
C	1.672228000	-1.552510000	-0.081694000
O	1.973820000	-1.671924000	-1.457021000
C	3.366326000	-1.473830000	-1.836335000
C	3.865144000	-0.062630000	-1.644932000
C	3.422946000	0.966070000	-2.492850000
C	3.877508000	2.273172000	-2.316193000
C	4.786525000	2.567659000	-1.292956000
C	5.239419000	1.550355000	-0.450150000
C	4.779171000	0.241319000	-0.626716000
H	-0.185000000	-3.996880000	3.998481000
H	1.565263000	-3.840894000	4.375156000
H	0.525873000	-2.378154000	4.281516000
H	1.212543000	-3.679238000	-0.024697000
H	-1.650447000	-1.541875000	-1.764337000
H	-1.150552000	-0.156607000	0.550570000
H	-2.204318000	1.808189000	-0.162408000
H	-1.906560000	2.346950000	-1.830939000
H	-3.954775000	0.211112000	0.296939000
H	-5.746717000	-1.305065000	-0.500427000
H	-5.989431000	-1.776141000	-2.929814000
H	-4.438345000	-0.714868000	-4.558210000
H	-2.640827000	0.794260000	-3.761372000
H	1.491628000	0.578506000	-0.365081000
H	1.563356000	1.297611000	3.034113000
H	1.756337000	1.953543000	1.399005000
H	-1.179244000	0.404282000	3.237802000
H	-3.356628000	1.551064000	3.520351000
H	-3.681041000	3.852559000	2.632002000
H	-1.814421000	4.994016000	1.449133000
H	0.355481000	3.838501000	1.145635000
H	2.555799000	-1.628786000	0.560749000
H	3.378406000	-1.761183000	-2.889716000
H	3.985532000	-2.184875000	-1.275443000
H	2.726277000	0.738954000	-3.295780000
H	3.537058000	3.059114000	-2.983619000
H	5.150092000	3.582686000	-1.164521000
H	5.956842000	1.770550000	0.334528000
H	5.150392000	-0.550190000	0.020359000
